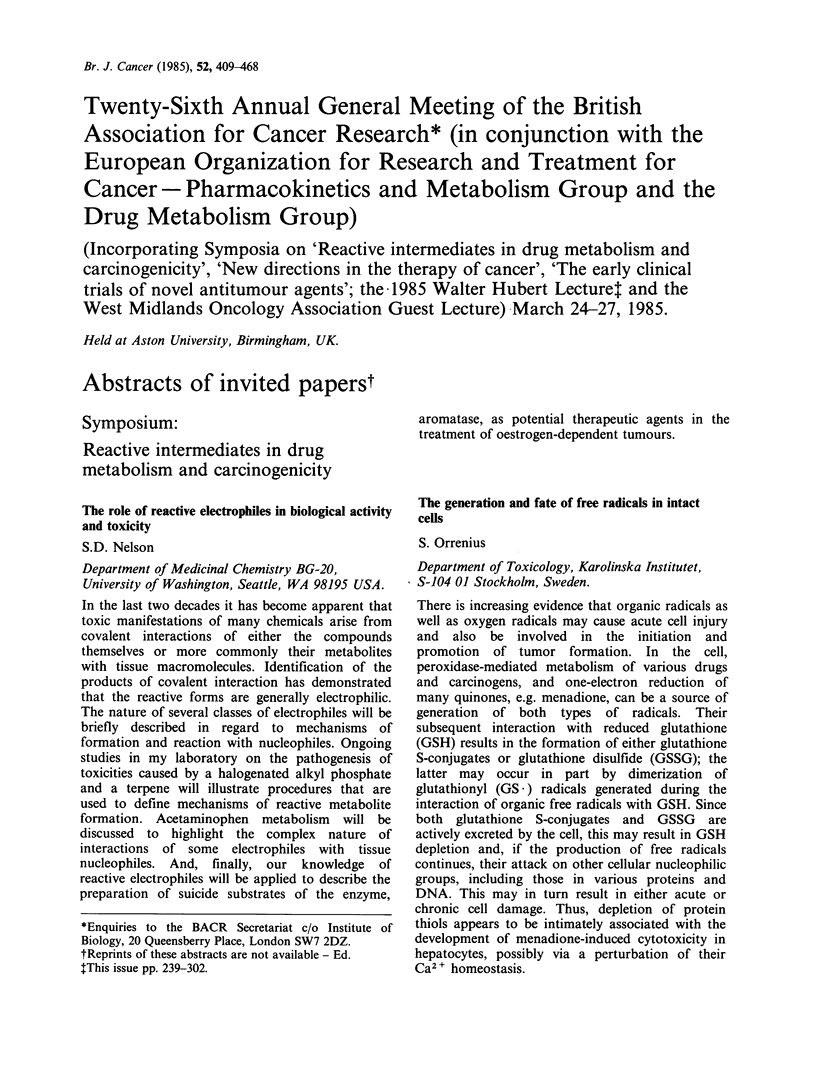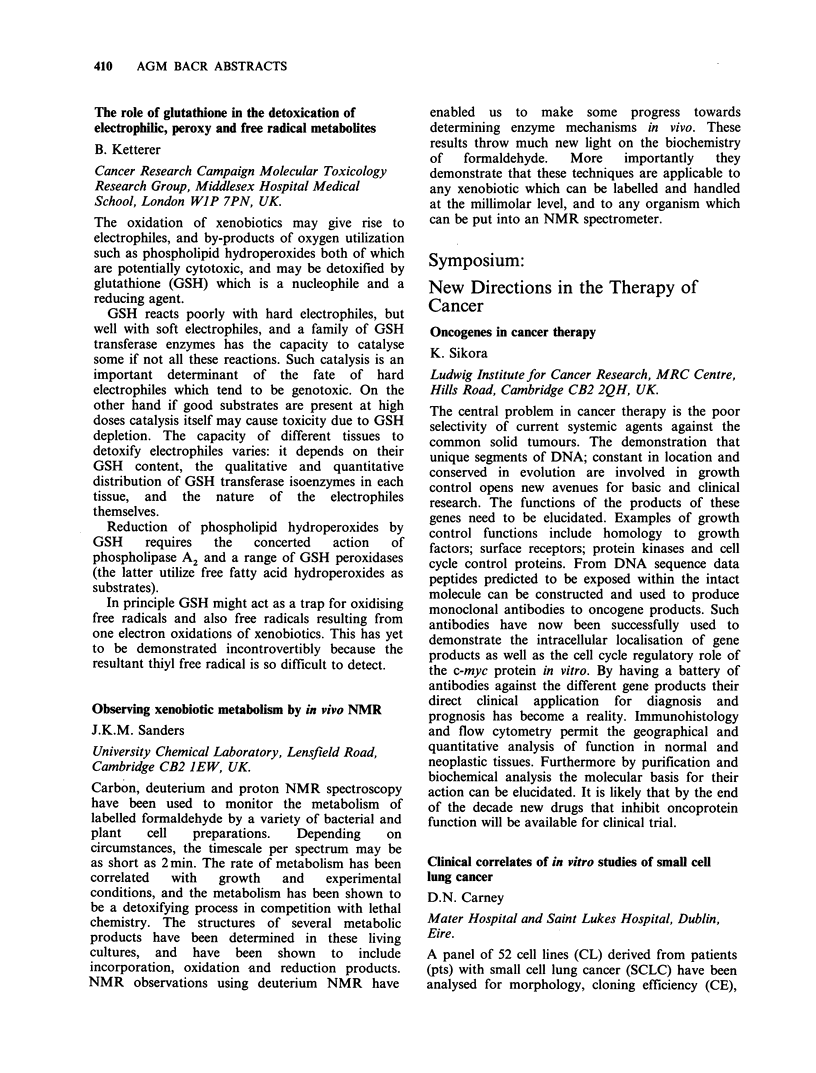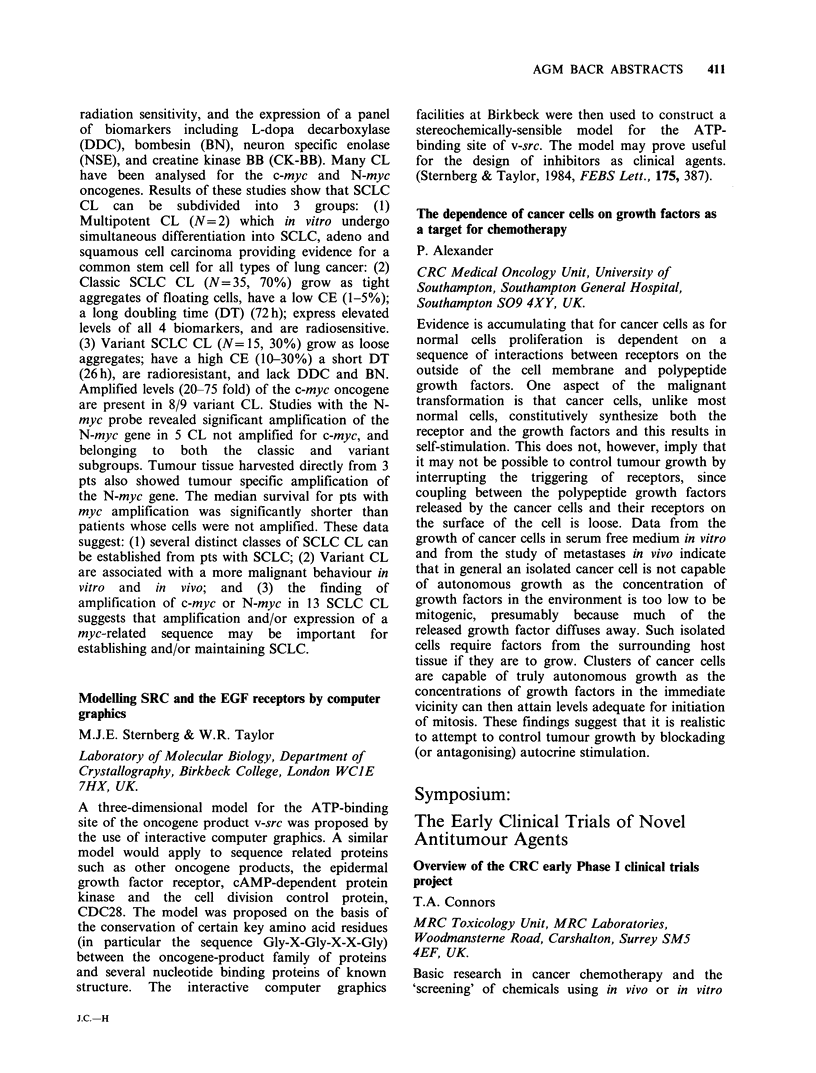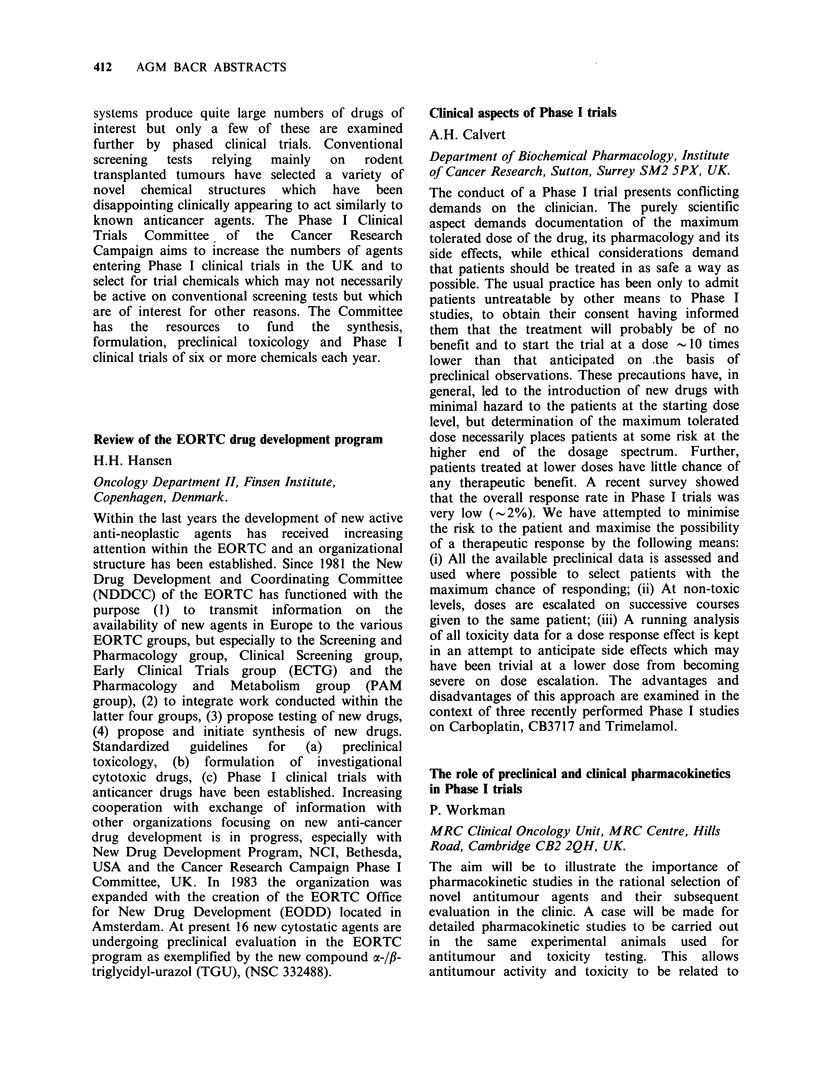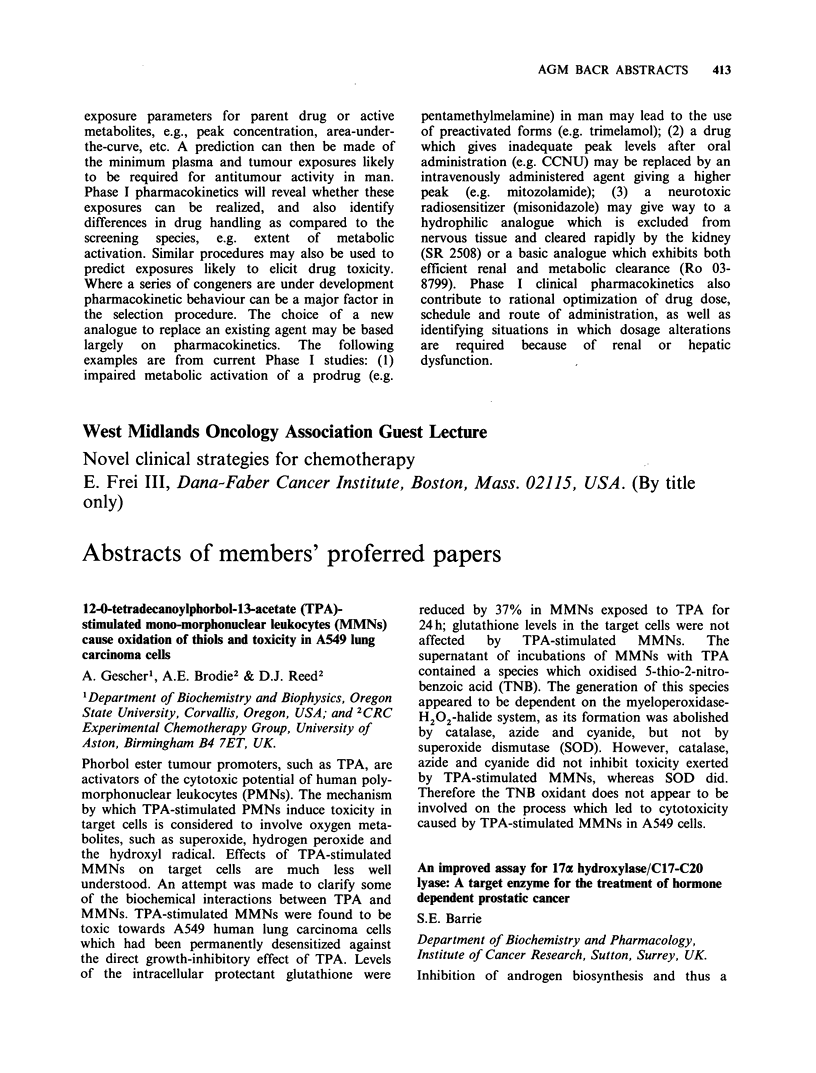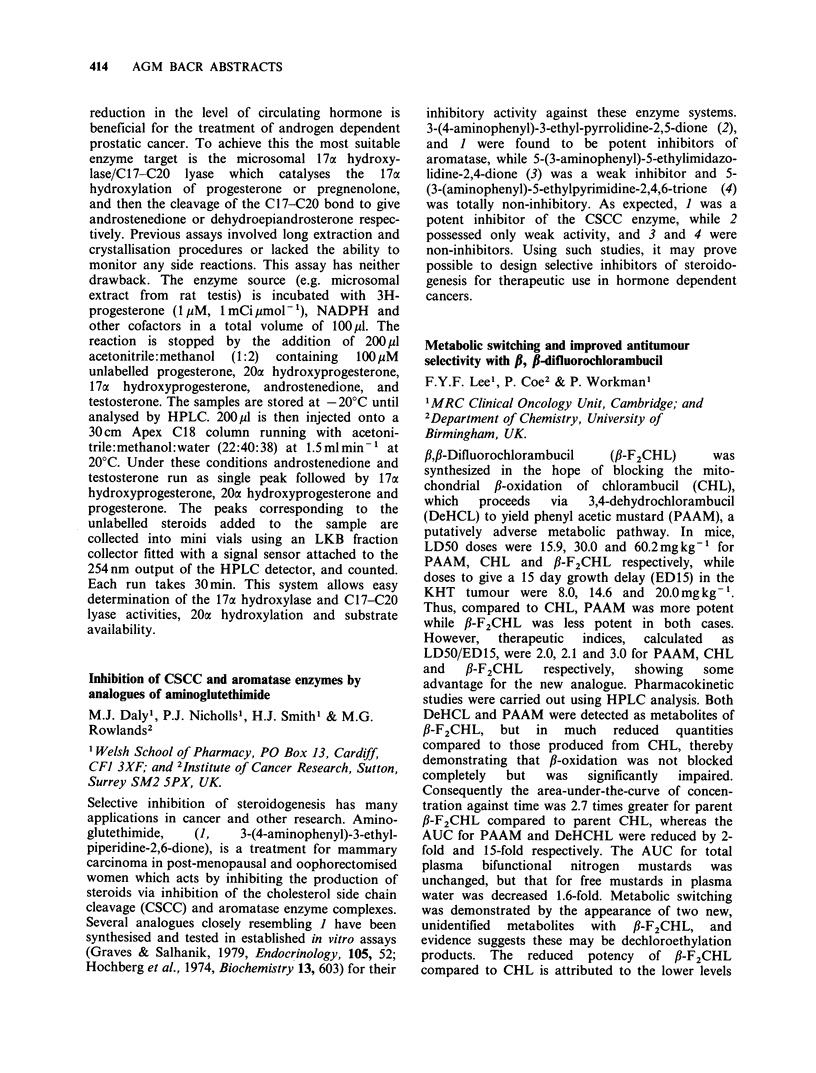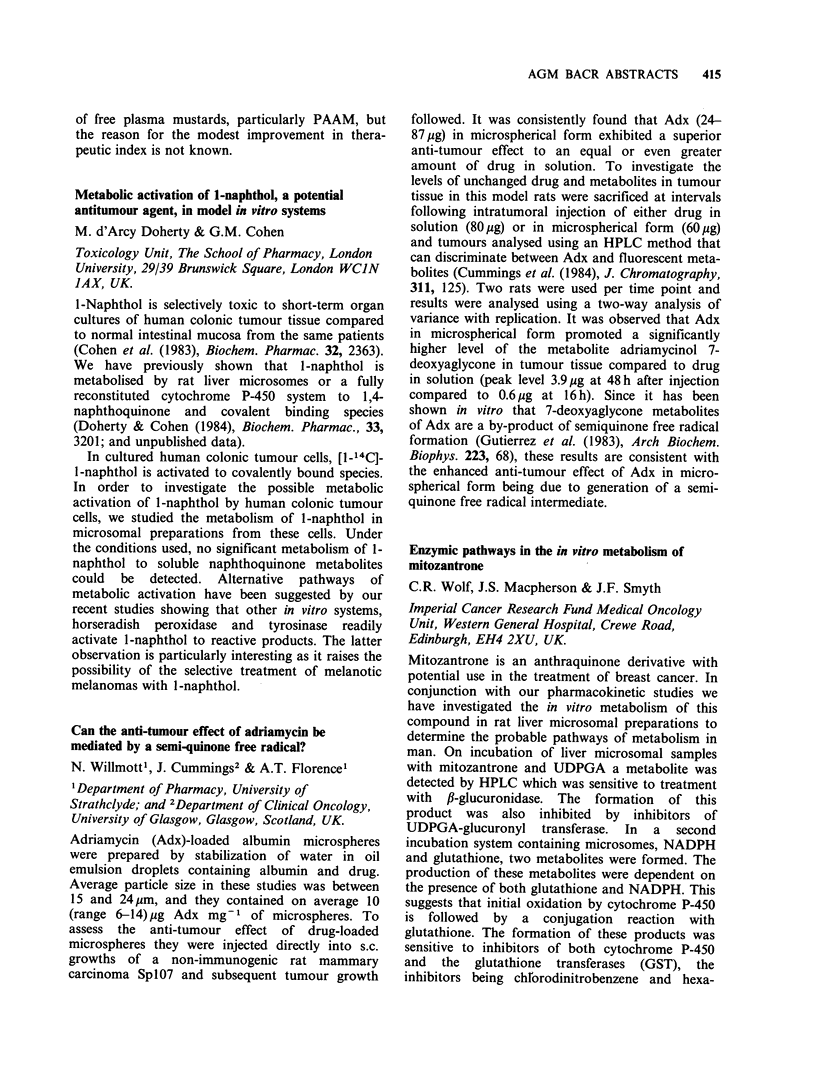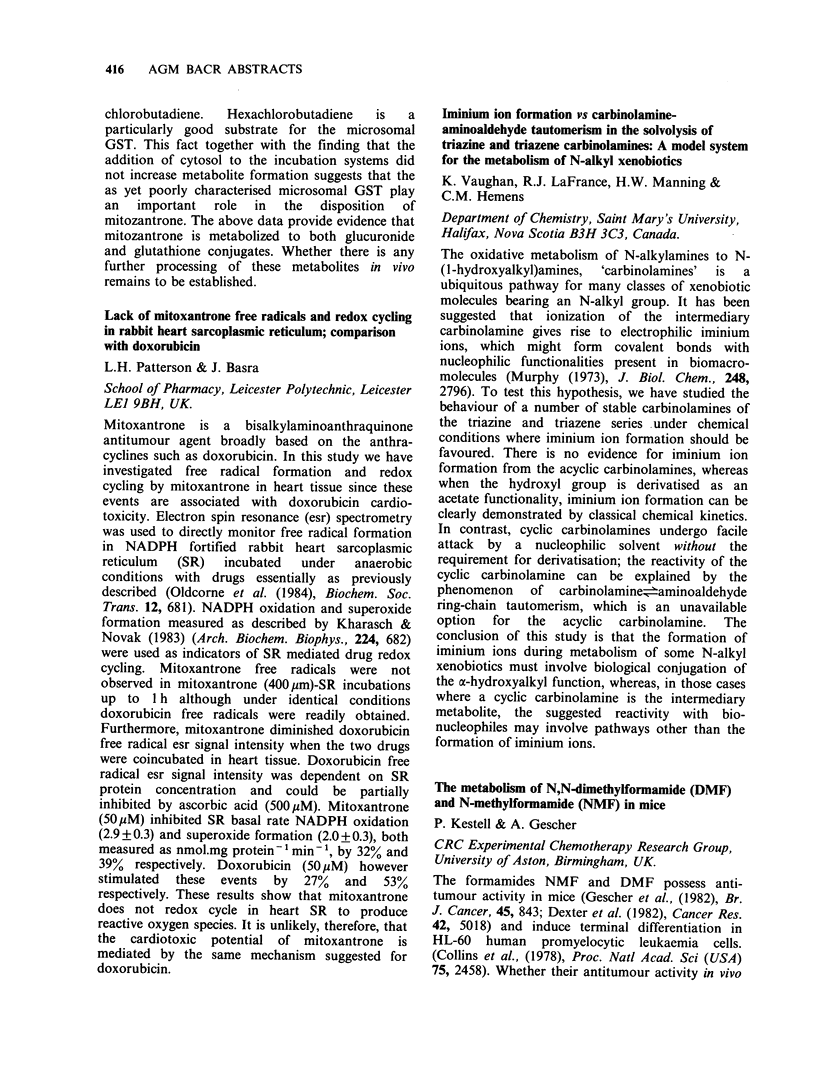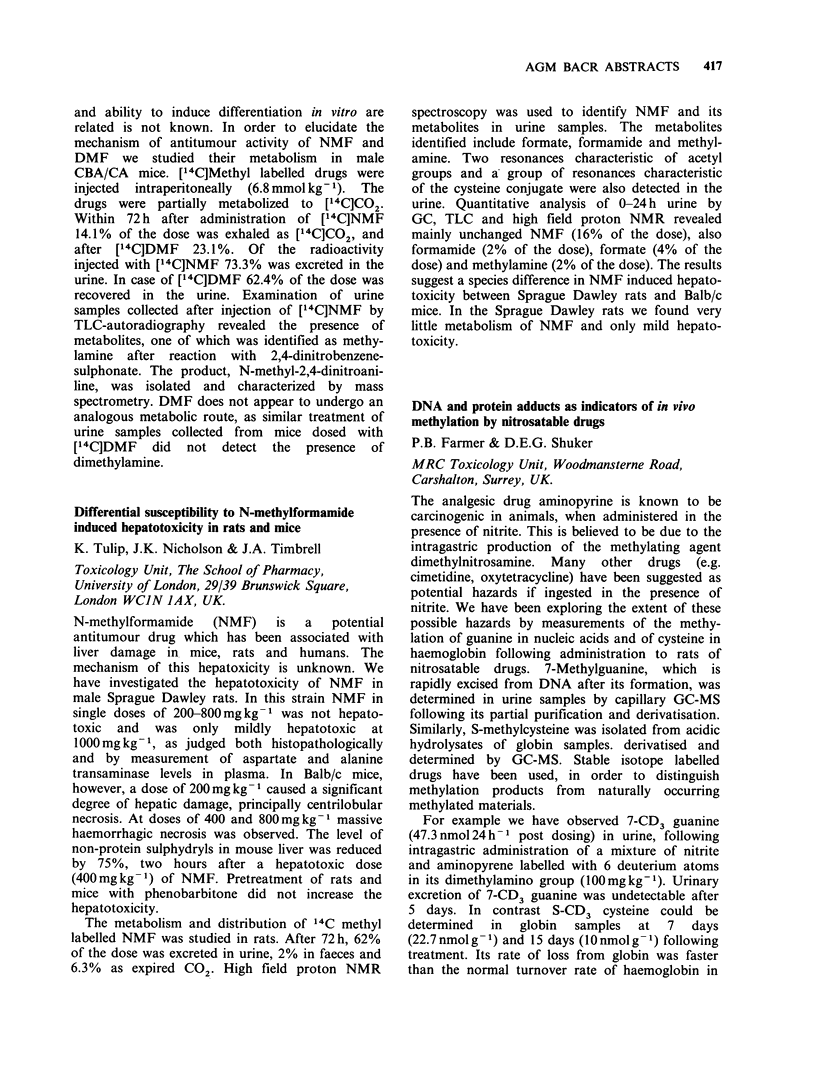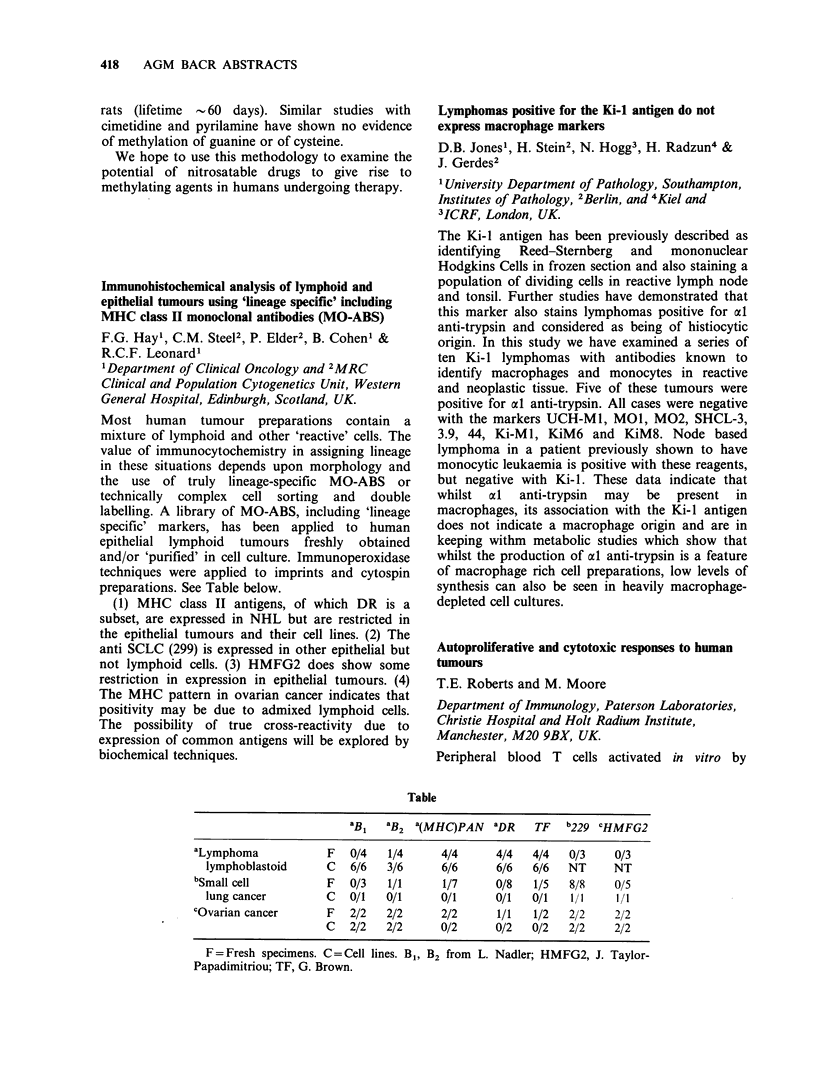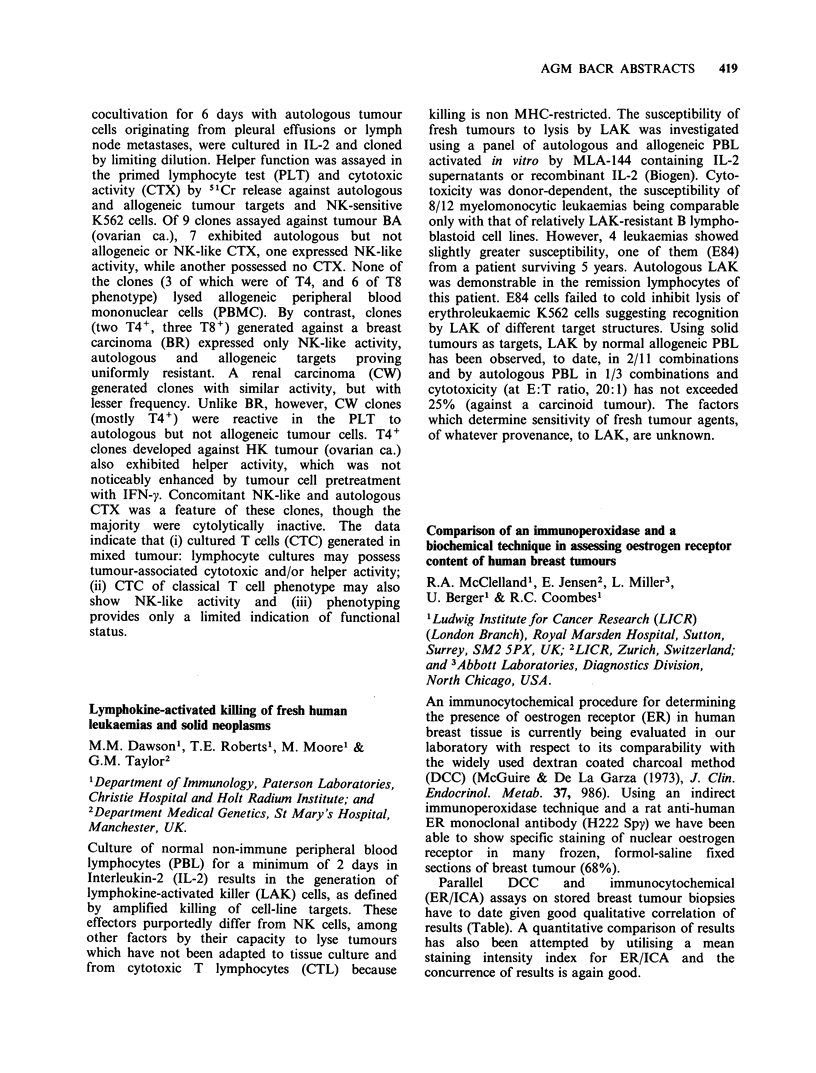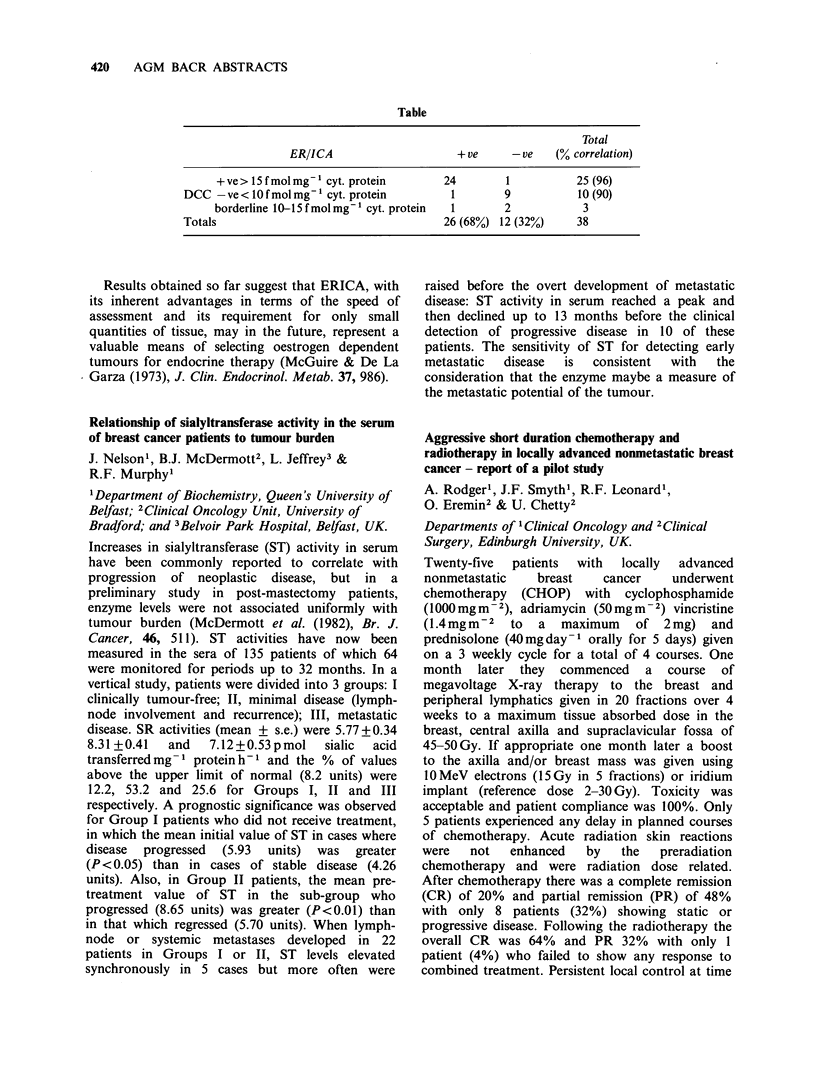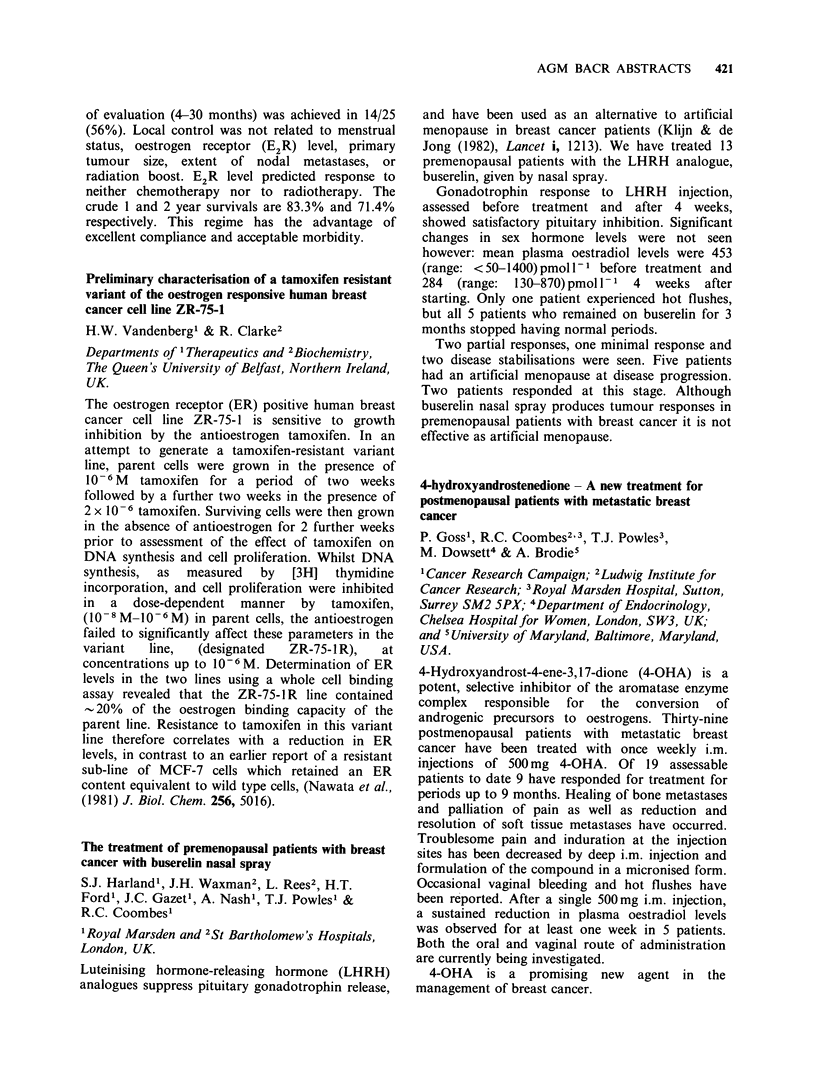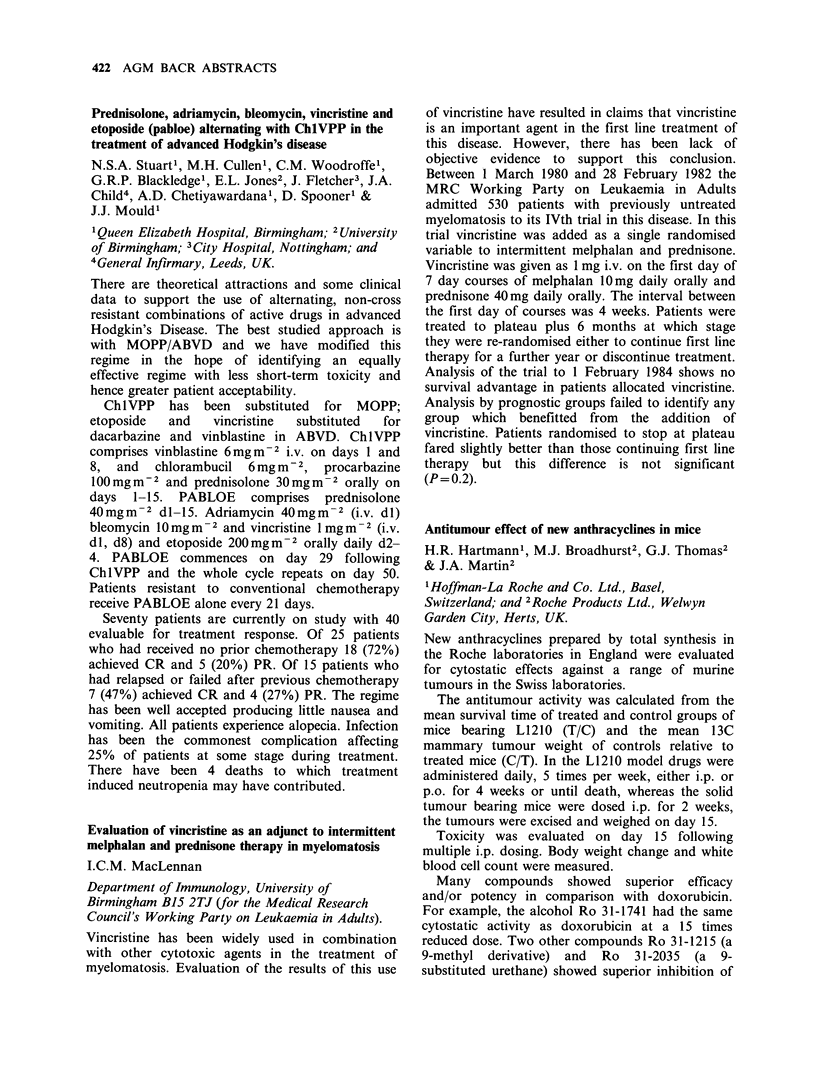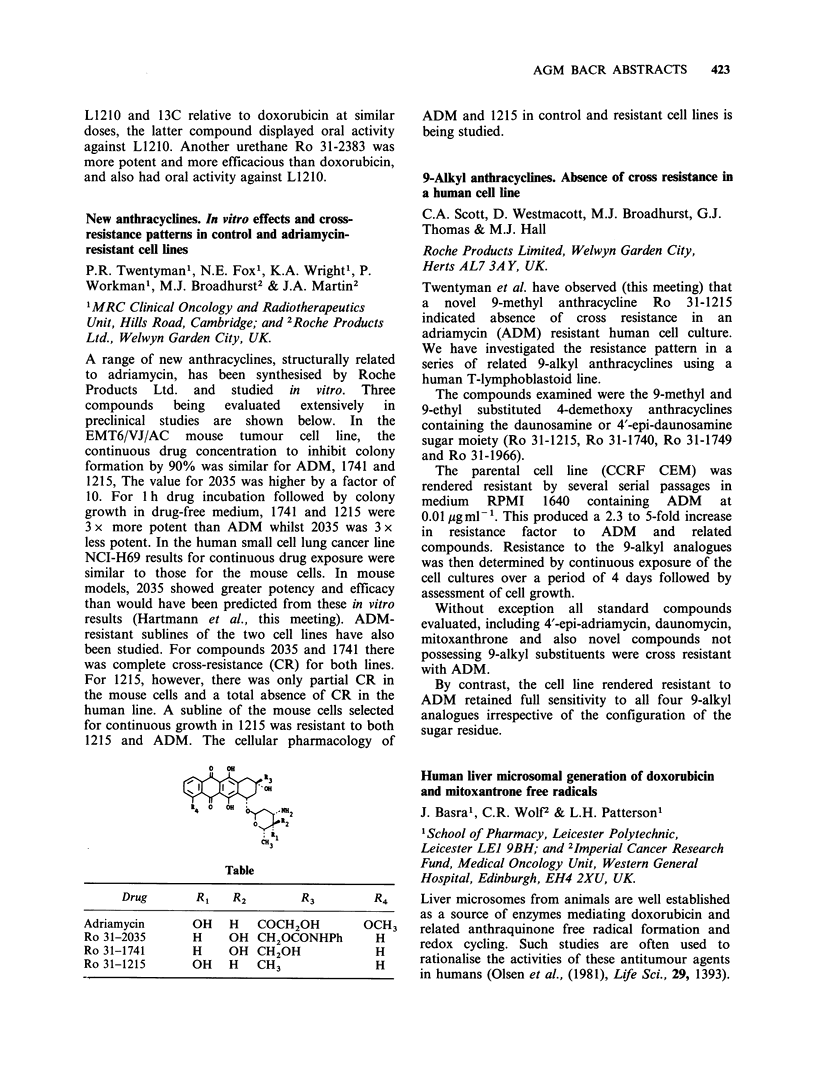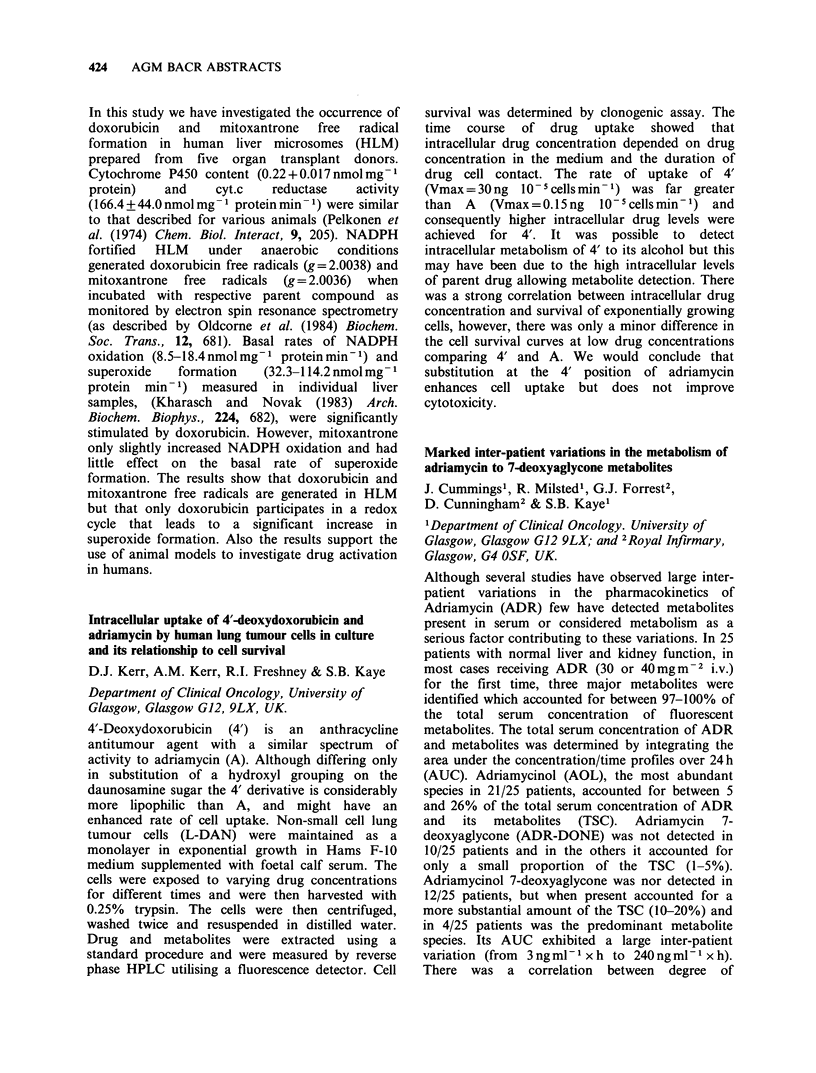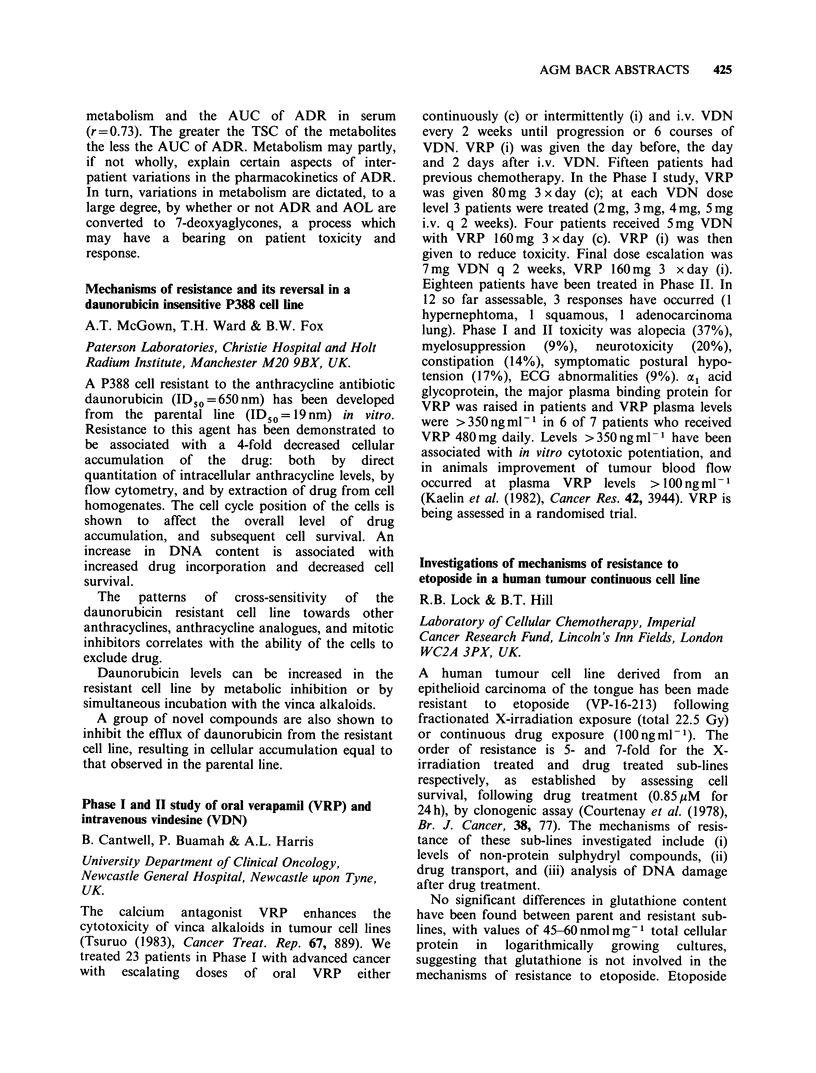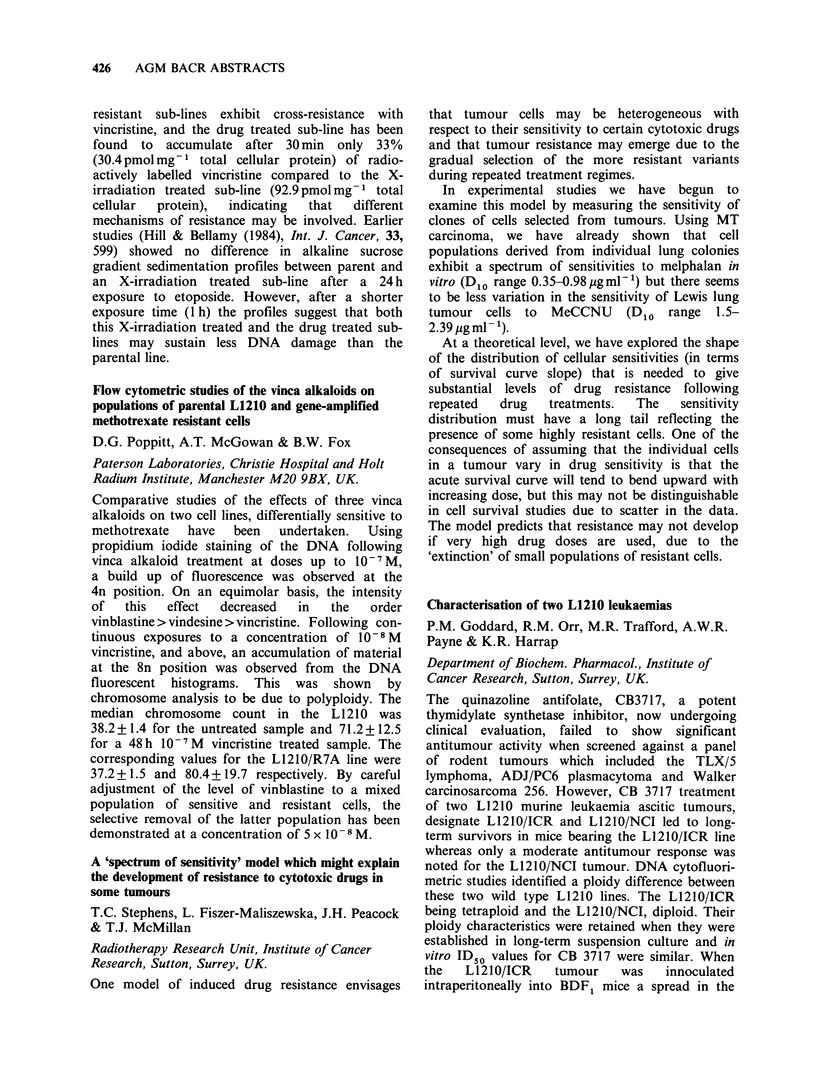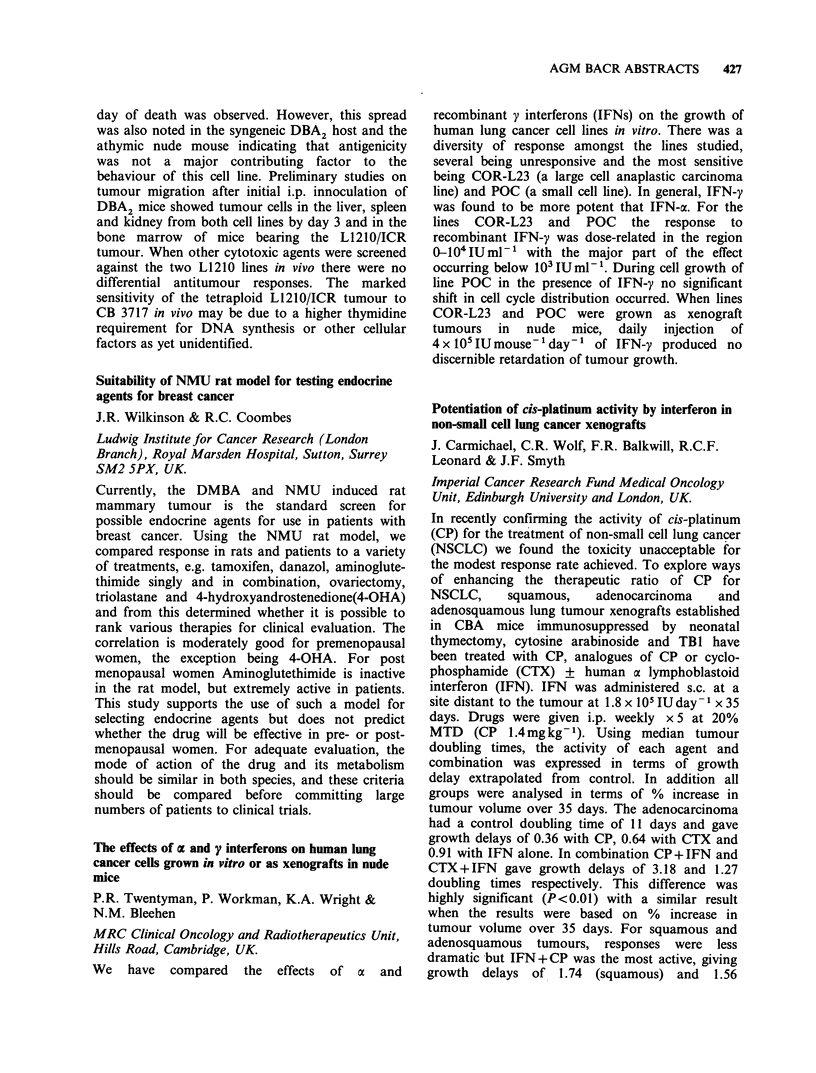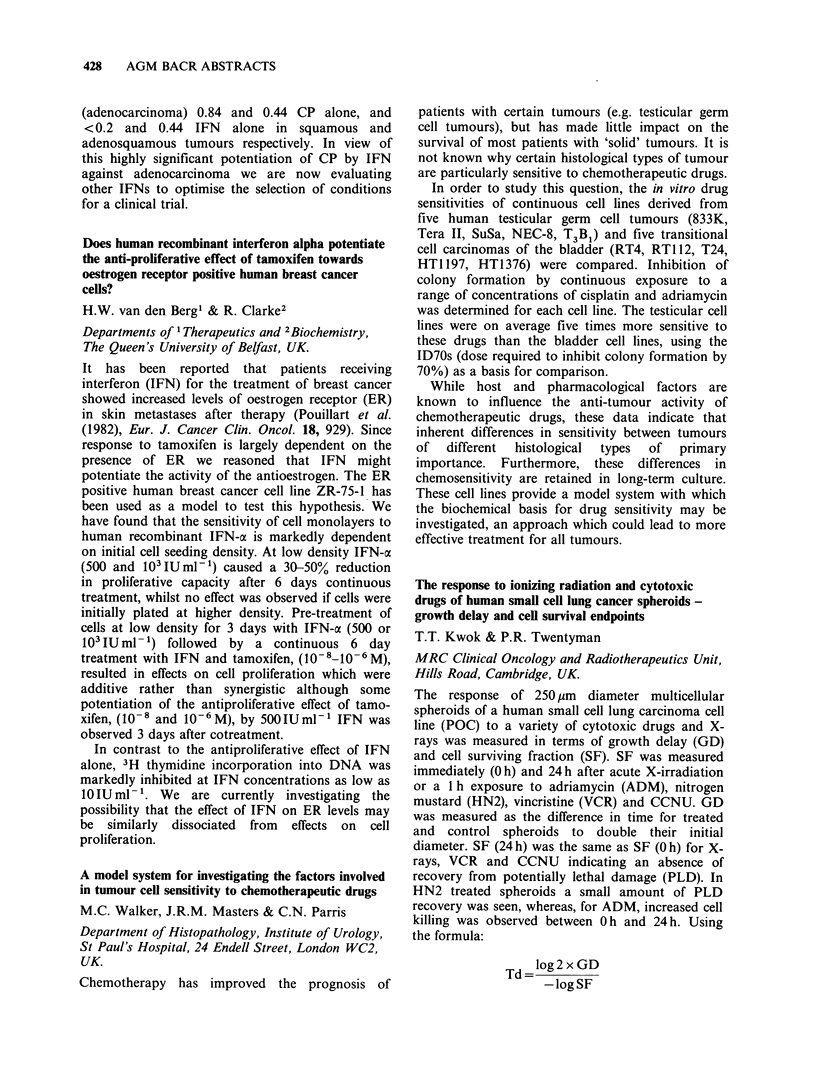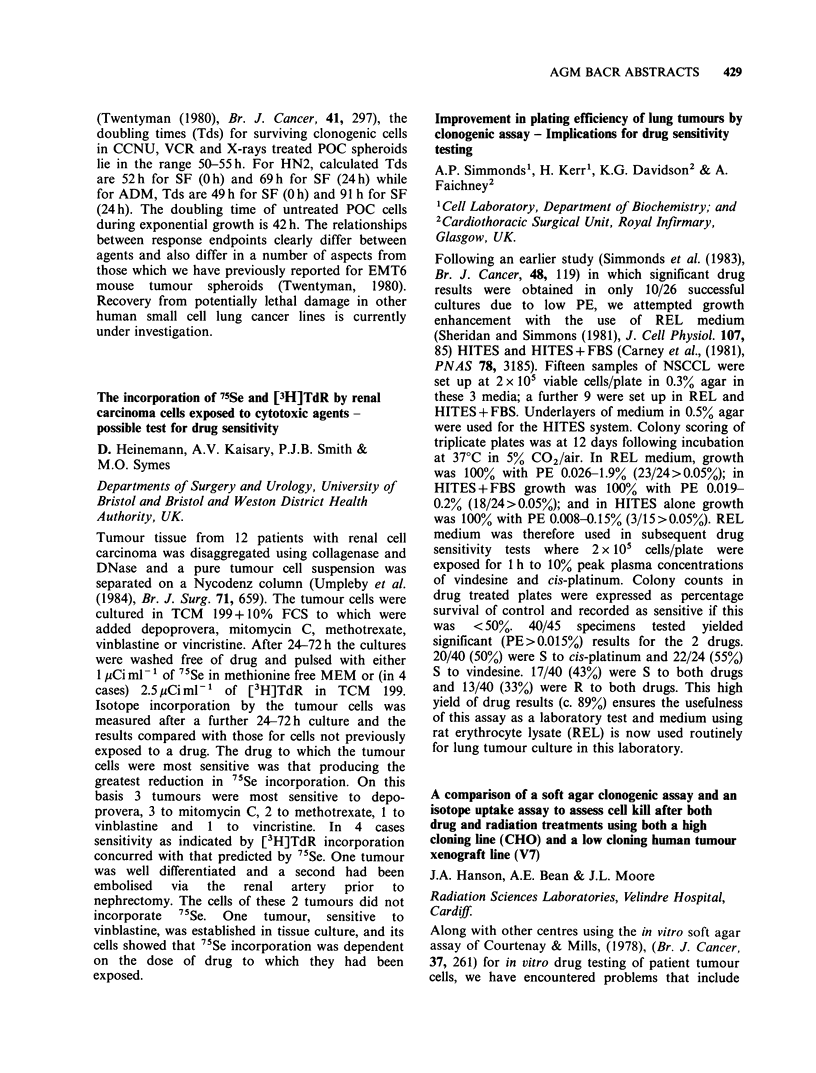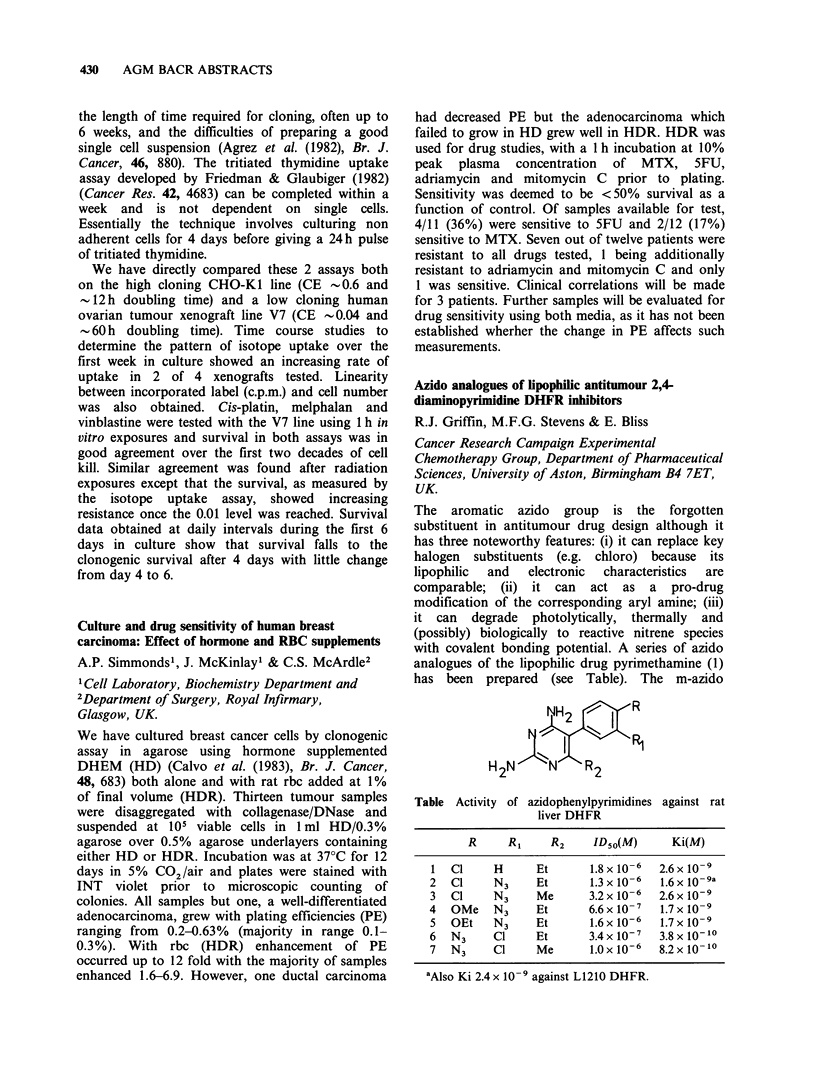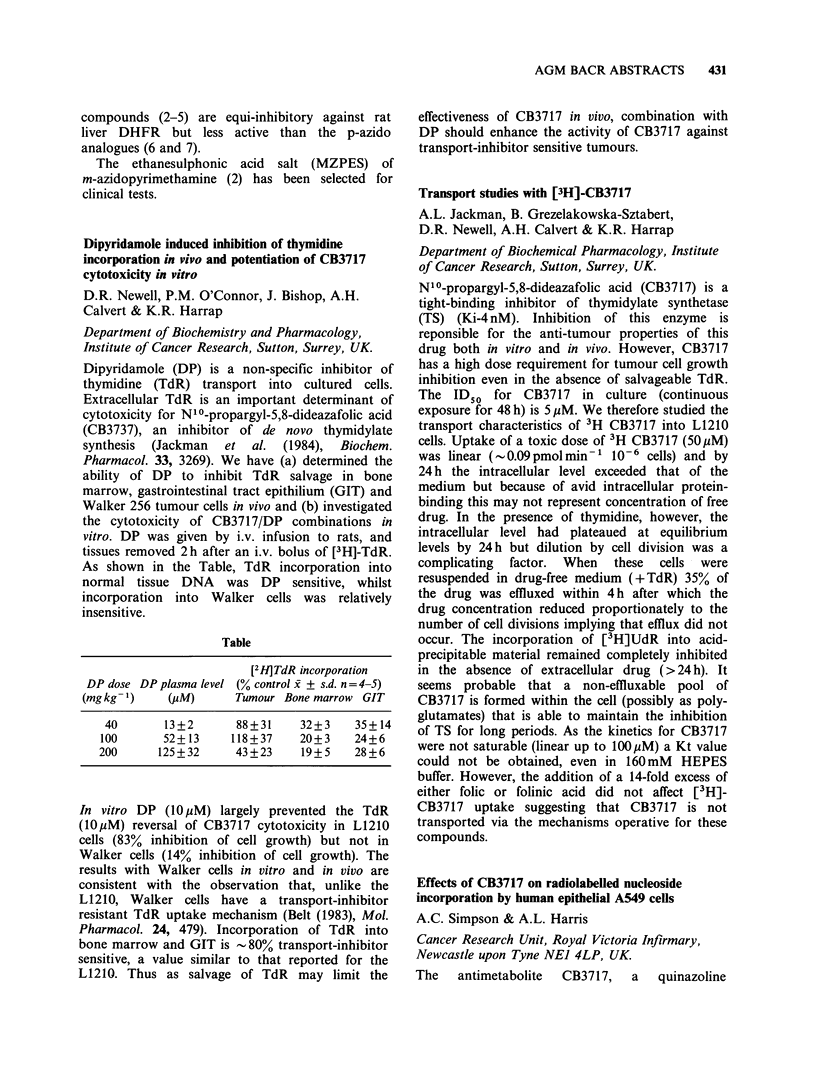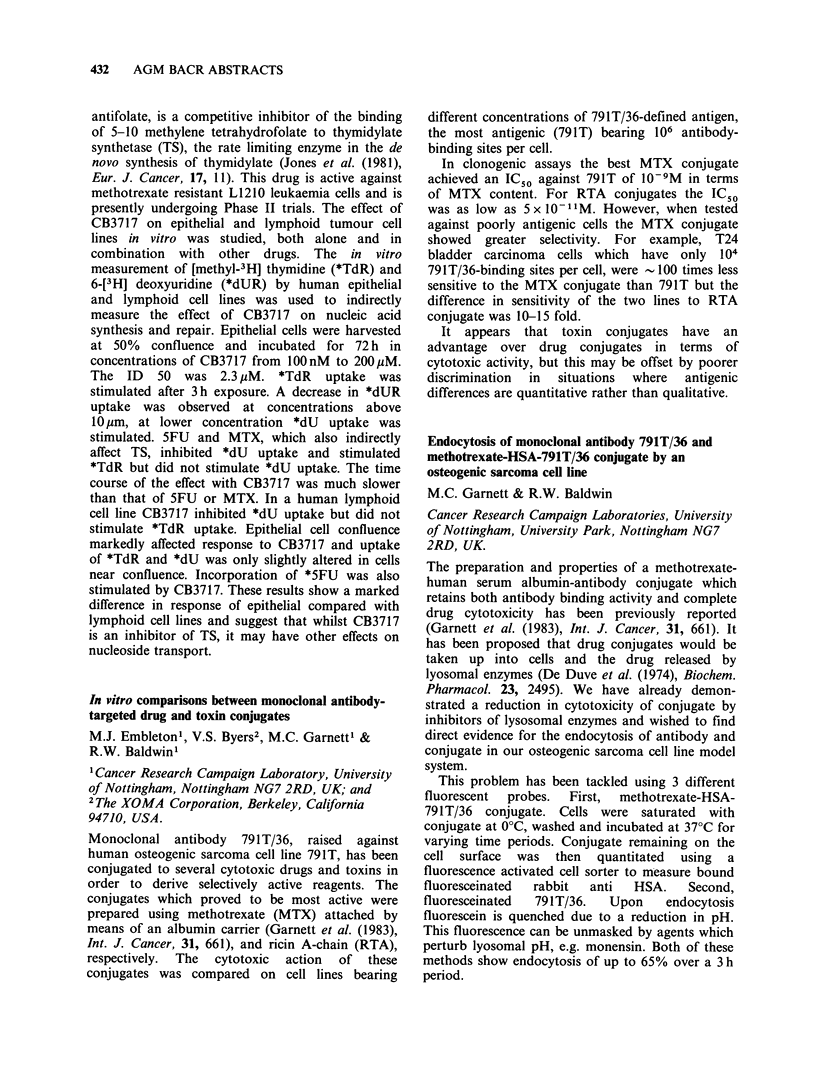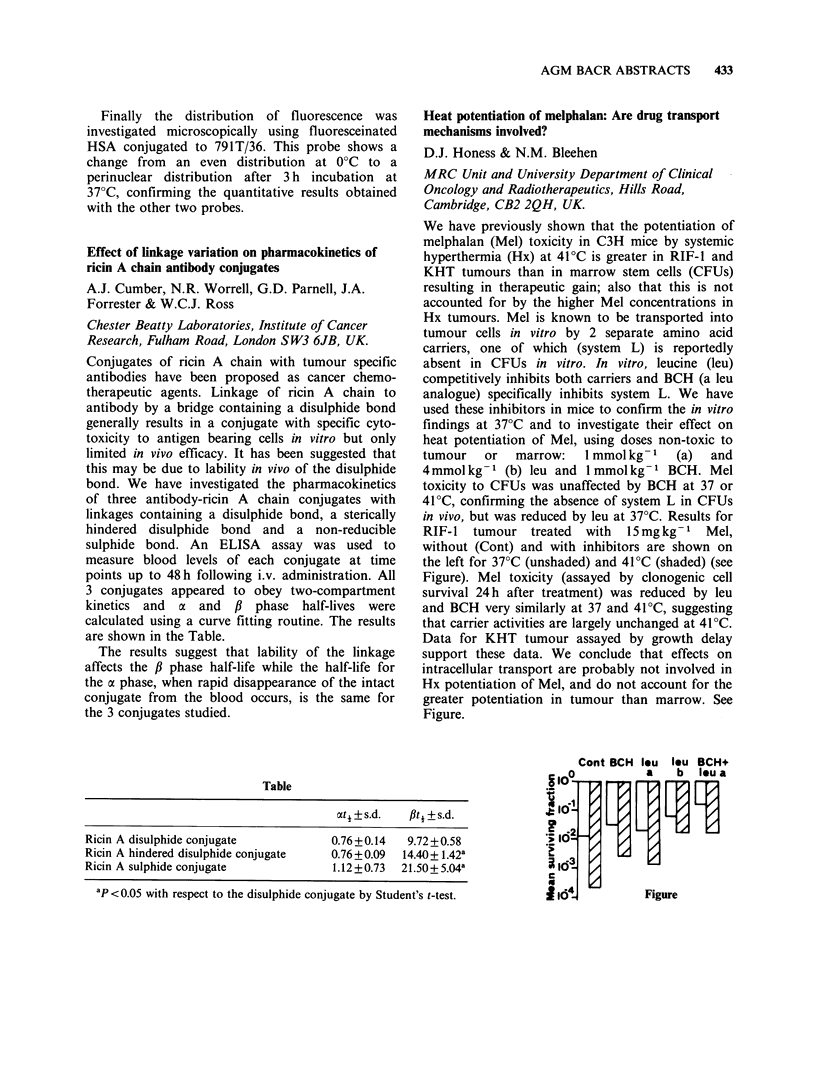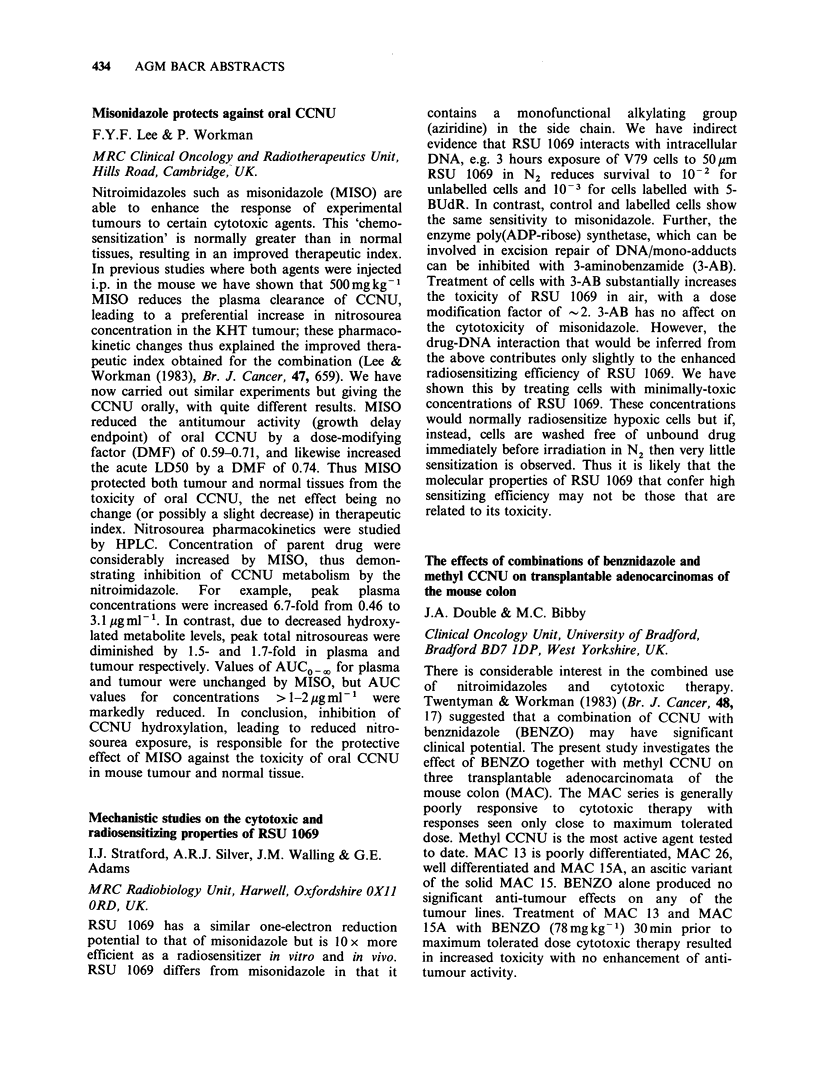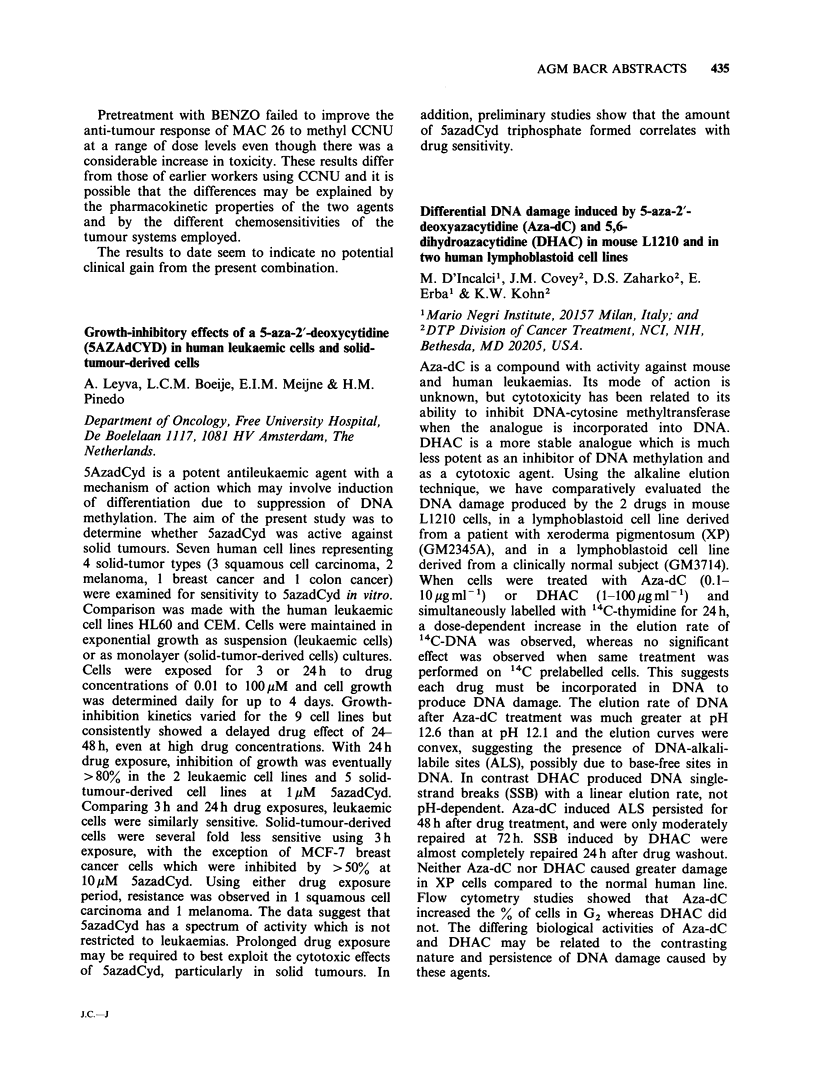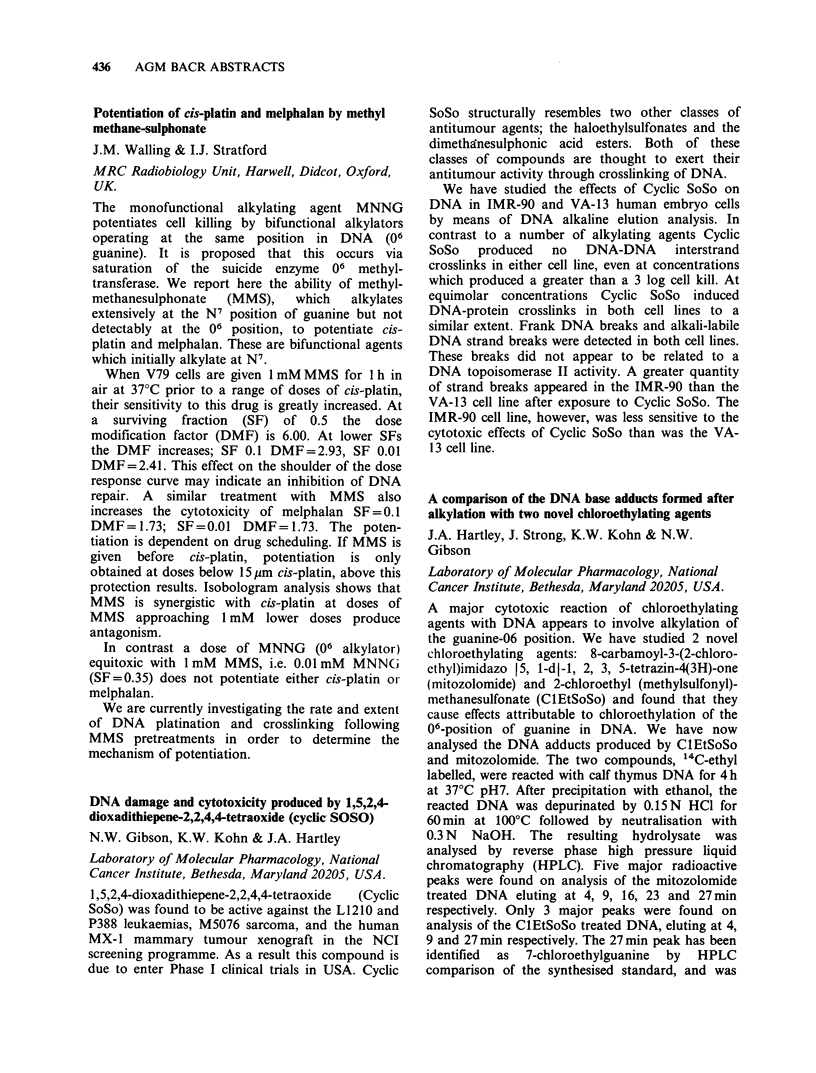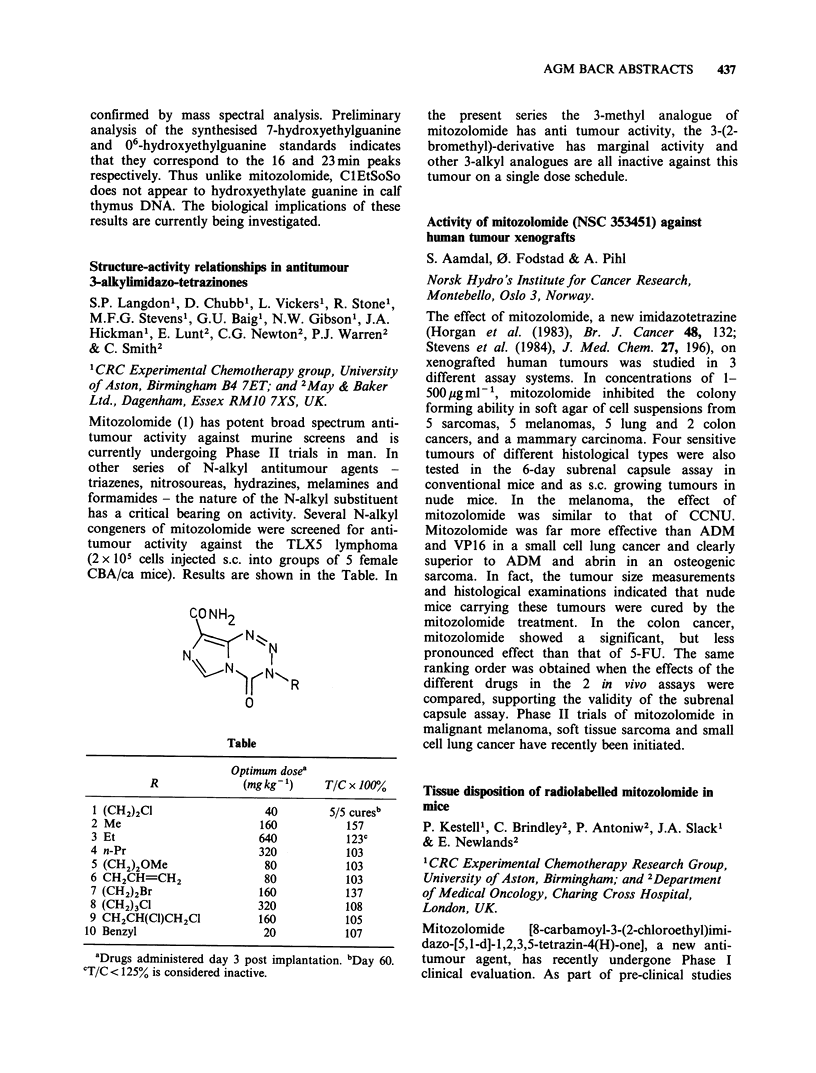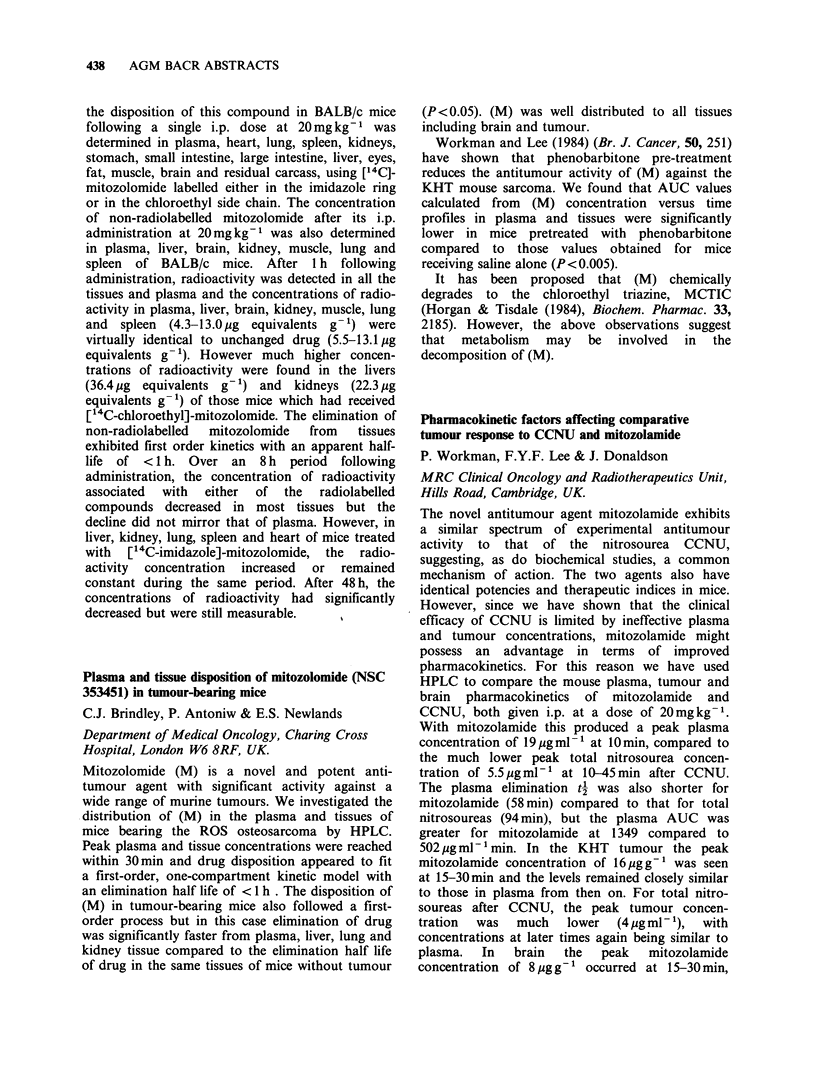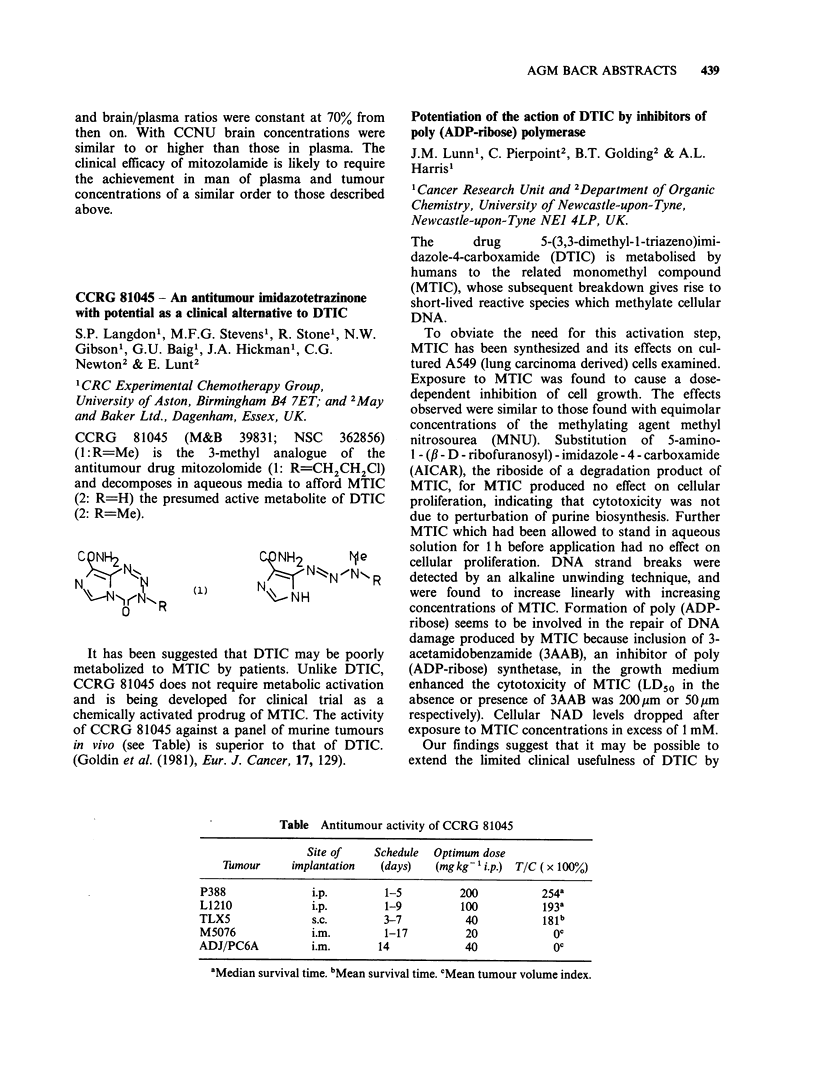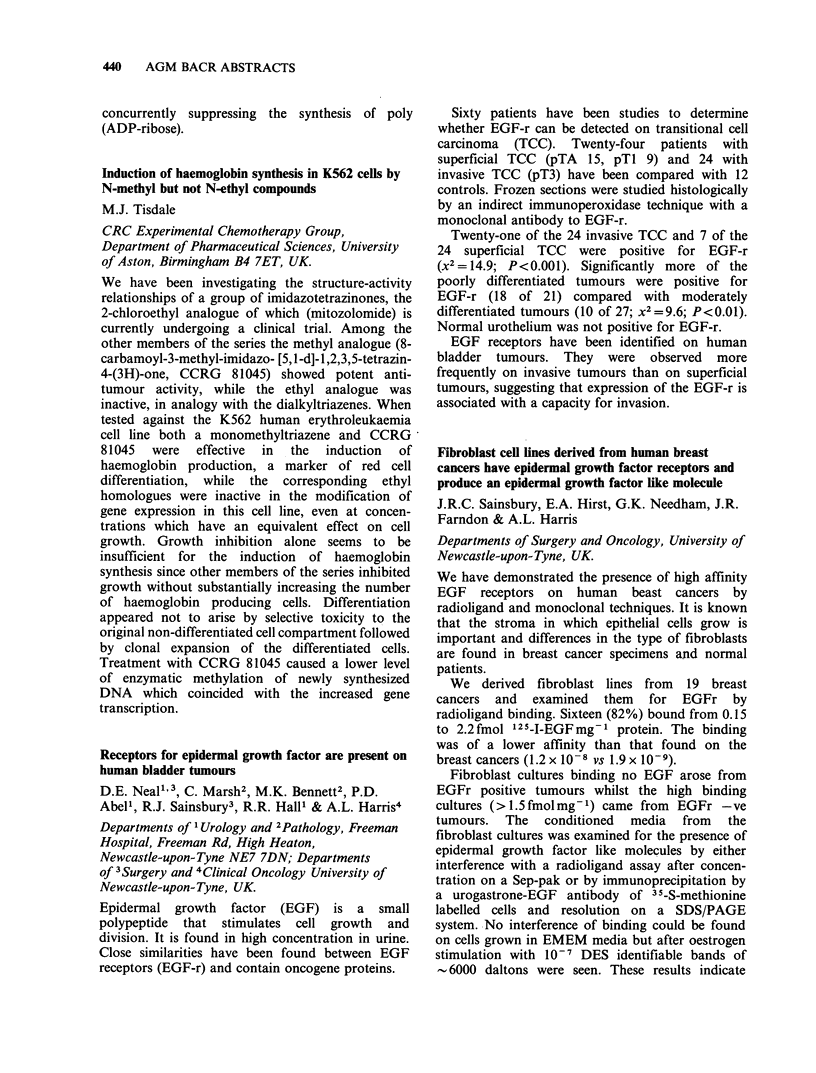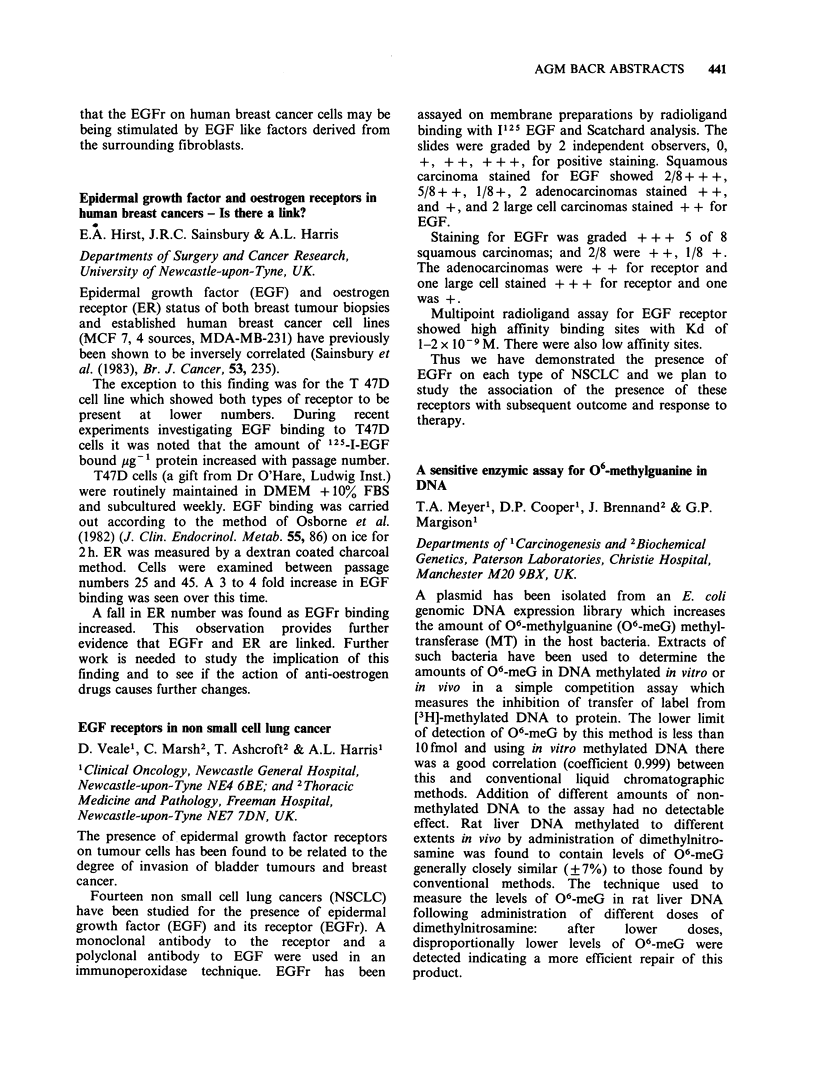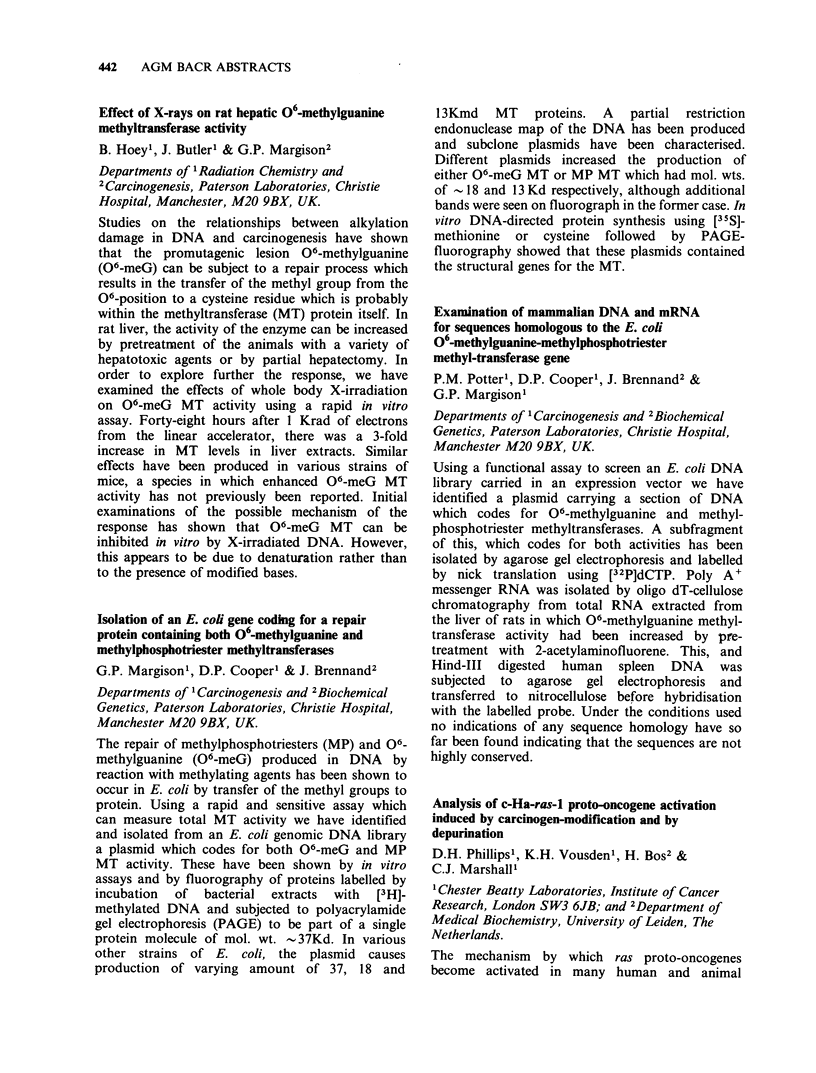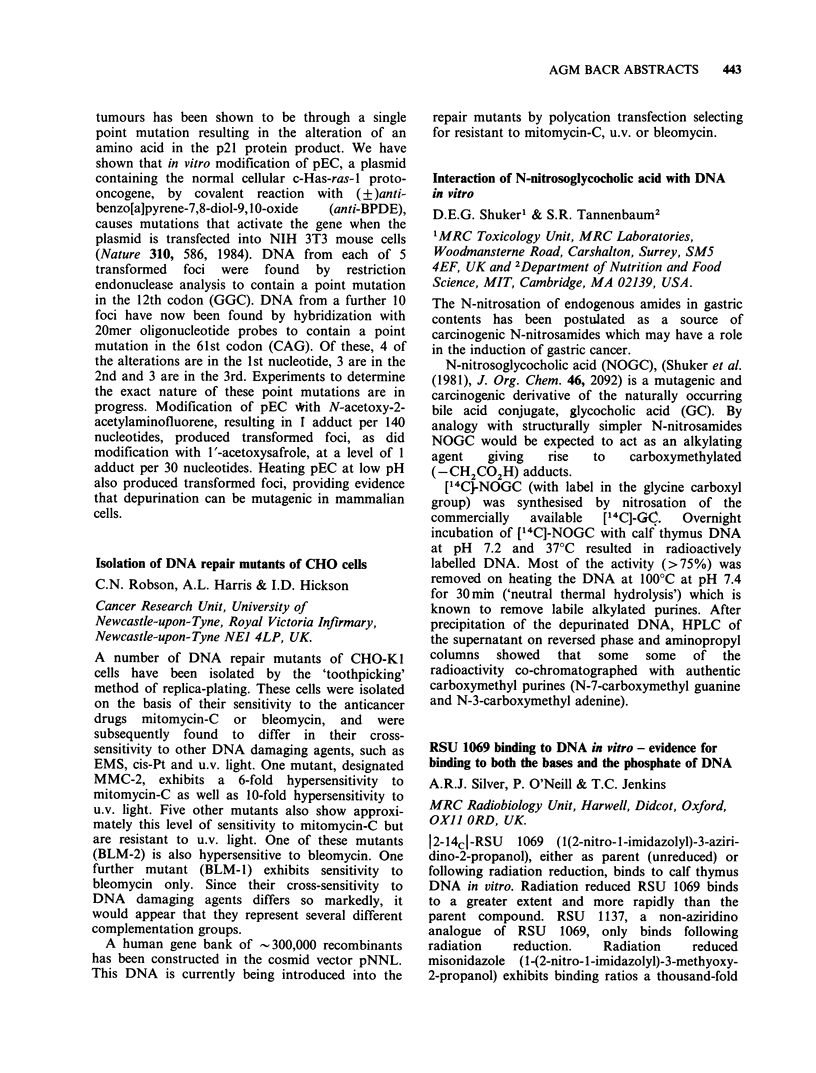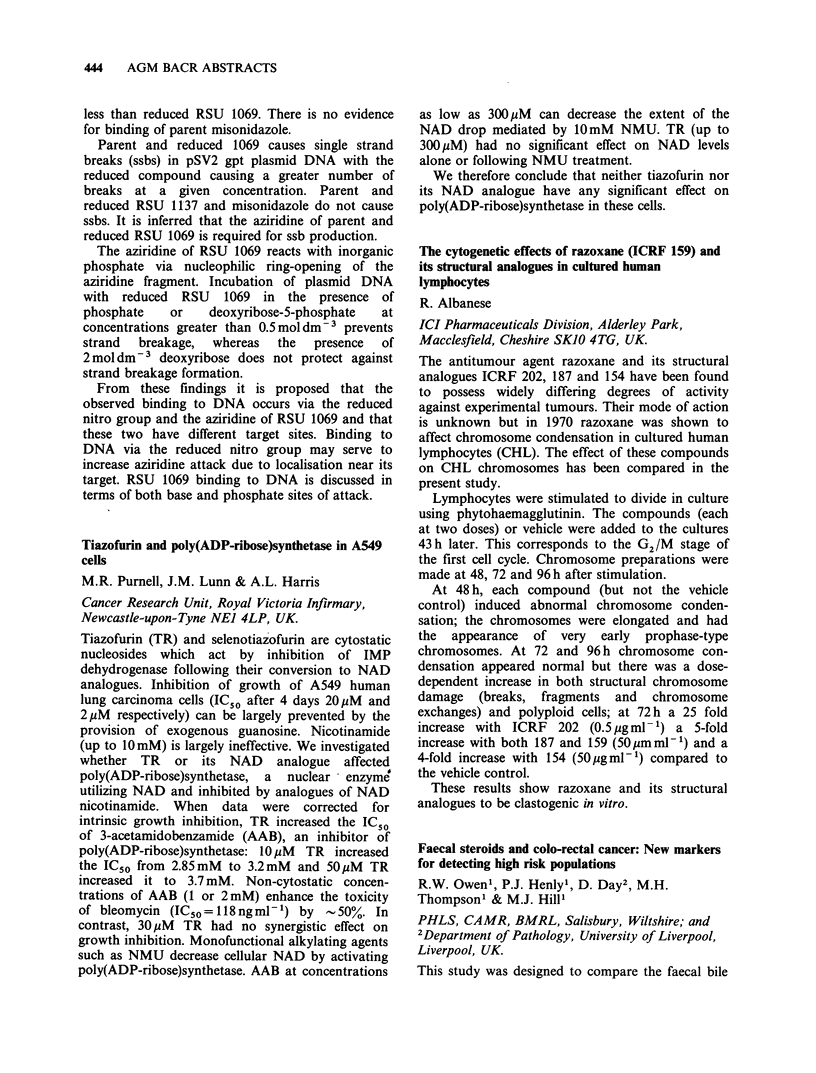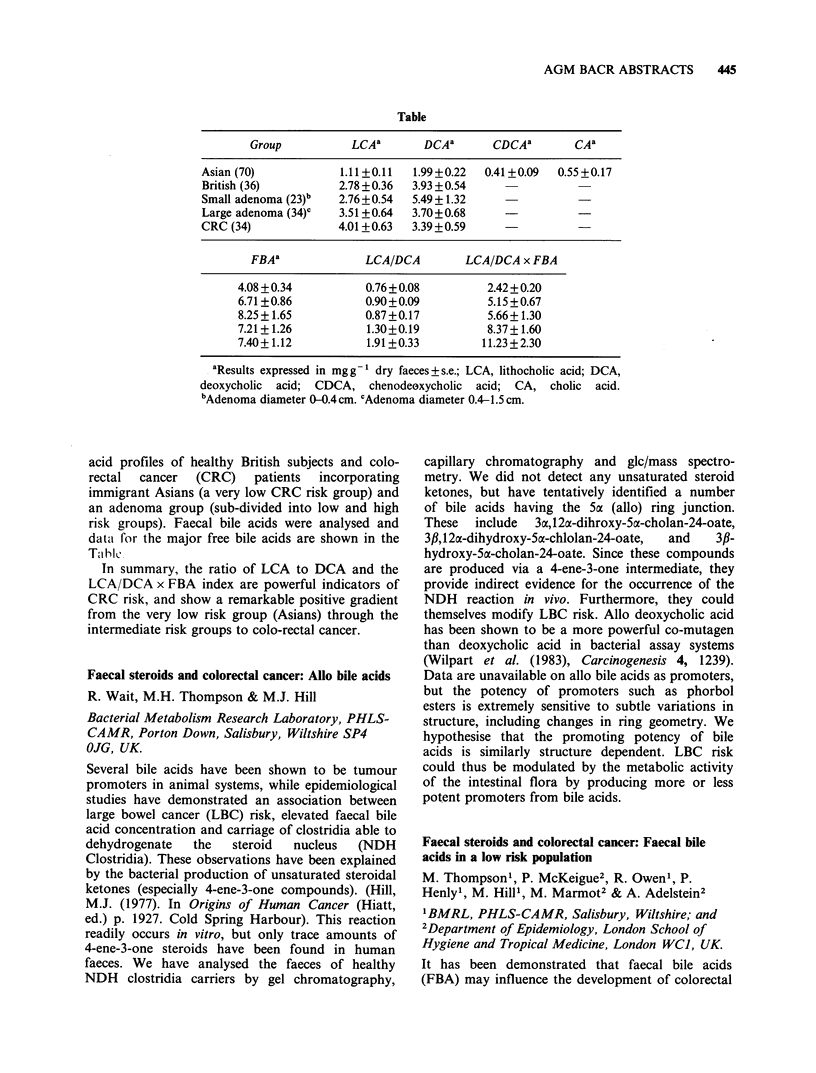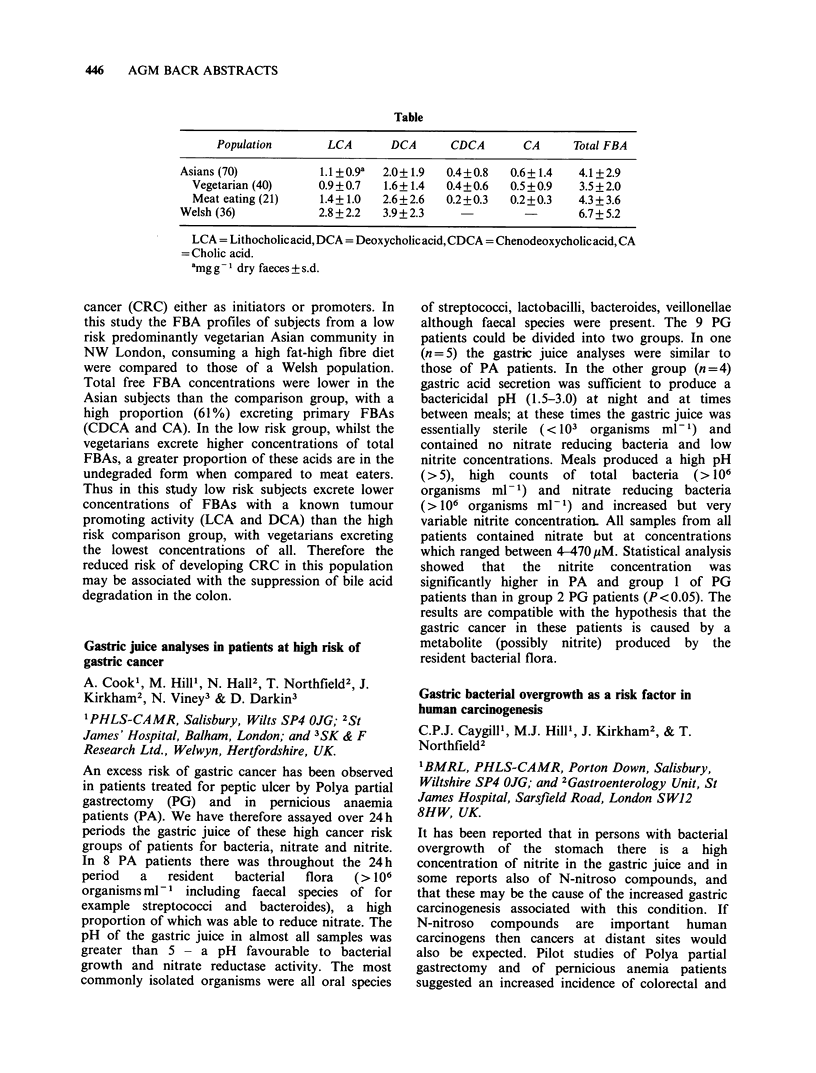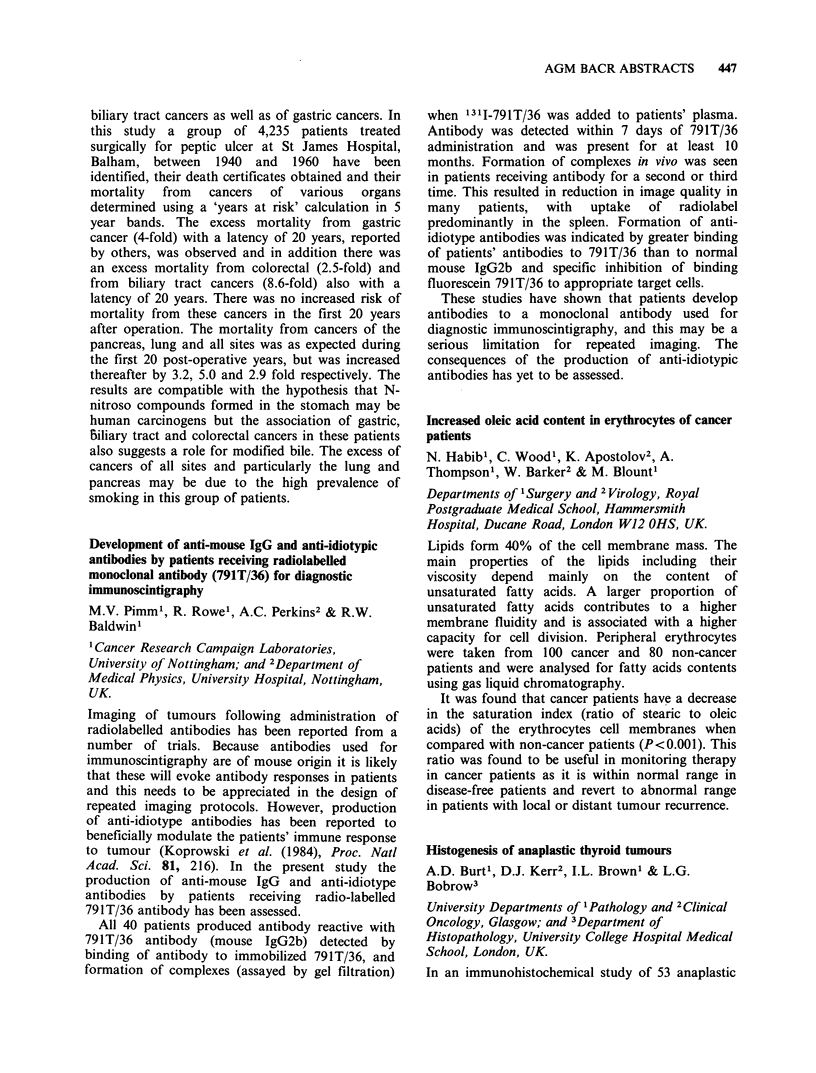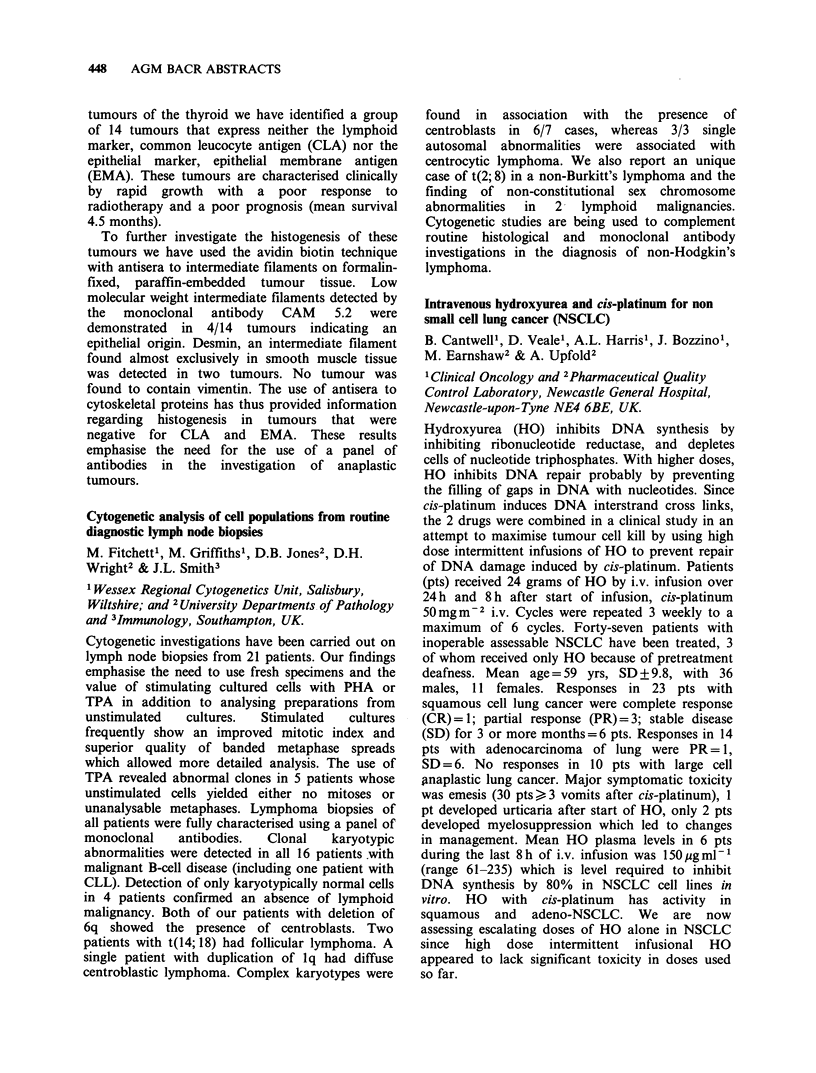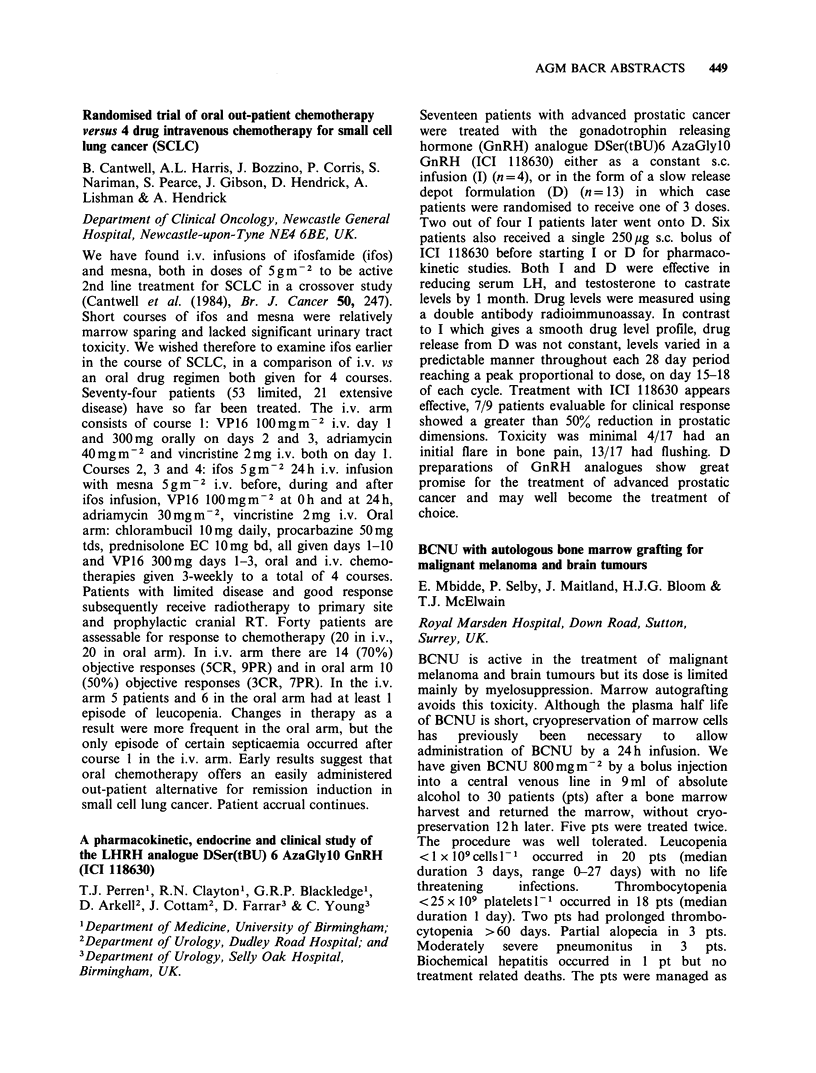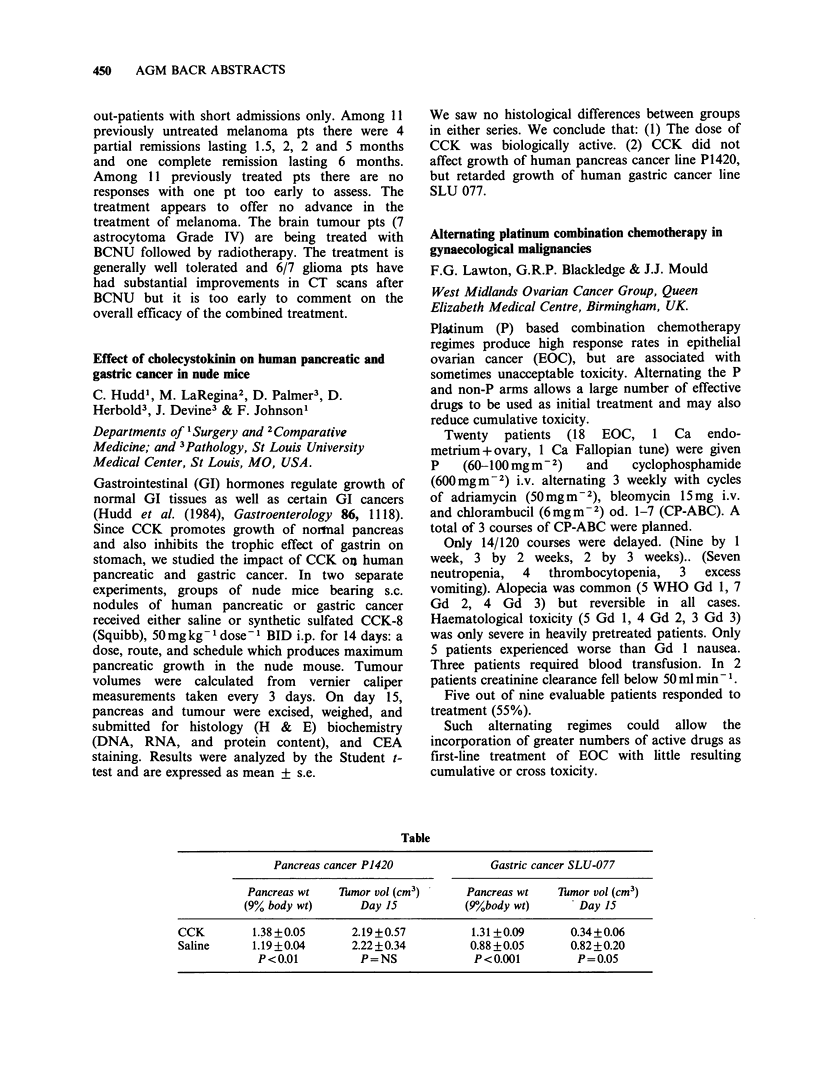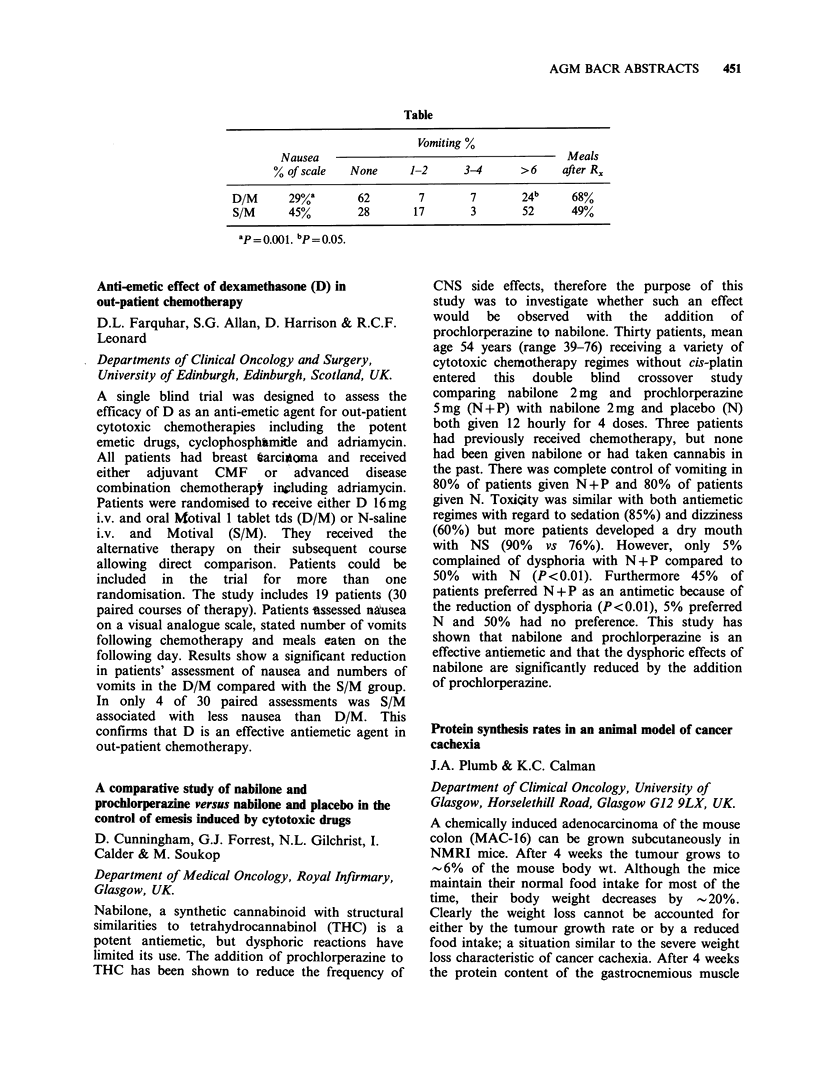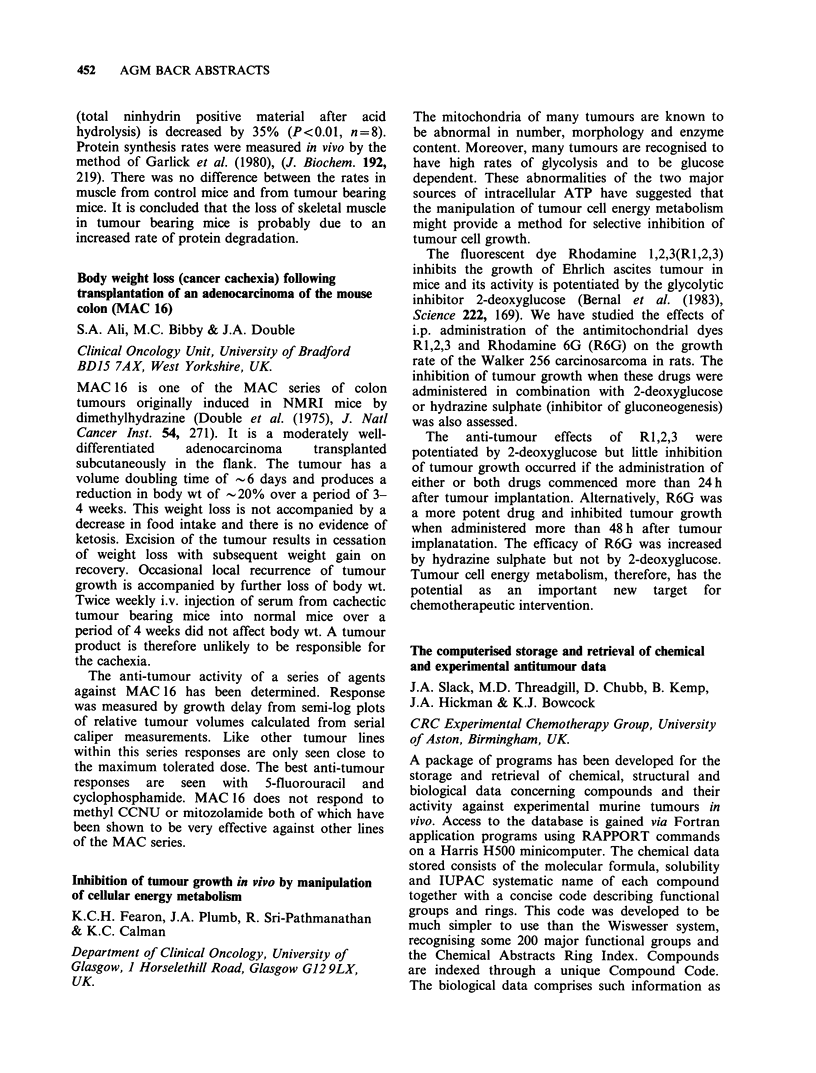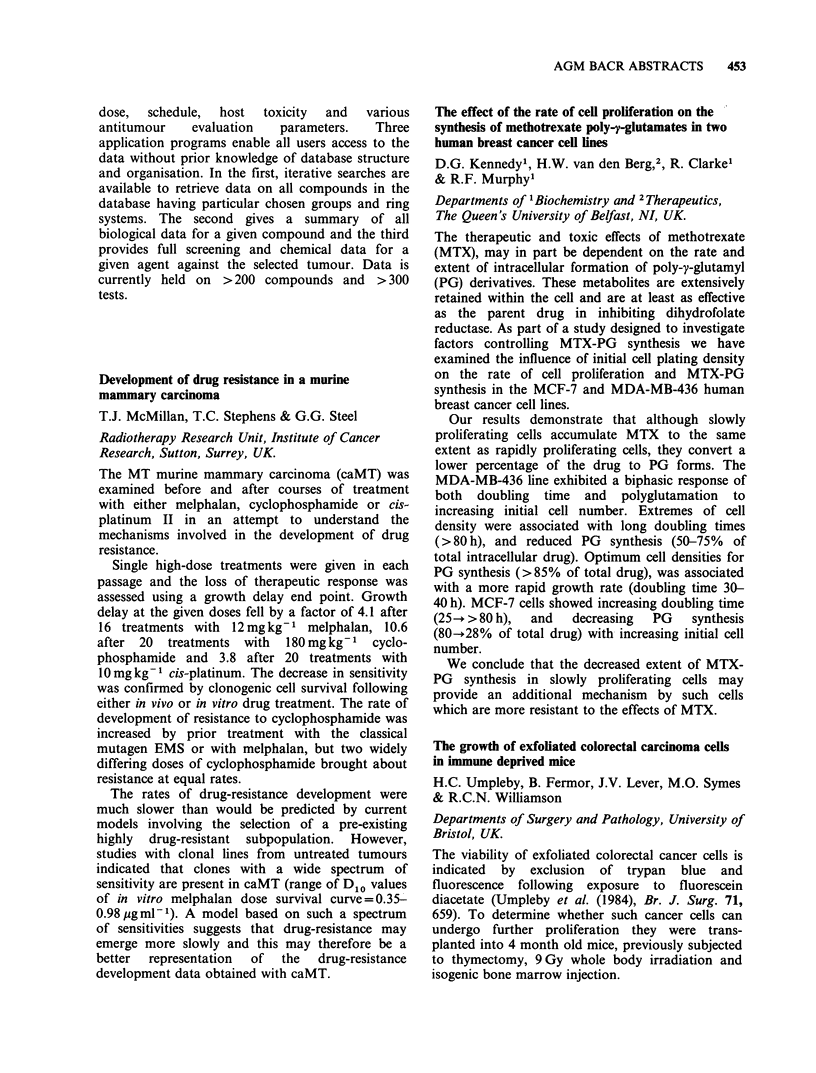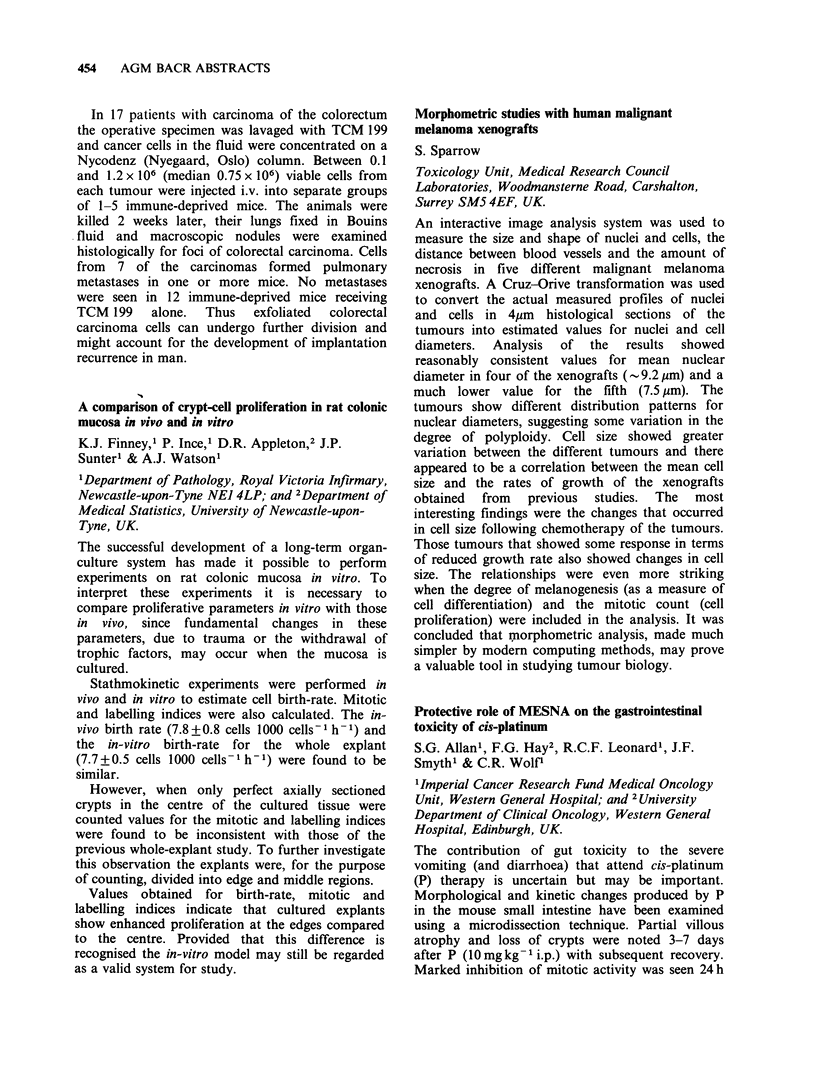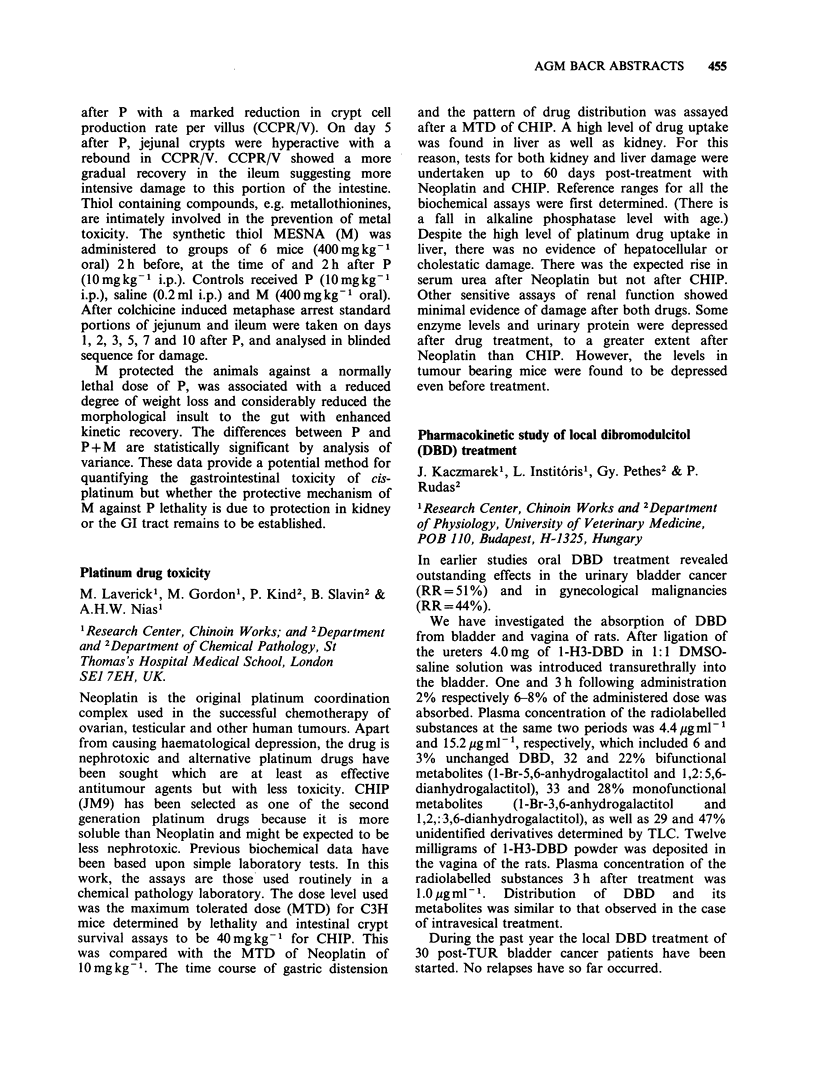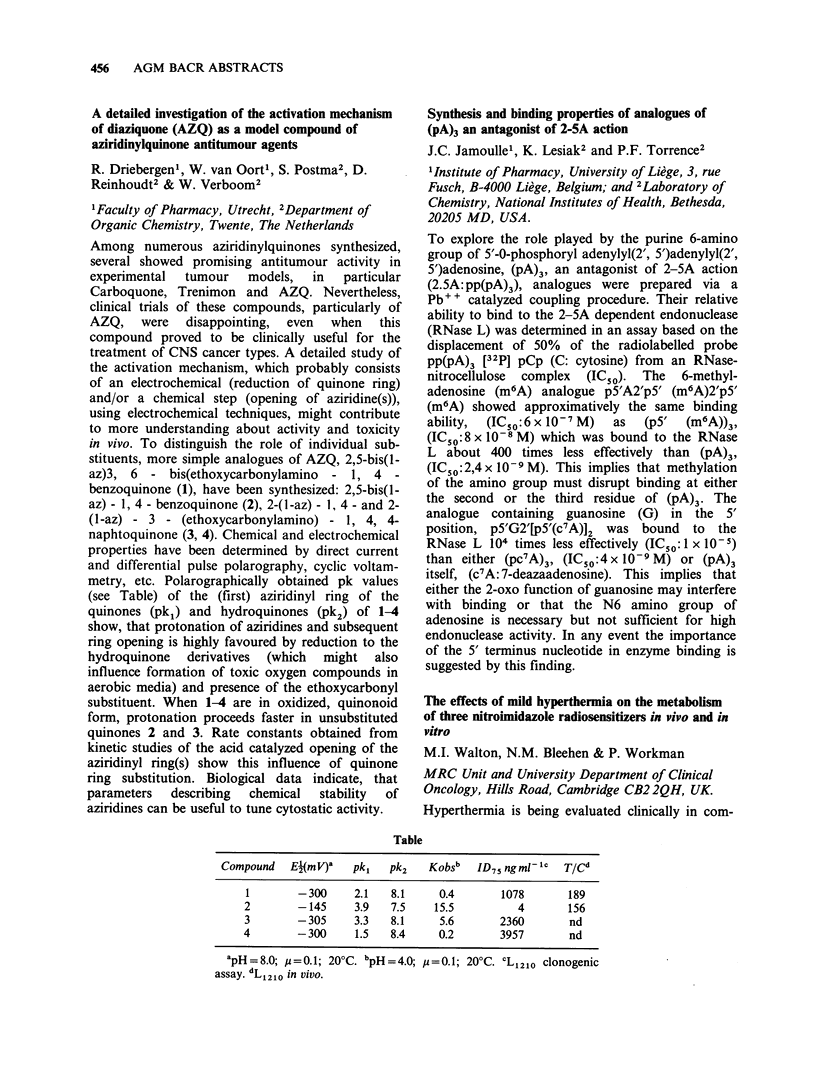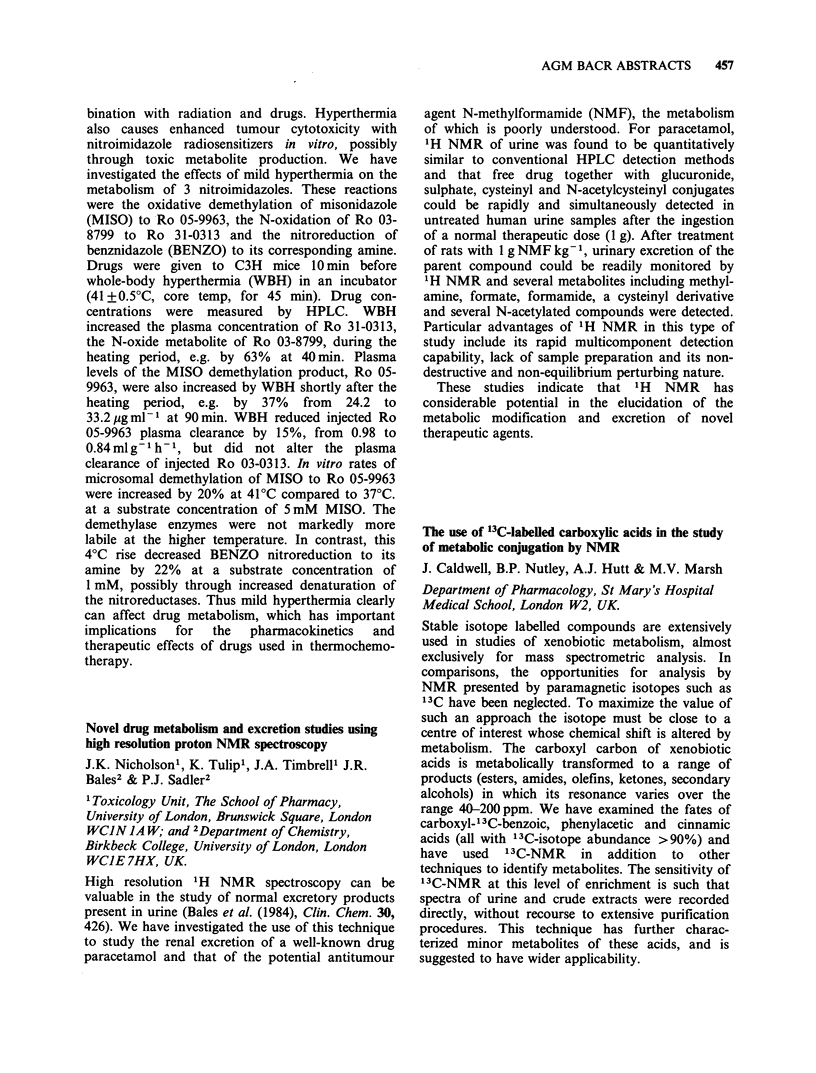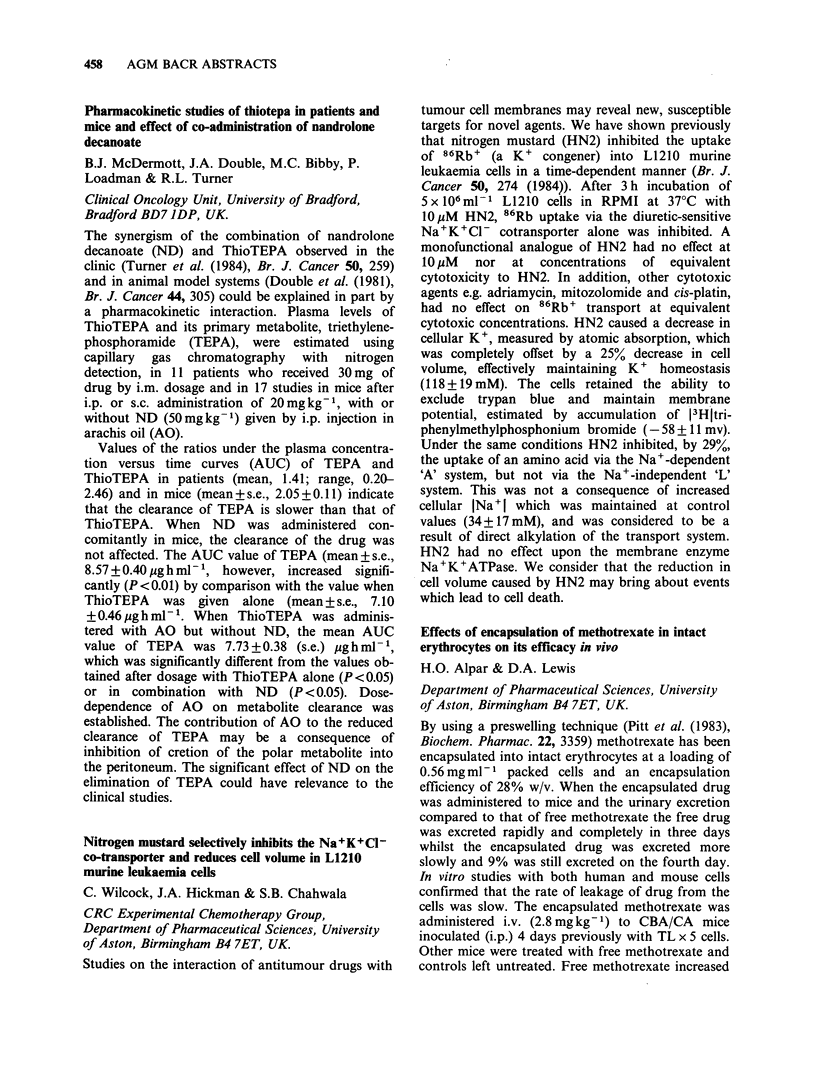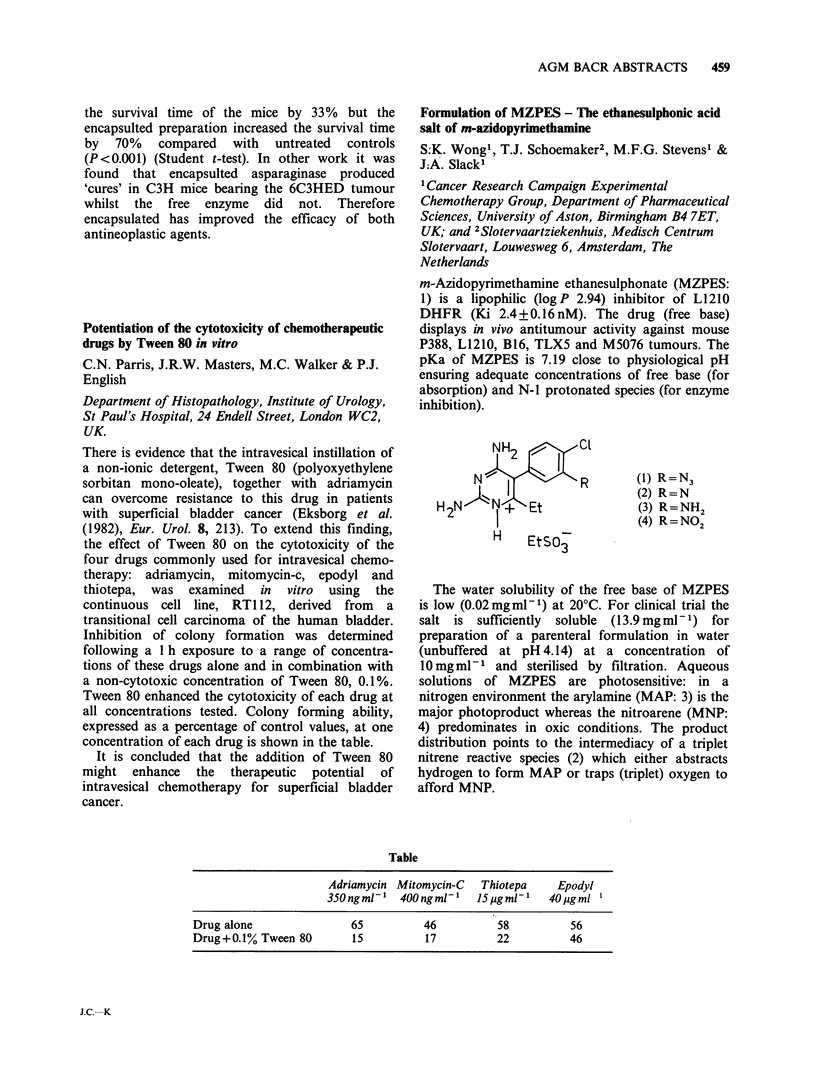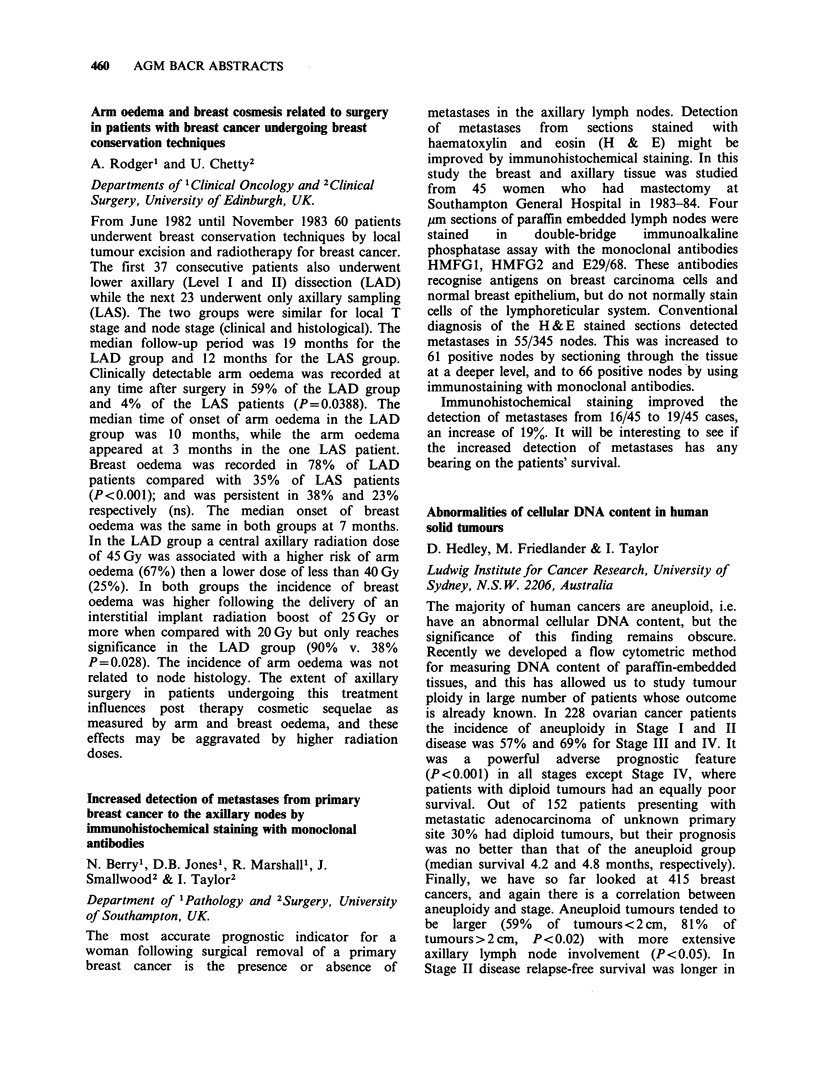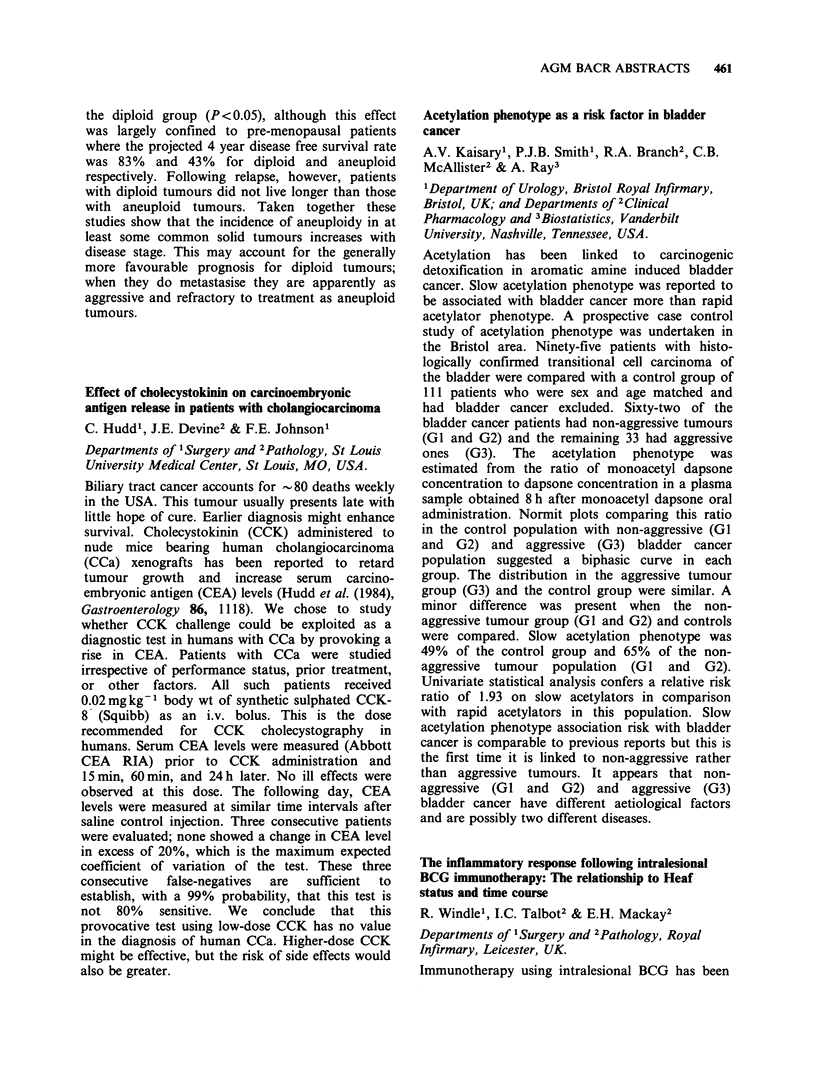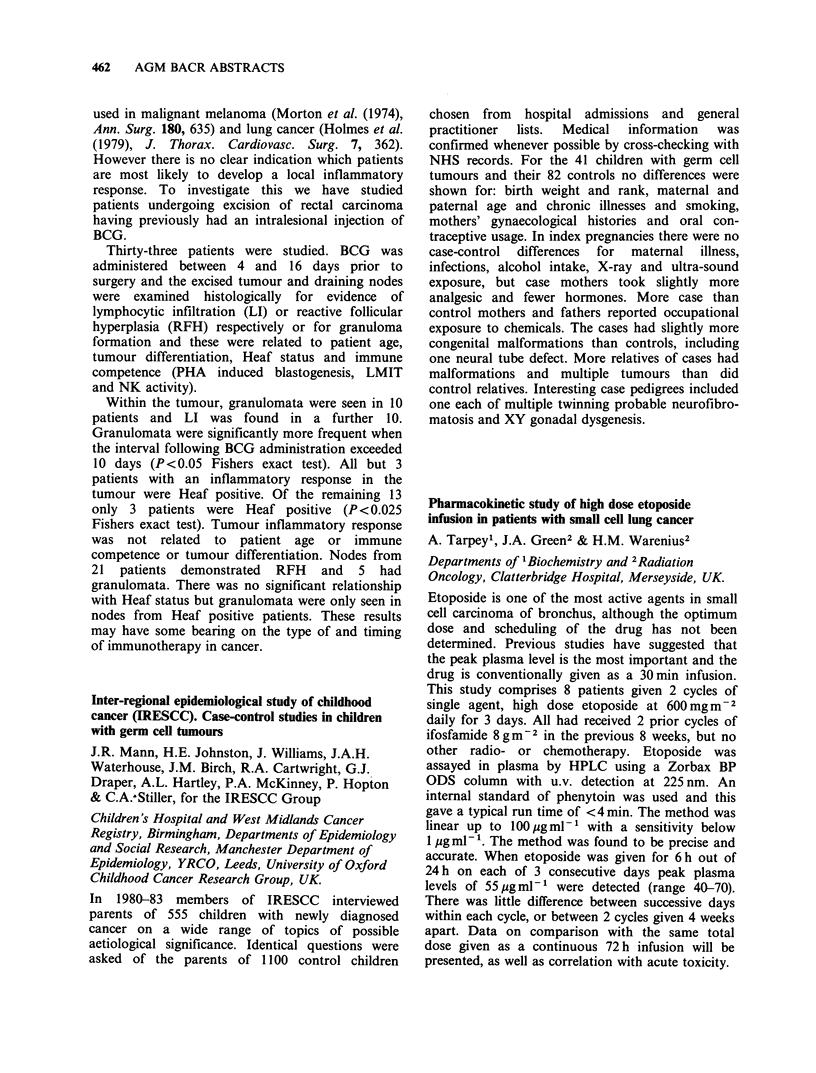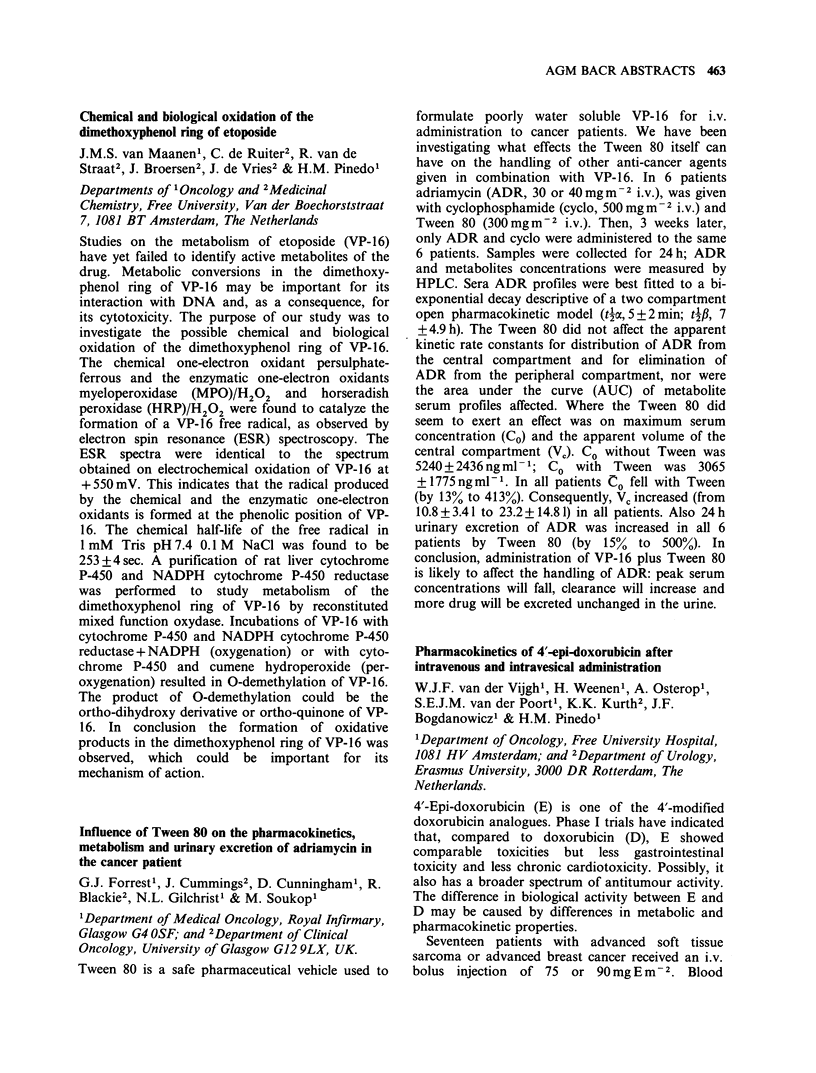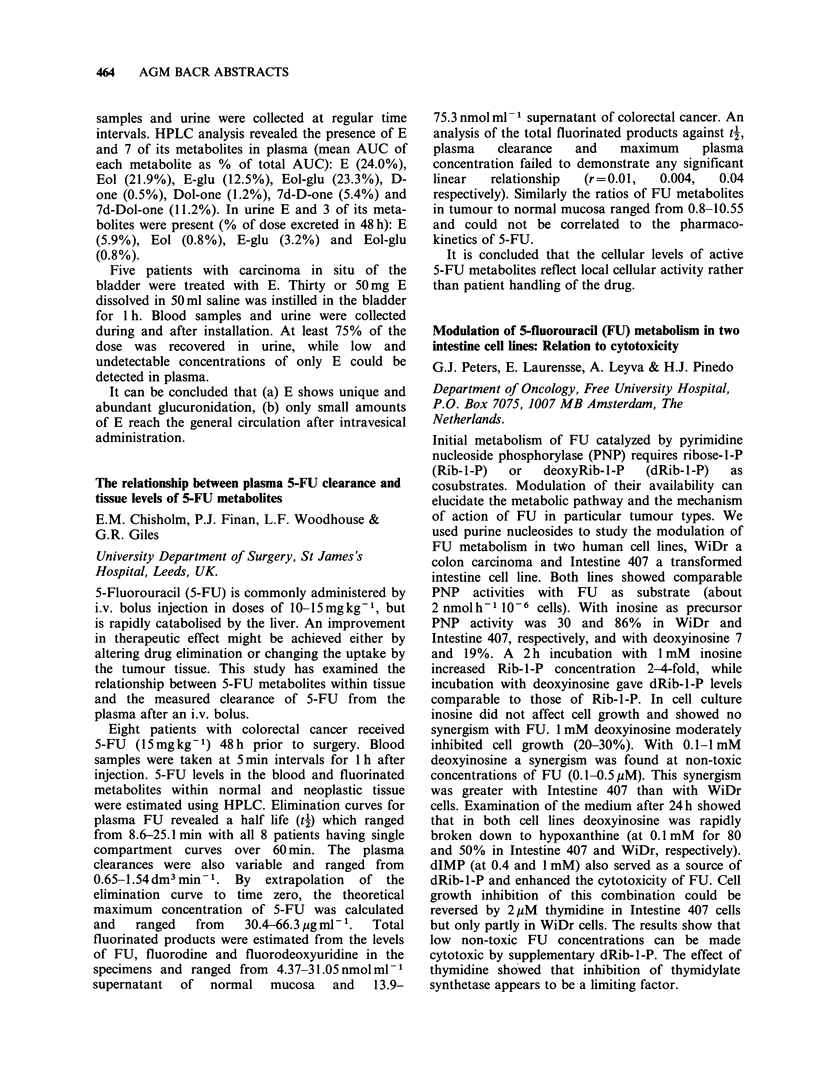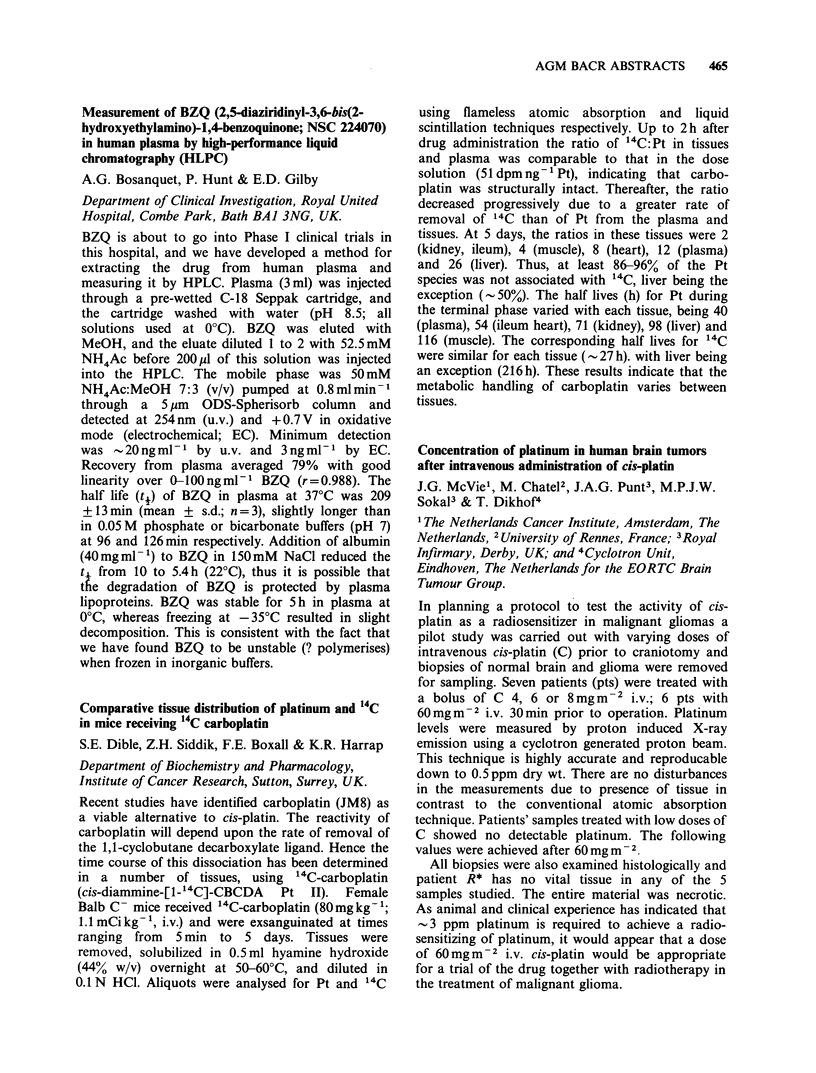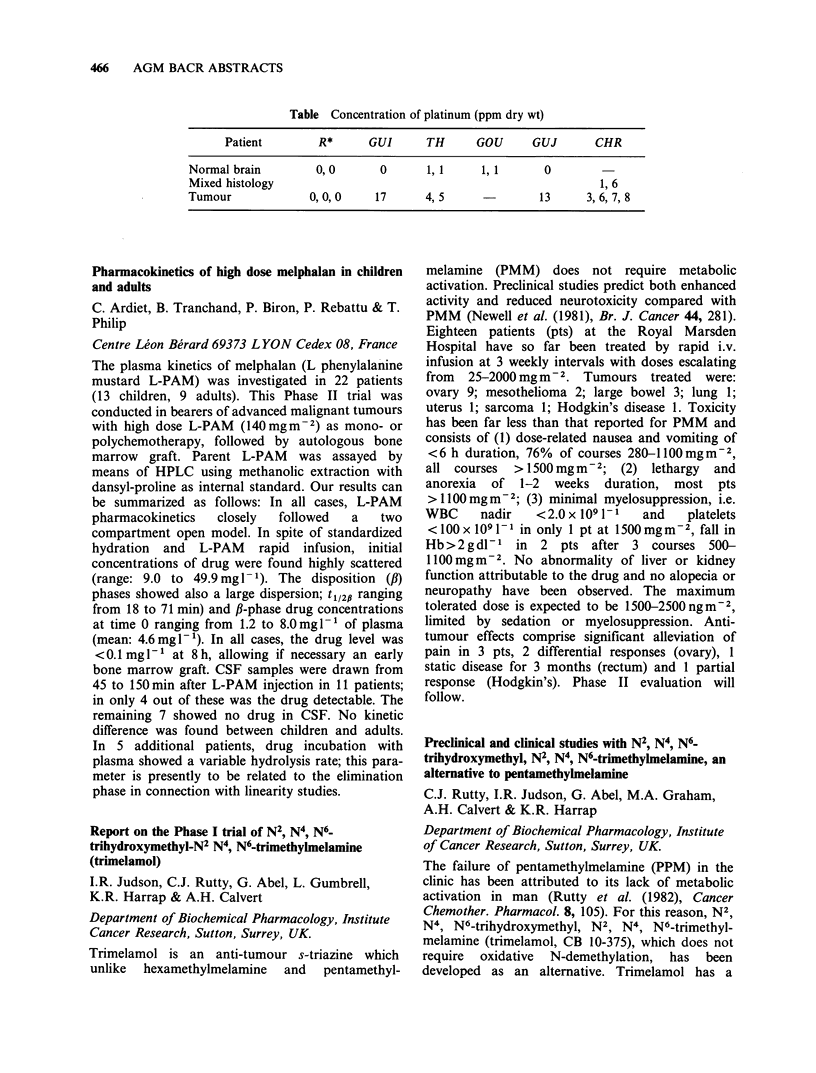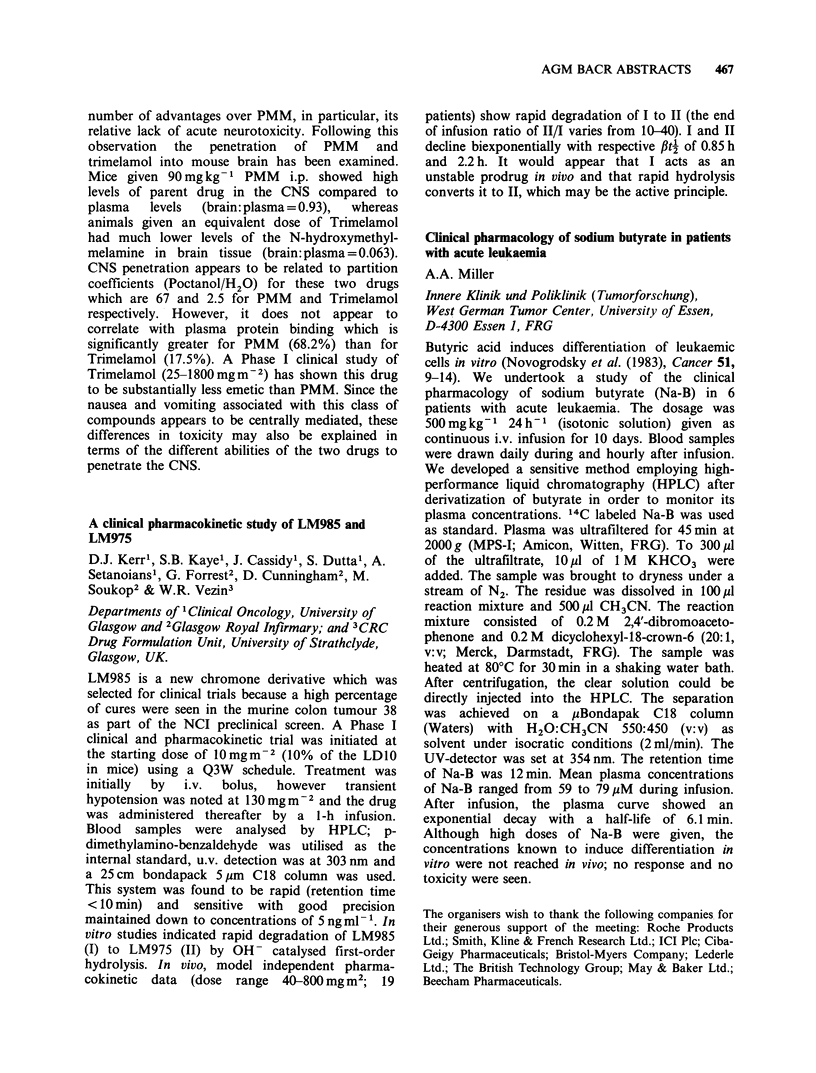# Twenty-sixth annual general meeting of the British Association for Cancer Research (in conjunction with the European Organization for Research and Treatment for Cancer--Pharmacokinetics and Metabolism Group and the Drug Metabolism Group). March 24-27, 1985, Birmingham, U.K.

**DOI:** 10.1038/bjc.1985.210

**Published:** 1985-09

**Authors:** 


*Br. J. Cancer* (1985), **52**, 409–468

### Twenty-Sixth Annual General Meeting of the British Association for Cancer Research* (in conjunction with the European Organization for Research and Treatment for Cancer — Pharmacokinetics and Metabolism Group and the Drug Metabolism Group)

(Incorporating Symposia on 'Reactive intermediates in drug metabolism and carcinogenicity', 'New directions in the therapy of cancer', 'The early clinical trials of novel antitumour agents'; the 1985 Walter Hubert Lecture‡ and the West Midlands Oncology Association Guest Lecture) March 24–27, 1985.

*Held at Aston University, Birmingham, UK.*

### Abstracts of invited papers†

### Symposium:

### Reactive intermediates in drug metabolism and carcinogenicity

### The role of reactive electrophiles in biological activity and toxicity

S.D. Nelson

*Department of Medicinal Chemistry BG-20, University of Washington, Seattle, WA 98195 USA.*

In the last two decades it has become apparent that toxic manifestations of many chemicals arise from covalent interactions of either the compounds themselves or more commonly their metabolites with tissue macromolecules. Identification of the products of covalent interaction has demonstrated that the reactive forms are generally electrophilic. The nature of several classes of electrophiles will be briefly described in regard to mechanisms of formation and reaction with nucleophiles. Ongoing studies in my laboratory on the pathogenesis of toxicities caused by a halogenated alkyl phosphate and a terpene will illustrate procedures that are used to define mechanisms of reactive metabolite formation. Acetaminophen metabolism will be discussed to highlight the complex nature of interactions of some electrophiles with tissue nucleophiles. And, finally, our knowledge of reactive electrophiles will be applied to describe the preparation of suicide substrates of the enzyme, aromatase, as potential therapeutic agents in the treatment of oestrogen-dependent tumours.

### The generation and fate of free radicals in intact cells

S. Orrenius

*Department of Toxicology, Karolinska Institutet, S-104 01 Stockholm, Sweden.*

There is increasing evidence that organic radicals as well as oxygen radicals may cause acute cell injury and also be involved in the initiation and promotion of tumor formation. In the cell, peroxidase-mediated metabolism of various drugs and carcinogens, and one-electron reduction of many quinones, e.g. menadione, can be a source of generation of both types of radicals. Their subsequent interaction with reduced glutathione (GSH) results in the formation of either glutathione S-conjugates or glutathione disulfide (GSSG); the latter may occur in part by dimerization of glutathionyl (GS·) radicals generated during the interaction of organic free radicals with GSH. Since both glutathione S-conjugates and GSSG are actively excreted by the cell, this may result in GSH depletion and, if the production of free radicals continues, their attack on other cellular nucleophilic groups, including those in various proteins and DNA. This may in turn result in either acute or chronic cell damage. Thus, depletion of protein thiols appears to be intimately associated with the development of menadione-induced cytotoxicity in hepatocytes, possibly via a perturbation of their $Ca^{2+}$ homeostasis.

*Enquiries to the BACR Secretariat c/o Institute of Biology, 20 Queensberry Place, London SW7 2DZ.
†Reprints of these abstracts are not available – Ed.
‡This issue pp. 239–302.

### The role of glutathione in the detoxication of electrophilic, peroxy and free radical metabolites

B. Ketterer

*Cancer Research Campaign Molecular Toxicology Research Group, Middlesex Hospital Medical School, London W1P 7PN, UK.*

The oxidation of xenobiotics may give rise to electrophiles, and by-products of oxygen utilization such as phospholipid hydroperoxides both of which are potentially cytotoxic, and may be detoxified by glutathione (GSH) which is a nucleophile and a reducing agent.

GSH reacts poorly with hard electrophiles, but well with soft electrophiles, and a family of GSH transferase enzymes has the capacity to catalyse some if not all these reactions. Such catalysis is an important determinant of the fate of hard electrophiles which tend to be genotoxic. On the other hand if good substrates are present at high doses catalysis itself may cause toxicity due to GSH depletion. The capacity of different tissues to detoxify electrophiles varies: it depends on their GSH content, the qualitative and quantitative distribution of GSH transferase isoenzymes in each tissue, and the nature of the electrophiles themselves.

Reduction of phospholipid hydroperoxides by GSH requires the concerted action of phospholipase $A_2$ and a range of GSH peroxidases (the latter utilize free fatty acid hydroperoxides as substrates).

In principle GSH might act as a trap for oxidising free radicals and also free radicals resulting from one electron oxidations of xenobiotics. This has yet to be demonstrated incontrovertibly because the resultant thiyl free radical is so difficult to detect.

### Observing xenobiotic metabolism by *in vivo* NMR

J.K.M. Sanders

*University Chemical Laboratory, Lensfield Road, Cambridge CB2 1EW, UK.*

Carbon, deuterium and proton NMR spectroscopy have been used to monitor the metabolism of labelled formaldehyde by a variety of bacterial and plant cell preparations. Depending on circumstances, the timescale per spectrum may be as short as 2 min. The rate of metabolism has been correlated with growth and experimental conditions, and the metabolism has been shown to be a detoxifying process in competition with lethal chemistry. The structures of several metabolic products have been determined in these living cultures, and have been shown to include incorporation, oxidation and reduction products. NMR observations using deuterium NMR have enabled us to make some progress towards determining enzyme mechanisms *in vivo*. These results throw much new light on the biochemistry of formaldehyde. More importantly they demonstrate that these techniques are applicable to any xenobiotic which can be labelled and handled at the millimolar level, and to any organism which can be put into an NMR spectrometer.

### Symposium:

### New Directions in the Therapy of Cancer

### Oncogenes in cancer therapy

K. Sikora

*Ludwig Institute for Cancer Research, MRC Centre, Hills Road, Cambridge CB2 2QH, UK.*

The central problem in cancer therapy is the poor selectivity of current systemic agents against the common solid tumours. The demonstration that unique segments of DNA; constant in location and conserved in evolution are involved in growth control opens new avenues for basic and clinical research. The functions of the products of these genes need to be elucidated. Examples of growth control functions include homology to growth factors; surface receptors; protein kinases and cell cycle control proteins. From DNA sequence data peptides predicted to be exposed within the intact molecule can be constructed and used to produce monoclonal antibodies to oncogene products. Such antibodies have now been successfully used to demonstrate the intracellular localisation of gene products as well as the cell cycle regulatory role of the c-*myc* protein *in vitro*. By having a battery of antibodies against the different gene products their direct clinical application for diagnosis and prognosis has become a reality. Immunohistology and flow cytometry permit the geographical and quantitative analysis of function in normal and neoplastic tissues. Furthermore by purification and biochemical analysis the molecular basis for their action can be elucidated. It is likely that by the end of the decade new drugs that inhibit oncoprotein function will be available for clinical trial.

### Clinical correlates of *in vitro* studies of small cell lung cancer

D.N. Carney

*Mater Hospital and Saint Lukes Hospital, Dublin, Eire.*

A panel of 52 cell lines (CL) derived from patients (pts) with small cell lung cancer (SCLC) have been analysed for morphology, cloning efficiency (CE), radiation sensitivity, and the expression of a panel of biomarkers including L-dopa decarboxylase (DDC), bombesin (BN), neuron specific enolase (NSE), and creatine kinase BB (CK-BB). Many CL have been analysed for the c-*myc* and N-*myc* oncogenes. Results of these studies show that SCLC CL can be subdivided into 3 groups: (1) Multipotent CL ($N=2$) which *in vitro* undergo simultaneous differentiation into SCLC, adeno and squamous cell carcinoma providing evidence for a common stem cell for all types of lung cancer: (2) Classic SCLC CL ($N=35$, 70%) grow as tight aggregates of floating cells, have a low CE (1–5%); a long doubling time (DT) (72 h); express elevated levels of all 4 biomarkers, and are radiosensitive. (3) Variant SCLC CL ($N=15$, 30%) grow as loose aggregates; have a high CE (10–30%) a short DT (26 h), are radioresistant, and lack DDC and BN. Amplified levels (20–75 fold) of the c-*myc* oncogene are present in 8/9 variant CL. Studies with the N-*myc* probe revealed significant amplification of the N-*myc* gene in 5 CL not amplified for c-*myc*, and belonging to both the classic and variant subgroups. Tumour tissue harvested directly from 3 pts also showed tumour specific amplification of the N-*myc* gene. The median survival for pts with *myc* amplification was significantly shorter than patients whose cells were not amplified. These data suggest: (1) several distinct classes of SCLC CL can be established from pts with SCLC; (2) Variant CL are associated with a more malignant behaviour *in vitro* and *in vivo*; and (3) the finding of amplification of c-*myc* or N-*myc* in 13 SCLC CL suggests that amplification and/or expression of a *myc*-related sequence may be important for establishing and/or maintaining SCLC.

### Modelling SRC and the EGF receptors by computer graphics

M.J.E. Sternberg & W.R. Taylor

*Laboratory of Molecular Biology, Department of Crystallography, Birkbeck College, London WC1E 7HX, UK.*

A three-dimensional model for the ATP-binding site of the oncogene product v-*src* was proposed by the use of interactive computer graphics. A similar model would apply to sequence related proteins such as other oncogene products, the epidermal growth factor receptor, cAMP-dependent protein kinase and the cell division control protein, CDC28. The model was proposed on the basis of the conservation of certain key amino acid residues (in particular the sequence Gly-X-Gly-X-X-Gly) between the oncogene-product family of proteins and several nucleotide binding proteins of known structure. The interactive computer graphics facilities at Birkbeck were then used to construct a stereochemically-sensible model for the ATP-binding site of v-*src*. The model may prove useful for the design of inhibitors as clinical agents. (Sternberg & Taylor, 1984, *FEBS Lett.*, **175,** 387).

### The dependence of cancer cells on growth factors as a target for chemotherapy

P. Alexander

*CRC Medical Oncology Unit, University of Southampton, Southampton General Hospital, Southampton SO9 4XY, UK.*

Evidence is accumulating that for cancer cells as for normal cells proliferation is dependent on a sequence of interactions between receptors on the outside of the cell membrane and polypeptide growth factors. One aspect of the malignant transformation is that cancer cells, unlike most normal cells, constitutively synthesize both the receptor and the growth factors and this results in self-stimulation. This does not, however, imply that it may not be possible to control tumour growth by interrupting the triggering of receptors, since coupling between the polypeptide growth factors released by the cancer cells and their receptors on the surface of the cell is loose. Data from the growth of cancer cells in serum free medium *in vitro* and from the study of metastases *in vivo* indicate that in general an isolated cancer cell is not capable of autonomous growth as the concentration of growth factors in the environment is too low to be mitogenic, presumably because much of the released growth factor diffuses away. Such isolated cells require factors from the surrounding host tissue if they are to grow. Clusters of cancer cells are capable of truly autonomous growth as the concentrations of growth factors in the immediate vicinity can then attain levels adequate for initiation of mitosis. These findings suggest that it is realistic to attempt to control tumour growth by blockading (or antagonising) autocrine stimulation.

### Symposium:

### The Early Clinical Trials of Novel Antitumour Agents

### Overview of the CRC early Phase I clinical trials project

T.A. Connors

*MRC Toxicology Unit, MRC Laboratories, Woodmansterne Road, Carshalton, Surrey SM5 4EF, UK.*

Basic research in cancer chemotherapy and the 'screening' of chemicals using *in vivo* or *in vitro*

systems produce quite large numbers of drugs of interest but only a few of these are examined further by phased clinical trials. Conventional screening tests relying mainly on rodent transplanted tumours have selected a variety of novel chemical structures which have been disappointing clinically appearing to act similarly to known anticancer agents. The Phase I Clinical Trials Committee of the Cancer Research Campaign aims to increase the numbers of agents entering Phase I clinical trials in the UK and to select for trial chemicals which may not necessarily be active on conventional screening tests but which are of interest for other reasons. The Committee has the resources to fund the synthesis, formulation, preclinical toxicology and Phase I clinical trials of six or more chemicals each year.

**Review of the EORTC drug development program**

H.H. Hansen

*Oncology Department II, Finsen Institute, Copenhagen, Denmark.*

Within the last years the development of new active anti-neoplastic agents has received increasing attention within the EORTC and an organizational structure has been established. Since 1981 the New Drug Development and Coordinating Committee (NDDCC) of the EORTC has functioned with the purpose (1) to transmit information on the availability of new agents in Europe to the various EORTC groups, but especially to the Screening and Pharmacology group, Clinical Screening group, Early Clinical Trials group (ECTG) and the Pharmacology and Metabolism group (PAM group), (2) to integrate work conducted within the latter four groups, (3) propose testing of new drugs, (4) propose and initiate synthesis of new drugs. Standardized guidelines for (a) preclinical toxicology, (b) formulation of investigational cytotoxic drugs, (c) Phase I clinical trials with anticancer drugs have been established. Increasing cooperation with exchange of information with other organizations focusing on new anti-cancer drug development is in progress, especially with New Drug Development Program, NCI, Bethesda, USA and the Cancer Research Campaign Phase I Committee, UK. In 1983 the organization was expanded with the creation of the EORTC Office for New Drug Development (EODD) located in Amsterdam. At present 16 new cytostatic agents are undergoing preclinical evaluation in the EORTC program as exemplified by the new compound α-/β-triglycidyl-urazol (TGU), (NSC 332488).

**Clinical aspects of Phase I trials**

A.H. Calvert

*Department of Biochemical Pharmacology, Institute of Cancer Research, Sutton, Surrey SM2 5PX, UK.*

The conduct of a Phase I trial presents conflicting demands on the clinician. The purely scientific aspect demands documentation of the maximum tolerated dose of the drug, its pharmacology and its side effects, while ethical considerations demand that patients should be treated in as safe a way as possible. The usual practice has been only to admit patients untreatable by other means to Phase I studies, to obtain their consent having informed them that the treatment will probably be of no benefit and to start the trial at a dose ~10 times lower than that anticipated on the basis of preclinical observations. These precautions have, in general, led to the introduction of new drugs with minimal hazard to the patients at the starting dose level, but determination of the maximum tolerated dose necessarily places patients at some risk at the higher end of the dosage spectrum. Further, patients treated at lower doses have little chance of any therapeutic benefit. A recent survey showed that the overall response rate in Phase I trials was very low (~2%). We have attempted to minimise the risk to the patient and maximise the possibility of a therapeutic response by the following means: (i) All the available preclinical data is assessed and used where possible to select patients with the maximum chance of responding; (ii) At non-toxic levels, doses are escalated on successive courses given to the same patient; (iii) A running analysis of all toxicity data for a dose response effect is kept in an attempt to anticipate side effects which may have been trivial at a lower dose from becoming severe on dose escalation. The advantages and disadvantages of this approach are examined in the context of three recently performed Phase I studies on Carboplatin, CB3717 and Trimelamol.

**The role of preclinical and clinical pharmacokinetics in Phase I trials**

P. Workman

*MRC Clinical Oncology Unit, MRC Centre, Hills Road, Cambridge CB2 2QH, UK.*

The aim will be to illustrate the importance of pharmacokinetic studies in the rational selection of novel antitumour agents and their subsequent evaluation in the clinic. A case will be made for detailed pharmacokinetic studies to be carried out in the same experimental animals used for antitumour and toxicity testing. This allows antitumour activity and toxicity to be related to exposure parameters for parent drug or active metabolites, e.g., peak concentration, area-under-the-curve, etc. A prediction can then be made of the minimum plasma and tumour exposures likely to be required for antitumour activity in man. Phase I pharmacokinetics will reveal whether these exposures can be realized, and also identify differences in drug handling as compared to the screening species, e.g. extent of metabolic activation. Similar procedures may also be used to predict exposures likely to elicit drug toxicity. Where a series of congeners are under development pharmacokinetic behaviour can be a major factor in the selection procedure. The choice of a new analogue to replace an existing agent may be based largely on pharmacokinetics. The following examples are from current Phase I studies: (1) impaired metabolic activation of a prodrug (e.g. pentamethylmelamine) in man may lead to the use of preactivated forms (e.g. trimelamol); (2) a drug which gives inadequate peak levels after oral administration (e.g. CCNU) may be replaced by an intravenously administered agent giving a higher peak (e.g. mitozolamide); (3) a neurotoxic radiosensitizer (misonidazole) may give way to a hydrophilic analogue which is excluded from nervous tissue and cleared rapidly by the kidney (SR 2508) or a basic analogue which exhibits both efficient renal and metabolic clearance (Ro 03-8799). Phase I clinical pharmacokinetics also contribute to rational optimization of drug dose, schedule and route of administration, as well as identifying situations in which dosage alterations are required because of renal or hepatic dysfunction.

### West Midlands Oncology Association Guest Lecture

### Novel clinical strategies for chemotherapy

E. Frei III, *Dana-Faber Cancer Institute, Boston, Mass. 02115, USA.* (By title only)

### Abstracts of members' proferred papers

### 12-0-tetradecanoylphorbol-13-acetate (TPA)-stimulated mono-morphonuclear leukocytes (MMNs) cause oxidation of thiols and toxicity in A549 lung carcinoma cells

A. Gescher[1], A.E. Brodie[2] & D.J. Reed[2]

[1]*Department of Biochemistry and Biophysics, Oregon State University, Corvallis, Oregon, USA; and* [2]*CRC Experimental Chemotherapy Group, University of Aston, Birmingham B4 7ET, UK.*

Phorbol ester tumour promoters, such as TPA, are activators of the cytotoxic potential of human poly-morphonuclear leukocytes (PMNs). The mechanism by which TPA-stimulated PMNs induce toxicity in target cells is considered to involve oxygen metabolites, such as superoxide, hydrogen peroxide and the hydroxyl radical. Effects of TPA-stimulated MMNs on target cells are much less well understood. An attempt was made to clarify some of the biochemical interactions between TPA and MMNs. TPA-stimulated MMNs were found to be toxic towards A549 human lung carcinoma cells which had been permanently desensitized against the direct growth-inhibitory effect of TPA. Levels of the intracellular protectant glutathione were reduced by 37% in MMNs exposed to TPA for 24 h; glutathione levels in the target cells were not affected by TPA-stimulated MMNs. The supernatant of incubations of MMNs with TPA contained a species which oxidised 5-thio-2-nitro-benzoic acid (TNB). The generation of this species appeared to be dependent on the myeloperoxidase-$H_2O_2$-halide system, as its formation was abolished by catalase, azide and cyanide, but not by superoxide dismutase (SOD). However, catalase, azide and cyanide did not inhibit toxicity exerted by TPA-stimulated MMNs, whereas SOD did. Therefore the TNB oxidant does not appear to be involved on the process which led to cytotoxicity caused by TPA-stimulated MMNs in A549 cells.

### An improved assay for 17α hydroxylase/C17-C20 lyase: A target enzyme for the treatment of hormone dependent prostatic cancer

S.E. Barrie

*Department of Biochemistry and Pharmacology, Institute of Cancer Research, Sutton, Surrey, UK.*

Inhibition of androgen biosynthesis and thus a reduction in the level of circulating hormone is beneficial for the treatment of androgen dependent prostatic cancer. To achieve this the most suitable enzyme target is the microsomal 17α hydroxylase/C17–C20 lyase which catalyses the 17α hydroxylation of progesterone or pregnenolone, and then the cleavage of the C17–C20 bond to give androstenedione or dehydroepiandrosterone respectively. Previous assays involved long extraction and crystallisation procedures or lacked the ability to monitor any side reactions. This assay has neither drawback. The enzyme source (e.g. microsomal extract from rat testis) is incubated with 3H-progesterone (1 $\mu$M, 1 mCi $\mu mol^{-1}$), NADPH and other cofactors in a total volume of 100 $\mu$l. The reaction is stopped by the addition of 200 $\mu$l acetonitrile:methanol (1:2) containing 100 $\mu$M unlabelled progesterone, 20α hydroxyprogesterone, 17α hydroxyprogesterone, androstenedione, and testosterone. The samples are stored at −20°C until analysed by HPLC. 200 $\mu$l is then injected onto a 30 cm Apex C18 column running with acetonitrile:methanol:water (22:40:38) at 1.5 ml $min^{-1}$ at 20°C. Under these conditions androstenedione and testosterone run as single peak followed by 17α hydroxyprogesterone, 20α hydroxyprogesterone and progesterone. The peaks corresponding to the unlabelled steroids added to the sample are collected into mini vials using an LKB fraction collector fitted with a signal sensor attached to the 254 nm output of the HPLC detector, and counted. Each run takes 30 min. This system allows easy determination of the 17α hydroxylase and C17–C20 lyase activities, 20α hydroxylation and substrate availability.

### Inhibition of CSCC and aromatase enzymes by analogues of aminoglutethimide

M.J. Daly[1], P.J. Nicholls[1], H.J. Smith[1] & M.G. Rowlands[2]

[1] *Welsh School of Pharmacy, PO Box 13, Cardiff, CF1 3XF; and* [2] *Institute of Cancer Research, Sutton, Surrey SM2 5PX, UK.*

Selective inhibition of steroidogenesis has many applications in cancer and other research. Aminoglutethimide, (*1*, 3-(4-aminophenyl)-3-ethylpiperidine-2,6-dione), is a treatment for mammary carcinoma in post-menopausal and oophorectomised women which acts by inhibiting the production of steroids via inhibition of the cholesterol side chain cleavage (CSCC) and aromatase enzyme complexes. Several analogues closely resembling *1* have been synthesised and tested in established *in vitro* assays (Graves & Salhanik, 1979, *Endocrinology*, **105**, 52; Hochberg *et al.*, 1974, *Biochemistry* **13**, 603) for their inhibitory activity against these enzyme systems. 3-(4-aminophenyl)-3-ethyl-pyrrolidine-2,5-dione (*2*), and *1* were found to be potent inhibitors of aromatase, while 5-(3-aminophenyl)-5-ethylimidazolidine-2,4-dione (*3*) was a weak inhibitor and 5-(3-(aminophenyl)-5-ethylpyrimidine-2,4,6-trione (*4*) was totally non-inhibitory. As expected, *1* was a potent inhibitor of the CSCC enzyme, while *2* possessed only weak activity, and *3* and *4* were non-inhibitors. Using such studies, it may prove possible to design selective inhibitors of steroidogenesis for therapeutic use in hormone dependent cancers.

### Metabolic switching and improved antitumour selectivity with β, β-difluorochlorambucil

F.Y.F. Lee[1], P. Coe[2] & P. Workman[1]

[1] *MRC Clinical Oncology Unit, Cambridge; and* [2] *Department of Chemistry, University of Birmingham, UK.*

β,β-Difluorochlorambucil (β-$F_2$CHL) was synthesized in the hope of blocking the mitochondrial β-oxidation of chlorambucil (CHL), which proceeds via 3,4-dehydrochlorambucil (DeHCL) to yield phenyl acetic mustard (PAAM), a putatively adverse metabolic pathway. In mice, LD50 doses were 15.9, 30.0 and 60.2 mg $kg^{-1}$ for PAAM, CHL and β-$F_2$CHL respectively, while doses to give a 15 day growth delay (ED15) in the KHT tumour were 8.0, 14.6 and 20.0 mg $kg^{-1}$. Thus, compared to CHL, PAAM was more potent while β-$F_2$CHL was less potent in both cases. However, therapeutic indices, calculated as LD50/ED15, were 2.0, 2.1 and 3.0 for PAAM, CHL and β-$F_2$CHL respectively, showing some advantage for the new analogue. Pharmacokinetic studies were carried out using HPLC analysis. Both DeHCL and PAAM were detected as metabolites of β-$F_2$CHL, but in much reduced quantities compared to those produced from CHL, thereby demonstrating that β-oxidation was not blocked completely but was significantly impaired. Consequently the area-under-the-curve of concentration against time was 2.7 times greater for parent β-$F_2$CHL compared to parent CHL, whereas the AUC for PAAM and DeHCHL were reduced by 2-fold and 15-fold respectively. The AUC for total plasma bifunctional nitrogen mustards was unchanged, but that for free mustards in plasma water was decreased 1.6-fold. Metabolic switching was demonstrated by the appearance of two new, unidentified metabolites with β-$F_2$CHL, and evidence suggests these may be dechloroethylation products. The reduced potency of β-$F_2$CHL compared to CHL is attributed to the lower levels of free plasma mustards, particularly PAAM, but the reason for the modest improvement in therapeutic index is not known.

### Metabolic activation of 1-naphthol, a potential antitumour agent, in model *in vitro* systems

M. d'Arcy Doherty & G.M. Cohen

*Toxicology Unit, The School of Pharmacy, London University, 29/39 Brunswick Square, London WC1N 1AX, UK.*

1-Naphthol is selectively toxic to short-term organ cultures of human colonic tumour tissue compared to normal intestinal mucosa from the same patients (Cohen *et al.* (1983), *Biochem. Pharmac.* **32**, 2363). We have previously shown that 1-naphthol is metabolised by rat liver microsomes or a fully reconstituted cytochrome P-450 system to 1,4-naphthoquinone and covalent binding species (Doherty & Cohen (1984), *Biochem. Pharmac.*, **33**, 3201; and unpublished data).

In cultured human colonic tumour cells, [1-$^{14}$C]-1-naphthol is activated to covalently bound species. In order to investigate the possible metabolic activation of 1-naphthol by human colonic tumour cells, we studied the metabolism of 1-naphthol in microsomal preparations from these cells. Under the conditions used, no significant metabolism of 1-naphthol to soluble naphthoquinone metabolites could be detected. Alternative pathways of metabolic activation have been suggested by our recent studies showing that other *in vitro* systems, horseradish peroxidase and tyrosinase readily activate 1-naphthol to reactive products. The latter observation is particularly interesting as it raises the possibility of the selective treatment of melanotic melanomas with 1-naphthol.

### Can the anti-tumour effect of adriamycin be mediated by a semi-quinone free radical?

N. Willmott[1], J. Cummings[2] & A.T. Florence[1]

[1]*Department of Pharmacy, University of Strathclyde; and* [2]*Department of Clinical Oncology, University of Glasgow, Glasgow, Scotland, UK.*

Adriamycin (Adx)-loaded albumin microspheres were prepared by stabilization of water in oil emulsion droplets containing albumin and drug. Average particle size in these studies was between 15 and 24 μm, and they contained on average 10 (range 6–14) μg Adx $mg^{-1}$ of microspheres. To assess the anti-tumour effect of drug-loaded microspheres they were injected directly into s.c. growths of a non-immunogenic rat mammary carcinoma Sp107 and subsequent tumour growth followed. It was consistently found that Adx (24–87 μg) in microspherical form exhibited a superior anti-tumour effect to an equal or even greater amount of drug in solution. To investigate the levels of unchanged drug and metabolites in tumour tissue in this model rats were sacrificed at intervals following intratumoral injection of either drug in solution (80 μg) or in microspherical form (60 μg) and tumours analysed using an HPLC method that can discriminate between Adx and fluorescent metabolites (Cummings *et al.* (1984), *J. Chromatography*, **311**, 125). Two rats were used per time point and results were analysed using a two-way analysis of variance with replication. It was observed that Adx in microspherical form promoted a significantly higher level of the metabolite adriamycinol 7-deoxyaglycone in tumour tissue compared to drug in solution (peak level 3.9 μg at 48 h after injection compared to 0.6 μg at 16 h). Since it has been shown *in vitro* that 7-deoxyaglycone metabolites of Adx are a by-product of semiquinone free radical formation (Gutierrez *et al.* (1983), *Arch Biochem. Biophys.* **223**, 68), these results are consistent with the enhanced anti-tumour effect of Adx in microspherical form being due to generation of a semiquinone free radical intermediate.

### Enzymic pathways in the *in vitro* metabolism of mitozantrone

C.R. Wolf, J.S. Macpherson & J.F. Smyth

*Imperial Cancer Research Fund Medical Oncology Unit, Western General Hospital, Crewe Road, Edinburgh, EH4 2XU, UK.*

Mitozantrone is an anthraquinone derivative with potential use in the treatment of breast cancer. In conjunction with our pharmacokinetic studies we have investigated the *in vitro* metabolism of this compound in rat liver microsomal preparations to determine the probable pathways of metabolism in man. On incubation of liver microsomal samples with mitozantrone and UDPGA a metabolite was detected by HPLC which was sensitive to treatment with β-glucuronidase. The formation of this product was also inhibited by inhibitors of UDPGA-glucuronyl transferase. In a second incubation system containing microsomes, NADPH and glutathione, two metabolites were formed. The production of these metabolites were dependent on the presence of both glutathione and NADPH. This suggests that initial oxidation by cytochrome P-450 is followed by a conjugation reaction with glutathione. The formation of these products was sensitive to inhibitors of both cytochrome P-450 and the glutathione transferases (GST), the inhibitors being chlorodinitrobenzene and hexachlorobutadiene. Hexachlorobutadiene is a particularly good substrate for the microsomal GST. This fact together with the finding that the addition of cytosol to the incubation systems did not increase metabolite formation suggests that the as yet poorly characterised microsomal GST play an important role in the disposition of mitozantrone. The above data provide evidence that mitozantrone is metabolized to both glucuronide and glutathione conjugates. Whether there is any further processing of these metabolites *in vivo* remains to be established.

**Lack of mitoxantrone free radicals and redox cycling in rabbit heart sarcoplasmic reticulum; comparison with doxorubicin**

L.H. Patterson & J. Basra

*School of Pharmacy, Leicester Polytechnic, Leicester LE1 9BH, UK.*

Mitoxantrone is a bisalkylaminoanthraquinone antitumour agent broadly based on the anthracyclines such as doxorubicin. In this study we have investigated free radical formation and redox cycling by mitoxantrone in heart tissue since these events are associated with doxorubicin cardiotoxicity. Electron spin resonance (esr) spectrometry was used to directly monitor free radical formation in NADPH fortified rabbit heart sarcoplasmic reticulum (SR) incubated under anaerobic conditions with drugs essentially as previously described (Oldcorne *et al.* (1984), *Biochem. Soc. Trans.* **12,** 681). NADPH oxidation and superoxide formation measured as described by Kharasch & Novak (1983) (*Arch. Biochem. Biophys.,* **224,** 682) were used as indicators of SR mediated drug redox cycling. Mitoxantrone free radicals were not observed in mitoxantrone (400 $\mu$m)-SR incubations up to 1 h although under identical conditions doxorubicin free radicals were readily obtained. Furthermore, mitoxantrone diminished doxorubicin free radical esr signal intensity when the two drugs were coincubated in heart tissue. Doxorubicin free radical esr signal intensity was dependent on SR protein concentration and could be partially inhibited by ascorbic acid (500 $\mu$M). Mitoxantrone (50 $\mu$M) inhibited SR basal rate NADPH oxidation ($2.9 \pm 0.3$) and superoxide formation ($2.0 \pm 0.3$), both measured as nmol.mg protein$^{-1}$ min$^{-1}$, by 32% and 39% respectively. Doxorubicin (50 $\mu$M) however stimulated these events by 27% and 53% respectively. These results show that mitoxantrone does not redox cycle in heart SR to produce reactive oxygen species. It is unlikely, therefore, that the cardiotoxic potential of mitoxantrone is mediated by the same mechanism suggested for doxorubicin.

**Iminium ion formation *vs* carbinolamine-aminoaldehyde tautomerism in the solvolysis of triazine and triazene carbinolamines: A model system for the metabolism of N-alkyl xenobiotics**

K. Vaughan, R.J. LaFrance, H.W. Manning & C.M. Hemens

*Department of Chemistry, Saint Mary's University, Halifax, Nova Scotia B3H 3C3, Canada.*

The oxidative metabolism of N-alkylamines to N-(1-hydroxyalkyl)amines, 'carbinolamines' is a ubiquitous pathway for many classes of xenobiotic molecules bearing an N-alkyl group. It has been suggested that ionization of the intermediary carbinolamine gives rise to electrophilic iminium ions, which might form covalent bonds with nucleophilic functionalities present in biomacromolecules (Murphy (1973), *J. Biol. Chem.,* **248,** 2796). To test this hypothesis, we have studied the behaviour of a number of stable carbinolamines of the triazine and triazene series under chemical conditions where iminium ion formation should be favoured. There is no evidence for iminium ion formation from the acyclic carbinolamines, whereas when the hydroxyl group is derivatised as an acetate functionality, iminium ion formation can be clearly demonstrated by classical chemical kinetics. In contrast, cyclic carbinolamines undergo facile attack by a nucleophilic solvent *without* the requirement for derivatisation; the reactivity of the cyclic carbinolamine can be explained by the phenomenon of carbinolamine$\rightleftharpoons$aminoaldehyde ring-chain tautomerism, which is an unavailable option for the acyclic carbinolamine. The conclusion of this study is that the formation of iminium ions during metabolism of some N-alkyl xenobiotics must involve biological conjugation of the $\alpha$-hydroxyalkyl function, whereas, in those cases where a cyclic carbinolamine is the intermediary metabolite, the suggested reactivity with bionucleophiles may involve pathways other than the formation of iminium ions.

**The metabolism of N,N-dimethylformamide (DMF) and N-methylformamide (NMF) in mice**

P. Kestell & A. Gescher

*CRC Experimental Chemotherapy Research Group, University of Aston, Birmingham, UK.*

The formamides NMF and DMF possess antitumour activity in mice (Gescher *et al.,* (1982), *Br. J. Cancer,* **45,** 843; Dexter et al. (1982), *Cancer Res.* **42,** 5018) and induce terminal differentiation in HL-60 human promyelocytic leukaemia cells. (Collins *et al.,* (1978), *Proc. Natl Acad. Sci* (*USA*) **75,** 2458). Whether their antitumour activity *in vivo*

and ability to induce differentiation *in vitro* are related is not known. In order to elucidate the mechanism of antitumour activity of NMF and DMF we studied their metabolism in male CBA/CA mice. [$^{14}$C]Methyl labelled drugs were injected intraperitoneally (6.8 mmol kg$^{-1}$). The drugs were partially metabolized to [$^{14}$C]$CO_2$. Within 72 h after administration of [$^{14}$C]NMF 14.1% of the dose was exhaled as [$^{14}$C]$CO_2$, and after [$^{14}$C]DMF 23.1%. Of the radioactivity injected with [$^{14}$C]NMF 73.3% was excreted in the urine. In case of [$^{14}$C]DMF 62.4% of the dose was recovered in the urine. Examination of urine samples collected after injection of [$^{14}$C]NMF by TLC-autoradiography revealed the presence of metabolites, one of which was identified as methylamine after reaction with 2,4-dinitrobenzenesulphonate. The product, N-methyl-2,4-dinitroaniline, was isolated and characterized by mass spectrometry. DMF does not appear to undergo an analogous metabolic route, as similar treatment of urine samples collected from mice dosed with [$^{14}$C]DMF did not detect the presence of dimethylamine.

### Differential susceptibility to N-methylformamide induced hepatotoxicity in rats and mice

K. Tulip, J.K. Nicholson & J.A. Timbrell

*Toxicology Unit, The School of Pharmacy, University of London, 29/39 Brunswick Square, London WC1N 1AX, UK.*

N-methylformamide (NMF) is a potential antitumour drug which has been associated with liver damage in mice, rats and humans. The mechanism of this hepatoxicity is unknown. We have investigated the hepatotoxicity of NMF in male Sprague Dawley rats. In this strain NMF in single doses of 200–800 mg kg$^{-1}$ was not hepatotoxic and was only mildly hepatotoxic at 1000 mg kg$^{-1}$, as judged both histopathologically and by measurement of aspartate and alanine transaminase levels in plasma. In Balb/c mice, however, a dose of 200 mg kg$^{-1}$ caused a significant degree of hepatic damage, principally centrilobular necrosis. At doses of 400 and 800 mg kg$^{-1}$ massive haemorrhagic necrosis was observed. The level of non-protein sulphydryls in mouse liver was reduced by 75%, two hours after a hepatotoxic dose (400 mg kg$^{-1}$) of NMF. Pretreatment of rats and mice with phenobarbitone did not increase the hepatotoxicity.

The metabolism and distribution of $^{14}$C methyl labelled NMF was studied in rats. After 72 h, 62% of the dose was excreted in urine, 2% in faeces and 6.3% as expired $CO_2$. High field proton NMR spectroscopy was used to identify NMF and its metabolites in urine samples. The metabolites identified include formate, formamide and methylamine. Two resonances characteristic of acetyl groups and a group of resonances characteristic of the cysteine conjugate were also detected in the urine. Quantitative analysis of 0–24 h urine by GC, TLC and high field proton NMR revealed mainly unchanged NMF (16% of the dose), also formamide (2% of the dose), formate (4% of the dose) and methylamine (2% of the dose). The results suggest a species difference in NMF induced hepatotoxicity between Sprague Dawley rats and Balb/c mice. In the Sprague Dawley rats we found very little metabolism of NMF and only mild hepatotoxicity.

### DNA and protein adducts as indicators of *in vivo* methylation by nitrosatable drugs

P.B. Farmer & D.E.G. Shuker

*MRC Toxicology Unit, Woodmansterne Road, Carshalton, Surrey, UK.*

The analgesic drug aminopyrine is known to be carcinogenic in animals, when administered in the presence of nitrite. This is believed to be due to the intragastric production of the methylating agent dimethylnitrosamine. Many other drugs (e.g. cimetidine, oxytetracycline) have been suggested as potential hazards if ingested in the presence of nitrite. We have been exploring the extent of these possible hazards by measurements of the methylation of guanine in nucleic acids and of cysteine in haemoglobin following administration to rats of nitrosatable drugs. 7-Methylguanine, which is rapidly excised from DNA after its formation, was determined in urine samples by capillary GC-MS following its partial purification and derivatisation. Similarly, S-methylcysteine was isolated from acidic hydrolysates of globin samples. derivatised and determined by GC-MS. Stable isotope labelled drugs have been used, in order to distinguish methylation products from naturally occurring methylated materials.

For example we have observed 7-$CD_3$ guanine (47.3 nmol 24 h$^{-1}$ post dosing) in urine, following intragastric administration of a mixture of nitrite and aminopyrene labelled with 6 deuterium atoms in its dimethylamino group (100 mg kg$^{-1}$). Urinary excretion of 7-$CD_3$ guanine was undetectable after 5 days. In contrast S-$CD_3$ cysteine could be determined in globin samples at 7 days (22.7 nmol g$^{-1}$) and 15 days (10 nmol g$^{-1}$) following treatment. Its rate of loss from globin was faster than the normal turnover rate of haemoglobin in rats (lifetime ~60 days). Similar studies with cimetidine and pyrilamine have shown no evidence of methylation of guanine or of cysteine.

We hope to use this methodology to examine the potential of nitrosatable drugs to give rise to methylating agents in humans undergoing therapy.

**Immunohistochemical analysis of lymphoid and epithelial tumours using 'lineage specific' including MHC class II monoclonal antibodies (MO-ABS)**

F.G. Hay[1], C.M. Steel[2], P. Elder[2], B. Cohen[1] & R.C.F. Leonard[1]

[1]*Department of Clinical Oncology and* [2]*MRC Clinical and Population Cytogenetics Unit, Western General Hospital, Edinburgh, Scotland, UK.*

Most human tumour preparations contain a mixture of lymphoid and other 'reactive' cells. The value of immunocytochemistry in assigning lineage in these situations depends upon morphology and the use of truly lineage-specific MO-ABS or technically complex cell sorting and double labelling. A library of MO-ABS, including 'lineage specific' markers, has been applied to human epithelial lymphoid tumours freshly obtained and/or 'purified' in cell culture. Immunoperoxidase techniques were applied to imprints and cytospin preparations. See Table below.

(1) MHC class II antigens, of which DR is a subset, are expressed in NHL but are restricted in the epithelial tumours and their cell lines. (2) The anti SCLC (299) is expressed in other epithelial but not lymphoid cells. (3) HMFG2 does show some restriction in expression in epithelial tumours. (4) The MHC pattern in ovarian cancer indicates that positivity may be due to admixed lymphoid cells. The possibility of true cross-reactivity due to expression of common antigens will be explored by biochemical techniques.

**Lymphomas positive for the Ki-1 antigen do not express macrophage markers**

D.B. Jones[1], H. Stein[2], N. Hogg[3], H. Radzun[4] & J. Gerdes[2]

[1]*University Department of Pathology, Southampton, Institutes of Pathology,* [2]*Berlin, and* [4]*Kiel and* [3]*ICRF, London, UK.*

The Ki-1 antigen has been previously described as identifying Reed–Sternberg and mononuclear Hodgkins Cells in frozen section and also staining a population of dividing cells in reactive lymph node and tonsil. Further studies have demonstrated that this marker also stains lymphomas positive for $\alpha 1$ anti-trypsin and considered as being of histiocytic origin. In this study we have examined a series of ten Ki-1 lymphomas with antibodies known to identify macrophages and monocytes in reactive and neoplastic tissue. Five of these tumours were positive for $\alpha 1$ anti-trypsin. All cases were negative with the markers UCH-M1, MO1, MO2, SHCL-3, 3.9, 44, Ki-M1, KiM6 and KiM8. Node based lymphoma in a patient previously shown to have monocytic leukaemia is positive with these reagents, but negative with Ki-1. These data indicate that whilst $\alpha 1$ anti-trypsin may be present in macrophages, its association with the Ki-1 antigen does not indicate a macrophage origin and are in keeping withm metabolic studies which show that whilst the production of $\alpha 1$ anti-trypsin is a feature of macrophage rich cell preparations, low levels of synthesis can also be seen in heavily macrophage-depleted cell cultures.

**Autoproliferative and cytotoxic responses to human tumours**

T.E. Roberts and M. Moore

*Department of Immunology, Paterson Laboratories, Christie Hospital and Holt Radium Institute, Manchester, M20 9BX, UK.*

Peripheral blood T cells activated *in vitro* by

**Table**

| | | [a]$B_1$ | [a]$B_2$ | [a](MHC)PAN | [a]DR | TF | [b]229 | [c]HMFG2 |
|---|---|---|---|---|---|---|---|---|
| [a]Lymphoma | F | 0/4 | 1/4 | 4/4 | 4/4 | 4/4 | 0/3 | 0/3 |
| lymphoblastoid | C | 6/6 | 3/6 | 6/6 | 6/6 | 6/6 | NT | NT |
| [b]Small cell | F | 0/3 | 1/1 | 1/7 | 0/8 | 1/5 | 8/8 | 0/5 |
| lung cancer | C | 0/1 | 0/1 | 0/1 | 0/1 | 0/1 | 1/1 | 1/1 |
| [c]Ovarian cancer | F | 2/2 | 2/2 | 2/2 | 1/1 | 1/2 | 2/2 | 2/2 |
| | C | 2/2 | 2/2 | 0/2 | 0/2 | 0/2 | 2/2 | 2/2 |

F = Fresh specimens. C = Cell lines. $B_1$, $B_2$ from L. Nadler; HMFG2, J. Taylor-Papadimitriou; TF, G. Brown.

cocultivation for 6 days with autologous tumour cells originating from pleural effusions or lymph node metastases, were cultured in IL-2 and cloned by limiting dilution. Helper function was assayed in the primed lymphocyte test (PLT) and cytotoxic activity (CTX) by $^{51}Cr$ release against autologous and allogeneic tumour targets and NK-sensitive K562 cells. Of 9 clones assayed against tumour BA (ovarian ca.), 7 exhibited autologous but not allogeneic or NK-like CTX, one expressed NK-like activity, while another possessed no CTX. None of the clones (3 of which were of T4, and 6 of T8 phenotype) lysed allogeneic peripheral blood mononuclear cells (PBMC). By contrast, clones (two $T4^+$, three $T8^+$) generated against a breast carcinoma (BR) expressed only NK-like activity, autologous and allogeneic targets proving uniformly resistant. A renal carcinoma (CW) generated clones with similar activity, but with lesser frequency. Unlike BR, however, CW clones (mostly $T4^+$) were reactive in the PLT to autologous but not allogeneic tumour cells. $T4^+$ clones developed against HK tumour (ovarian ca.) also exhibited helper activity, which was not noticeably enhanced by tumour cell pretreatment with IFN-$\gamma$. Concomitant NK-like and autologous CTX was a feature of these clones, though the majority were cytolytically inactive. The data indicate that (i) cultured T cells (CTC) generated in mixed tumour: lymphocyte cultures may possess tumour-associated cytotoxic and/or helper activity; (ii) CTC of classical T cell phenotype may also show NK-like activity and (iii) phenotyping provides only a limited indication of functional status.

**Lymphokine-activated killing of fresh human leukaemias and solid neoplasms**

M.M. Dawson[1], T.E. Roberts[1], M. Moore[1] & G.M. Taylor[2]

[1]*Department of Immunology, Paterson Laboratories, Christie Hospital and Holt Radium Institute; and* [2]*Department Medical Genetics, St Mary's Hospital, Manchester, UK.*

Culture of normal non-immune peripheral blood lymphocytes (PBL) for a minimum of 2 days in Interleukin-2 (IL-2) results in the generation of lymphokine-activated killer (LAK) cells, as defined by amplified killing of cell-line targets. These effectors purportedly differ from NK cells, among other factors by their capacity to lyse tumours which have not been adapted to tissue culture and from cytotoxic T lymphocytes (CTL) because killing is non MHC-restricted. The susceptibility of fresh tumours to lysis by LAK was investigated using a panel of autologous and allogeneic PBL activated *in vitro* by MLA-144 containing IL-2 supernatants or recombinant IL-2 (Biogen). Cytotoxicity was donor-dependent, the susceptibility of 8/12 myelomonocytic leukaemias being comparable only with that of relatively LAK-resistant B lymphoblastoid cell lines. However, 4 leukaemias showed slightly greater susceptibility, one of them (E84) from a patient surviving 5 years. Autologous LAK was demonstrable in the remission lymphocytes of this patient. E84 cells failed to cold inhibit lysis of erythroleukaemic K562 cells suggesting recognition by LAK of different target structures. Using solid tumours as targets, LAK by normal allogeneic PBL has been observed, to date, in 2/11 combinations and by autologous PBL in 1/3 combinations and cytotoxicity (at E:T ratio, 20:1) has not exceeded 25% (against a carcinoid tumour). The factors which determine sensitivity of fresh tumour agents, of whatever provenance, to LAK, are unknown.

**Comparison of an immunoperoxidase and a biochemical technique in assessing oestrogen receptor content of human breast tumours**

R.A. McClelland[1], E. Jensen[2], L. Miller[3], U. Berger[1] & R.C. Coombes[1]

[1]*Ludwig Institute for Cancer Research (LICR) (London Branch), Royal Marsden Hospital, Sutton, Surrey, SM2 5PX, UK;* [2]*LICR, Zurich, Switzerland; and* [3]*Abbott Laboratories, Diagnostics Division, North Chicago, USA.*

An immunocytochemical procedure for determining the presence of oestrogen receptor (ER) in human breast tissue is currently being evaluated in our laboratory with respect to its comparability with the widely used dextran coated charcoal method (DCC) (McGuire & De La Garza (1973), *J. Clin. Endocrinol. Metab.* **37**, 986). Using an indirect immunoperoxidase technique and a rat anti-human ER monoclonal antibody (H222 Sp$\gamma$) we have been able to show specific staining of nuclear oestrogen receptor in many frozen, formol-saline fixed sections of breast tumour (68%).

Parallel DCC and immunocytochemical (ER/ICA) assays on stored breast tumour biopsies have to date given good qualitative correlation of results (Table). A quantitative comparison of results has also been attempted by utilising a mean staining intensity index for ER/ICA and the concurrence of results is again good.

**Table**

| ER/ICA | +ve | −ve | Total (% correlation) |
|---|---|---|---|
| +ve > 15 f mol $mg^{-1}$ cyt. protein | 24 | 1 | 25 (96) |
| DCC −ve < 10 f mol $mg^{-1}$ cyt. protein | 1 | 9 | 10 (90) |
| borderline 10–15 f mol $mg^{-1}$ cyt. protein | 1 | 2 | 3 |
| Totals | 26 (68%) | 12 (32%) | 38 |

Results obtained so far suggest that ERICA, with its inherent advantages in terms of the speed of assessment and its requirement for only small quantities of tissue, may in the future, represent a valuable means of selecting oestrogen dependent tumours for endocrine therapy (McGuire & De La Garza (1973), *J. Clin. Endocrinol. Metab.* **37**, 986).

### Relationship of sialyltransferase activity in the serum of breast cancer patients to tumour burden

J. Nelson[1], B.J. McDermott[2], L. Jeffrey[3] & R.F. Murphy[1]

[1]*Department of Biochemistry, Queen's University of Belfast;* [2]*Clinical Oncology Unit, University of Bradford; and* [3]*Belvoir Park Hospital, Belfast, UK.*

Increases in sialyltransferase (ST) activity in serum have been commonly reported to correlate with progression of neoplastic disease, but in a preliminary study in post-mastectomy patients, enzyme levels were not associated uniformly with tumour burden (McDermott *et al.* (1982), *Br. J. Cancer,* **46**, 511). ST activities have now been measured in the sera of 135 patients of which 64 were monitored for periods up to 32 months. In a vertical study, patients were divided into 3 groups: I clinically tumour-free; II, minimal disease (lymph-node involvement and recurrence); III, metastatic disease. SR activities (mean ± s.e.) were 5.77±0.34 8.31±0.41 and 7.12±0.53 p mol sialic acid transferred $mg^{-1}$ protein $h^{-1}$ and the % of values above the upper limit of normal (8.2 units) were 12.2, 53.2 and 25.6 for Groups I, II and III respectively. A prognostic significance was observed for Group I patients who did not receive treatment, in which the mean initial value of ST in cases where disease progressed (5.93 units) was greater ($P<0.05$) than in cases of stable disease (4.26 units). Also, in Group II patients, the mean pre-treatment value of ST in the sub-group who progressed (8.65 units) was greater ($P<0.01$) than in that which regressed (5.70 units). When lymph-node or systemic metastases developed in 22 patients in Groups I or II, ST levels elevated synchronously in 5 cases but more often were raised before the overt development of metastatic disease: ST activity in serum reached a peak and then declined up to 13 months before the clinical detection of progressive disease in 10 of these patients. The sensitivity of ST for detecting early metastatic disease is consistent with the consideration that the enzyme maybe a measure of the metastatic potential of the tumour.

### Aggressive short duration chemotherapy and radiotherapy in locally advanced nonmetastatic breast cancer – report of a pilot study

A. Rodger[1], J.F. Smyth[1], R.F. Leonard[1], O. Eremin[2] & U. Chetty[2]

*Departments of* [1]*Clinical Oncology and* [2]*Clinical Surgery, Edinburgh University, UK.*

Twenty-five patients with locally advanced nonmetastatic breast cancer underwent chemotherapy (CHOP) with cyclophosphamide (1000 mg $m^{-2}$), adriamycin (50 mg $m^{-2}$) vincristine (1.4 mg $m^{-2}$ to a maximum of 2 mg) and prednisolone (40 mg $day^{-1}$ orally for 5 days) given on a 3 weekly cycle for a total of 4 courses. One month later they commenced a course of megavoltage X-ray therapy to the breast and peripheral lymphatics given in 20 fractions over 4 weeks to a maximum tissue absorbed dose in the breast, central axilla and supraclavicular fossa of 45–50 Gy. If appropriate one month later a boost to the axilla and/or breast mass was given using 10 MeV electrons (15 Gy in 5 fractions) or iridium implant (reference dose 2–30 Gy). Toxicity was acceptable and patient compliance was 100%. Only 5 patients experienced any delay in planned courses of chemotherapy. Acute radiation skin reactions were not enhanced by the preradiation chemotherapy and were radiation dose related. After chemotherapy there was a complete remission (CR) of 20% and partial remission (PR) of 48% with only 8 patients (32%) showing static or progressive disease. Following the radiotherapy the overall CR was 64% and PR 32% with only 1 patient (4%) who failed to show any response to combined treatment. Persistent local control at time of evaluation (4–30 months) was achieved in 14/25 (56%). Local control was not related to menstrual status, oestrogen receptor ($E_2R$) level, primary tumour size, extent of nodal metastases, or radiation boost. $E_2R$ level predicted response to neither chemotherapy nor to radiotherapy. The crude 1 and 2 year survivals are 83.3% and 71.4% respectively. This regime has the advantage of excellent compliance and acceptable morbidity.

### Preliminary characterisation of a tamoxifen resistant variant of the oestrogen responsive human breast cancer cell line ZR-75-1

H.W. Vandenberg[1] & R. Clarke[2]

*Departments of [1]Therapeutics and [2]Biochemistry, The Queen's University of Belfast, Northern Ireland, UK.*

The oestrogen receptor (ER) positive human breast cancer cell line ZR-75-1 is sensitive to growth inhibition by the antioestrogen tamoxifen. In an attempt to generate a tamoxifen-resistant variant line, parent cells were grown in the presence of $10^{-6}$M tamoxifen for a period of two weeks followed by a further two weeks in the presence of $2 \times 10^{-6}$ tamoxifen. Surviving cells were then grown in the absence of antioestrogen for 2 further weeks prior to assessment of the effect of tamoxifen on DNA synthesis and cell proliferation. Whilst DNA synthesis, as measured by [3H] thymidine incorporation, and cell proliferation were inhibited in a dose-dependent manner by tamoxifen, ($10^{-8}$M–$10^{-6}$M) in parent cells, the antioestrogen failed to significantly affect these parameters in the variant line, (designated ZR-75-1R), at concentrations up to $10^{-6}$M. Determination of ER levels in the two lines using a whole cell binding assay revealed that the ZR-75-1R line contained ~20% of the oestrogen binding capacity of the parent line. Resistance to tamoxifen in this variant line therefore correlates with a reduction in ER levels, in contrast to an earlier report of a resistant sub-line of MCF-7 cells which retained an ER content equivalent to wild type cells, (Nawata *et al.*, (1981) *J. Biol. Chem.* **256,** 5016).

### The treatment of premenopausal patients with breast cancer with buserelin nasal spray

S.J. Harland[1], J.H. Waxman[2], L. Rees[2], H.T. Ford[1], J.C. Gazet[1], A. Nash[1], T.J. Powles[1] & R.C. Coombes[1]

*[1]Royal Marsden and [2]St Bartholomew's Hospitals, London, UK.*

Luteinising hormone-releasing hormone (LHRH) analogues suppress pituitary gonadotrophin release, and have been used as an alternative to artificial menopause in breast cancer patients (Klijn & de Jong (1982), *Lancet* **i,** 1213). We have treated 13 premenopausal patients with the LHRH analogue, buserelin, given by nasal spray.

Gonadotrophin response to LHRH injection, assessed before treatment and after 4 weeks, showed satisfactory pituitary inhibition. Significant changes in sex hormone levels were not seen however: mean plasma oestradiol levels were 453 (range: <50–1400) $pmol\,l^{-1}$ before treatment and 284 (range: 130–870) $pmol\,l^{-1}$ 4 weeks after starting. Only one patient experienced hot flushes, but all 5 patients who remained on buserelin for 3 months stopped having normal periods.

Two partial responses, one minimal response and two disease stabilisations were seen. Five patients had an artificial menopause at disease progression. Two patients responded at this stage. Although buserelin nasal spray produces tumour responses in premenopausal patients with breast cancer it is not effective as artificial menopause.

### 4-hydroxyandrostenedione – A new treatment for postmenopausal patients with metastatic breast cancer

P. Goss[1], R.C. Coombes[2,3], T.J. Powles[3], M. Dowsett[4] & A. Brodie[5]

*[1]Cancer Research Campaign; [2]Ludwig Institute for Cancer Research; [3]Royal Marsden Hospital, Sutton, Surrey SM2 5PX; [4]Department of Endocrinology, Chelsea Hospital for Women, London, SW3, UK; and [5]University of Maryland, Baltimore, Maryland, USA.*

4-Hydroxyandrost-4-ene-3,17-dione (4-OHA) is a potent, selective inhibitor of the aromatase enzyme complex responsible for the conversion of androgenic precursors to oestrogens. Thirty-nine postmenopausal patients with metastatic breast cancer have been treated with once weekly i.m. injections of 500 mg 4-OHA. Of 19 assessable patients to date 9 have responded for treatment for periods up to 9 months. Healing of bone metastases and palliation of pain as well as reduction and resolution of soft tissue metastases have occurred. Troublesome pain and induration at the injection sites has been decreased by deep i.m. injection and formulation of the compound in a micronised form. Occasional vaginal bleeding and hot flushes have been reported. After a single 500 mg i.m. injection, a sustained reduction in plasma oestradiol levels was observed for at least one week in 5 patients. Both the oral and vaginal route of administration are currently being investigated.

4-OHA is a promising new agent in the management of breast cancer.

**Prednisolone, adriamycin, bleomycin, vincristine and etoposide (pabloe) alternating with ChlVPP in the treatment of advanced Hodgkin's disease**

N.S.A. Stuart[1], M.H. Cullen[1], C.M. Woodroffe[1], G.R.P. Blackledge[1], E.L. Jones[2], J. Fletcher[3], J.A. Child[4], A.D. Chetiyawardana[1], D. Spooner[1] & J.J. Mould[1]

[1]*Queen Elizabeth Hospital, Birmingham;* [2]*University of Birmingham;* [3]*City Hospital, Nottingham; and* [4]*General Infirmary, Leeds, UK.*

There are theoretical attractions and some clinical data to support the use of alternating, non-cross resistant combinations of active drugs in advanced Hodgkin's Disease. The best studied approach is with MOPP/ABVD and we have modified this regime in the hope of identifying an equally effective regime with less short-term toxicity and hence greater patient acceptability.

ChlVPP has been substituted for MOPP; etoposide and vincristine substituted for dacarbazine and vinblastine in ABVD. ChlVPP comprises vinblastine $6\,mg\,m^{-2}$ i.v. on days 1 and 8, and chlorambucil $6\,mg\,m^{-2}$, procarbazine $100\,mg\,m^{-2}$ and prednisolone $30\,mg\,m^{-2}$ orally on days 1–15. PABLOE comprises prednisolone $40\,mg\,m^{-2}$ d1–15. Adriamycin $40\,mg\,m^{-2}$ (i.v. d1) bleomycin $10\,mg\,m^{-2}$ and vincristine $1\,mg\,m^{-2}$ (i.v. d1, d8) and etoposide $200\,mg\,m^{-2}$ orally daily d2–4. PABLOE commences on day 29 following ChlVPP and the whole cycle repeats on day 50. Patients resistant to conventional chemotherapy receive PABLOE alone every 21 days.

Seventy patients are currently on study with 40 evaluable for treatment response. Of 25 patients who had received no prior chemotherapy 18 (72%) achieved CR and 5 (20%) PR. Of 15 patients who had relapsed or failed after previous chemotherapy 7 (47%) achieved CR and 4 (27%) PR. The regime has been well accepted producing little nausea and vomiting. All patients experience alopecia. Infection has been the commonest complication affecting 25% of patients at some stage during treatment. There have been 4 deaths to which treatment induced neutropenia may have contributed.

**Evaluation of vincristine as an adjunct to intermittent melphalan and prednisone therapy in myelomatosis**

I.C.M. MacLennan

*Department of Immunology, University of Birmingham B15 2TJ (for the Medical Research Council's Working Party on Leukaemia in Adults).*

Vincristine has been widely used in combination with other cytotoxic agents in the treatment of myelomatosis. Evaluation of the results of this use of vincristine have resulted in claims that vincristine is an important agent in the first line treatment of this disease. However, there has been lack of objective evidence to support this conclusion. Between 1 March 1980 and 28 February 1982 the MRC Working Party on Leukaemia in Adults admitted 530 patients with previously untreated myelomatosis to its IVth trial in this disease. In this trial vincristine was added as a single randomised variable to intermittent melphalan and prednisone. Vincristine was given as 1 mg i.v. on the first day of 7 day courses of melphalan 10 mg daily orally and prednisone 40 mg daily orally. The interval between the first day of courses was 4 weeks. Patients were treated to plateau plus 6 months at which stage they were re-randomised either to continue first line therapy for a further year or discontinue treatment. Analysis of the trial to 1 February 1984 shows no survival advantage in patients allocated vincristine. Analysis by prognostic groups failed to identify any group which benefitted from the addition of vincristine. Patients randomised to stop at plateau fared slightly better than those continuing first line therapy but this difference is not significant ($P=0.2$).

**Antitumour effect of new anthracyclines in mice**

H.R. Hartmann[1], M.J. Broadhurst[2], G.J. Thomas[2] & J.A. Martin[2]

[1]*Hoffman-La Roche and Co. Ltd., Basel, Switzerland; and* [2]*Roche Products Ltd., Welwyn Garden City, Herts, UK.*

New anthracyclines prepared by total synthesis in the Roche laboratories in England were evaluated for cytostatic effects against a range of murine tumours in the Swiss laboratories.

The antitumour activity was calculated from the mean survival time of treated and control groups of mice bearing L1210 (T/C) and the mean 13C mammary tumour weight of controls relative to treated mice (C/T). In the L1210 model drugs were administered daily, 5 times per week, either i.p. or p.o. for 4 weeks or until death, whereas the solid tumour bearing mice were dosed i.p. for 2 weeks, the tumours were excised and weighed on day 15.

Toxicity was evaluated on day 15 following multiple i.p. dosing. Body weight change and white blood cell count were measured.

Many compounds showed superior efficacy and/or potency in comparison with doxorubicin. For example, the alcohol Ro 31-1741 had the same cytostatic activity as doxorubicin at a 15 times reduced dose. Two other compounds Ro 31-1215 (a 9-methyl derivative) and Ro 31-2035 (a 9-substituted urethane) showed superior inhibition of

L1210 and 13C relative to doxorubicin at similar doses, the latter compound displayed oral activity against L1210. Another urethane Ro 31-2383 was more potent and more efficacious than doxorubicin, and also had oral activity against L1210.

### New anthracyclines. *In vitro* effects and cross-resistance patterns in control and adriamycin-resistant cell lines

P.R. Twentyman[1], N.E. Fox[1], K.A. Wright[1], P. Workman[1], M.J. Broadhurst[2] & J.A. Martin[2]

[1]*MRC Clinical Oncology and Radiotherapeutics Unit, Hills Road, Cambridge; and* [2]*Roche Products Ltd., Welwyn Garden City, UK.*

A range of new anthracyclines, structurally related to adriamycin, has been synthesised by Roche Products Ltd. and studied *in vitro*. Three compounds being evaluated extensively in preclinical studies are shown below. In the EMT6/VJ/AC mouse tumour cell line, the continuous drug concentration to inhibit colony formation by 90% was similar for ADM, 1741 and 1215, The value for 2035 was higher by a factor of 10. For 1 h drug incubation followed by colony growth in drug-free medium, 1741 and 1215 were 3 × more potent than ADM whilst 2035 was 3 × less potent. In the human small cell lung cancer line NCI-H69 results for continuous drug exposure were similar to those for the mouse cells. In mouse models, 2035 showed greater potency and efficacy than would have been predicted from these *in vitro* results (Hartmann *et al.*, this meeting). ADM-resistant sublines of the two cell lines have also been studied. For compounds 2035 and 1741 there was complete cross-resistance (CR) for both lines. For 1215, however, there was only partial CR in the mouse cells and a total absence of CR in the human line. A subline of the mouse cells selected for continuous growth in 1215 was resistant to both 1215 and ADM. The cellular pharmacology of ADM and 1215 in control and resistant cell lines is being studied.

Table

| *Drug* | $R_1$ | $R_2$ | $R_3$ | $R_4$ |
|---|---|---|---|---|
| Adriamycin | OH | H | $COCH_2OH$ | $OCH_3$ |
| Ro 31-2035 | H | OH | $CH_2OCONHPh$ | H |
| Ro 31-1741 | H | OH | $CH_2OH$ | H |
| Ro 31-1215 | OH | H | $CH_3$ | H |

### 9-Alkyl anthracyclines. Absence of cross resistance in a human cell line

C.A. Scott, D. Westmacott, M.J. Broadhurst, G.J. Thomas & M.J. Hall

*Roche Products Limited, Welwyn Garden City, Herts AL7 3AY, UK.*

Twentyman *et al.* have observed (this meeting) that a novel 9-methyl anthracycline Ro 31-1215 indicated absence of cross resistance in an adriamycin (ADM) resistant human cell culture. We have investigated the resistance pattern in a series of related 9-alkyl anthracyclines using a human T-lymphoblastoid line.

The compounds examined were the 9-methyl and 9-ethyl substituted 4-demethoxy anthracyclines containing the daunosamine or 4′-epi-daunosamine sugar moiety (Ro 31-1215, Ro 31-1740, Ro 31-1749 and Ro 31-1966).

The parental cell line (CCRF CEM) was rendered resistant by several serial passages in medium RPMI 1640 containing ADM at 0.01 $\mu g\,ml^{-1}$. This produced a 2.3 to 5-fold increase in resistance factor to ADM and related compounds. Resistance to the 9-alkyl analogues was then determined by continuous exposure of the cell cultures over a period of 4 days followed by assessment of cell growth.

Without exception all standard compounds evaluated, including 4′-epi-adriamycin, daunomycin, mitoxanthrone and also novel compounds not possessing 9-alkyl substituents were cross resistant with ADM.

By contrast, the cell line rendered resistant to ADM retained full sensitivity to all four 9-alkyl analogues irrespective of the configuration of the sugar residue.

### Human liver microsomal generation of doxorubicin and mitoxantrone free radicals

J. Basra[1], C.R. Wolf[2] & L.H. Patterson[1]

[1]*School of Pharmacy, Leicester Polytechnic, Leicester LE1 9BH; and* [2]*Imperial Cancer Research Fund, Medical Oncology Unit, Western General Hospital, Edinburgh, EH4 2XU, UK.*

Liver microsomes from animals are well established as a source of enzymes mediating doxorubicin and related anthraquinone free radical formation and redox cycling. Such studies are often used to rationalise the activities of these antitumour agents in humans (Olsen *et al.*, (1981), *Life Sci.*, **29**, 1393).

In this study we have investigated the occurrence of doxorubicin and mitoxantrone free radical formation in human liver microsomes (HLM) prepared from five organ transplant donors. Cytochrome P450 content ($0.22 + 0.017\,nmol\,mg^{-1}$ protein) and cyt.c reductase activity ($166.4 \pm 44.0\,nmol\,mg^{-1}$ protein $min^{-1}$) were similar to that described for various animals (Pelkonen *et al.* (1974) *Chem. Biol. Interact,* **9,** 205). NADPH fortified HLM under anaerobic conditions generated doxorubicin free radicals ($g = 2.0038$) and mitoxantrone free radicals ($g = 2.0036$) when incubated with respective parent compound as monitored by electron spin resonance spectrometry (as described by Oldcorne *et al.* (1984) *Biochem. Soc. Trans.,* **12,** 681). Basal rates of NADPH oxidation ($8.5$–$18.4\,nmol\,mg^{-1}$ protein $min^{-1}$) and superoxide formation ($32.3$–$114.2\,nmol\,mg^{-1}$ protein $min^{-1}$) measured in individual liver samples, (Kharasch and Novak (1983) *Arch. Biochem. Biophys.,* **224,** 682), were significantly stimulated by doxorubicin. However, mitoxantrone only slightly increased NADPH oxidation and had little effect on the basal rate of superoxide formation. The results show that doxorubicin and mitoxantrone free radicals are generated in HLM but that only doxorubicin participates in a redox cycle that leads to a significant increase in superoxide formation. Also the results support the use of animal models to investigate drug activation in humans.

### Intracellular uptake of 4′-deoxydoxorubicin and adriamycin by human lung tumour cells in culture and its relationship to cell survival

D.J. Kerr, A.M. Kerr, R.I. Freshney & S.B. Kaye

*Department of Clinical Oncology, University of Glasgow, Glasgow G12, 9LX, UK.*

4′-Deoxydoxorubicin (4′) is an anthracycline antitumour agent with a similar spectrum of activity to adriamycin (A). Although differing only in substitution of a hydroxyl grouping on the daunosamine sugar the 4′ derivative is considerably more lipophilic than A, and might have an enhanced rate of cell uptake. Non-small cell lung tumour cells (L-DAN) were maintained as a monolayer in exponential growth in Hams F-10 medium supplemented with foetal calf serum. The cells were exposed to varying drug concentrations for different times and were then harvested with 0.25% trypsin. The cells were then centrifuged, washed twice and resuspended in distilled water. Drug and metabolites were extracted using a standard procedure and were measured by reverse phase HPLC utilising a fluorescence detector. Cell survival was determined by clonogenic assay. The time course of drug uptake showed that intracellular drug concentration depended on drug concentration in the medium and the duration of drug cell contact. The rate of uptake of 4′ ($Vmax = 30\,ng\ 10^{-5}\,cells\,min^{-1}$) was far greater than A ($Vmax = 0.15\,ng\ 10^{-5}\,cells\,min^{-1}$) and consequently higher intracellular drug levels were achieved for 4′. It was possible to detect intracellular metabolism of 4′ to its alcohol but this may have been due to the high intracellular levels of parent drug allowing metabolite detection. There was a strong correlation between intracellular drug concentration and survival of exponentially growing cells, however, there was only a minor difference in the cell survival curves at low drug concentrations comparing 4′ and A. We would conclude that substitution at the 4′ position of adriamycin enhances cell uptake but does not improve cytotoxicity.

### Marked inter-patient variations in the metabolism of adriamycin to 7-deoxyaglycone metabolites

J. Cummings[1], R. Milsted[1], G.J. Forrest[2], D. Cunningham[2] & S.B. Kaye[1]

[1]*Department of Clinical Oncology. University of Glasgow, Glasgow G12 9LX; and* [2]*Royal Infirmary, Glasgow, G4 0SF, UK.*

Although several studies have observed large inter-patient variations in the pharmacokinetics of Adriamycin (ADR) few have detected metabolites present in serum or considered metabolism as a serious factor contributing to these variations. In 25 patients with normal liver and kidney function, in most cases receiving ADR (30 or $40\,mg\,m^{-2}$ i.v.) for the first time, three major metabolites were identified which accounted for between 97–100% of the total serum concentration of fluorescent metabolites. The total serum concentration of ADR and metabolites was determined by integrating the area under the concentration/time profiles over 24 h (AUC). Adriamycinol (AOL), the most abundant species in 21/25 patients, accounted for between 5 and 26% of the total serum concentration of ADR and its metabolites (TSC). Adriamycin 7-deoxyaglycone (ADR-DONE) was not detected in 10/25 patients and in the others it accounted for only a small proportion of the TSC (1–5%). Adriamycinol 7-deoxyaglycone was nor detected in 12/25 patients, but when present accounted for a more substantial amount of the TSC (10–20%) and in 4/25 patients was the predominant metabolite species. Its AUC exhibited a large inter-patient variation (from $3\,ng\,ml^{-1} \times h$ to $240\,ng\,ml^{-1} \times h$). There was a correlation between degree of metabolism and the AUC of ADR in serum ($r=0.73$). The greater the TSC of the metabolites the less the AUC of ADR. Metabolism may partly, if not wholly, explain certain aspects of inter-patient variations in the pharmacokinetics of ADR. In turn, variations in metabolism are dictated, to a large degree, by whether or not ADR and AOL are converted to 7-deoxyaglycones, a process which may have a bearing on patient toxicity and response.

**Mechanisms of resistance and its reversal in a daunorubicin insensitive P388 cell line**

A.T. McGown, T.H. Ward & B.W. Fox

*Paterson Laboratories, Christie Hospital and Holt Radium Institute, Manchester M20 9BX, UK.*

A P388 cell resistant to the anthracycline antibiotic daunorubicin ($ID_{50}=650$ nm) has been developed from the parental line ($ID_{50}=19$ nm) *in vitro*. Resistance to this agent has been demonstrated to be associated with a 4-fold decreased cellular accumulation of the drug: both by direct quantitation of intracellular anthracycline levels, by flow cytometry, and by extraction of drug from cell homogenates. The cell cycle position of the cells is shown to affect the overall level of drug accumulation, and subsequent cell survival. An increase in DNA content is associated with increased drug incorporation and decreased cell survival.

The patterns of cross-sensitivity of the daunorubicin resistant cell line towards other anthracyclines, anthracycline analogues, and mitotic inhibitors correlates with the ability of the cells to exclude drug.

Daunorubicin levels can be increased in the resistant cell line by metabolic inhibition or by simultaneous incubation with the vinca alkaloids.

A group of novel compounds are also shown to inhibit the efflux of daunorubicin from the resistant cell line, resulting in cellular accumulation equal to that observed in the parental line.

**Phase I and II study of oral verapamil (VRP) and intravenous vindesine (VDN)**

B. Cantwell, P. Buamah & A.L. Harris

*University Department of Clinical Oncology, Newcastle General Hospital, Newcastle upon Tyne, UK.*

The calcium antagonist VRP enhances the cytotoxicity of vinca alkaloids in tumour cell lines (Tsuruo (1983), *Cancer Treat. Rep.* **67**, 889). We treated 23 patients in Phase I with advanced cancer with escalating doses of oral VRP either continuously (c) or intermittently (i) and i.v. VDN every 2 weeks until progression or 6 courses of VDN. VRP (i) was given the day before, the day and 2 days after i.v. VDN. Fifteen patients had previous chemotherapy. In the Phase I study, VRP was given 80 mg 3 × day (c); at each VDN dose level 3 patients were treated (2 mg, 3 mg, 4 mg, 5 mg i.v. q 2 weeks). Four patients received 5 mg VDN with VRP 160 mg 3 × day (c). VRP (i) was then given to reduce toxicity. Final dose escalation was 7 mg VDN q 2 weeks, VRP 160 mg 3 × day (i). Eighteen patients have been treated in Phase II. In 12 so far assessable, 3 responses have occurred (1 hypernephtoma, 1 squamous, 1 adenocarcinoma lung). Phase I and II toxicity was alopecia (37%), myelosuppression (9%), neurotoxicity (20%), constipation (14%), symptomatic postural hypotension (17%), ECG abnormalities (9%). $\alpha_1$ acid glycoprotein, the major plasma binding protein for VRP was raised in patients and VRP plasma levels were $>350$ ng ml$^{-1}$ in 6 of 7 patients who received VRP 480 mg daily. Levels $>350$ ng ml$^{-1}$ have been associated with *in vitro* cytotoxic potentiation, and in animals improvement of tumour blood flow occurred at plasma VRP levels $>100$ ng ml$^{-1}$ (Kaelin *et al.* (1982), *Cancer Res.* **42**, 3944). VRP is being assessed in a randomised trial.

**Investigations of mechanisms of resistance to etoposide in a human tumour continuous cell line**

R.B. Lock & B.T. Hill

*Laboratory of Cellular Chemotherapy, Imperial Cancer Research Fund, Lincoln's Inn Fields, London WC2A 3PX, UK.*

A human tumour cell line derived from an epithelioid carcinoma of the tongue has been made resistant to etoposide (VP-16-213) following fractionated X-irradiation exposure (total 22.5 Gy) or continuous drug exposure (100 ng ml$^{-1}$). The order of resistance is 5- and 7-fold for the X-irradiation treated and drug treated sub-lines respectively, as established by assessing cell survival, following drug treatment (0.85 $\mu$M for 24 h), by clonogenic assay (Courtenay *et al.* (1978), *Br. J. Cancer*, **38**, 77). The mechanisms of resistance of these sub-lines investigated include (i) levels of non-protein sulphydryl compounds, (ii) drug transport, and (iii) analysis of DNA damage after drug treatment.

No significant differences in glutathione content have been found between parent and resistant sub-lines, with values of 45–60 nmol mg$^{-1}$ total cellular protein in logarithmically growing cultures, suggesting that glutathione is not involved in the mechanisms of resistance to etoposide. Etoposide resistant sub-lines exhibit cross-resistance with vincristine, and the drug treated sub-line has been found to accumulate after 30 min only 33% (30.4 pmol $mg^{-1}$ total cellular protein) of radioactively labelled vincristine compared to the X-irradiation treated sub-line (92.9 pmol $mg^{-1}$ total cellular protein), indicating that different mechanisms of resistance may be involved. Earlier studies (Hill & Bellamy (1984), *Int. J. Cancer*, **33**, 599) showed no difference in alkaline sucrose gradient sedimentation profiles between parent and an X-irradiation treated sub-line after a 24 h exposure to etoposide. However, after a shorter exposure time (1 h) the profiles suggest that both this X-irradiation treated and the drug treated sub-lines may sustain less DNA damage than the parental line.

### Flow cytometric studies of the vinca alkaloids on populations of parental L1210 and gene-amplified methotrexate resistant cells

D.G. Poppitt, A.T. McGowan & B.W. Fox

*Paterson Laboratories, Christie Hospital and Holt Radium Institute, Manchester M20 9BX, UK.*

Comparative studies of the effects of three vinca alkaloids on two cell lines, differentially sensitive to methotrexate have been undertaken. Using propidium iodide staining of the DNA following vinca alkaloid treatment at doses up to $10^{-7}$ M, a build up of fluorescence was observed at the 4n position. On an equimolar basis, the intensity of this effect decreased in the order vinblastine > vindesine > vincristine. Following continuous exposures to a concentration of $10^{-8}$ M vincristine, and above, an accumulation of material at the 8n position was observed from the DNA fluorescent histograms. This was shown by chromosome analysis to be due to polyploidy. The median chromosome count in the L1210 was $38.2 \pm 1.4$ for the untreated sample and $71.2 \pm 12.5$ for a 48 h $10^{-7}$ M vincristine treated sample. The corresponding values for the L1210/R7A line were $37.2 \pm 1.5$ and $80.4 \pm 19.7$ respectively. By careful adjustment of the level of vinblastine to a mixed population of sensitive and resistant cells, the selective removal of the latter population has been demonstrated at a concentration of $5 \times 10^{-8}$ M.

### A 'spectrum of sensitivity' model which might explain the development of resistance to cytotoxic drugs in some tumours

T.C. Stephens, L. Fiszer-Maliszewska, J.H. Peacock & T.J. McMillan

*Radiotherapy Research Unit, Institute of Cancer Research, Sutton, Surrey, UK.*

One model of induced drug resistance envisages that tumour cells may be heterogeneous with respect to their sensitivity to certain cytotoxic drugs and that tumour resistance may emerge due to the gradual selection of the more resistant variants during repeated treatment regimes.

In experimental studies we have begun to examine this model by measuring the sensitivity of clones of cells selected from tumours. Using MT carcinoma, we have already shown that cell populations derived from individual lung colonies exhibit a spectrum of sensitivities to melphalan *in vitro* ($D_{10}$ range 0.35–0.98 $\mu g\,ml^{-1}$) but there seems to be less variation in the sensitivity of Lewis lung tumour cells to MeCCNU ($D_{10}$ range 1.5–2.39 $\mu g\,ml^{-1}$).

At a theoretical level, we have explored the shape of the distribution of cellular sensitivities (in terms of survival curve slope) that is needed to give substantial levels of drug resistance following repeated drug treatments. The sensitivity distribution must have a long tail reflecting the presence of some highly resistant cells. One of the consequences of assuming that the individual cells in a tumour vary in drug sensitivity is that the acute survival curve will tend to bend upward with increasing dose, but this may not be distinguishable in cell survival studies due to scatter in the data. The model predicts that resistance may not develop if very high drug doses are used, due to the 'extinction' of small populations of resistant cells.

### Characterisation of two L1210 leukaemias

P.M. Goddard, R.M. Orr, M.R. Trafford, A.W.R. Payne & K.R. Harrap

*Department of Biochem. Pharmacol., Institute of Cancer Research, Sutton, Surrey, UK.*

The quinazoline antifolate, CB3717, a potent thymidylate synthetase inhibitor, now undergoing clinical evaluation, failed to show significant antitumour activity when screened against a panel of rodent tumours which included the TLX/5 lymphoma, ADJ/PC6 plasmacytoma and Walker carcinosarcoma 256. However, CB 3717 treatment of two L1210 murine leukaemia ascitic tumours, designate L1210/ICR and L1210/NCI led to long-term survivors in mice bearing the L1210/ICR line whereas only a moderate antitumour response was noted for the L1210/NCI tumour. DNA cytofluorimetric studies identified a ploidy difference between these two wild type L1210 lines. The L1210/ICR being tetraploid and the L1210/NCI, diploid. Their ploidy characteristics were retained when they were established in long-term suspension culture and *in vitro* $ID_{50}$ values for CB 3717 were similar. When the L1210/ICR tumour was innoculated intraperitoneally into $BDF_1$ mice a spread in the day of death was observed. However, this spread was also noted in the syngeneic $DBA_2$ host and the athymic nude mouse indicating that antigenicity was not a major contributing factor to the behaviour of this cell line. Preliminary studies on tumour migration after initial i.p. innoculation of $DBA_2$ mice showed tumour cells in the liver, spleen and kidney from both cell lines by day 3 and in the bone marrow of mice bearing the L1210/ICR tumour. When other cytotoxic agents were screened against the two L1210 lines *in vivo* there were no differential antitumour responses. The marked sensitivity of the tetraploid L1210/ICR tumour to CB 3717 *in vivo* may be due to a higher thymidine requirement for DNA synthesis or other cellular factors as yet unidentified.

**Suitability of NMU rat model for testing endocrine agents for breast cancer**

J.R. Wilkinson & R.C. Coombes

*Ludwig Institute for Cancer Research (London Branch), Royal Marsden Hospital, Sutton, Surrey SM2 5PX, UK.*

Currently, the DMBA and NMU induced rat mammary tumour is the standard screen for possible endocrine agents for use in patients with breast cancer. Using the NMU rat model, we compared response in rats and patients to a variety of treatments, e.g. tamoxifen, danazol, aminoglutethimide singly and in combination, ovariectomy, triolastane and 4-hydroxyandrostenedione(4-OHA) and from this determined whether it is possible to rank various therapies for clinical evaluation. The correlation is moderately good for premenopausal women, the exception being 4-OHA. For post menopausal women Aminoglutethimide is inactive in the rat model, but extremely active in patients. This study supports the use of such a model for selecting endocrine agents but does not predict whether the drug will be effective in pre- or post-menopausal women. For adequate evaluation, the mode of action of the drug and its metabolism should be similar in both species, and these criteria should be compared before committing large numbers of patients to clinical trials.

**The effects of α and γ interferons on human lung cancer cells grown *in vitro* or as xenografts in nude mice**

P.R. Twentyman, P. Workman, K.A. Wright & N.M. Bleehen

*MRC Clinical Oncology and Radiotherapeutics Unit, Hills Road, Cambridge, UK.*

We have compared the effects of α and recombinant γ interferons (IFNs) on the growth of human lung cancer cell lines *in vitro*. There was a diversity of response amongst the lines studied, several being unresponsive and the most sensitive being COR-L23 (a large cell anaplastic carcinoma line) and POC (a small cell line). In general, IFN-γ was found to be more potent that IFN-α. For the lines COR-L23 and POC the response to recombinant IFN-γ was dose-related in the region 0–$10^4$ IU $ml^{-1}$ with the major part of the effect occurring below $10^3$ IU $ml^{-1}$. During cell growth of line POC in the presence of IFN-γ no significant shift in cell cycle distribution occurred. When lines COR-L23 and POC were grown as xenograft tumours in nude mice, daily injection of $4 \times 10^5$ IU $mouse^{-1} day^{-1}$ of IFN-γ produced no discernible retardation of tumour growth.

**Potentiation of *cis*-platinum activity by interferon in non-small cell lung cancer xenografts**

J. Carmichael, C.R. Wolf, F.R. Balkwill, R.C.F. Leonard & J.F. Smyth

*Imperial Cancer Research Fund Medical Oncology Unit, Edinburgh University and London, UK.*

In recently confirming the activity of *cis*-platinum (CP) for the treatment of non-small cell lung cancer (NSCLC) we found the toxicity unacceptable for the modest response rate achieved. To explore ways of enhancing the therapeutic ratio of CP for NSCLC, squamous, adenocarcinoma and adenosquamous lung tumour xenografts established in CBA mice immunosuppressed by neonatal thymectomy, cytosine arabinoside and TB1 have been treated with CP, analogues of CP or cyclophosphamide (CTX) ± human α lymphoblastoid interferon (IFN). IFN was administered s.c. at a site distant to the tumour at $1.8 \times 10^5$ IU $day^{-1} \times 35$ days. Drugs were given i.p. weekly ×5 at 20% MTD (CP 1.4 mg $kg^{-1}$). Using median tumour doubling times, the activity of each agent and combination was expressed in terms of growth delay extrapolated from control. In addition all groups were analysed in terms of % increase in tumour volume over 35 days. The adenocarcinoma had a control doubling time of 11 days and gave growth delays of 0.36 with CP, 0.64 with CTX and 0.91 with IFN alone. In combination CP+IFN and CTX+IFN gave growth delays of 3.18 and 1.27 doubling times respectively. This difference was highly significant ($P<0.01$) with a similar result when the results were based on % increase in tumour volume over 35 days. For squamous and adenosquamous tumours, responses were less dramatic but IFN+CP was the most active, giving growth delays of 1.74 (squamous) and 1.56

(adenocarcinoma) 0.84 and 0.44 CP alone, and <0.2 and 0.44 IFN alone in squamous and adenosquamous tumours respectively. In view of this highly significant potentiation of CP by IFN against adenocarcinoma we are now evaluating other IFNs to optimise the selection of conditions for a clinical trial.

**Does human recombinant interferon alpha potentiate the anti-proliferative effect of tamoxifen towards oestrogen receptor positive human breast cancer cells?**

H.W. van den Berg[1] & R. Clarke[2]

*Departments of [1]Therapeutics and [2]Biochemistry, The Queen's University of Belfast, UK.*

It has been reported that patients receiving interferon (IFN) for the treatment of breast cancer showed increased levels of oestrogen receptor (ER) in skin metastases after therapy (Pouillart *et al.* (1982), *Eur. J. Cancer Clin. Oncol.* **18**, 929). Since response to tamoxifen is largely dependent on the presence of ER we reasoned that IFN might potentiate the activity of the antioestrogen. The ER positive human breast cancer cell line ZR-75-1 has been used as a model to test this hypothesis. We have found that the sensitivity of cell monolayers to human recombinant IFN-$\alpha$ is markedly dependent on initial cell seeding density. At low density IFN-$\alpha$ (500 and $10^3$ IU ml$^{-1}$) caused a 30–50% reduction in proliferative capacity after 6 days continuous treatment, whilst no effect was observed if cells were initially plated at higher density. Pre-treatment of cells at low density for 3 days with IFN-$\alpha$ (500 or $10^3$ IU ml$^{-1}$) followed by a continuous 6 day treatment with IFN and tamoxifen, ($10^{-8}$–$10^{-6}$ M), resulted in effects on cell proliferation which were additive rather than synergistic although some potentiation of the antiproliferative effect of tamoxifen, ($10^{-8}$ and $10^{-6}$ M), by 500 IU ml$^{-1}$ IFN was observed 3 days after cotreatment.

In contrast to the antiproliferative effect of IFN alone, $^3$H thymidine incorporation into DNA was markedly inhibited at IFN concentrations as low as 10 IU ml$^{-1}$. We are currently investigating the possibility that the effect of IFN on ER levels may be similarly dissociated from effects on cell proliferation.

**A model system for investigating the factors involved in tumour cell sensitivity to chemotherapeutic drugs**

M.C. Walker, J.R.M. Masters & C.N. Parris

*Department of Histopathology, Institute of Urology, St Paul's Hospital, 24 Endell Street, London WC2, UK.*

Chemotherapy has improved the prognosis of patients with certain tumours (e.g. testicular germ cell tumours), but has made little impact on the survival of most patients with 'solid' tumours. It is not known why certain histological types of tumour are particularly sensitive to chemotherapeutic drugs.

In order to study this question, the *in vitro* drug sensitivities of continuous cell lines derived from five human testicular germ cell tumours (833K, Tera II, SuSa, NEC-8, $T_3B_1$) and five transitional cell carcinomas of the bladder (RT4, RT112, T24, HT1197, HT1376) were compared. Inhibition of colony formation by continuous exposure to a range of concentrations of cisplatin and adriamycin was determined for each cell line. The testicular cell lines were on average five times more sensitive to these drugs than the bladder cell lines, using the ID70s (dose required to inhibit colony formation by 70%) as a basis for comparison.

While host and pharmacological factors are known to influence the anti-tumour activity of chemotherapeutic drugs, these data indicate that inherent differences in sensitivity between tumours of different histological types of primary importance. Furthermore, these differences in chemosensitivity are retained in long-term culture. These cell lines provide a model system with which the biochemical basis for drug sensitivity may be investigated, an approach which could lead to more effective treatment for all tumours.

**The response to ionizing radiation and cytotoxic drugs of human small cell lung cancer spheroids – growth delay and cell survival endpoints**

T.T. Kwok & P.R. Twentyman

*MRC Clinical Oncology and Radiotherapeutics Unit, Hills Road, Cambridge, UK.*

The response of 250 $\mu$m diameter multicellular spheroids of a human small cell lung carcinoma cell line (POC) to a variety of cytotoxic drugs and X-rays was measured in terms of growth delay (GD) and cell surviving fraction (SF). SF was measured immediately (0 h) and 24 h after acute X-irradiation or a 1 h exposure to adriamycin (ADM), nitrogen mustard (HN2), vincristine (VCR) and CCNU. GD was measured as the difference in time for treated and control spheroids to double their initial diameter. SF (24 h) was the same as SF (0 h) for X-rays, VCR and CCNU indicating an absence of recovery from potentially lethal damage (PLD). In HN2 treated spheroids a small amount of PLD recovery was seen, whereas, for ADM, increased cell killing was observed between 0 h and 24 h. Using the formula:

$$\mathrm{Td} = \frac{\log 2 \times \mathrm{GD}}{-\log \mathrm{SF}}$$

(Twentyman (1980), *Br. J. Cancer,* **41,** 297), the doubling times (Tds) for surviving clonogenic cells in CCNU, VCR and X-rays treated POC spheroids lie in the range 50–55 h. For HN2, calculated Tds are 52 h for SF (0 h) and 69 h for SF (24 h) while for ADM, Tds are 49 h for SF (0 h) and 91 h for SF (24 h). The doubling time of untreated POC cells during exponential growth is 42 h. The relationships between response endpoints clearly differ between agents and also differ in a number of aspects from those which we have previously reported for EMT6 mouse tumour spheroids (Twentyman, 1980). Recovery from potentially lethal damage in other human small cell lung cancer lines is currently under investigation.

### The incorporation of $^{75}$Se and [$^{3}$H]TdR by renal carcinoma cells exposed to cytotoxic agents – possible test for drug sensitivity

D. Heinemann, A.V. Kaisary, P.J.B. Smith & M.O. Symes

*Departments of Surgery and Urology, University of Bristol and Bristol and Weston District Health Authority, UK.*

Tumour tissue from 12 patients with renal cell carcinoma was disaggregated using collagenase and DNase and a pure tumour cell suspension was separated on a Nycodenz column (Umpleby *et al.* (1984), *Br. J. Surg.* **71,** 659). The tumour cells were cultured in TCM 199+10% FCS to which were added depoprovera, mitomycin C, methotrexate, vinblastine or vincristine. After 24–72 h the cultures were washed free of drug and pulsed with either 1 $\mu$Ci ml$^{-1}$ of $^{75}$Se in methionine free MEM or (in 4 cases) 2.5 $\mu$Ci ml$^{-1}$ of [$^{3}$H]TdR in TCM 199. Isotope incorporation by the tumour cells was measured after a further 24–72 h culture and the results compared with those for cells not previously exposed to a drug. The drug to which the tumour cells were most sensitive was that producing the greatest reduction in $^{75}$Se incorporation. On this basis 3 tumours were most sensitive to depoprovera, 3 to mitomycin C, 2 to methotrexate, 1 to vinblastine and 1 to vincristine. In 4 cases sensitivity as indicated by [$^{3}$H]TdR incorporation concurred with that predicted by $^{75}$Se. One tumour was well differentiated and a second had been embolised via the renal artery prior to nephrectomy. The cells of these 2 tumours did not incorporate $^{75}$Se. One tumour, sensitive to vinblastine, was established in tissue culture, and its cells showed that $^{75}$Se incorporation was dependent on the dose of drug to which they had been exposed.

### Improvement in plating efficiency of lung tumours by clonogenic assay – Implications for drug sensitivity testing

A.P. Simmonds[1], H. Kerr[1], K.G. Davidson[2] & A. Faichney[2]

[1]*Cell Laboratory, Department of Biochemistry; and* [2]*Cardiothoracic Surgical Unit, Royal Infirmary, Glasgow, UK.*

Following an earlier study (Simmonds *et al.* (1983), *Br. J. Cancer,* **48,** 119) in which significant drug results were obtained in only 10/26 successful cultures due to low PE, we attempted growth enhancement with the use of REL medium (Sheridan and Simmons (1981), *J. Cell Physiol.* **107,** 85) HITES and HITES+FBS (Carney *et al.,* (1981), *PNAS* **78,** 3185). Fifteen samples of NSCCL were set up at $2\times10^{5}$ viable cells/plate in 0.3% agar in these 3 media; a further 9 were set up in REL and HITES+FBS. Underlayers of medium in 0.5% agar were used for the HITES system. Colony scoring of triplicate plates was at 12 days following incubation at 37°C in 5% $CO_2$/air. In REL medium, growth was 100% with PE 0.026–1.9% (23/24>0.05%); in HITES+FBS growth was 100% with PE 0.019–0.2% (18/24>0.05%); and in HITES alone growth was 100% with PE 0.008–0.15% (3/15>0.05%). REL medium was therefore used in subsequent drug sensitivity tests where $2\times10^{5}$ cells/plate were exposed for 1 h to 10% peak plasma concentrations of vindesine and *cis*-platinum. Colony counts in drug treated plates were expressed as percentage survival of control and recorded as sensitive if this was <50%. 40/45 specimens tested yielded significant (PE>0.015%) results for the 2 drugs. 20/40 (50%) were S to *cis*-platinum and 22/24 (55%) S to vindesine. 17/40 (43%) were S to both drugs and 13/40 (33%) were R to both drugs. This high yield of drug results (c. 89%) ensures the usefulness of this assay as a laboratory test and medium using rat erythrocyte lysate (REL) is now used routinely for lung tumour culture in this laboratory.

### A comparison of a soft agar clonogenic assay and an isotope uptake assay to assess cell kill after both drug and radiation treatments using both a high cloning line (CHO) and a low cloning human tumour xenograft line (V7)

J.A. Hanson, A.E. Bean & J.L. Moore

*Radiation Sciences Laboratories, Velindre Hospital, Cardiff.*

Along with other centres using the *in vitro* soft agar assay of Courtenay & Mills, (1978), (*Br. J. Cancer,* **37,** 261) for *in vitro* drug testing of patient tumour cells, we have encountered problems that include the length of time required for cloning, often up to 6 weeks, and the difficulties of preparing a good single cell suspension (Agrez *et al.* (1982), *Br. J. Cancer*, **46**, 880). The tritiated thymidine uptake assay developed by Friedman & Glaubiger (1982) (*Cancer Res.* **42**, 4683) can be completed within a week and is not dependent on single cells. Essentially the technique involves culturing non adherent cells for 4 days before giving a 24 h pulse of tritiated thymidine.

We have directly compared these 2 assays both on the high cloning CHO-K1 line (CE ~0.6 and ~12 h doubling time) and a low cloning human ovarian tumour xenograft line V7 (CE ~0.04 and ~60 h doubling time). Time course studies to determine the pattern of isotope uptake over the first week in culture showed an increasing rate of uptake in 2 of 4 xenografts tested. Linearity between incorporated label (c.p.m.) and cell number was also obtained. *Cis*-platin, melphalan and vinblastine were tested with the V7 line using 1 h *in vitro* exposures and survival in both assays was in good agreement over the first two decades of cell kill. Similar agreement was found after radiation exposures except that the survival, as measured by the isotope uptake assay, showed increasing resistance once the 0.01 level was reached. Survival data obtained at daily intervals during the first 6 days in culture show that survival falls to the clonogenic survival after 4 days with little change from day 4 to 6.

### Culture and drug sensitivity of human breast carcinoma: Effect of hormone and RBC supplements

A.P. Simmonds[1], J. McKinlay[1] & C.S. McArdle[2]

[1]*Cell Laboratory, Biochemistry Department and* [2]*Department of Surgery, Royal Infirmary, Glasgow, UK.*

We have cultured breast cancer cells by clonogenic assay in agarose using hormone supplemented DHEM (HD) (Calvo *et al.* (1983), *Br. J. Cancer*, **48**, 683) both alone and with rat rbc added at 1% of final volume (HDR). Thirteen tumour samples were disaggregated with collagenase/DNase and suspended at $10^5$ viable cells in 1 ml HD/0.3% agarose over 0.5% agarose underlayers containing either HD or HDR. Incubation was at 37°C for 12 days in 5% $CO_2$/air and plates were stained with INT violet prior to microscopic counting of colonies. All samples but one, a well-differentiated adenocarcinoma, grew with plating efficiencies (PE) ranging from 0.2–0.63% (majority in range 0.1–0.3%). With rbc (HDR) enhancement of PE occurred up to 12 fold with the majority of samples enhanced 1.6–6.9. However, one ductal carcinoma had decreased PE but the adenocarcinoma which failed to grow in HD grew well in HDR. HDR was used for drug studies, with a 1 h incubation at 10% peak plasma concentration of MTX, 5FU, adriamycin and mitomycin C prior to plating. Sensitivity was deemed to be <50% survival as a function of control. Of samples available for test, 4/11 (36%) were sensitive to 5FU and 2/12 (17%) sensitive to MTX. Seven out of twelve patients were resistant to all drugs tested, 1 being additionally resistant to adriamycin and mitomycin C and only 1 was sensitive. Clinical correlations will be made for 3 patients. Further samples will be evaluated for drug sensitivity using both media, as it has not been established wherher the change in PE affects such measurements.

### Azido analogues of lipophilic antitumour 2,4-diaminopyrimidine DHFR inhibitors

R.J. Griffin, M.F.G. Stevens & E. Bliss

*Cancer Research Campaign Experimental Chemotherapy Group, Department of Pharmaceutical Sciences, University of Aston, Birmingham B4 7ET, UK.*

The aromatic azido group is the forgotten substituent in antitumour drug design although it has three noteworthy features: (i) it can replace key halogen substituents (e.g. chloro) because its lipophilic and electronic characteristics are comparable; (ii) it can act as a pro-drug modification of the corresponding aryl amine; (iii) it can degrade photolytically, thermally and (possibly) biologically to reactive nitrene species with covalent bonding potential. A series of azido analogues of the lipophilic drug pyrimethamine (1) has been prepared (see Table). The m-azido

**Table** Activity of azidophenylpyrimidines against rat liver DHFR

| | *R* | $R_1$ | $R_2$ | $ID_{50}(M)$ | Ki(*M*) |
|---|---|---|---|---|---|
| 1 | Cl | H | Et | $1.8\times10^{-6}$ | $2.6\times10^{-9}$ |
| 2 | Cl | $N_3$ | Et | $1.3\times10^{-6}$ | $1.6\times10^{-9}$[a] |
| 3 | Cl | $N_3$ | Me | $3.2\times10^{-6}$ | $2.6\times10^{-9}$ |
| 4 | OMe | $N_3$ | Et | $6.6\times10^{-7}$ | $1.7\times10^{-9}$ |
| 5 | OEt | $N_3$ | Et | $1.6\times10^{-6}$ | $1.7\times10^{-9}$ |
| 6 | $N_3$ | Cl | Et | $3.4\times10^{-7}$ | $3.8\times10^{-10}$ |
| 7 | $N_3$ | Cl | Me | $1.0\times10^{-6}$ | $8.2\times10^{-10}$ |

[a]Also Ki $2.4\times10^{-9}$ against L1210 DHFR.

compounds (2–5) are equi-inhibitory against rat liver DHFR but less active than the p-azido analogues (6 and 7).

The ethanesulphonic acid salt (MZPES) of *m*-azidopyrimethamine (2) has been selected for clinical tests.

### Dipyridamole induced inhibition of thymidine incorporation *in vivo* and potentiation of CB3717 cytotoxicity *in vitro*

D.R. Newell, P.M. O'Connor, J. Bishop, A.H. Calvert & K.R. Harrap

*Department of Biochemistry and Pharmacology, Institute of Cancer Research, Sutton, Surrey, UK.*

Dipyridamole (DP) is a non-specific inhibitor of thymidine (TdR) transport into cultured cells. Extracellular TdR is an important determinant of cytotoxicity for $N^{10}$-propargyl-5,8-dideazafolic acid (CB3737), an inhibitor of *de novo* thymidylate synthesis (Jackman *et al.* (1984), *Biochem. Pharmacol.* **33**, 3269). We have (a) determined the ability of DP to inhibit TdR salvage in bone marrow, gastrointestinal tract epithilium (GIT) and Walker 256 tumour cells *in vivo* and (b) investigated the cytotoxicity of CB3717/DP combinations *in vitro*. DP was given by i.v. infusion to rats, and tissues removed 2 h after an i.v. bolus of [$^3$H]-TdR. As shown in the Table, TdR incorporation into normal tissue DNA was DP sensitive, whilst incorporation into Walker cells was relatively insensitive.

**Table**

| *DP dose ($mg\,kg^{-1}$)* | *DP plasma level ($\mu M$)* | *[$^2H$]TdR incorporation (% control $\bar{x}$ ± s.d. n=4–5)* Tumour | Bone marrow | GIT |
|---|---|---|---|---|
| 40 | 13±2 | 88±31 | 32±3 | 35±14 |
| 100 | 52±13 | 118±37 | 20±3 | 24±6 |
| 200 | 125±32 | 43±23 | 19±5 | 28±6 |

*In vitro* DP (10 $\mu$M) largely prevented the TdR (10 $\mu$M) reversal of CB3717 cytotoxicity in L1210 cells (83% inhibition of cell growth) but not in Walker cells (14% inhibition of cell growth). The results with Walker cells *in vitro* and *in vivo* are consistent with the observation that, unlike the L1210, Walker cells have a transport-inhibitor resistant TdR uptake mechanism (Belt (1983), *Mol. Pharmacol.* **24**, 479). Incorporation of TdR into bone marrow and GIT is ~80% transport-inhibitor sensitive, a value similar to that reported for the L1210. Thus as salvage of TdR may limit the effectiveness of CB3717 *in vivo*, combination with DP should enhance the activity of CB3717 against transport-inhibitor sensitive tumours.

### Transport studies with [$^3$H]-CB3717

A.L. Jackman, B. Grezelakowska-Sztabert, D.R. Newell, A.H. Calvert & K.R. Harrap

*Department of Biochemical Pharmacology, Institute of Cancer Research, Sutton, Surrey, UK.*

$N^{10}$-propargyl-5,8-dideazafolic acid (CB3717) is a tight-binding inhibitor of thymidylate synthetase (TS) (Ki-4 nM). Inhibition of this enzyme is reponsible for the anti-tumour properties of this drug both *in vitro* and *in vivo*. However, CB3717 has a high dose requirement for tumour cell growth inhibition even in the absence of salvageable TdR. The $ID_{50}$ for CB3717 in culture (continuous exposure for 48 h) is 5 $\mu$M. We therefore studied the transport characteristics of $^3$H CB3717 into L1210 cells. Uptake of a toxic dose of $^3$H CB3717 (50 $\mu$M) was linear (~0.09 pmol min$^{-1}$ 10$^{-6}$ cells) and by 24 h the intracellular level exceeded that of the medium but because of avid intracellular protein-binding this may not represent concentration of free drug. In the presence of thymidine, however, the intracellular level had plateaued at equilibrium levels by 24 h but dilution by cell division was a complicating factor. When these cells were resuspended in drug-free medium (+TdR) 35% of the drug was effluxed within 4 h after which the drug concentration reduced proportionately to the number of cell divisions implying that efflux did not occur. The incorporation of [$^3$H]UdR into acid-precipitable material remained completely inhibited in the absence of extracellular drug (>24 h). It seems probable that a non-effluxable pool of CB3717 is formed within the cell (possibly as poly-glutamates) that is able to maintain the inhibition of TS for long periods. As the kinetics for CB3717 were not saturable (linear up to 100 $\mu$M) a Kt value could not be obtained, even in 160 mM HEPES buffer. However, the addition of a 14-fold excess of either folic or folinic acid did not affect [$^3$H]-CB3717 uptake suggesting that CB3717 is not transported via the mechanisms operative for these compounds.

### Effects of CB3717 on radiolabelled nucleoside incorporation by human epithelial A549 cells

A.C. Simpson & A.L. Harris

*Cancer Research Unit, Royal Victoria Infirmary, Newcastle upon Tyne NE1 4LP, UK.*

The antimetabolite CB3717, a quinazoline antifolate, is a competitive inhibitor of the binding of 5–10 methylene tetrahydrofolate to thymidylate synthetase (TS), the rate limiting enzyme in the *de novo* synthesis of thymidylate (Jones *et al.* (1981), *Eur. J. Cancer,* **17,** 11). This drug is active against methotrexate resistant L1210 leukaemia cells and is presently undergoing Phase II trials. The effect of CB3717 on epithelial and lymphoid tumour cell lines *in vitro* was studied, both alone and in combination with other drugs. The *in vitro* measurement of [methyl-$^3$H] thymidine (*TdR) and 6-[$^3$H] deoxyuridine (*dUR) by human epithelial and lymphoid cell lines was used to indirectly measure the effect of CB3717 on nucleic acid synthesis and repair. Epithelial cells were harvested at 50% confluence and incubated for 72 h in concentrations of CB3717 from 100 nM to 200 $\mu$M. The ID 50 was 2.3 $\mu$M. *TdR uptake was stimulated after 3 h exposure. A decrease in *dUR uptake was observed at concentrations above 10 $\mu$m, at lower concentration *dU uptake was stimulated. 5FU and MTX, which also indirectly affect TS, inhibited *dU uptake and stimulated *TdR but did not stimulate *dU uptake. The time course of the effect with CB3717 was much slower than that of 5FU or MTX. In a human lymphoid cell line CB3717 inhibited *dU uptake but did not stimulate *TdR uptake. Epithelial cell confluence markedly affected response to CB3717 and uptake of *TdR and *dU was only slightly altered in cells near confluence. Incorporation of *5FU was also stimulated by CB3717. These results show a marked difference in response of epithelial compared with lymphoid cell lines and suggest that whilst CB3717 is an inhibitor of TS, it may have other effects on nucleoside transport.

### *In vitro* comparisons between monoclonal antibody-targeted drug and toxin conjugates

M.J. Embleton[1], V.S. Byers[2], M.C. Garnett[1] & R.W. Baldwin[1]

[1]*Cancer Research Campaign Laboratory, University of Nottingham, Nottingham NG7 2RD, UK; and* [2]*The XOMA Corporation, Berkeley, California 94710, USA.*

Monoclonal antibody 791T/36, raised against human osteogenic sarcoma cell line 791T, has been conjugated to several cytotoxic drugs and toxins in order to derive selectively active reagents. The conjugates which proved to be most active were prepared using methotrexate (MTX) attached by means of an albumin carrier (Garnett *et al.* (1983), *Int. J. Cancer,* **31,** 661), and ricin A-chain (RTA), respectively. The cytotoxic action of these conjugates was compared on cell lines bearing different concentrations of 791T/36-defined antigen, the most antigenic (791T) bearing $10^6$ antibody-binding sites per cell.

In clonogenic assays the best MTX conjugate achieved an $IC_{50}$ against 791T of $10^{-9}$M in terms of MTX content. For RTA conjugates the $IC_{50}$ was as low as $5\times10^{-11}$M. However, when tested against poorly antigenic cells the MTX conjugate showed greater selectivity. For example, T24 bladder carcinoma cells which have only $10^4$ 791T/36-binding sites per cell, were $\sim$100 times less sensitive to the MTX conjugate than 791T but the difference in sensitivity of the two lines to RTA conjugate was 10–15 fold.

It appears that toxin conjugates have an advantage over drug conjugates in terms of cytotoxic activity, but this may be offset by poorer discrimination in situations where antigenic differences are quantitative rather than qualitative.

### Endocytosis of monoclonal antibody 791T/36 and methotrexate-HSA-791T/36 conjugate by an osteogenic sarcoma cell line

M.C. Garnett & R.W. Baldwin

*Cancer Research Campaign Laboratories, University of Nottingham, University Park, Nottingham NG7 2RD, UK.*

The preparation and properties of a methotrexate-human serum albumin-antibody conjugate which retains both antibody binding activity and complete drug cytotoxicity has been previously reported (Garnett *et al.* (1983), *Int. J. Cancer,* **31,** 661). It has been proposed that drug conjugates would be taken up into cells and the drug released by lyosomal enzymes (De Duve *et al.* (1974), *Biochem. Pharmacol.* **23,** 2495). We have already demonstrated a reduction in cytotoxicity of conjugate by inhibitors of lysosomal enzymes and wished to find direct evidence for the endocytosis of antibody and conjugate in our osteogenic sarcoma cell line model system.

This problem has been tackled using 3 different fluorescent probes. First, methotrexate-HSA-791T/36 conjugate. Cells were saturated with conjugate at 0°C, washed and incubated at 37°C for varying time periods. Conjugate remaining on the cell surface was then quantitated using a fluorescence activated cell sorter to measure bound fluoresceinated rabbit anti HSA. Second, fluoresceinated 791T/36. Upon endocytosis fluorescein is quenched due to a reduction in pH. This fluorescence can be unmasked by agents which perturb lysosomal pH, e.g. monensin. Both of these methods show endocytosis of up to 65% over a 3 h period.

Finally the distribution of fluorescence was investigated microscopically using fluoresceinated HSA conjugated to 791T/36. This probe shows a change from an even distribution at 0°C to a perinuclear distribution after 3 h incubation at 37°C, confirming the quantitative results obtained with the other two probes.

### Effect of linkage variation on pharmacokinetics of ricin A chain antibody conjugates

A.J. Cumber, N.R. Worrell, G.D. Parnell, J.A. Forrester & W.C.J. Ross

*Chester Beatty Laboratories, Institute of Cancer Research, Fulham Road, London SW3 6JB, UK.*

Conjugates of ricin A chain with tumour specific antibodies have been proposed as cancer chemotherapeutic agents. Linkage of ricin A chain to antibody by a bridge containing a disulphide bond generally results in a conjugate with specific cytotoxicity to antigen bearing cells *in vitro* but only limited *in vivo* efficacy. It has been suggested that this may be due to lability *in vivo* of the disulphide bond. We have investigated the pharmacokinetics of three antibody-ricin A chain conjugates with linkages containing a disulphide bond, a sterically hindered disulphide bond and a non-reducible sulphide bond. An ELISA assay was used to measure blood levels of each conjugate at time points up to 48 h following i.v. administration. All 3 conjugates appeared to obey two-compartment kinetics and $\alpha$ and $\beta$ phase half-lives were calculated using a curve fitting routine. The results are shown in the Table.

The results suggest that lability of the linkage affects the $\beta$ phase half-life while the half-life for the $\alpha$ phase, when rapid disappearance of the intact conjugate from the blood occurs, is the same for the 3 conjugates studied.

**Table**

| | $\alpha t_{\frac{1}{2}} \pm$ s.d. | $\beta t_{\frac{1}{2}} \pm$ s.d. |
|---|---|---|
| Ricin A disulphide conjugate | 0.76±0.14 | 9.72±0.58 |
| Ricin A hindered disulphide conjugate | 0.76±0.09 | 14.40±1.42[a] |
| Ricin A sulphide conjugate | 1.12±0.73 | 21.50±5.04[a] |

[a] $P<0.05$ with respect to the disulphide conjugate by Student's $t$-test.

### Heat potentiation of melphalan: Are drug transport mechanisms involved?

D.J. Honess & N.M. Bleehen

*MRC Unit and University Department of Clinical Oncology and Radiotherapeutics, Hills Road, Cambridge, CB2 2QH, UK.*

We have previously shown that the potentiation of melphalan (Mel) toxicity in C3H mice by systemic hyperthermia (Hx) at 41°C is greater in RIF-1 and KHT tumours than in marrow stem cells (CFUs) resulting in therapeutic gain; also that this is not accounted for by the higher Mel concentrations in Hx tumours. Mel is known to be transported into tumour cells *in vitro* by 2 separate amino acid carriers, one of which (system L) is reportedly absent in CFUs *in vitro. In vitro,* leucine (leu) competitively inhibits both carriers and BCH (a leu analogue) specifically inhibits system L. We have used these inhibitors in mice to confirm the *in vitro* findings at 37°C and to investigate their effect on heat potentiation of Mel, using doses non-toxic to tumour or marrow: 1 mmol kg$^{-1}$ (a) and 4 mmol kg$^{-1}$ (b) leu and 1 mmol kg$^{-1}$ BCH. Mel toxicity to CFUs was unaffected by BCH at 37 or 41°C, confirming the absence of system L in CFUs *in vivo,* but was reduced by leu at 37°C. Results for RIF-1 tumour treated with 15 mg kg$^{-1}$ Mel, without (Cont) and with inhibitors are shown on the left for 37°C (unshaded) and 41°C (shaded) (see Figure). Mel toxicity (assayed by clonogenic cell survival 24 h after treatment) was reduced by leu and BCH very similarly at 37 and 41°C, suggesting that carrier activities are largely unchanged at 41°C. Data for KHT tumour assayed by growth delay support these data. We conclude that effects on intracellular transport are probably not involved in Hx potentiation of Mel, and do not account for the greater potentiation in tumour than marrow. See Figure.

Figure

### Misonidazole protects against oral CCNU

F.Y.F. Lee & P. Workman

*MRC Clinical Oncology and Radiotherapeutics Unit, Hills Road, Cambridge, UK.*

Nitroimidazoles such as misonidazole (MISO) are able to enhance the response of experimental tumours to certain cytotoxic agents. This 'chemosensitization' is normally greater than in normal tissues, resulting in an improved therapeutic index. In previous studies where both agents were injected i.p. in the mouse we have shown that $500\,mg\,kg^{-1}$ MISO reduces the plasma clearance of CCNU, leading to a preferential increase in nitrosourea concentration in the KHT tumour; these pharmacokinetic changes thus explained the improved therapeutic index obtained for the combination (Lee & Workman (1983), *Br. J. Cancer*, **47**, 659). We have now carried out similar experiments but giving the CCNU orally, with quite different results. MISO reduced the antitumour activity (growth delay endpoint) of oral CCNU by a dose-modifying factor (DMF) of 0.59–0.71, and likewise increased the acute LD50 by a DMF of 0.74. Thus MISO protected both tumour and normal tissues from the toxicity of oral CCNU, the net effect being no change (or possibly a slight decrease) in therapeutic index. Nitrosourea pharmacokinetics were studied by HPLC. Concentration of parent drug were considerably increased by MISO, thus demonstrating inhibition of CCNU metabolism by the nitroimidazole. For example, peak plasma concentrations were increased 6.7-fold from 0.46 to $3.1\,\mu g\,ml^{-1}$. In contrast, due to decreased hydroxylated metabolite levels, peak total nitrosoureas were diminished by 1.5- and 1.7-fold in plasma and tumour respectively. Values of $AUC_{0-\infty}$ for plasma and tumour were unchanged by MISO, but AUC values for concentrations $>1\text{–}2\,\mu g\,ml^{-1}$ were markedly reduced. In conclusion, inhibition of CCNU hydroxylation, leading to reduced nitrosourea exposure, is responsible for the protective effect of MISO against the toxicity of oral CCNU in mouse tumour and normal tissue.

### Mechanistic studies on the cytotoxic and radiosensitizing properties of RSU 1069

I.J. Stratford, A.R.J. Silver, J.M. Walling & G.E. Adams

*MRC Radiobiology Unit, Harwell, Oxfordshire OX11 0RD, UK.*

RSU 1069 has a similar one-electron reduction potential to that of misonidazole but is 10× more efficient as a radiosensitizer *in vitro* and *in vivo*. RSU 1069 differs from misonidazole in that it contains a monofunctional alkylating group (aziridine) in the side chain. We have indirect evidence that RSU 1069 interacts with intracellular DNA, e.g. 3 hours exposure of V79 cells to 50 μm RSU 1069 in $N_2$ reduces survival to $10^{-2}$ for unlabelled cells and $10^{-3}$ for cells labelled with 5-BUdR. In contrast, control and labelled cells show the same sensitivity to misonidazole. Further, the enzyme poly(ADP-ribose) synthetase, which can be involved in excision repair of DNA/mono-adducts can be inhibited with 3-aminobenzamide (3-AB). Treatment of cells with 3-AB substantially increases the toxicity of RSU 1069 in air, with a dose modification factor of ~2. 3-AB has no affect on the cytotoxicity of misonidazole. However, the drug-DNA interaction that would be inferred from the above contributes only slightly to the enhanced radiosensitizing efficiency of RSU 1069. We have shown this by treating cells with minimally-toxic concentrations of RSU 1069. These concentrations would normally radiosensitize hypoxic cells but if, instead, cells are washed free of unbound drug immediately before irradiation in $N_2$ then very little sensitization is observed. Thus it is likely that the molecular properties of RSU 1069 that confer high sensitizing efficiency may not be those that are related to its toxicity.

### The effects of combinations of benznidazole and methyl CCNU on transplantable adenocarcinomas of the mouse colon

J.A. Double & M.C. Bibby

*Clinical Oncology Unit, University of Bradford, Bradford BD7 1DP, West Yorkshire, UK.*

There is considerable interest in the combined use of nitroimidazoles and cytotoxic therapy. Twentyman & Workman (1983) (*Br. J. Cancer*, **48**, 17) suggested that a combination of CCNU with benznidazole (BENZO) may have significant clinical potential. The present study investigates the effect of BENZO together with methyl CCNU on three transplantable adenocarcinomata of the mouse colon (MAC). The MAC series is generally poorly responsive to cytotoxic therapy with responses seen only close to maximum tolerated dose. Methyl CCNU is the most active agent tested to date. MAC 13 is poorly differentiated, MAC 26, well differentiated and MAC 15A, an ascitic variant of the solid MAC 15. BENZO alone produced no significant anti-tumour effects on any of the tumour lines. Treatment of MAC 13 and MAC 15A with BENZO ($78\,mg\,kg^{-1}$) 30 min prior to maximum tolerated dose cytotoxic therapy resulted in increased toxicity with no enhancement of anti-tumour activity.

Pretreatment with BENZO failed to improve the anti-tumour response of MAC 26 to methyl CCNU at a range of dose levels even though there was a considerable increase in toxicity. These results differ from those of earlier workers using CCNU and it is possible that the differences may be explained by the pharmacokinetic properties of the two agents and by the different chemosensitivities of the tumour systems employed.

The results to date seem to indicate no potential clinical gain from the present combination.

**Growth-inhibitory effects of a 5-aza-2′-deoxycytidine (5AZAdCYD) in human leukaemic cells and solid-tumour-derived cells**

A. Leyva, L.C.M. Boeije, E.I.M. Meijne & H.M. Pinedo

*Department of Oncology, Free University Hospital, De Boelelaan 1117, 1081 HV Amsterdam, The Netherlands.*

5AzadCyd is a potent antileukaemic agent with a mechanism of action which may involve induction of differentiation due to suppression of DNA methylation. The aim of the present study was to determine whether 5azadCyd was active against solid tumours. Seven human cell lines representing 4 solid-tumor types (3 squamous cell carcinoma, 2 melanoma, 1 breast cancer and 1 colon cancer) were examined for sensitivity to 5azadCyd *in vitro*. Comparison was made with the human leukaemic cell lines HL60 and CEM. Cells were maintained in exponential growth as suspension (leukaemic cells) or as monolayer (solid-tumor-derived cells) cultures. Cells were exposed for 3 or 24 h to drug concentrations of 0.01 to 100 $\mu$M and cell growth was determined daily for up to 4 days. Growth-inhibition kinetics varied for the 9 cell lines but consistently showed a delayed drug effect of 24–48 h, even at high drug concentrations. With 24 h drug exposure, inhibition of growth was eventually >80% in the 2 leukaemic cell lines and 5 solid-tumour-derived cell lines at 1 $\mu$M 5azadCyd. Comparing 3 h and 24 h drug exposures, leukaemic cells were similarly sensitive. Solid-tumour-derived cells were several fold less sensitive using 3 h exposure, with the exception of MCF-7 breast cancer cells which were inhibited by >50% at 10 $\mu$M 5azadCyd. Using either drug exposure period, resistance was observed in 1 squamous cell carcinoma and 1 melanoma. The data suggest that 5azadCyd has a spectrum of activity which is not restricted to leukaemias. Prolonged drug exposure may be required to best exploit the cytotoxic effects of 5azadCyd, particularly in solid tumours. In addition, preliminary studies show that the amount of 5azadCyd triphosphate formed correlates with drug sensitivity.

**Differential DNA damage induced by 5-aza-2′-deoxyazacytidine (Aza-dC) and 5,6-dihydroazacytidine (DHAC) in mouse L1210 and in two human lymphoblastoid cell lines**

M. D'Incalci[1], J.M. Covey[2], D.S. Zaharko[2], E. Erba[1] & K.W. Kohn[2]

[1]*Mario Negri Institute, 20157 Milan, Italy; and* [2]*DTP Division of Cancer Treatment, NCI, NIH, Bethesda, MD 20205, USA.*

Aza-dC is a compound with activity against mouse and human leukaemias. Its mode of action is unknown, but cytotoxicity has been related to its ability to inhibit DNA-cytosine methyltransferase when the analogue is incorporated into DNA. DHAC is a more stable analogue which is much less potent as an inhibitor of DNA methylation and as a cytotoxic agent. Using the alkaline elution technique, we have comparatively evaluated the DNA damage produced by the 2 drugs in mouse L1210 cells, in a lymphoblastoid cell line derived from a patient with xeroderma pigmentosum (XP) (GM2345A), and in a lymphoblastoid cell line derived from a clinically normal subject (GM3714). When cells were treated with Aza-dC (0.1–10 $\mu g\,ml^{-1}$) or DHAC (1–100 $\mu g\,ml^{-1}$) and simultaneously labelled with $^{14}$C-thymidine for 24 h, a dose-dependent increase in the elution rate of $^{14}$C-DNA was observed, whereas no significant effect was observed when same treatment was performed on $^{14}$C prelabelled cells. This suggests each drug must be incorporated in DNA to produce DNA damage. The elution rate of DNA after Aza-dC treatment was much greater at pH 12.6 than at pH 12.1 and the elution curves were convex, suggesting the presence of DNA-alkali-labile sites (ALS), possibly due to base-free sites in DNA. In contrast DHAC produced DNA single-strand breaks (SSB) with a linear elution rate, not pH-dependent. Aza-dC induced ALS persisted for 48 h after drug treatment, and were only moderately repaired at 72 h. SSB induced by DHAC were almost completely repaired 24 h after drug washout. Neither Aza-dC nor DHAC caused greater damage in XP cells compared to the normal human line. Flow cytometry studies showed that Aza-dC increased the % of cells in $G_2$ whereas DHAC did not. The differing biological activities of Aza-dC and DHAC may be related to the contrasting nature and persistence of DNA damage caused by these agents.

### Potentiation of *cis*-platin and melphalan by methyl methane-sulphonate

J.M. Walling & I.J. Stratford

*MRC Radiobiology Unit, Harwell, Didcot, Oxford, UK.*

The monofunctional alkylating agent MNNG potentiates cell killing by bifunctional alkylators operating at the same position in DNA ($0^6$ guanine). It is proposed that this occurs via saturation of the suicide enzyme $0^6$ methyl-transferase. We report here the ability of methyl-methanesulphonate (MMS), which alkylates extensively at the $N^7$ position of guanine but not detectably at the $0^6$ position, to potentiate *cis*-platin and melphalan. These are bifunctional agents which initially alkylate at $N^7$.

When V79 cells are given 1 mM MMS for 1 h in air at 37°C prior to a range of doses of *cis*-platin, their sensitivity to this drug is greatly increased. At a surviving fraction (SF) of 0.5 the dose modification factor (DMF) is 6.00. At lower SFs the DMF increases; SF 0.1 DMF = 2.93, SF 0.01 DMF = 2.41. This effect on the shoulder of the dose response curve may indicate an inhibition of DNA repair. A similar treatment with MMS also increases the cytotoxicity of melphalan SF = 0.1 DMF = 1.73; SF = 0.01 DMF = 1.73. The potentiation is dependent on drug scheduling. If MMS is given before *cis*-platin, potentiation is only obtained at doses below 15 $\mu$m *cis*-platin, above this protection results. Isobologram analysis shows that MMS is synergistic with *cis*-platin at doses of MMS approaching 1 mM lower doses produce antagonism.

In contrast a dose of MNNG ($0^6$ alkylator) equitoxic with 1 mM MMS, i.e. 0.01 mM MNNG (SF = 0.35) does not potentiate either *cis*-platin or melphalan.

We are currently investigating the rate and extent of DNA platination and crosslinking following MMS pretreatments in order to determine the mechanism of potentiation.

### DNA damage and cytotoxicity produced by 1,5,2,4-dioxadithiepene-2,2,4,4-tetraoxide (cyclic SOSO)

N.W. Gibson, K.W. Kohn & J.A. Hartley

*Laboratory of Molecular Pharmacology, National Cancer Institute, Bethesda, Maryland 20205, USA.*

1,5,2,4-dioxadithiepene-2,2,4,4-tetraoxide (Cyclic SoSo) was found to be active against the L1210 and P388 leukaemias, M5076 sarcoma, and the human MX-1 mammary tumour xenograft in the NCI screening programme. As a result this compound is due to enter Phase I clinical trials in USA. Cyclic SoSo structurally resembles two other classes of antitumour agents; the haloethylsulfonates and the dimethanesulphonic acid esters. Both of these classes of compounds are thought to exert their antitumour activity through crosslinking of DNA.

We have studied the effects of Cyclic SoSo on DNA in IMR-90 and VA-13 human embryo cells by means of DNA alkaline elution analysis. In contrast to a number of alkylating agents Cyclic SoSo produced no DNA-DNA interstrand crosslinks in either cell line, even at concentrations which produced a greater than a 3 log cell kill. At equimolar concentrations Cyclic SoSo induced DNA-protein crosslinks in both cell lines to a similar extent. Frank DNA breaks and alkali-labile DNA strand breaks were detected in both cell lines. These breaks did not appear to be related to a DNA topoisomerase II activity. A greater quantity of strand breaks appeared in the IMR-90 than the VA-13 cell line after exposure to Cyclic SoSo. The IMR-90 cell line, however, was less sensitive to the cytotoxic effects of Cyclic SoSo than was the VA-13 cell line.

### A comparison of the DNA base adducts formed after alkylation with two novel chloroethylating agents

J.A. Hartley, J. Strong, K.W. Kohn & N.W. Gibson

*Laboratory of Molecular Pharmacology, National Cancer Institute, Bethesda, Maryland 20205, USA.*

A major cytotoxic reaction of chloroethylating agents with DNA appears to involve alkylation of the guanine-06 position. We have studied 2 novel chloroethylating agents: 8-carbamoyl-3-(2-chloroethyl)imidazo |5, 1-d|-1, 2, 3, 5-tetrazin-4(3H)-one (mitozolomide) and 2-chloroethyl (methylsulfonyl)-methanesulfonate (C1EtSoSo) and found that they cause effects attributable to chloroethylation of the $0^6$-position of guanine in DNA. We have now analysed the DNA adducts produced by C1EtSoSo and mitozolomide. The two compounds, $^{14}C$-ethyl labelled, were reacted with calf thymus DNA for 4 h at 37°C pH7. After precipitation with ethanol, the reacted DNA was depurinated by 0.15 N HCl for 60 min at 100°C followed by neutralisation with 0.3 N NaOH. The resulting hydrolysate was analysed by reverse phase high pressure liquid chromatography (HPLC). Five major radioactive peaks were found on analysis of the mitozolomide treated DNA eluting at 4, 9, 16, 23 and 27 min respectively. Only 3 major peaks were found on analysis of the C1EtSoSo treated DNA, eluting at 4, 9 and 27 min respectively. The 27 min peak has been identified as 7-chloroethylguanine by HPLC comparison of the synthesised standard, and was confirmed by mass spectral analysis. Preliminary analysis of the synthesised 7-hydroxyethylguanine and $0^6$-hydroxyethylguanine standards indicates that they correspond to the 16 and 23 min peaks respectively. Thus unlike mitozolomide, C1EtSoSo does not appear to hydroxyethylate guanine in calf thymus DNA. The biological implications of these results are currently being investigated.

### Structure-activity relationships in antitumour 3-alkylimidazo-tetrazinones

S.P. Langdon[1], D. Chubb[1], L. Vickers[1], R. Stone[1], M.F.G. Stevens[1], G.U. Baig[1], N.W. Gibson[1], J.A. Hickman[1], E. Lunt[2], C.G. Newton[2], P.J. Warren[2] & C. Smith[2]

[1]*CRC Experimental Chemotherapy group, University of Aston, Birmingham B4 7ET; and* [2]*May & Baker Ltd., Dagenham, Essex RM10 7XS, UK.*

Mitozolomide (1) has potent broad spectrum antitumour activity against murine screens and is currently undergoing Phase II trials in man. In other series of N-alkyl antitumour agents – triazenes, nitrosoureas, hydrazines, melamines and formamides – the nature of the N-alkyl substituent has a critical bearing on activity. Several N-alkyl congeners of mitozolomide were screened for antitumour activity against the TLX5 lymphoma ($2 \times 10^5$ cells injected s.c. into groups of 5 female CBA/ca mice). Results are shown in the Table. In the present series the 3-methyl analogue of mitozolomide has anti tumour activity, the 3-(2-bromethyl)-derivative has marginal activity and other 3-alkyl analogues are all inactive against this tumour on a single dose schedule.

**Table**

| R | *Optimum dose*[a] *(mg kg$^{-1}$)* | *T/C × 100%* |
|---|---|---|
| 1 $(CH_2)_2Cl$ | 40 | 5/5 cures[b] |
| 2 Me | 160 | 157 |
| 3 Et | 640 | 123[c] |
| 4 *n*-Pr | 320 | 103 |
| 5 $(CH_2)_2OMe$ | 80 | 103 |
| 6 $CH_2CH{=}CH_2$ | 80 | 103 |
| 7 $(CH_2)_2Br$ | 160 | 137 |
| 8 $(CH_2)_3Cl$ | 320 | 108 |
| 9 $CH_2CH(Cl)CH_2Cl$ | 160 | 105 |
| 10 Benzyl | 20 | 107 |

[a]Drugs administered day 3 post implantation. [b]Day 60. [c]T/C < 125% is considered inactive.

### Activity of mitozolomide (NSC 353451) against human tumour xenografts

S. Aamdal, Ø. Fodstad & A. Pihl

*Norsk Hydro's Institute for Cancer Research, Montebello, Oslo 3, Norway.*

The effect of mitozolomide, a new imidazotetrazine (Horgan *et al.* (1983), *Br. J. Cancer* **48,** 132; Stevens *et al.* (1984), *J. Med. Chem.* **27,** 196), on xenografted human tumours was studied in 3 different assay systems. In concentrations of 1–500 $\mu g\, ml^{-1}$, mitozolomide inhibited the colony forming ability in soft agar of cell suspensions from 5 sarcomas, 5 melanomas, 5 lung and 2 colon cancers, and a mammary carcinoma. Four sensitive tumours of different histological types were also tested in the 6-day subrenal capsule assay in conventional mice and as s.c. growing tumours in nude mice. In the melanoma, the effect of mitozolomide was similar to that of CCNU. Mitozolomide was far more effective than ADM and VP16 in a small cell lung cancer and clearly superior to ADM and abrin in an osteogenic sarcoma. In fact, the tumour size measurements and histological examinations indicated that nude mice carrying these tumours were cured by the mitozolomide treatment. In the colon cancer, mitozolomide showed a significant, but less pronounced effect than that of 5-FU. The same ranking order was obtained when the effects of the different drugs in the 2 *in vivo* assays were compared, supporting the validity of the subrenal capsule assay. Phase II trials of mitozolomide in malignant melanoma, soft tissue sarcoma and small cell lung cancer have recently been initiated.

### Tissue disposition of radiolabelled mitozolomide in mice

P. Kestell[1], C. Brindley[2], P. Antoniw[2], J.A. Slack[1] & E. Newlands[2]

[1]*CRC Experimental Chemotherapy Research Group, University of Aston, Birmingham; and* [2]*Department of Medical Oncology, Charing Cross Hospital, London, UK.*

Mitozolomide [8-carbamoyl-3-(2-chloroethyl)imidazo-[5,1-d]-1,2,3,5-tetrazin-4(H)-one], a new antitumour agent, has recently undergone Phase I clinical evaluation. As part of pre-clinical studies the disposition of this compound in BALB/c mice following a single i.p. dose at 20 mg kg$^{-1}$ was determined in plasma, heart, lung, spleen, kidneys, stomach, small intestine, large intestine, liver, eyes, fat, muscle, brain and residual carcass, using [$^{14}$C]-mitozolomide labelled either in the imidazole ring or in the chloroethyl side chain. The concentration of non-radiolabelled mitozolomide after its i.p. administration at 20 mg kg$^{-1}$ was also determined in plasma, liver, brain, kidney, muscle, lung and spleen of BALB/c mice. After 1 h following administration, radioactivity was detected in all the tissues and plasma and the concentrations of radioactivity in plasma, liver, brain, kidney, muscle, lung and spleen (4.3–13.0 μg equivalents g$^{-1}$) were virtually identical to unchanged drug (5.5–13.1 μg equivalents g$^{-1}$). However much higher concentrations of radioactivity were found in the livers (36.4 μg equivalents g$^{-1}$) and kidneys (22.3 μg equivalents g$^{-1}$) of those mice which had received [$^{14}$C-chloroethyl]-mitozolomide. The elimination of non-radiolabelled mitozolomide from tissues exhibited first order kinetics with an apparent half-life of <1 h. Over an 8 h period following administration, the concentration of radioactivity associated with either of the radiolabelled compounds decreased in most tissues but the decline did not mirror that of plasma. However, in liver, kidney, lung, spleen and heart of mice treated with [$^{14}$C-imidazole]-mitozolomide, the radioactivity concentration increased or remained constant during the same period. After 48 h, the concentrations of radioactivity had significantly decreased but were still measurable.

### Plasma and tissue disposition of mitozolomide (NSC 353451) in tumour-bearing mice

C.J. Brindley, P. Antoniw & E.S. Newlands

*Department of Medical Oncology, Charing Cross Hospital, London W6 8RF, UK.*

Mitozolomide (M) is a novel and potent anti-tumour agent with significant activity against a wide range of murine tumours. We investigated the distribution of (M) in the plasma and tissues of mice bearing the ROS osteosarcoma by HPLC. Peak plasma and tissue concentrations were reached within 30 min and drug disposition appeared to fit a first-order, one-compartment kinetic model with an elimination half life of <1 h . The disposition of (M) in tumour-bearing mice also followed a first-order process but in this case elimination of drug was significantly faster from plasma, liver, lung and kidney tissue compared to the elimination half life of drug in the same tissues of mice without tumour ($P<0.05$). (M) was well distributed to all tissues including brain and tumour.

Workman and Lee (1984) (*Br. J. Cancer*, **50**, 251) have shown that phenobarbitone pre-treatment reduces the antitumour activity of (M) against the KHT mouse sarcoma. We found that AUC values calculated from (M) concentration versus time profiles in plasma and tissues were significantly lower in mice pretreated with phenobarbitone compared to those values obtained for mice receiving saline alone ($P<0.005$).

It has been proposed that (M) chemically degrades to the chloroethyl triazine, MCTIC (Horgan & Tisdale (1984), *Biochem. Pharmac.* **33,** 2185). However, the above observations suggest that metabolism may be involved in the decomposition of (M).

### Pharmacokinetic factors affecting comparative tumour response to CCNU and mitozolamide

P. Workman, F.Y.F. Lee & J. Donaldson

*MRC Clinical Oncology and Radiotherapeutics Unit, Hills Road, Cambridge, UK.*

The novel antitumour agent mitozolamide exhibits a similar spectrum of experimental antitumour activity to that of the nitrosourea CCNU, suggesting, as do biochemical studies, a common mechanism of action. The two agents also have identical potencies and therapeutic indices in mice. However, since we have shown that the clinical efficacy of CCNU is limited by ineffective plasma and tumour concentrations, mitozolamide might possess an advantage in terms of improved pharmacokinetics. For this reason we have used HPLC to compare the mouse plasma, tumour and brain pharmacokinetics of mitozolamide and CCNU, both given i.p. at a dose of 20 mg kg$^{-1}$. With mitozolamide this produced a peak plasma concentration of 19 μg ml$^{-1}$ at 10 min, compared to the much lower peak total nitrosourea concentration of 5.5 μg ml$^{-1}$ at 10–45 min after CCNU. The plasma elimination $t\frac{1}{2}$ was also shorter for mitozolamide (58 min) compared to that for total nitrosoureas (94 min), but the plasma AUC was greater for mitozolamide at 1349 compared to 502 μg ml$^{-1}$ min. In the KHT tumour the peak mitozolamide concentration of 16 μg g$^{-1}$ was seen at 15–30 min and the levels remained closely similar to those in plasma from then on. For total nitrosoureas after CCNU, the peak tumour concentration was much lower (4 μg ml$^{-1}$), with concentrations at later times again being similar to plasma. In brain the peak mitozolamide concentration of 8 μg g$^{-1}$ occurred at 15–30 min, and brain/plasma ratios were constant at 70% from then on. With CCNU brain concentrations were similar to or higher than those in plasma. The clinical efficacy of mitozolamide is likely to require the achievement in man of plasma and tumour concentrations of a similar order to those described above.

### CCRG 81045 – An antitumour imidazotetrazinone with potential as a clinical alternative to DTIC

S.P. Langdon[1], M.F.G. Stevens[1], R. Stone[1], N.W. Gibson[1], G.U. Baig[1], J.A. Hickman[1], C.G. Newton[2] & E. Lunt[2]

[1]*CRC Experimental Chemotherapy Group, University of Aston, Birmingham B4 7ET; and* [2]*May and Baker Ltd., Dagenham, Essex, UK.*

CCRG 81045 (M&B 39831; NSC 362856) (1:R=Me) is the 3-methyl analogue of the antitumour drug mitozolomide (1: R=$CH_2CH_2Cl$) and decomposes in aqueous media to afford MTIC (2: R=H) the presumed active metabolite of DTIC (2: R=Me).

(1)

It has been suggested that DTIC may be poorly metabolized to MTIC by patients. Unlike DTIC, CCRG 81045 does not require metabolic activation and is being developed for clinical trial as a chemically activated prodrug of MTIC. The activity of CCRG 81045 against a panel of murine tumours *in vivo* (see Table) is superior to that of DTIC. (Goldin *et al.* (1981), *Eur. J. Cancer*, **17**, 129).

**Table** Antitumour activity of CCRG 81045

| *Tumour* | *Site of implantation* | *Schedule (days)* | *Optimum dose* ($mg\,kg^{-1}$ *i.p.*) | $T/C$ ($\times$ 100%) |
|---|---|---|---|---|
| P388 | i.p. | 1–5 | 200 | 254[a] |
| L1210 | i.p. | 1–9 | 100 | 193[a] |
| TLX5 | s.c. | 3–7 | 40 | 181[b] |
| M5076 | i.m. | 1–17 | 20 | 0[c] |
| ADJ/PC6A | i.m. | 14 | 40 | 0[c] |

[a]Median survival time. [b]Mean survival time. [c]Mean tumour volume index.

### Potentiation of the action of DTIC by inhibitors of poly (ADP-ribose) polymerase

J.M. Lunn[1], C. Pierpoint[2], B.T. Golding[2] & A.L. Harris[1]

[1]*Cancer Research Unit and* [2]*Department of Organic Chemistry, University of Newcastle-upon-Tyne, Newcastle-upon-Tyne NE1 4LP, UK.*

The drug 5-(3,3-dimethyl-1-triazeno)imidazole-4-carboxamide (DTIC) is metabolised by humans to the related monomethyl compound (MTIC), whose subsequent breakdown gives rise to short-lived reactive species which methylate cellular DNA.

To obviate the need for this activation step, MTIC has been synthesized and its effects on cultured A549 (lung carcinoma derived) cells examined. Exposure to MTIC was found to cause a dose-dependent inhibition of cell growth. The effects observed were similar to those found with equimolar concentrations of the methylating agent methyl nitrosourea (MNU). Substitution of 5-amino-1-($\beta$-D-ribofuranosyl)-imidazole-4-carboxamide (AICAR), the riboside of a degradation product of MTIC, for MTIC produced no effect on cellular proliferation, indicating that cytotoxicity was not due to perturbation of purine biosynthesis. Further MTIC which had been allowed to stand in aqueous solution for 1 h before application had no effect on cellular proliferation. DNA strand breaks were detected by an alkaline unwinding technique, and were found to increase linearly with increasing concentrations of MTIC. Formation of poly (ADP-ribose) seems to be involved in the repair of DNA damage produced by MTIC because inclusion of 3-acetamidobenzamide (3AAB), an inhibitor of poly (ADP-ribose) synthetase, in the growth medium enhanced the cytotoxicity of MTIC ($LD_{50}$ in the absence or presence of 3AAB was 200 $\mu$m or 50 $\mu$m respectively). Cellular NAD levels dropped after exposure to MTIC concentrations in excess of 1 mM.

Our findings suggest that it may be possible to extend the limited clinical usefulness of DTIC by concurrently suppressing the synthesis of poly (ADP-ribose).

### Induction of haemoglobin synthesis in K562 cells by N-methyl but not N-ethyl compounds

M.J. Tisdale

*CRC Experimental Chemotherapy Group, Department of Pharmaceutical Sciences, University of Aston, Birmingham B4 7ET, UK.*

We have been investigating the structure-activity relationships of a group of imidazotetrazinones, the 2-chloroethyl analogue of which (mitozolomide) is currently undergoing a clinical trial. Among the other members of the series the methyl analogue (8-carbamoyl-3-methyl-imidazo- [5,1-d]-1,2,3,5-tetrazin-4-(3H)-one, CCRG 81045) showed potent anti-tumour activity, while the ethyl analogue was inactive, in analogy with the dialkyltriazenes. When tested against the K562 human erythroleukaemia cell line both a monomethyltriazene and CCRG 81045 were effective in the induction of haemoglobin production, a marker of red cell differentiation, while the corresponding ethyl homologues were inactive in the modification of gene expression in this cell line, even at concentrations which have an equivalent effect on cell growth. Growth inhibition alone seems to be insufficient for the induction of haemoglobin synthesis since other members of the series inhibited growth without substantially increasing the number of haemoglobin producing cells. Differentiation appeared not to arise by selective toxicity to the original non-differentiated cell compartment followed by clonal expansion of the differentiated cells. Treatment with CCRG 81045 caused a lower level of enzymatic methylation of newly synthesized DNA which coincided with the increased gene transcription.

### Receptors for epidermal growth factor are present on human bladder tumours

D.E. Neal[1,3], C. Marsh[2], M.K. Bennett[2], P.D. Abel[1], R.J. Sainsbury[3], R.R. Hall[1] & A.L. Harris[4]

*Departments of [1]Urology and [2]Pathology, Freeman Hospital, Freeman Rd, High Heaton, Newcastle-upon-Tyne NE7 7DN; Departments of [3]Surgery and [4]Clinical Oncology University of Newcastle-upon-Tyne, UK.*

Epidermal growth factor (EGF) is a small polypeptide that stimulates cell growth and division. It is found in high concentration in urine. Close similarities have been found between EGF receptors (EGF-r) and contain oncogene proteins.

Sixty patients have been studies to determine whether EGF-r can be detected on transitional cell carcinoma (TCC). Twenty-four patients with superficial TCC (pTA 15, pT1 9) and 24 with invasive TCC (pT3) have been compared with 12 controls. Frozen sections were studied histologically by an indirect immunoperoxidase technique with a monoclonal antibody to EGF-r.

Twenty-one of the 24 invasive TCC and 7 of the 24 superficial TCC were positive for EGF-r ($x^2 = 14.9$; $P < 0.001$). Significantly more of the poorly differentiated tumours were positive for EGF-r (18 of 21) compared with moderately differentiated tumours (10 of 27; $x^2 = 9.6$; $P < 0.01$). Normal urothelium was not positive for EGF-r.

EGF receptors have been identified on human bladder tumours. They were observed more frequently on invasive tumours than on superficial tumours, suggesting that expression of the EGF-r is associated with a capacity for invasion.

### Fibroblast cell lines derived from human breast cancers have epidermal growth factor receptors and produce an epidermal growth factor like molecule

J.R.C. Sainsbury, E.A. Hirst, G.K. Needham, J.R. Farndon & A.L. Harris

*Departments of Surgery and Oncology, University of Newcastle-upon-Tyne, UK.*

We have demonstrated the presence of high affinity EGF receptors on human beast cancers by radioligand and monoclonal techniques. It is known that the stroma in which epithelial cells grow is important and differences in the type of fibroblasts are found in breast cancer specimens and normal patients.

We derived fibroblast lines from 19 breast cancers and examined them for EGFr by radioligand binding. Sixteen (82%) bound from 0.15 to 2.2 fmol $^{125}$-I-EGF mg$^{-1}$ protein. The binding was of a lower affinity than that found on the breast cancers ($1.2 \times 10^{-8}$ *vs* $1.9 \times 10^{-9}$).

Fibroblast cultures binding no EGF arose from EGFr positive tumours whilst the high binding cultures (>1.5 fmol mg$^{-1}$) came from EGFr −ve tumours. The conditioned media from the fibroblast cultures was examined for the presence of epidermal growth factor like molecules by either interference with a radioligand assay after concentration on a Sep-pak or by immunoprecipitation by a urogastrone-EGF antibody of $^{35}$-S-methionine labelled cells and resolution on a SDS/PAGE system. No interference of binding could be found on cells grown in EMEM media but after oestrogen stimulation with $10^{-7}$ DES identifiable bands of ~6000 daltons were seen. These results indicate that the EGFr on human breast cancer cells may be being stimulated by EGF like factors derived from the surrounding fibroblasts.

### Epidermal growth factor and oestrogen receptors in human breast cancers – Is there a link?

E.A. Hirst, J.R.C. Sainsbury & A.L. Harris

*Departments of Surgery and Cancer Research, University of Newcastle-upon-Tyne, UK.*

Epidermal growth factor (EGF) and oestrogen receptor (ER) status of both breast tumour biopsies and established human breast cancer cell lines (MCF 7, 4 sources, MDA-MB-231) have previously been shown to be inversely correlated (Sainsbury *et al.* (1983), *Br. J. Cancer*, **53**, 235).

The exception to this finding was for the T 47D cell line which showed both types of receptor to be present at lower numbers. During recent experiments investigating EGF binding to T47D cells it was noted that the amount of $^{125}$-I-EGF bound $\mu g^{-1}$ protein increased with passage number.

T47D cells (a gift from Dr O'Hare, Ludwig Inst.) were routinely maintained in DMEM +10% FBS and subcultured weekly. EGF binding was carried out according to the method of Osborne *et al.* (1982) (*J. Clin. Endocrinol. Metab.* **55**, 86) on ice for 2 h. ER was measured by a dextran coated charcoal method. Cells were examined between passage numbers 25 and 45. A 3 to 4 fold increase in EGF binding was seen over this time.

A fall in ER number was found as EGFr binding increased. This observation provides further evidence that EGFr and ER are linked. Further work is needed to study the implication of this finding and to see if the action of anti-oestrogen drugs causes further changes.

### EGF receptors in non small cell lung cancer

D. Veale[1], C. Marsh[2], T. Ashcroft[2] & A.L. Harris[1]

*[1]Clinical Oncology, Newcastle General Hospital, Newcastle-upon-Tyne NE4 6BE; and [2]Thoracic Medicine and Pathology, Freeman Hospital, Newcastle-upon-Tyne NE7 7DN, UK.*

The presence of epidermal growth factor receptors on tumour cells has been found to be related to the degree of invasion of bladder tumours and breast cancer.

Fourteen non small cell lung cancers (NSCLC) have been studied for the presence of epidermal growth factor (EGF) and its receptor (EGFr). A monoclonal antibody to the receptor and a polyclonal antibody to EGF were used in an immunoperoxidase technique. EGFr has been assayed on membrane preparations by radioligand binding with $I^{125}$ EGF and Scatchard analysis. The slides were graded by 2 independent observers, 0, +, ++, +++, for positive staining. Squamous carcinoma stained for EGF showed 2/8+++, 5/8++, 1/8+, 2 adenocarcinomas stained ++, and +, and 2 large cell carcinomas stained ++ for EGF.

Staining for EGFr was graded +++ 5 of 8 squamous carcinomas; and 2/8 were ++, 1/8 +. The adenocarcinomas were ++ for receptor and one large cell stained +++ for receptor and one was +.

Multipoint radioligand assay for EGF receptor showed high affinity binding sites with Kd of $1\text{–}2 \times 10^{-9}$ M. There were also low affinity sites.

Thus we have demonstrated the presence of EGFr on each type of NSCLC and we plan to study the association of the presence of these receptors with subsequent outcome and response to therapy.

### A sensitive enzymic assay for O$^6$-methylguanine in DNA

T.A. Meyer[1], D.P. Cooper[1], J. Brennand[2] & G.P. Margison[1]

*Departments of [1]Carcinogenesis and [2]Biochemical Genetics, Paterson Laboratories, Christie Hospital, Manchester M20 9BX, UK.*

A plasmid has been isolated from an *E. coli* genomic DNA expression library which increases the amount of O$^6$-methylguanine (O$^6$-meG) methyltransferase (MT) in the host bacteria. Extracts of such bacteria have been used to determine the amounts of O$^6$-meG in DNA methylated *in vitro* or *in vivo* in a simple competition assay which measures the inhibition of transfer of label from [$^3$H]-methylated DNA to protein. The lower limit of detection of O$^6$-meG by this method is less than 10 fmol and using *in vitro* methylated DNA there was a good correlation (coefficient 0.999) between this and conventional liquid chromatographic methods. Addition of different amounts of non-methylated DNA to the assay had no detectable effect. Rat liver DNA methylated to different extents *in vivo* by administration of dimethylnitrosamine was found to contain levels of O$^6$-meG generally closely similar ($\pm$7%) to those found by conventional methods. The technique used to measure the levels of O$^6$-meG in rat liver DNA following administration of different doses of dimethylnitrosamine: after lower doses, disproportionally lower levels of O$^6$-meG were detected indicating a more efficient repair of this product.

**Effect of X-rays on rat hepatic $O^6$-methylguanine methyltransferase activity**

B. Hoey[1], J. Butler[1] & G.P. Margison[2]

*Departments of [1]Radiation Chemistry and [2]Carcinogenesis, Paterson Laboratories, Christie Hospital, Manchester, M20 9BX, UK.*

Studies on the relationships between alkylation damage in DNA and carcinogenesis have shown that the promutagenic lesion $O^6$-methylguanine ($O^6$-meG) can be subject to a repair process which results in the transfer of the methyl group from the $O^6$-position to a cysteine residue which is probably within the methyltransferase (MT) protein itself. In rat liver, the activity of the enzyme can be increased by pretreatment of the animals with a variety of hepatotoxic agents or by partial hepatectomy. In order to explore further the response, we have examined the effects of whole body X-irradiation on $O^6$-meG MT activity using a rapid *in vitro* assay. Forty-eight hours after 1 Krad of electrons from the linear accelerator, there was a 3-fold increase in MT levels in liver extracts. Similar effects have been produced in various strains of mice, a species in which enhanced $O^6$-meG MT activity has not previously been reported. Initial examinations of the possible mechanism of the response has shown that $O^6$-meG MT can be inhibited *in vitro* by X-irradiated DNA. However, this appears to be due to denaturation rather than to the presence of modified bases.

**Isolation of an *E. coli* gene coding for a repair protein containing both $O^6$-methylguanine and methylphosphotriester methyltransferases**

G.P. Margison[1], D.P. Cooper[1] & J. Brennand[2]

*Departments of [1]Carcinogenesis and [2]Biochemical Genetics, Paterson Laboratories, Christie Hospital, Manchester M20 9BX, UK.*

The repair of methylphosphotriesters (MP) and $O^6$-methylguanine ($O^6$-meG) produced in DNA by reaction with methylating agents has been shown to occur in *E. coli* by transfer of the methyl groups to protein. Using a rapid and sensitive assay which can measure total MT activity we have identified and isolated from an *E. coli* genomic DNA library a plasmid which codes for both $O^6$-meG and MP MT activity. These have been shown by *in vitro* assays and by fluorography of proteins labelled by incubation of bacterial extracts with [$^3$H]-methylated DNA and subjected to polyacrylamide gel electrophoresis (PAGE) to be part of a single protein molecule of mol. wt. $\sim$37Kd. In various other strains of *E. coli*, the plasmid causes production of varying amount of 37, 18 and 13Kmd MT proteins. A partial restriction endonuclease map of the DNA has been produced and subclone plasmids have been characterised. Different plasmids increased the production of either $O^6$-meG MT or MP MT which had mol. wts. of $\sim$18 and 13 Kd respectively, although additional bands were seen on fluorograph in the former case. *In vitro* DNA-directed protein synthesis using [$^{35}$S]-methionine or cysteine followed by PAGE-fluorography showed that these plasmids contained the structural genes for the MT.

**Examination of mammalian DNA and mRNA for sequences homologous to the *E. coli* $O^6$-methylguanine-methylphosphotriester methyl-transferase gene**

P.M. Potter[1], D.P. Cooper[1], J. Brennand[2] & G.P. Margison[1]

*Departments of [1]Carcinogenesis and [2]Biochemical Genetics, Paterson Laboratories, Christie Hospital, Manchester M20 9BX, UK.*

Using a functional assay to screen an *E. coli* DNA library carried in an expression vector we have identified a plasmid carrying a section of DNA which codes for $O^6$-methylguanine and methyl-phosphotriester methyltransferases. A subfragment of this, which codes for both activities has been isolated by agarose gel electrophoresis and labelled by nick translation using [$^{32}$P]dCTP. Poly $A^+$ messenger RNA was isolated by oligo dT-cellulose chromatography from total RNA extracted from the liver of rats in which $O^6$-methylguanine methyl-transferase activity had been increased by pre-treatment with 2-acetylaminofluorene. This, and Hind-III digested human spleen DNA was subjected to agarose gel electrophoresis and transferred to nitrocellulose before hybridisation with the labelled probe. Under the conditions used no indications of any sequence homology have so far been found indicating that the sequences are not highly conserved.

**Analysis of c-Ha-*ras*-1 proto-oncogene activation induced by carcinogen-modification and by depurination**

D.H. Phillips[1], K.H. Vousden[1], H. Bos[2] & C.J. Marshall[1]

*[1]Chester Beatty Laboratories, Institute of Cancer Research, London SW3 6JB; and [2]Department of Medical Biochemistry, University of Leiden, The Netherlands.*

The mechanism by which *ras* proto-oncogenes become activated in many human and animal tumours has been shown to be through a single point mutation resulting in the alteration of an amino acid in the p21 protein product. We have shown that *in vitro* modification of pEC, a plasmid containing the normal cellular c-Has-*ras*-1 proto-oncogene, by covalent reaction with (±)*anti*-benzo[a]pyrene-7,8-diol-9,10-oxide (*anti*-BPDE), causes mutations that activate the gene when the plasmid is transfected into NIH 3T3 mouse cells (*Nature* **310,** 586, 1984). DNA from each of 5 transformed foci were found by restriction endonuclease analysis to contain a point mutation in the 12th codon (GGC). DNA from a further 10 foci have now been found by hybridization with 20mer oligonucleotide probes to contain a point mutation in the 61st codon (CAG). Of these, 4 of the alterations are in the 1st nucleotide, 3 are in the 2nd and 3 are in the 3rd. Experiments to determine the exact nature of these point mutations are in progress. Modification of pEC with *N*-acetoxy-2-acetylaminofluorene, resulting in 1 adduct per 140 nucleotides, produced transformed foci, as did modification with 1′-acetoxysafrole, at a level of 1 adduct per 30 nucleotides. Heating pEC at low pH also produced transformed foci, providing evidence that depurination can be mutagenic in mammalian cells.

### Isolation of DNA repair mutants of CHO cells

C.N. Robson, A.L. Harris & I.D. Hickson

*Cancer Research Unit, University of Newcastle-upon-Tyne, Royal Victoria Infirmary, Newcastle-upon-Tyne NE1 4LP, UK.*

A number of DNA repair mutants of CHO-K1 cells have been isolated by the 'toothpicking' method of replica-plating. These cells were isolated on the basis of their sensitivity to the anticancer drugs mitomycin-C or bleomycin, and were subsequently found to differ in their cross-sensitivity to other DNA damaging agents, such as EMS, cis-Pt and u.v. light. One mutant, designated MMC-2, exhibits a 6-fold hypersensitivity to mitomycin-C as well as 10-fold hypersensitivity to u.v. light. Five other mutants also show approximately this level of sensitivity to mitomycin-C but are resistant to u.v. light. One of these mutants (BLM-2) is also hypersensitive to bleomycin. One further mutant (BLM-1) exhibits sensitivity to bleomycin only. Since their cross-sensitivity to DNA damaging agents differs so markedly, it would appear that they represent several different complementation groups.

A human gene bank of ~300,000 recombinants has been constructed in the cosmid vector pNNL. This DNA is currently being introduced into the repair mutants by polycation transfection selecting for resistant to mitomycin-C, u.v. or bleomycin.

### Interaction of N-nitrosoglycocholic acid with DNA *in vitro*

D.E.G. Shuker[1] & S.R. Tannenbaum[2]

[1]*MRC Toxicology Unit, MRC Laboratories, Woodmansterne Road, Carshalton, Surrey, SM5 4EF, UK and* [2]*Department of Nutrition and Food Science, MIT, Cambridge, MA 02139, USA.*

The N-nitrosation of endogenous amides in gastric contents has been postulated as a source of carcinogenic N-nitrosamides which may have a role in the induction of gastric cancer.

N-nitrosoglycocholic acid (NOGC), (Shuker *et al.* (1981), *J. Org. Chem.* **46,** 2092) is a mutagenic and carcinogenic derivative of the naturally occurring bile acid conjugate, glycocholic acid (GC). By analogy with structurally simpler N-nitrosamides NOGC would be expected to act as an alkylating agent giving rise to carboxymethylated ($-CH_2CO_2H$) adducts.

[$^{14}$C]-NOGC (with label in the glycine carboxyl group) was synthesised by nitrosation of the commercially available [$^{14}$C]-GC. Overnight incubation of [$^{14}$C]-NOGC with calf thymus DNA at pH 7.2 and 37°C resulted in radioactively labelled DNA. Most of the activity (>75%) was removed on heating the DNA at 100°C at pH 7.4 for 30 min ('neutral thermal hydrolysis') which is known to remove labile alkylated purines. After precipitation of the depurinated DNA, HPLC of the supernatant on reversed phase and aminopropyl columns showed that some some of the radioactivity co-chromatographed with authentic carboxymethyl purines (N-7-carboxymethyl guanine and N-3-carboxymethyl adenine).

### RSU 1069 binding to DNA *in vitro* – evidence for binding to both the bases and the phosphate of DNA

A.R.J. Silver, P. O'Neill & T.C. Jenkins

*MRC Radiobiology Unit, Harwell, Didcot, Oxford, OX11 0RD, UK.*

|2-$^{14}$C|-RSU 1069 (1(2-nitro-1-imidazolyl)-3-aziridino-2-propanol), either as parent (unreduced) or following radiation reduction, binds to calf thymus DNA *in vitro*. Radiation reduced RSU 1069 binds to a greater extent and more rapidly than the parent compound. RSU 1137, a non-aziridino analogue of RSU 1069, only binds following radiation reduction. Radiation reduced misonidazole (1-(2-nitro-1-imidazolyl)-3-methyoxy-2-propanol) exhibits binding ratios a thousand-fold less than reduced RSU 1069. There is no evidence for binding of parent misonidazole.

Parent and reduced 1069 causes single strand breaks (ssbs) in pSV2 gpt plasmid DNA with the reduced compound causing a greater number of breaks at a given concentration. Parent and reduced RSU 1137 and misonidazole do not cause ssbs. It is inferred that the aziridine of parent and reduced RSU 1069 is required for ssb production.

The aziridine of RSU 1069 reacts with inorganic phosphate via nucleophilic ring-opening of the aziridine fragment. Incubation of plasmid DNA with reduced RSU 1069 in the presence of phosphate or deoxyribose-5-phosphate at concentrations greater than $0.5\,mol\,dm^{-3}$ prevents strand breakage, whereas the presence of $2\,mol\,dm^{-3}$ deoxyribose does not protect against strand breakage formation.

From these findings it is proposed that the observed binding to DNA occurs via the reduced nitro group and the aziridine of RSU 1069 and that these two have different target sites. Binding to DNA via the reduced nitro group may serve to increase aziridine attack due to localisation near its target. RSU 1069 binding to DNA is discussed in terms of both base and phosphate sites of attack.

**Tiazofurin and poly(ADP-ribose)synthetase in A549 cells**

M.R. Purnell, J.M. Lunn & A.L. Harris

*Cancer Research Unit, Royal Victoria Infirmary, Newcastle-upon-Tyne NE1 4LP, UK.*

Tiazofurin (TR) and selenotiazofurin are cytostatic nucleosides which act by inhibition of IMP dehydrogenase following their conversion to NAD analogues. Inhibition of growth of A549 human lung carcinoma cells ($IC_{50}$ after 4 days $20\,\mu M$ and $2\,\mu M$ respectively) can be largely prevented by the provision of exogenous guanosine. Nicotinamide (up to 10 mM) is largely ineffective. We investigated whether TR or its NAD analogue affected poly(ADP-ribose)synthetase, a nuclear enzyme utilizing NAD and inhibited by analogues of NAD nicotinamide. When data were corrected for intrinsic growth inhibition, TR increased the $IC_{50}$ of 3-acetamidobenzamide (AAB), an inhibitor of poly(ADP-ribose)synthetase: $10\,\mu M$ TR increased the $IC_{50}$ from 2.85 mM to 3.2 mM and $50\,\mu M$ TR increased it to 3.7 mM. Non-cytostatic concentrations of AAB (1 or 2 mM) enhance the toxicity of bleomycin ($IC_{50} = 118\,ng\,ml^{-1}$) by $\sim 50\%$. In contrast, $30\,\mu M$ TR had no synergistic effect on growth inhibition. Monofunctional alkylating agents such as NMU decrease cellular NAD by activating poly(ADP-ribose)synthetase. AAB at concentrations as low as $300\,\mu M$ can decrease the extent of the NAD drop mediated by 10 mM NMU. TR (up to $300\,\mu M$) had no significant effect on NAD levels alone or following NMU treatment.

We therefore conclude that neither tiazofurin nor its NAD analogue have any significant effect on poly(ADP-ribose)synthetase in these cells.

**The cytogenetic effects of razoxane (ICRF 159) and its structural analogues in cultured human lymphocytes**

R. Albanese

*ICI Pharmaceuticals Division, Alderley Park, Macclesfield, Cheshire SK10 4TG, UK.*

The antitumour agent razoxane and its structural analogues ICRF 202, 187 and 154 have been found to possess widely differing degrees of activity against experimental tumours. Their mode of action is unknown but in 1970 razoxane was shown to affect chromosome condensation in cultured human lymphocytes (CHL). The effect of these compounds on CHL chromosomes has been compared in the present study.

Lymphocytes were stimulated to divide in culture using phytohaemagglutinin. The compounds (each at two doses) or vehicle were added to the cultures 43 h later. This corresponds to the $G_2/M$ stage of the first cell cycle. Chromosome preparations were made at 48, 72 and 96 h after stimulation.

At 48 h, each compound (but not the vehicle control) induced abnormal chromosome condensation; the chromosomes were elongated and had the appearance of very early prophase-type chromosomes. At 72 and 96 h chromosome condensation appeared normal but there was a dose-dependent increase in both structural chromosome damage (breaks, fragments and chromosome exchanges) and polyploid cells; at 72 h a 25 fold increase with ICRF 202 ($0.5\,\mu g\,ml^{-1}$) a 5-fold increase with both 187 and 159 ($50\,\mu m\,ml^{-1}$) and a 4-fold increase with 154 ($50\,\mu g\,ml^{-1}$) compared to the vehicle control.

These results show razoxane and its structural analogues to be clastogenic *in vitro*.

**Faecal steroids and colo-rectal cancer: New markers for detecting high risk populations**

R.W. Owen[1], P.J. Henly[1], D. Day[2], M.H. Thompson[1] & M.J. Hill[1]

*PHLS, CAMR, BMRL, Salisbury, Wiltshire; and [2]Department of Pathology, University of Liverpool, Liverpool, UK.*

This study was designed to compare the faecal bile

**Table**

| *Group* | *LCA*[a] | *DCA*[a] | *CDCA*[a] | *CA*[a] | *FBA*[a] | *LCA/DCA* | *LCA/DCA × FBA* |
|---|---|---|---|---|---|---|---|
| Asian (70) | 1.11±0.11 | 1.99±0.22 | 0.41±0.09 | 0.55±0.17 | 4.08±0.34 | 0.76±0.08 | 2.42±0.20 |
| British (36) | 2.78±0.36 | 3.93±0.54 | — | — | 6.71±0.86 | 0.90±0.09 | 5.15±0.67 |
| Small adenoma (23)[b] | 2.76±0.54 | 5.49±1.32 | — | — | 8.25±1.65 | 0.87±0.17 | 5.66±1.30 |
| Large adenoma (34)[c] | 3.51±0.64 | 3.70±0.68 | — | — | 7.21±1.26 | 1.30±0.19 | 8.37±1.60 |
| CRC (34) | 4.01±0.63 | 3.39±0.59 | — | — | 7.40±1.12 | 1.91±0.33 | 11.23±2.30 |

[a]Results expressed in $mg\,g^{-1}$ dry faeces±s.e.; LCA, lithocholic acid; DCA, deoxycholic acid; CDCA, chenodeoxycholic acid; CA, cholic acid. [b]Adenoma diameter 0–0.4 cm. [c]Adenoma diameter 0.4–1.5 cm.

acid profiles of healthy British subjects and colo-rectal cancer (CRC) patients incorporating immigrant Asians (a very low CRC risk group) and an adenoma group (sub-divided into low and high risk groups). Faecal bile acids were analysed and data for the major free bile acids are shown in the Table.

In summary, the ratio of LCA to DCA and the LCA/DCA × FBA index are powerful indicators of CRC risk, and show a remarkable positive gradient from the very low risk group (Asians) through the intermediate risk groups to colo-rectal cancer.

### Faecal steroids and colorectal cancer: Allo bile acids

R. Wait, M.H. Thompson & M.J. Hill

*Bacterial Metabolism Research Laboratory, PHLS-CAMR, Porton Down, Salisbury, Wiltshire SP4 0JG, UK.*

Several bile acids have been shown to be tumour promoters in animal systems, while epidemiological studies have demonstrated an association between large bowel cancer (LBC) risk, elevated faecal bile acid concentration and carriage of clostridia able to dehydrogenate the steroid nucleus (NDH Clostridia). These observations have been explained by the bacterial production of unsaturated steroidal ketones (especially 4-ene-3-one compounds). (Hill, M.J. (1977). In *Origins of Human Cancer* (Hiatt, ed.) p. 1927. Cold Spring Harbour). This reaction readily occurs *in vitro*, but only trace amounts of 4-ene-3-one steroids have been found in human faeces. We have analysed the faeces of healthy NDH clostridia carriers by gel chromatography, capillary chromatography and glc/mass spectrometry. We did not detect any unsaturated steroid ketones, but have tentatively identified a number of bile acids having the 5α (allo) ring junction. These include 3α,12α-dihroxy-5α-cholan-24-oate, 3β,12α-dihydroxy-5α-chlolan-24-oate, and 3β-hydroxy-5α-cholan-24-oate. Since these compounds are produced via a 4-ene-3-one intermediate, they provide indirect evidence for the occurrence of the NDH reaction *in vivo*. Furthermore, they could themselves modify LBC risk. Allo deoxycholic acid has been shown to be a more powerful co-mutagen than deoxycholic acid in bacterial assay systems (Wilpart *et al.* (1983), *Carcinogenesis* **4,** 1239). Data are unavailable on allo bile acids as promoters, but the potency of promoters such as phorbol esters is extremely sensitive to subtle variations in structure, including changes in ring geometry. We hypothesise that the promoting potency of bile acids is similarly structure dependent. LBC risk could thus be modulated by the metabolic activity of the intestinal flora by producing more or less potent promoters from bile acids.

### Faecal steroids and colorectal cancer: Faecal bile acids in a low risk population

M. Thompson[1], P. McKeigue[2], R. Owen[1], P. Henly[1], M. Hill[1], M. Marmot[2] & A. Adelstein[2]

[1]*BMRL, PHLS-CAMR, Salisbury, Wiltshire; and* [2]*Department of Epidemiology, London School of Hygiene and Tropical Medicine, London WC1, UK.*

It has been demonstrated that faecal bile acids (FBA) may influence the development of colorectal

**Table**

| *Population* | *LCA* | *DCA* | *CDCA* | *CA* | *Total FBA* |
|---|---|---|---|---|---|
| Asians (70) | 1.1±0.9[a] | 2.0±1.9 | 0.4±0.8 | 0.6±1.4 | 4.1±2.9 |
| Vegetarian (40) | 0.9±0.7 | 1.6±1.4 | 0.4±0.6 | 0.5±0.9 | 3.5±2.0 |
| Meat eating (21) | 1.4±1.0 | 2.6±2.6 | 0.2±0.3 | 0.2±0.3 | 4.3±3.6 |
| Welsh (36) | 2.8±2.2 | 3.9±2.3 | — | — | 6.7±5.2 |

LCA = Lithocholic acid, DCA = Deoxycholic acid, CDCA = Chenodeoxycholic acid, CA = Cholic acid.
[a]$mg\,g^{-1}$ dry faeces ± s.d.

cancer (CRC) either as initiators or promoters. In this study the FBA profiles of subjects from a low risk predominantly vegetarian Asian community in NW London, consuming a high fat-high fibre diet were compared to those of a Welsh population. Total free FBA concentrations were lower in the Asian subjects than the comparison group, with a high proportion (61%) excreting primary FBAs (CDCA and CA). In the low risk group, whilst the vegetarians excrete higher concentrations of total FBAs, a greater proportion of these acids are in the undegraded form when compared to meat eaters. Thus in this study low risk subjects excrete lower concentrations of FBAs with a known tumour promoting activity (LCA and DCA) than the high risk comparison group, with vegetarians excreting the lowest concentrations of all. Therefore the reduced risk of developing CRC in this population may be associated with the suppression of bile acid degradation in the colon.

### Gastric juice analyses in patients at high risk of gastric cancer

A. Cook[1], M. Hill[1], N. Hall[2], T. Northfield[2], J. Kirkham[2], N. Viney[3] & D. Darkin[3]

[1]*PHLS-CAMR, Salisbury, Wilts SP4 0JG;* [2]*St James' Hospital, Balham, London; and* [3]*SK & F Research Ltd., Welwyn, Hertfordshire, UK.*

An excess risk of gastric cancer has been observed in patients treated for peptic ulcer by Polya partial gastrectomy (PG) and in pernicious anaemia patients (PA). We have therefore assayed over 24 h periods the gastric juice of these high cancer risk groups of patients for bacteria, nitrate and nitrite. In 8 PA patients there was throughout the 24 h period a resident bacterial flora ($>10^6$ organisms $ml^{-1}$ including faecal species of for example streptococci and bacteroides), a high proportion of which was able to reduce nitrate. The pH of the gastric juice in almost all samples was greater than 5 – a pH favourable to bacterial growth and nitrate reductase activity. The most commonly isolated organisms were all oral species of streptococci, lactobacilli, bacteroides, veillonellae although faecal species were present. The 9 PG patients could be divided into two groups. In one ($n=5$) the gastric juice analyses were similar to those of PA patients. In the other group ($n=4$) gastric acid secretion was sufficient to produce a bactericidal pH (1.5–3.0) at night and at times between meals; at these times the gastric juice was essentially sterile ($<10^3$ organisms $ml^{-1}$) and contained no nitrate reducing bacteria and low nitrite concentrations. Meals produced a high pH ($>5$), high counts of total bacteria ($>10^6$ organisms $ml^{-1}$) and nitrate reducing bacteria ($>10^6$ organisms $ml^{-1}$) and increased but very variable nitrite concentration. All samples from all patients contained nitrate but at concentrations which ranged between 4–470 $\mu M$. Statistical analysis showed that the nitrite concentration was significantly higher in PA and group 1 of PG patients than in group 2 PG patients ($P<0.05$). The results are compatible with the hypothesis that the gastric cancer in these patients is caused by a metabolite (possibly nitrite) produced by the resident bacterial flora.

### Gastric bacterial overgrowth as a risk factor in human carcinogenesis

C.P.J. Caygill[1], M.J. Hill[1], J. Kirkham[2], & T. Northfield[2]

[1]*BMRL, PHLS-CAMR, Porton Down, Salisbury, Wiltshire SP4 0JG; and* [2]*Gastroenterology Unit, St James Hospital, Sarsfield Road, London SW12 8HW, UK.*

It has been reported that in persons with bacterial overgrowth of the stomach there is a high concentration of nitrite in the gastric juice and in some reports also of N-nitroso compounds, and that these may be the cause of the increased gastric carcinogenesis associated with this condition. If N-nitroso compounds are important human carcinogens then cancers at distant sites would also be expected. Pilot studies of Polya partial gastrectomy and of pernicious anemia patients suggested an increased incidence of colorectal and biliary tract cancers as well as of gastric cancers. In this study a group of 4,235 patients treated surgically for peptic ulcer at St James Hospital, Balham, between 1940 and 1960 have been identified, their death certificates obtained and their mortality from cancers of various organs determined using a 'years at risk' calculation in 5 year bands. The excess mortality from gastric cancer (4-fold) with a latency of 20 years, reported by others, was observed and in addition there was an excess mortality from colorectal (2.5-fold) and from biliary tract cancers (8.6-fold) also with a latency of 20 years. There was no increased risk of mortality from these cancers in the first 20 years after operation. The mortality from cancers of the pancreas, lung and all sites was as expected during the first 20 post-operative years, but was increased thereafter by 3.2, 5.0 and 2.9 fold respectively. The results are compatible with the hypothesis that N-nitroso compounds formed in the stomach may be human carcinogens but the association of gastric, biliary tract and colorectal cancers in these patients also suggests a role for modified bile. The excess of cancers of all sites and particularly the lung and pancreas may be due to the high prevalence of smoking in this group of patients.

**Development of anti-mouse IgG and anti-idiotypic antibodies by patients receiving radiolabelled monoclonal antibody (791T/36) for diagnostic immunoscintigraphy**

M.V. Pimm[1], R. Rowe[1], A.C. Perkins[2] & R.W. Baldwin[1]

[1]*Cancer Research Campaign Laboratories, University of Nottingham; and* [2]*Department of Medical Physics, University Hospital, Nottingham, UK.*

Imaging of tumours following administration of radiolabelled antibodies has been reported from a number of trials. Because antibodies used for immunoscintigraphy are of mouse origin it is likely that these will evoke antibody responses in patients and this needs to be appreciated in the design of repeated imaging protocols. However, production of anti-idiotype antibodies has been reported to beneficially modulate the patients' immune response to tumour (Koprowski *et al.* (1984), *Proc. Natl Acad. Sci.* **81**, 216). In the present study the production of anti-mouse IgG and anti-idiotype antibodies by patients receiving radio-labelled 791T/36 antibody has been assessed.

All 40 patients produced antibody reactive with 791T/36 antibody (mouse IgG2b) detected by binding of antibody to immobilized 791T/36, and formation of complexes (assayed by gel filtration) when $^{131}$I-791T/36 was added to patients' plasma. Antibody was detected within 7 days of 791T/36 administration and was present for at least 10 months. Formation of complexes *in vivo* was seen in patients receiving antibody for a second or third time. This resulted in reduction in image quality in many patients, with uptake of radiolabel predominantly in the spleen. Formation of anti-idiotype antibodies was indicated by greater binding of patients' antibodies to 791T/36 than to normal mouse IgG2b and specific inhibition of binding fluorescein 791T/36 to appropriate target cells.

These studies have shown that patients develop antibodies to a monoclonal antibody used for diagnostic immunoscintigraphy, and this may be a serious limitation for repeated imaging. The consequences of the production of anti-idiotypic antibodies has yet to be assessed.

**Increased oleic acid content in erythrocytes of cancer patients**

N. Habib[1], C. Wood[1], K. Apostolov[2], A. Thompson[1], W. Barker[2] & M. Blount[1]

*Departments of* [1]*Surgery and* [2]*Virology, Royal Postgraduate Medical School, Hammersmith Hospital, Ducane Road, London W12 0HS, UK.*

Lipids form 40% of the cell membrane mass. The main properties of the lipids including their viscosity depend mainly on the content of unsaturated fatty acids. A larger proportion of unsaturated fatty acids contributes to a higher membrane fluidity and is associated with a higher capacity for cell division. Peripheral erythrocytes were taken from 100 cancer and 80 non-cancer patients and were analysed for fatty acids contents using gas liquid chromatography.

It was found that cancer patients have a decrease in the saturation index (ratio of stearic to oleic acids) of the erythrocytes cell membranes when compared with non-cancer patients ($P<0.001$). This ratio was found to be useful in monitoring therapy in cancer patients as it is within normal range in disease-free patients and revert to abnormal range in patients with local or distant tumour recurrence.

**Histogenesis of anaplastic thyroid tumours**

A.D. Burt[1], D.J. Kerr[2], I.L. Brown[1] & L.G. Bobrow[3]

*University Departments of* [1]*Pathology and* [2]*Clinical Oncology, Glasgow; and* [3]*Department of Histopathology, University College Hospital Medical School, London, UK.*

In an immunohistochemical study of 53 anaplastic tumours of the thyroid we have identified a group of 14 tumours that express neither the lymphoid marker, common leucocyte antigen (CLA) nor the epithelial marker, epithelial membrane antigen (EMA). These tumours are characterised clinically by rapid growth with a poor response to radiotherapy and a poor prognosis (mean survival 4.5 months).

To further investigate the histogenesis of these tumours we have used the avidin biotin technique with antisera to intermediate filaments on formalin-fixed, paraffin-embedded tumour tissue. Low molecular weight intermediate filaments detected by the monoclonal antibody CAM 5.2 were demonstrated in 4/14 tumours indicating an epithelial origin. Desmin, an intermediate filament found almost exclusively in smooth muscle tissue was detected in two tumours. No tumour was found to contain vimentin. The use of antisera to cytoskeletal proteins has thus provided information regarding histogenesis in tumours that were negative for CLA and EMA. These results emphasise the need for the use of a panel of antibodies in the investigation of anaplastic tumours.

**Cytogenetic analysis of cell populations from routine diagnostic lymph node biopsies**

M. Fitchett[1], M. Griffiths[1], D.B. Jones[2], D.H. Wright[2] & J.L. Smith[3]

[1]*Wessex Regional Cytogenetics Unit, Salisbury, Wiltshire; and* [2]*University Departments of Pathology and* [3]*Immunology, Southampton, UK.*

Cytogenetic investigations have been carried out on lymph node biopsies from 21 patients. Our findings emphasise the need to use fresh specimens and the value of stimulating cultured cells with PHA or TPA in addition to analysing preparations from unstimulated cultures. Stimulated cultures frequently show an improved mitotic index and superior quality of banded metaphase spreads which allowed more detailed analysis. The use of TPA revealed abnormal clones in 5 patients whose unstimulated cells yielded either no mitoses or unanalysable metaphases. Lymphoma biopsies of all patients were fully characterised using a panel of monoclonal antibodies. Clonal karyotypic abnormalities were detected in all 16 patients with malignant B-cell disease (including one patient with CLL). Detection of only karyotypically normal cells in 4 patients confirmed an absence of lymphoid malignancy. Both of our patients with deletion of 6q showed the presence of centroblasts. Two patients with t(14; 18) had follicular lymphoma. A single patient with duplication of 1q had diffuse centroblastic lymphoma. Complex karyotypes were found in association with the presence of centroblasts in 6/7 cases, whereas 3/3 single autosomal abnormalities were associated with centrocytic lymphoma. We also report an unique case of t(2; 8) in a non-Burkitt's lymphoma and the finding of non-constitutional sex chromosome abnormalities in 2 lymphoid malignancies. Cytogenetic studies are being used to complement routine histological and monoclonal antibody investigations in the diagnosis of non-Hodgkin's lymphoma.

**Intravenous hydroxyurea and *cis*-platinum for non small cell lung cancer (NSCLC)**

B. Cantwell[1], D. Veale[1], A.L. Harris[1], J. Bozzino[1], M. Earnshaw[2] & A. Upfold[2]

[1]*Clinical Oncology and* [2]*Pharmaceutical Quality Control Laboratory, Newcastle General Hospital, Newcastle-upon-Tyne NE4 6BE, UK.*

Hydroxyurea (HO) inhibits DNA synthesis by inhibiting ribonucleotide reductase, and depletes cells of nucleotide triphosphates. With higher doses, HO inhibits DNA repair probably by preventing the filling of gaps in DNA with nucleotides. Since *cis*-platinum induces DNA interstrand cross links, the 2 drugs were combined in a clinical study in an attempt to maximise tumour cell kill by using high dose intermittent infusions of HO to prevent repair of DNA damage induced by *cis*-platinum. Patients (pts) received 24 grams of HO by i.v. infusion over 24 h and 8 h after start of infusion, *cis*-platinum $50\,mg\,m^{-2}$ i.v. Cycles were repeated 3 weekly to a maximum of 6 cycles. Forty-seven patients with inoperable assessable NSCLC have been treated, 3 of whom received only HO because of pretreatment deafness. Mean age = 59 yrs, SD $\pm$ 9.8, with 36 males, 11 females. Responses in 23 pts with squamous cell lung cancer were complete response (CR) = 1; partial response (PR) = 3; stable disease (SD) for 3 or more months = 6 pts. Responses in 14 pts with adenocarcinoma of lung were PR = 1, SD = 6. No responses in 10 pts with large cell anaplastic lung cancer. Major symptomatic toxicity was emesis (30 pts $\geqslant$ 3 vomits after *cis*-platinum), 1 pt developed urticaria after start of HO, only 2 pts developed myelosuppression which led to changes in management. Mean HO plasma levels in 6 pts during the last 8 h of i.v. infusion was $150\,\mu g\,ml^{-1}$ (range 61–235) which is level required to inhibit DNA synthesis by 80% in NSCLC cell lines *in vitro*. HO with *cis*-platinum has activity in squamous and adeno-NSCLC. We are now assessing escalating doses of HO alone in NSCLC since high dose intermittent infusional HO appeared to lack significant toxicity in doses used so far.

### Randomised trial of oral out-patient chemotherapy *versus* 4 drug intravenous chemotherapy for small cell lung cancer (SCLC)

B. Cantwell, A.L. Harris, J. Bozzino, P. Corris, S. Nariman, S. Pearce, J. Gibson, D. Hendrick, A. Lishman & A. Hendrick

*Department of Clinical Oncology, Newcastle General Hospital, Newcastle-upon-Tyne NE4 6BE, UK.*

We have found i.v. infusions of ifosfamide (ifos) and mesna, both in doses of $5\,g\,m^{-2}$ to be active 2nd line treatment for SCLC in a crossover study (Cantwell *et al.* (1984), *Br. J. Cancer* **50,** 247). Short courses of ifos and mesna were relatively marrow sparing and lacked significant urinary tract toxicity. We wished therefore to examine ifos earlier in the course of SCLC, in a comparison of i.v. *vs* an oral drug regimen both given for 4 courses. Seventy-four patients (53 limited, 21 extensive disease) have so far been treated. The i.v. arm consists of course 1: VP16 $100\,mg\,m^{-2}$ i.v. day 1 and 300 mg orally on days 2 and 3, adriamycin $40\,mg\,m^{-2}$ and vincristine 2 mg i.v. both on day 1. Courses 2, 3 and 4: ifos $5\,g\,m^{-2}$ 24 h i.v. infusion with mesna $5\,g\,m^{-2}$ i.v. before, during and after ifos infusion, VP16 $100\,mg\,m^{-2}$ at 0 h and at 24 h, adriamycin $30\,mg\,m^{-2}$, vincristine 2 mg i.v. Oral arm: chlorambucil 10 mg daily, procarbazine 50 mg tds, prednisolone EC 10 mg bd, all given days 1–10 and VP16 300 mg days 1–3, oral and i.v. chemotherapies given 3-weekly to a total of 4 courses. Patients with limited disease and good response subsequently receive radiotherapy to primary site and prophylactic cranial RT. Forty patients are assessable for response to chemotherapy (20 in i.v., 20 in oral arm). In i.v. arm there are 14 (70%) objective responses (5CR, 9PR) and in oral arm 10 (50%) objective responses (3CR, 7PR). In the i.v. arm 5 patients and 6 in the oral arm had at least 1 episode of leucopenia. Changes in therapy as a result were more frequent in the oral arm, but the only episode of certain septicaemia occurred after course 1 in the i.v. arm. Early results suggest that oral chemotherapy offers an easily administered out-patient alternative for remission induction in small cell lung cancer. Patient accrual continues.

### A pharmacokinetic, endocrine and clinical study of the LHRH analogue DSer(tBU) 6 AzaGly10 GnRH (ICI 118630)

T.J. Perren[1], R.N. Clayton[1], G.R.P. Blackledge[1], D. Arkell[2], J. Cottam[2], D. Farrar[3] & C. Young[3]

[1]*Department of Medicine, University of Birmingham;* [2]*Department of Urology, Dudley Road Hospital; and* [3]*Department of Urology, Selly Oak Hospital, Birmingham, UK.*

Seventeen patients with advanced prostatic cancer were treated with the gonadotrophin releasing hormone (GnRH) analogue DSer(tBU)6 AzaGly10 GnRH (ICI 118630) either as a constant s.c. infusion (I) ($n=4$), or in the form of a slow release depot formulation (D) ($n=13$) in which case patients were randomised to receive one of 3 doses. Two out of four I patients later went onto D. Six patients also received a single 250 $\mu$g s.c. bolus of ICI 118630 before starting I or D for pharmacokinetic studies. Both I and D were effective in reducing serum LH, and testosterone to castrate levels by 1 month. Drug levels were measured using a double antibody radioimmunoassay. In contrast to I which gives a smooth drug level profile, drug release from D was not constant, levels varied in a predictable manner throughout each 28 day period reaching a peak proportional to dose, on day 15–18 of each cycle. Treatment with ICI 118630 appears effective, 7/9 patients evaluable for clinical response showed a greater than 50% reduction in prostatic dimensions. Toxicity was minimal 4/17 had an initial flare in bone pain, 13/17 had flushing. D preparations of GnRH analogues show great promise for the treatment of advanced prostatic cancer and may well become the treatment of choice.

### BCNU with autologous bone marrow grafting for malignant melanoma and brain tumours

E. Mbidde, P. Selby, J. Maitland, H.J.G. Bloom & T.J. McElwain

*Royal Marsden Hospital, Down Road, Sutton, Surrey, UK.*

BCNU is active in the treatment of malignant melanoma and brain tumours but its dose is limited mainly by myelosuppression. Marrow autografting avoids this toxicity. Although the plasma half life of BCNU is short, cryopreservation of marrow cells has previously been necessary to allow administration of BCNU by a 24 h infusion. We have given BCNU $800\,mg\,m^{-2}$ by a bolus injection into a central venous line in 9 ml of absolute alcohol to 30 patients (pts) after a bone marrow harvest and returned the marrow, without cryopreservation 12 h later. Five pts were treated twice. The procedure was well tolerated. Leucopenia $<1\times10^{9}$ cells $l^{-1}$ occurred in 20 pts (median duration 3 days, range 0–27 days) with no life threatening infections. Thrombocytopenia $<25\times10^{9}$ platelets $l^{-1}$ occurred in 18 pts (median duration 1 day). Two pts had prolonged thrombocytopenia $>60$ days. Partial alopecia in 3 pts. Moderately severe pneumonitus in 3 pts. Biochemical hepatitis occurred in 1 pt but no treatment related deaths. The pts were managed as out-patients with short admissions only. Among 11 previously untreated melanoma pts there were 4 partial remissions lasting 1.5, 2, 2 and 5 months and one complete remission lasting 6 months. Among 11 previously treated pts there are no responses with one pt too early to assess. The treatment appears to offer no advance in the treatment of melanoma. The brain tumour pts (7 astrocytoma Grade IV) are being treated with BCNU followed by radiotherapy. The treatment is generally well tolerated and 6/7 glioma pts have had substantial improvements in CT scans after BCNU but it is too early to comment on the overall efficacy of the combined treatment.

### Effect of cholecystokinin on human pancreatic and gastric cancer in nude mice

C. Hudd[1], M. LaRegina[2], D. Palmer[3], D. Herbold[3], J. Devine[3] & F. Johnson[1]

*Departments of [1]Surgery and [2]Comparative Medicine; and [3]Pathology, St Louis University Medical Center, St Louis, MO, USA.*

Gastrointestinal (GI) hormones regulate growth of normal GI tissues as well as certain GI cancers (Hudd *et al.* (1984), *Gastroenterology* **86**, 1118). Since CCK promotes growth of normal pancreas and also inhibits the trophic effect of gastrin on stomach, we studied the impact of CCK on human pancreatic and gastric cancer. In two separate experiments, groups of nude mice bearing s.c. nodules of human pancreatic or gastric cancer received either saline or synthetic sulfated CCK-8 (Squibb), $50\,mg\,kg^{-1}\,dose^{-1}$ BID i.p. for 14 days: a dose, route, and schedule which produces maximum pancreatic growth in the nude mouse. Tumour volumes were calculated from vernier caliper measurements taken every 3 days. On day 15, pancreas and tumour were excised, weighed, and submitted for histology (H & E) biochemistry (DNA, RNA, and protein content), and CEA staining. Results were analyzed by the Student *t*-test and are expressed as mean ± s.e.

We saw no histological differences between groups in either series. We conclude that: (1) The dose of CCK was biologically active. (2) CCK did not affect growth of human pancreas cancer line P1420, but retarded growth of human gastric cancer line SLU 077.

### Alternating platinum combination chemotherapy in gynaecological malignancies

F.G. Lawton, G.R.P. Blackledge & J.J. Mould

*West Midlands Ovarian Cancer Group, Queen Elizabeth Medical Centre, Birmingham, UK.*

Platinum (P) based combination chemotherapy regimes produce high response rates in epithelial ovarian cancer (EOC), but are associated with sometimes unacceptable toxicity. Alternating the P and non-P arms allows a large number of effective drugs to be used as initial treatment and may also reduce cumulative toxicity.

Twenty patients (18 EOC, 1 Ca endometrium + ovary, 1 Ca Fallopian tune) were given P ($60–100\,mg\,m^{-2}$) and cyclophosphamide ($600\,mg\,m^{-2}$) i.v. alternating 3 weekly with cycles of adriamycin ($50\,mg\,m^{-2}$), bleomycin 15 mg i.v. and chlorambucil ($6\,mg\,m^{-2}$) od. 1–7 (CP-ABC). A total of 3 courses of CP-ABC were planned.

Only 14/120 courses were delayed. (Nine by 1 week, 3 by 2 weeks, 2 by 3 weeks).. (Seven neutropenia, 4 thrombocytopenia, 3 excess vomiting). Alopecia was common (5 WHO Gd 1, 7 Gd 2, 4 Gd 3) but reversible in all cases. Haematological toxicity (5 Gd 1, 4 Gd 2, 3 Gd 3) was only severe in heavily pretreated patients. Only 5 patients experienced worse than Gd 1 nausea. Three patients required blood transfusion. In 2 patients creatinine clearance fell below $50\,ml\,min^{-1}$.

Five out of nine evaluable patients responded to treatment (55%).

Such alternating regimes could allow the incorporation of greater numbers of active drugs as first-line treatment of EOC with little resulting cumulative or cross toxicity.

**Table**

| | *Pancreas cancer P1420* | | *Gastric cancer SLU-077* | |
|---|---|---|---|---|
| | *Pancreas wt (9% body wt)* | *Tumor vol (cm³) Day 15* | *Pancreas wt (9%body wt)* | *Tumor vol (cm³) Day 15* |
| CCK | 1.38 ± 0.05 | 2.19 ± 0.57 | 1.31 ± 0.09 | 0.34 ± 0.06 |
| Saline | 1.19 ± 0.04 | 2.22 ± 0.34 | 0.88 ± 0.05 | 0.82 ± 0.20 |
| | $P<0.01$ | $P=NS$ | $P<0.001$ | $P=0.05$ |

**Table**

| | Nausea % of scale | Vomiting % None | 1–2 | 3–4 | >6 | Meals after $R_x$ |
|---|---|---|---|---|---|---|
| D/M | 29%[a] | 62 | 7 | 7 | 24[b] | 68% |
| S/M | 45% | 28 | 17 | 3 | 52 | 49% |

[a]$P=0.001$. [b]$P=0.05$.

### Anti-emetic effect of dexamethasone (D) in out-patient chemotherapy

D.L. Farquhar, S.G. Allan, D. Harrison & R.C.F. Leonard

*Departments of Clinical Oncology and Surgery, University of Edinburgh, Edinburgh, Scotland, UK.*

A single blind trial was designed to assess the efficacy of D as an anti-emetic agent for out-patient cytotoxic chemotherapies including the potent emetic drugs, cyclophosphamide and adriamycin. All patients had breast carcinoma and received either adjuvant CMF or advanced disease combination chemotherapy including adriamycin. Patients were randomised to receive either D 16 mg i.v. and oral Motival 1 tablet tds (D/M) or N-saline i.v. and Motival (S/M). They received the alternative therapy on their subsequent course allowing direct comparison. Patients could be included in the trial for more than one randomisation. The study includes 19 patients (30 paired courses of therapy). Patients assessed nausea on a visual analogue scale, stated number of vomits following chemotherapy and meals eaten on the following day. Results show a significant reduction in patients' assessment of nausea and numbers of vomits in the D/M compared with the S/M group. In only 4 of 30 paired assessments was S/M associated with less nausea than D/M. This confirms that D is an effective antiemetic agent in out-patient chemotherapy.

### A comparative study of nabilone and prochlorperazine *versus* nabilone and placebo in the control of emesis induced by cytotoxic drugs

D. Cunningham, G.J. Forrest, N.L. Gilchrist, I. Calder & M. Soukop

*Department of Medical Oncology, Royal Infirmary, Glasgow, UK.*

Nabilone, a synthetic cannabinoid with structural similarities to tetrahydrocannabinol (THC) is a potent antiemetic, but dysphoric reactions have limited its use. The addition of prochlorperazine to THC has been shown to reduce the frequency of CNS side effects, therefore the purpose of this study was to investigate whether such an effect would be observed with the addition of prochlorperazine to nabilone. Thirty patients, mean age 54 years (range 39–76) receiving a variety of cytotoxic chemotherapy regimes without *cis*-platin entered this double blind crossover study comparing nabilone 2 mg and prochlorperazine 5 mg (N+P) with nabilone 2 mg and placebo (N) both given 12 hourly for 4 doses. Three patients had previously received chemotherapy, but none had been given nabilone or had taken cannabis in the past. There was complete control of vomiting in 80% of patients given N+P and 80% of patients given N. Toxicity was similar with both antiemetic regimes with regard to sedation (85%) and dizziness (60%) but more patients developed a dry mouth with NS (90% *vs* 76%). However, only 5% complained of dysphoria with N+P compared to 50% with N ($P<0.01$). Furthermore 45% of patients preferred N+P as an antimetic because of the reduction of dysphoria ($P<0.01$), 5% preferred N and 50% had no preference. This study has shown that nabilone and prochlorperazine is an effective antiemetic and that the dysphoric effects of nabilone are significantly reduced by the addition of prochlorperazine.

### Protein synthesis rates in an animal model of cancer cachexia

J.A. Plumb & K.C. Calman

*Department of Climical Oncology, University of Glasgow, Horselethill Road, Glasgow G12 9LX, UK.*

A chemically induced adenocarcinoma of the mouse colon (MAC-16) can be grown subcutaneously in NMRI mice. After 4 weeks the tumour grows to ~6% of the mouse body wt. Although the mice maintain their normal food intake for most of the time, their body weight decreases by ~20%. Clearly the weight loss cannot be accounted for either by the tumour growth rate or by a reduced food intake; a situation similar to the severe weight loss characteristic of cancer cachexia. After 4 weeks the protein content of the gastrocnemious muscle

(total ninhydrin positive material after acid hydrolysis) is decreased by 35% ($P<0.01$, $n=8$). Protein synthesis rates were measured *in vivo* by the method of Garlick *et al.* (1980), (*J. Biochem.* **192,** 219). There was no difference between the rates in muscle from control mice and from tumour bearing mice. It is concluded that the loss of skeletal muscle in tumour bearing mice is probably due to an increased rate of protein degradation.

### Body weight loss (cancer cachexia) following transplantation of an adenocarcinoma of the mouse colon (MAC 16)

S.A. Ali, M.C. Bibby & J.A. Double

*Clinical Oncology Unit, University of Bradford BD15 7AX, West Yorkshire, UK.*

MAC 16 is one of the MAC series of colon tumours originally induced in NMRI mice by dimethylhydrazine (Double *et al.* (1975), *J. Natl Cancer Inst.* **54,** 271). It is a moderately well-differentiated adenocarcinoma transplanted subcutaneously in the flank. The tumour has a volume doubling time of ~6 days and produces a reduction in body wt of ~20% over a period of 3–4 weeks. This weight loss is not accompanied by a decrease in food intake and there is no evidence of ketosis. Excision of the tumour results in cessation of weight loss with subsequent weight gain on recovery. Occasional local recurrence of tumour growth is accompanied by further loss of body wt. Twice weekly i.v. injection of serum from cachectic tumour bearing mice into normal mice over a period of 4 weeks did not affect body wt. A tumour product is therefore unlikely to be responsible for the cachexia.

The anti-tumour activity of a series of agents against MAC 16 has been determined. Response was measured by growth delay from semi-log plots of relative tumour volumes calculated from serial caliper measurements. Like other tumour lines within this series responses are only seen close to the maximum tolerated dose. The best anti-tumour responses are seen with 5-fluorouracil and cyclophosphamide. MAC 16 does not respond to methyl CCNU or mitozolamide both of which have been shown to be very effective against other lines of the MAC series.

### Inhibition of tumour growth *in vivo* by manipulation of cellular energy metabolism

K.C.H. Fearon, J.A. Plumb, R. Sri-Pathmanathan & K.C. Calman

*Department of Clinical Oncology, University of Glasgow, 1 Horselethill Road, Glasgow G12 9LX, UK.*

The mitochondria of many tumours are known to be abnormal in number, morphology and enzyme content. Moreover, many tumours are recognised to have high rates of glycolysis and to be glucose dependent. These abnormalities of the two major sources of intracellular ATP have suggested that the manipulation of tumour cell energy metabolism might provide a method for selective inhibition of tumour cell growth.

The fluorescent dye Rhodamine 1,2,3(R1,2,3) inhibits the growth of Ehrlich ascites tumour in mice and its activity is potentiated by the glycolytic inhibitor 2-deoxyglucose (Bernal *et al.* (1983), *Science* **222,** 169). We have studied the effects of i.p. administration of the antimitochondrial dyes R1,2,3 and Rhodamine 6G (R6G) on the growth rate of the Walker 256 carcinosarcoma in rats. The inhibition of tumour growth when these drugs were administered in combination with 2-deoxyglucose or hydrazine sulphate (inhibitor of gluconeogenesis) was also assessed.

The anti-tumour effects of R1,2,3 were potentiated by 2-deoxyglucose but little inhibition of tumour growth occurred if the administration of either or both drugs commenced more than 24 h after tumour implantation. Alternatively, R6G was a more potent drug and inhibited tumour growth when administered more than 48 h after tumour implanatation. The efficacy of R6G was increased by hydrazine sulphate but not by 2-deoxyglucose. Tumour cell energy metabolism, therefore, has the potential as an important new target for chemotherapeutic intervention.

### The computerised storage and retrieval of chemical and experimental antitumour data

J.A. Slack, M.D. Threadgill, D. Chubb, B. Kemp, J.A. Hickman & K.J. Bowcock

*CRC Experimental Chemotherapy Group, University of Aston, Birmingham, UK.*

A package of programs has been developed for the storage and retrieval of chemical, structural and biological data concerning compounds and their activity against experimental murine tumours *in vivo*. Access to the database is gained *via* Fortran application programs using RAPPORT commands on a Harris H500 minicomputer. The chemical data stored consists of the molecular formula, solubility and IUPAC systematic name of each compound together with a concise code describing functional groups and rings. This code was developed to be much simpler to use than the Wiswesser system, recognising some 200 major functional groups and the Chemical Abstracts Ring Index. Compounds are indexed through a unique Compound Code. The biological data comprises such information as dose, schedule, host toxicity and various antitumour evaluation parameters. Three application programs enable all users access to the data without prior knowledge of database structure and organisation. In the first, iterative searches are available to retrieve data on all compounds in the database having particular chosen groups and ring systems. The second gives a summary of all biological data for a given compound and the third provides full screening and chemical data for a given agent against the selected tumour. Data is currently held on >200 compounds and >300 tests.

**Development of drug resistance in a murine mammary carcinoma**

T.J. McMillan, T.C. Stephens & G.G. Steel

*Radiotherapy Research Unit, Institute of Cancer Research, Sutton, Surrey, UK.*

The MT murine mammary carcinoma (caMT) was examined before and after courses of treatment with either melphalan, cyclophosphamide or *cis*-platinum II in an attempt to understand the mechanisms involved in the development of drug resistance.

Single high-dose treatments were given in each passage and the loss of therapeutic response was assessed using a growth delay end point. Growth delay at the given doses fell by a factor of 4.1 after 16 treatments with $12\,mg\,kg^{-1}$ melphalan, 10.6 after 20 treatments with $180\,mg\,kg^{-1}$ cyclophosphamide and 3.8 after 20 treatments with $10\,mg\,kg^{-1}$ *cis*-platinum. The decrease in sensitivity was confirmed by clonogenic cell survival following either *in vivo* or *in vitro* drug treatment. The rate of development of resistance to cyclophosphamide was increased by prior treatment with the classical mutagen EMS or with melphalan, but two widely differing doses of cyclophosphamide brought about resistance at equal rates.

The rates of drug-resistance development were much slower than would be predicted by current models involving the selection of a pre-existing highly drug-resistant subpopulation. However, studies with clonal lines from untreated tumours indicated that clones with a wide spectrum of sensitivity are present in caMT (range of $D_{10}$ values of *in vitro* melphalan dose survival curve = 0.35–0.98 $\mu g\,ml^{-1}$). A model based on such a spectrum of sensitivities suggests that drug-resistance may emerge more slowly and this may therefore be a better representation of the drug-resistance development data obtained with caMT.

**The effect of the rate of cell proliferation on the synthesis of methotrexate poly-γ-glutamates in two human breast cancer cell lines**

D.G. Kennedy[1], H.W. van den Berg,[2], R. Clarke[1] & R.F. Murphy[1]

*Departments of [1]Biochemistry and [2]Therapeutics, The Queen's University of Belfast, NI, UK.*

The therapeutic and toxic effects of methotrexate (MTX), may in part be dependent on the rate and extent of intracellular formation of poly-γ-glutamyl (PG) derivatives. These metabolites are extensively retained within the cell and are at least as effective as the parent drug in inhibiting dihydrofolate reductase. As part of a study designed to investigate factors controlling MTX-PG synthesis we have examined the influence of initial cell plating density on the rate of cell proliferation and MTX-PG synthesis in the MCF-7 and MDA-MB-436 human breast cancer cell lines.

Our results demonstrate that although slowly proliferating cells accumulate MTX to the same extent as rapidly proliferating cells, they convert a lower percentage of the drug to PG forms. The MDA-MB-436 line exhibited a biphasic response of both doubling time and polyglutamation to increasing initial cell number. Extremes of cell density were associated with long doubling times (>80 h), and reduced PG synthesis (50–75% of total intracellular drug). Optimum cell densities for PG synthesis (>85% of total drug), was associated with a more rapid growth rate (doubling time 30–40 h). MCF-7 cells showed increasing doubling time (25→>80 h), and decreasing PG synthesis (80→28% of total drug) with increasing initial cell number.

We conclude that the decreased extent of MTX-PG synthesis in slowly proliferating cells may provide an additional mechanism by such cells which are more resistant to the effects of MTX.

**The growth of exfoliated colorectal carcinoma cells in immune deprived mice**

H.C. Umpleby, B. Fermor, J.V. Lever, M.O. Symes & R.C.N. Williamson

*Departments of Surgery and Pathology, University of Bristol, UK.*

The viability of exfoliated colorectal cancer cells is indicated by exclusion of trypan blue and fluorescence following exposure to fluorescein diacetate (Umpleby *et al.* (1984), *Br. J. Surg.* **71,** 659). To determine whether such cancer cells can undergo further proliferation they were transplanted into 4 month old mice, previously subjected to thymectomy, 9 Gy whole body irradiation and isogenic bone marrow injection.

In 17 patients with carcinoma of the colorectum the operative specimen was lavaged with TCM 199 and cancer cells in the fluid were concentrated on a Nycodenz (Nyegaard, Oslo) column. Between 0.1 and $1.2 \times 10^6$ (median $0.75 \times 10^6$) viable cells from each tumour were injected i.v. into separate groups of 1–5 immune-deprived mice. The animals were killed 2 weeks later, their lungs fixed in Bouins fluid and macroscopic nodules were examined histologically for foci of colorectal carcinoma. Cells from 7 of the carcinomas formed pulmonary metastases in one or more mice. No metastases were seen in 12 immune-deprived mice receiving TCM 199 alone. Thus exfoliated colorectal carcinoma cells can undergo further division and might account for the development of implantation recurrence in man.

### A comparison of crypt-cell proliferation in rat colonic mucosa *in vivo* and *in vitro*

K.J. Finney,[1] P. Ince,[1] D.R. Appleton,[2] J.P. Sunter[1] & A.J. Watson[1]

[1]*Department of Pathology, Royal Victoria Infirmary, Newcastle-upon-Tyne NE1 4LP; and* [2]*Department of Medical Statistics, University of Newcastle-upon-Tyne, UK.*

The successful development of a long-term organ-culture system has made it possible to perform experiments on rat colonic mucosa *in vitro*. To interpret these experiments it is necessary to compare proliferative parameters *in vitro* with those *in vivo*, since fundamental changes in these parameters, due to trauma or the withdrawal of trophic factors, may occur when the mucosa is cultured.

Stathmokinetic experiments were performed *in vivo* and *in vitro* to estimate cell birth-rate. Mitotic and labelling indices were also calculated. The *in-vivo* birth rate ($7.8 \pm 0.8$ cells 1000 cells$^{-1}$ h$^{-1}$) and the *in-vitro* birth-rate for the whole explant ($7.7 \pm 0.5$ cells 1000 cells$^{-1}$ h$^{-1}$) were found to be similar.

However, when only perfect axially sectioned crypts in the centre of the cultured tissue were counted values for the mitotic and labelling indices were found to be inconsistent with those of the previous whole-explant study. To further investigate this observation the explants were, for the purpose of counting, divided into edge and middle regions.

Values obtained for birth-rate, mitotic and labelling indices indicate that cultured explants show enhanced proliferation at the edges compared to the centre. Provided that this difference is recognised the *in-vitro* model may still be regarded as a valid system for study.

### Morphometric studies with human malignant melanoma xenografts

S. Sparrow

*Toxicology Unit, Medical Research Council Laboratories, Woodmansterne Road, Carshalton, Surrey SM5 4EF, UK.*

An interactive image analysis system was used to measure the size and shape of nuclei and cells, the distance between blood vessels and the amount of necrosis in five different malignant melanoma xenografts. A Cruz–Orive transformation was used to convert the actual measured profiles of nuclei and cells in $4 \mu m$ histological sections of the tumours into estimated values for nuclei and cell diameters. Analysis of the results showed reasonably consistent values for mean nuclear diameter in four of the xenografts ($\sim 9.2 \mu m$) and a much lower value for the fifth ($7.5 \mu m$). The tumours show different distribution patterns for nuclear diameters, suggesting some variation in the degree of polyploidy. Cell size showed greater variation between the different tumours and there appeared to be a correlation between the mean cell size and the rates of growth of the xenografts obtained from previous studies. The most interesting findings were the changes that occurred in cell size following chemotherapy of the tumours. Those tumours that showed some response in terms of reduced growth rate also showed changes in cell size. The relationships were even more striking when the degree of melanogenesis (as a measure of cell differentiation) and the mitotic count (cell proliferation) were included in the analysis. It was concluded that morphometric analysis, made much simpler by modern computing methods, may prove a valuable tool in studying tumour biology.

### Protective role of MESNA on the gastrointestinal toxicity of *cis*-platinum

S.G. Allan[1], F.G. Hay[2], R.C.F. Leonard[1], J.F. Smyth[1] & C.R. Wolf[1]

[1]*Imperial Cancer Research Fund Medical Oncology Unit, Western General Hospital; and* [2]*University Department of Clinical Oncology, Western General Hospital, Edinburgh, UK.*

The contribution of gut toxicity to the severe vomiting (and diarrhoea) that attend *cis*-platinum (P) therapy is uncertain but may be important. Morphological and kinetic changes produced by P in the mouse small intestine have been examined using a microdissection technique. Partial villous atrophy and loss of crypts were noted 3–7 days after P (10 mg kg$^{-1}$ i.p.) with subsequent recovery. Marked inhibition of mitotic activity was seen 24 h after P with a marked reduction in crypt cell production rate per villus (CCPR/V). On day 5 after P, jejunal crypts were hyperactive with a rebound in CCPR/V. CCPR/V showed a more gradual recovery in the ileum suggesting more intensive damage to this portion of the intestine. Thiol containing compounds, e.g. metallothionines, are intimately involved in the prevention of metal toxicity. The synthetic thiol MESNA (M) was administered to groups of 6 mice ($400\,mg\,kg^{-1}$ oral) 2 h before, at the time of and 2 h after P ($10\,mg\,kg^{-1}$ i.p.). Controls received P ($10\,mg\,kg^{-1}$ i.p.), saline (0.2 ml i.p.) and M ($400\,mg\,kg^{-1}$ oral). After colchicine induced metaphase arrest standard portions of jejunum and ileum were taken on days 1, 2, 3, 5, 7 and 10 after P, and analysed in blinded sequence for damage.

M protected the animals against a normally lethal dose of P, was associated with a reduced degree of weight loss and considerably reduced the morphological insult to the gut with enhanced kinetic recovery. The differences between P and P+M are statistically significant by analysis of variance. These data provide a potential method for quantifying the gastrointestinal toxicity of *cis*-platinum but whether the protective mechanism of M against P lethality is due to protection in kidney or the GI tract remains to be established.

### Platinum drug toxicity

M. Laverick[1], M. Gordon[1], P. Kind[2], B. Slavin[2] & A.H.W. Nias[1]

*[1]Research Center, Chinoin Works; and [2]Department and [2]Department of Chemical Pathology, St Thomas's Hospital Medical School, London SE1 7EH, UK.*

Neoplatin is the original platinum coordination complex used in the successful chemotherapy of ovarian, testicular and other human tumours. Apart from causing haematological depression, the drug is nephrotoxic and alternative platinum drugs have been sought which are at least as effective antitumour agents but with less toxicity. CHIP (JM9) has been selected as one of the second generation platinum drugs because it is more soluble than Neoplatin and might be expected to be less nephrotoxic. Previous biochemical data have been based upon simple laboratory tests. In this work, the assays are those used routinely in a chemical pathology laboratory. The dose level used was the maximum tolerated dose (MTD) for C3H mice determined by lethality and intestinal crypt survival assays to be $40\,mg\,kg^{-1}$ for CHIP. This was compared with the MTD of Neoplatin of $10\,mg\,kg^{-1}$. The time course of gastric distension and the pattern of drug distribution was assayed after a MTD of CHIP. A high level of drug uptake was found in liver as well as kidney. For this reason, tests for both kidney and liver damage were undertaken up to 60 days post-treatment with Neoplatin and CHIP. Reference ranges for all the biochemical assays were first determined. (There is a fall in alkaline phosphatase level with age.) Despite the high level of platinum drug uptake in liver, there was no evidence of hepatocellular or cholestatic damage. There was the expected rise in serum urea after Neoplatin but not after CHIP. Other sensitive assays of renal function showed minimal evidence of damage after both drugs. Some enzyme levels and urinary protein were depressed after drug treatment, to a greater extent after Neoplatin than CHIP. However, the levels in tumour bearing mice were found to be depressed even before treatment.

### Pharmacokinetic study of local dibromodulcitol (DBD) treatment

J. Kaczmarek[1], L. Institóris[1], Gy. Pethes[2] & P. Rudas[2]

*[1]Research Center, Chinoin Works and [2]Department of Physiology, University of Veterinary Medicine, POB 110, Budapest, H-1325, Hungary*

In earlier studies oral DBD treatment revealed outstanding effects in the urinary bladder cancer (RR = 51%) and in gynecological malignancies (RR = 44%).

We have investigated the absorption of DBD from bladder and vagina of rats. After ligation of the ureters 4.0 mg of 1-H3-DBD in 1:1 DMSO-saline solution was introduced transurethrally into the bladder. One and 3 h following administration 2% respectively 6–8% of the administered dose was absorbed. Plasma concentration of the radiolabelled substances at the same two periods was $4.4\,\mu g\,ml^{-1}$ and $15.2\,\mu g\,ml^{-1}$, respectively, which included 6 and 3% unchanged DBD, 32 and 22% bifunctional metabolites (1-Br-5,6-anhydrogalactitol and 1,2:5,6-dianhydrogalactitol), 33 and 28% monofunctional metabolites (1-Br-3,6-anhydrogalactitol and 1,2,:3,6-dianhydrogalactitol), as well as 29 and 47% unidentified derivatives determined by TLC. Twelve milligrams of 1-H3-DBD powder was deposited in the vagina of the rats. Plasma concentration of the radiolabelled substances 3 h after treatment was $1.0\,\mu g\,ml^{-1}$. Distribution of DBD and its metabolites was similar to that observed in the case of intravesical treatment.

During the past year the local DBD treatment of 30 post-TUR bladder cancer patients have been started. No relapses have so far occurred.

### A detailed investigation of the activation mechanism of diaziquone (AZQ) as a model compound of aziridinylquinone antitumour agents

R. Driebergen[1], W. van Oort[1], S. Postma[2], D. Reinhoudt[2] & W. Verboom[2]

[1]*Faculty of Pharmacy, Utrecht,* [2]*Department of Organic Chemistry, Twente, The Netherlands*

Among numerous aziridinylquinones synthesized, several showed promising antitumour activity in experimental tumour models, in particular Carboquone, Trenimon and AZQ. Nevertheless, clinical trials of these compounds, particularly of AZQ, were disappointing, even when this compound proved to be clinically useful for the treatment of CNS cancer types. A detailed study of the activation mechanism, which probably consists of an electrochemical (reduction of quinone ring) and/or a chemical step (opening of aziridine(s)), using electrochemical techniques, might contribute to more understanding about activity and toxicity *in vivo*. To distinguish the role of individual substituents, more simple analogues of AZQ, 2,5-bis(1-az)3, 6 - bis(ethoxycarbonylamino - 1, 4 - benzoquinone (**1**), have been synthesized: 2,5-bis(1-az) - 1, 4 - benzoquinone (**2**), 2-(1-az) - 1, 4 - and 2-(1-az) - 3 - (ethoxycarbonylamino) - 1, 4, 4-naphtoquinone (**3**, **4**). Chemical and electrochemical properties have been determined by direct current and differential pulse polarography, cyclic voltammetry, etc. Polarographically obtained pk values (see Table) of the (first) aziridinyl ring of the quinones ($pk_1$) and hydroquinones ($pk_2$) of **1–4** show, that protonation of aziridines and subsequent ring opening is highly favoured by reduction to the hydroquinone derivatives (which might also influence formation of toxic oxygen compounds in aerobic media) and presence of the ethoxycarbonyl substituent. When **1–4** are in oxidized, quinonoid form, protonation proceeds faster in unsubstituted quinones **2** and **3**. Rate constants obtained from kinetic studies of the acid catalyzed opening of the aziridinyl ring(s) show this influence of quinone ring substitution. Biological data indicate, that parameters describing chemical stability of aziridines can be useful to tune cytostatic activity.

**Table**

| *Compound* | $E\frac{1}{2}(mV)^a$ | $pk_1$ | $pk_2$ | $Kobs^b$ | $ID_{75}\,ng\,ml^{-1c}$ | $T/C^d$ |
|---|---|---|---|---|---|---|
| 1 | −300 | 2.1 | 8.1 | 0.4 | 1078 | 189 |
| 2 | −145 | 3.9 | 7.5 | 15.5 | 4 | 156 |
| 3 | −305 | 3.3 | 8.1 | 5.6 | 2360 | nd |
| 4 | −300 | 1.5 | 8.4 | 0.2 | 3957 | nd |

[a]pH = 8.0; $\mu = 0.1$; 20°C. [b]pH = 4.0; $\mu = 0.1$; 20°C. [c]$L_{1210}$ clonogenic assay. [d]$L_{1210}$ *in vivo*.

### Synthesis and binding properties of analogues of $(pA)_3$ an antagonist of 2-5A action

J.C. Jamoulle[1], K. Lesiak[2] and P.F. Torrence[2]

[1]*Institute of Pharmacy, University of Liège, 3, rue Fusch, B-4000 Liège, Belgium; and* [2]*Laboratory of Chemistry, National Institutes of Health, Bethesda, 20205 MD, USA.*

To explore the role played by the purine 6-amino group of 5′-0-phosphoryl adenylyl(2′, 5′)adenylyl(2′, 5′)adenosine, $(pA)_3$, an antagonist of 2–5A action (2.5A:pp$(pA)_3$), analogues were prepared via a $Pb^{++}$ catalyzed coupling procedure. Their relative ability to bind to the 2–5A dependent endonuclease (RNase L) was determined in an assay based on the displacement of 50% of the radiolabelled probe pp$(pA)_3$ [$^{32}$P] pCp (C: cytosine) from an RNase-nitrocellulose complex ($IC_{50}$). The 6-methyl-adenosine ($m^6$A) analogue p5′A2′p5′ ($m^6$A)2′p5′ ($m^6$A) showed approximatively the same binding ability, ($IC_{50}$:$6\times10^{-7}$ M) as (p5′ ($m^6$A))$_3$, ($IC_{50}$:$8\times10^{-8}$ M) which was bound to the RNase L about 400 times less effectively than $(pA)_3$, ($IC_{50}$:$2,4\times10^{-9}$ M). This implies that methylation of the amino group must disrupt binding at either the second or the third residue of $(pA)_3$. The analogue containing guanosine (G) in the 5′ position, p5′G2′[p5′($c^7$A)]$_2$ was bound to the RNase L $10^4$ times less effectively ($IC_{50}$:$1\times10^{-5}$) than either (p$c^7$A)$_3$, ($IC_{50}$:$4\times10^{-9}$ M) or $(pA)_3$ itself, ($c^7$A: 7-deazaadenosine). This implies that either the 2-oxo function of guanosine may interfere with binding or that the N6 amino group of adenosine is necessary but not sufficient for high endonuclease activity. In any event the importance of the 5′ terminus nucleotide in enzyme binding is suggested by this finding.

### The effects of mild hyperthermia on the metabolism of three nitroimidazole radiosensitizers *in vivo* and *in vitro*

M.I. Walton, N.M. Bleehen & P. Workman

*MRC Unit and University Department of Clinical Oncology, Hills Road, Cambridge CB2 2QH, UK.*

Hyperthermia is being evaluated clinically in combination with radiation and drugs. Hyperthermia also causes enhanced tumour cytotoxicity with nitroimidazole radiosensitizers *in vitro*, possibly through toxic metabolite production. We have investigated the effects of mild hyperthermia on the metabolism of 3 nitroimidazoles. These reactions were the oxidative demethylation of misonidazole (MISO) to Ro 05-9963, the N-oxidation of Ro 03-8799 to Ro 31-0313 and the nitroreduction of benznidazole (BENZO) to its corresponding amine. Drugs were given to C3H mice 10 min before whole-body hyperthermia (WBH) in an incubator ($41 \pm 0.5°C$, core temp, for 45 min). Drug concentrations were measured by HPLC. WBH increased the plasma concentration of Ro 31-0313, the N-oxide metabolite of Ro 03-8799, during the heating period, e.g. by 63% at 40 min. Plasma levels of the MISO demethylation product, Ro 05-9963, were also increased by WBH shortly after the heating period, e.g. by 37% from 24.2 to $33.2\,\mu g\,ml^{-1}$ at 90 min. WBH reduced injected Ro 05-9963 plasma clearance by 15%, from 0.98 to $0.84\,ml\,g^{-1}\,h^{-1}$, but did not alter the plasma clearance of injected Ro 03-0313. *In vitro* rates of microsomal demethylation of MISO to Ro 05-9963 were increased by 20% at 41°C compared to 37°C. at a substrate concentration of 5 mM MISO. The demethylase enzymes were not markedly more labile at the higher temperature. In contrast, this 4°C rise decreased BENZO nitroreduction to its amine by 22% at a substrate concentration of 1 mM, possibly through increased denaturation of the nitroreductases. Thus mild hyperthermia clearly can affect drug metabolism, which has important implications for the pharmacokinetics and therapeutic effects of drugs used in thermochemotherapy.

### Novel drug metabolism and excretion studies using high resolution proton NMR spectroscopy

J.K. Nicholson[1], K. Tulip[1], J.A. Timbrell[1] J.R. Bales[2] & P.J. Sadler[2]

[1]*Toxicology Unit, The School of Pharmacy, University of London, Brunswick Square, London WC1N 1AW; and* [2]*Department of Chemistry, Birkbeck College, University of London, London WC1E 7HX, UK.*

High resolution $^{1}H$ NMR spectroscopy can be valuable in the study of normal excretory products present in urine (Bales *et al.* (1984), *Clin. Chem.* **30**, 426). We have investigated the use of this technique to study the renal excretion of a well-known drug paracetamol and that of the potential antitumour agent N-methylformamide (NMF), the metabolism of which is poorly understood. For paracetamol, $^{1}H$ NMR of urine was found to be quantitatively similar to conventional HPLC detection methods and that free drug together with glucuronide, sulphate, cysteinyl and N-acetylcysteinyl conjugates could be rapidly and simultaneously detected in untreated human urine samples after the ingestion of a normal therapeutic dose (1 g). After treatment of rats with $1\,g\,NMF\,kg^{-1}$, urinary excretion of the parent compound could be readily monitored by $^{1}H$ NMR and several metabolites including methylamine, formate, formamide, a cysteinyl derivative and several N-acetylated compounds were detected. Particular advantages of $^{1}H$ NMR in this type of study include its rapid multicomponent detection capability, lack of sample preparation and its non-destructive and non-equilibrium perturbing nature.

These studies indicate that $^{1}H$ NMR has considerable potential in the elucidation of the metabolic modification and excretion of novel therapeutic agents.

### The use of $^{13}C$-labelled carboxylic acids in the study of metabolic conjugation by NMR

J. Caldwell, B.P. Nutley, A.J. Hutt & M.V. Marsh

*Department of Pharmacology, St Mary's Hospital Medical School, London W2, UK.*

Stable isotope labelled compounds are extensively used in studies of xenobiotic metabolism, almost exclusively for mass spectrometric analysis. In comparisons, the opportunities for analysis by NMR presented by paramagnetic isotopes such as $^{13}C$ have been neglected. To maximize the value of such an approach the isotope must be close to a centre of interest whose chemical shift is altered by metabolism. The carboxyl carbon of xenobiotic acids is metabolically transformed to a range of products (esters, amides, olefins, ketones, secondary alcohols) in which its resonance varies over the range 40–200 ppm. We have examined the fates of carboxyl-$^{13}C$-benzoic, phenylacetic and cinnamic acids (all with $^{13}C$-isotope abundance >90%) and have used $^{13}C$-NMR in addition to other techniques to identify metabolites. The sensitivity of $^{13}C$-NMR at this level of enrichment is such that spectra of urine and crude extracts were recorded directly, without recourse to extensive purification procedures. This technique has further characterized minor metabolites of these acids, and is suggested to have wider applicability.

### Pharmacokinetic studies of thiotepa in patients and mice and effect of co-administration of nandrolone decanoate

B.J. McDermott, J.A. Double, M.C. Bibby, P. Loadman & R.L. Turner

*Clinical Oncology Unit, University of Bradford, Bradford BD7 1DP, UK.*

The synergism of the combination of nandrolone decanoate (ND) and ThioTEPA observed in the clinic (Turner *et al.* (1984), *Br. J. Cancer* **50,** 259) and in animal model systems (Double *et al.* (1981), *Br. J. Cancer* **44,** 305) could be explained in part by a pharmacokinetic interaction. Plasma levels of ThioTEPA and its primary metabolite, triethylenephosphoramide (TEPA), were estimated using capillary gas chromatography with nitrogen detection, in 11 patients who received 30 mg of drug by i.m. dosage and in 17 studies in mice after i.p. or s.c. administration of 20 mg kg$^{-1}$, with or without ND (50 mg kg$^{-1}$) given by i.p. injection in arachis oil (AO).

Values of the ratios under the plasma concentration versus time curves (AUC) of TEPA and ThioTEPA in patients (mean, 1.41; range, 0.20–2.46) and in mice (mean $\pm$ s.e., 2.05 $\pm$ 0.11) indicate that the clearance of TEPA is slower than that of ThioTEPA. When ND was administered concomitantly in mice, the clearance of the drug was not affected. The AUC value of TEPA (mean $\pm$ s.e., 8.57 $\pm$ 0.40 $\mu$g h ml$^{-1}$, however, increased significantly ($P < 0.01$) by comparison with the value when ThioTEPA was given alone (mean $\pm$ s.e., 7.10 $\pm$ 0.46 $\mu$g h ml$^{-1}$. When ThioTEPA was administered with AO but without ND, the mean AUC value of TEPA was 7.73 $\pm$ 0.38 (s.e.) $\mu$g h ml$^{-1}$, which was significantly different from the values obtained after dosage with ThioTEPA alone ($P < 0.05$) or in combination with ND ($P < 0.05$). Dose-dependence of AO on metabolite clearance was established. The contribution of AO to the reduced clearance of TEPA may be a consequence of inhibition of cretion of the polar metabolite into the peritoneum. The significant effect of ND on the elimination of TEPA could have relevance to the clinical studies.

### Nitrogen mustard selectively inhibits the $Na^+K^+Cl^-$ co-transporter and reduces cell volume in L1210 murine leukaemia cells

C. Wilcock, J.A. Hickman & S.B. Chahwala

*CRC Experimental Chemotherapy Group, Department of Pharmaceutical Sciences, University of Aston, Birmingham B4 7ET, UK.*

Studies on the interaction of antitumour drugs with tumour cell membranes may reveal new, susceptible targets for novel agents. We have shown previously that nitrogen mustard (HN2) inhibited the uptake of $^{86}Rb^+$ (a $K^+$ congener) into L1210 murine leukaemia cells in a time-dependent manner (*Br. J. Cancer* **50,** 274 (1984)). After 3 h incubation of $5 \times 10^6$ ml$^{-1}$ L1210 cells in RPMI at 37°C with 10 $\mu$M HN2, $^{86}$Rb uptake via the diuretic-sensitive $Na^+K^+Cl^-$ cotransporter alone was inhibited. A monofunctional analogue of HN2 had no effect at 10 $\mu$M nor at concentrations of equivalent cytotoxicity to HN2. In addition, other cytotoxic agents e.g. adriamycin, mitozolomide and *cis*-platin, had no effect on $^{86}Rb^+$ transport at equivalent cytotoxic concentrations. HN2 caused a decrease in cellular $K^+$, measured by atomic absorption, which was completely offset by a 25% decrease in cell volume, effectively maintaining $K^+$ homeostasis (118 $\pm$ 19 mM). The cells retained the ability to exclude trypan blue and maintain membrane potential, estimated by accumulation of |$^3$H|triphenylmethylphosphonium bromide ($-58 \pm 11$ mv). Under the same conditions HN2 inhibited, by 29%, the uptake of an amino acid via the $Na^+$-dependent 'A' system, but not via the $Na^+$-independent 'L' system. This was not a consequence of increased cellular |$Na^+$| which was maintained at control values (34 $\pm$ 17 mM), and was considered to be a result of direct alkylation of the transport system. HN2 had no effect upon the membrane enzyme $Na^+K^+$ATPase. We consider that the reduction in cell volume caused by HN2 may bring about events which lead to cell death.

### Effects of encapsulation of methotrexate in intact erythrocytes on its efficacy *in vivo*

H.O. Alpar & D.A. Lewis

*Department of Pharmaceutical Sciences, University of Aston, Birmingham B4 7ET, UK.*

By using a preswelling technique (Pitt *et al.* (1983), *Biochem. Pharmac.* **22,** 3359) methotrexate has been encapsulated into intact erythrocytes at a loading of 0.56 mg ml$^{-1}$ packed cells and an encapsulation efficiency of 28% w/v. When the encapsulated drug was administered to mice and the urinary excretion compared to that of free methotrexate the free drug was excreted rapidly and completely in three days whilst the encapsulated drug was excreted more slowly and 9% was still excreted on the fourth day. *In vitro* studies with both human and mouse cells confirmed that the rate of leakage of drug from the cells was slow. The encapsulated methotrexate was administered i.v. (2.8 mg kg$^{-1}$) to CBA/CA mice inoculated (i.p.) 4 days previously with TL $\times$ 5 cells. Other mice were treated with free methotrexate and controls left untreated. Free methotrexate increased the survival time of the mice by 33% but the encapsulted preparation increased the survival time by 70% compared with untreated controls ($P<0.001$) (Student $t$-test). In other work it was found that encapsulted asparaginase produced 'cures' in C3H mice bearing the 6C3HED tumour whilst the free enzyme did not. Therefore encapsulated has improved the efficacy of both antineoplastic agents.

**Potentiation of the cytotoxicity of chemotherapeutic drugs by Tween 80 *in vitro***

C.N. Parris, J.R.W. Masters, M.C. Walker & P.J. English

*Department of Histopathology, Institute of Urology, St Paul's Hospital, 24 Endell Street, London WC2, UK.*

There is evidence that the intravesical instillation of a non-ionic detergent, Tween 80 (polyoxyethylene sorbitan mono-oleate), together with adriamycin can overcome resistance to this drug in patients with superficial bladder cancer (Eksborg *et al.* (1982), *Eur. Urol.* **8**, 213). To extend this finding, the effect of Tween 80 on the cytotoxicity of the four drugs commonly used for intravesical chemotherapy: adriamycin, mitomycin-c, epodyl and thiotepa, was examined *in vitro* using the continuous cell line, RT112, derived from a transitional cell carcinoma of the human bladder. Inhibition of colony formation was determined following a 1 h exposure to a range of concentrations of these drugs alone and in combination with a non-cytotoxic concentration of Tween 80, 0.1%. Tween 80 enhanced the cytotoxicity of each drug at all concentrations tested. Colony forming ability, expressed as a percentage of control values, at one concentration of each drug is shown in the table.

It is concluded that the addition of Tween 80 might enhance the therapeutic potential of intravesical chemotherapy for superficial bladder cancer.

**Formulation of MZPES – The ethanesulphonic acid salt of *m*-azidopyrimethamine**

S.K. Wong[1], T.J. Schoemaker[2], M.F.G. Stevens[1] & J.A. Slack[1]

[1]*Cancer Research Campaign Experimental Chemotherapy Group, Department of Pharmaceutical Sciences, University of Aston, Birmingham B4 7ET, UK; and* [2]*Slotervaartziekenhuis, Medisch Centrum Slotervaart, Louwesweg 6, Amsterdam, The Netherlands*

*m*-Azidopyrimethamine ethanesulphonate (MZPES: 1) is a lipophilic (log $P$ 2.94) inhibitor of L1210 DHFR (Ki $2.4 \pm 0.16$ nM). The drug (free base) displays *in vivo* antitumour activity against mouse P388, L1210, B16, TLX5 and M5076 tumours. The pKa of MZPES is 7.19 close to physiological pH ensuring adequate concentrations of free base (for absorption) and N-1 protonated species (for enzyme inhibition).

(1) $R=N_3$
(2) $R=N$
(3) $R=NH_2$
(4) $R=NO_2$

The water solubility of the free base of MZPES is low ($0.02\,mg\,ml^{-1}$) at 20°C. For clinical trial the salt is sufficiently soluble ($13.9\,mg\,ml^{-1}$) for preparation of a parenteral formulation in water (unbuffered at pH 4.14) at a concentration of $10\,mg\,ml^{-1}$ and sterilised by filtration. Aqueous solutions of MZPES are photosensitive: in a nitrogen environment the arylamine (MAP: 3) is the major photoproduct whereas the nitroarene (MNP: 4) predominates in oxic conditions. The product distribution points to the intermediacy of a triplet nitrene reactive species (2) which either abstracts hydrogen to form MAP or traps (triplet) oxygen to afford MNP.

**Table**

| | *Adriamycin* $350\,ng\,ml^{-1}$ | *Mitomycin-C* $400\,ng\,ml^{-1}$ | *Thiotepa* $15\,\mu g\,ml^{-1}$ | *Epodyl* $40\,\mu g\,ml^{-1}$ |
|---|---|---|---|---|
| Drug alone | 65 | 46 | 58 | 56 |
| Drug + 0.1% Tween 80 | 15 | 17 | 22 | 46 |

### Arm oedema and breast cosmesis related to surgery in patients with breast cancer undergoing breast conservation techniques

A. Rodger[1] and U. Chetty[2]

*Departments of [1]Clinical Oncology and [2]Clinical Surgery, University of Edinburgh, UK.*

From June 1982 until November 1983 60 patients underwent breast conservation techniques by local tumour excision and radiotherapy for breast cancer. The first 37 consecutive patients also underwent lower axillary (Level I and II) dissection (LAD) while the next 23 underwent only axillary sampling (LAS). The two groups were similar for local T stage and node stage (clinical and histological). The median follow-up period was 19 months for the LAD group and 12 months for the LAS group. Clinically detectable arm oedema was recorded at any time after surgery in 59% of the LAD group and 4% of the LAS patients ($P=0.0388$). The median time of onset of arm oedema in the LAD group was 10 months, while the arm oedema appeared at 3 months in the one LAS patient. Breast oedema was recorded in 78% of LAD patients compared with 35% of LAS patients ($P<0.001$); and was persistent in 38% and 23% respectively (ns). The median onset of breast oedema was the same in both groups at 7 months. In the LAD group a central axillary radiation dose of 45 Gy was associated with a higher risk of arm oedema (67%) then a lower dose of less than 40 Gy (25%). In both groups the incidence of breast oedema was higher following the delivery of an interstitial implant radiation boost of 25 Gy or more when compared with 20 Gy but only reaches significance in the LAD group (90% v. 38% $P=0.028$). The incidence of arm oedema was not related to node histology. The extent of axillary surgery in patients undergoing this treatment influences post therapy cosmetic sequelae as measured by arm and breast oedema, and these effects may be aggravated by higher radiation doses.

### Increased detection of metastases from primary breast cancer to the axillary nodes by immunohistochemical staining with monoclonal antibodies

N. Berry[1], D.B. Jones[1], R. Marshall[1], J. Smallwood[2] & I. Taylor[2]

*Department of [1]Pathology and [2]Surgery, University of Southampton, UK.*

The most accurate prognostic indicator for a woman following surgical removal of a primary breast cancer is the presence or absence of metastases in the axillary lymph nodes. Detection of metastases from sections stained with haematoxylin and eosin (H & E) might be improved by immunohistochemical staining. In this study the breast and axillary tissue was studied from 45 women who had mastectomy at Southampton General Hospital in 1983–84. Four μm sections of paraffin embedded lymph nodes were stained in double-bridge immunoalkaline phosphatase assay with the monoclonal antibodies HMFG1, HMFG2 and E29/68. These antibodies recognise antigens on breast carcinoma cells and normal breast epithelium, but do not normally stain cells of the lymphoreticular system. Conventional diagnosis of the H&E stained sections detected metastases in 55/345 nodes. This was increased to 61 positive nodes by sectioning through the tissue at a deeper level, and to 66 positive nodes by using immunostaining with monoclonal antibodies.

Immunohistochemical staining improved the detection of metastases from 16/45 to 19/45 cases, an increase of 19%. It will be interesting to see if the increased detection of metastases has any bearing on the patients' survival.

### Abnormalities of cellular DNA content in human solid tumours

D. Hedley, M. Friedlander & I. Taylor

*Ludwig Institute for Cancer Research, University of Sydney, N.S.W. 2206, Australia*

The majority of human cancers are aneuploid, i.e. have an abnormal cellular DNA content, but the significance of this finding remains obscure. Recently we developed a flow cytometric method for measuring DNA content of paraffin-embedded tissues, and this has allowed us to study tumour ploidy in large number of patients whose outcome is already known. In 228 ovarian cancer patients the incidence of aneuploidy in Stage I and II disease was 57% and 69% for Stage III and IV. It was a powerful adverse prognostic feature ($P<0.001$) in all stages except Stage IV, where patients with diploid tumours had an equally poor survival. Out of 152 patients presenting with metastatic adenocarcinoma of unknown primary site 30% had diploid tumours, but their prognosis was no better than that of the aneuploid group (median survival 4.2 and 4.8 months, respectively). Finally, we have so far looked at 415 breast cancers, and again there is a correlation between aneuploidy and stage. Aneuploid tumours tended to be larger (59% of tumours < 2 cm, 81% of tumours > 2 cm, $P<0.02$) with more extensive axillary lymph node involvement ($P<0.05$). In Stage II disease relapse-free survival was longer in the diploid group ($P<0.05$), although this effect was largely confined to pre-menopausal patients where the projected 4 year disease free survival rate was 83% and 43% for diploid and aneuploid respectively. Following relapse, however, patients with diploid tumours did not live longer than those with aneuploid tumours. Taken together these studies show that the incidence of aneuploidy in at least some common solid tumours increases with disease stage. This may account for the generally more favourable prognosis for diploid tumours; when they do metastasise they are apparently as aggressive and refractory to treatment as aneuploid tumours.

**Effect of cholecystokinin on carcinoembryonic antigen release in patients with cholangiocarcinoma**

C. Hudd[1], J.E. Devine[2] & F.E. Johnson[1]

*Departments of [1]Surgery and [2]Pathology, St Louis University Medical Center, St Louis, MO, USA.*

Biliary tract cancer accounts for ~80 deaths weekly in the USA. This tumour usually presents late with little hope of cure. Earlier diagnosis might enhance survival. Cholecystokinin (CCK) administered to nude mice bearing human cholangiocarcinoma (CCa) xenografts has been reported to retard tumour growth and increase serum carcino-embryonic antigen (CEA) levels (Hudd *et al.* (1984), *Gastroenterology* **86**, 1118). We chose to study whether CCK challenge could be exploited as a diagnostic test in humans with CCa by provoking a rise in CEA. Patients with CCa were studied irrespective of performance status, prior treatment, or other factors. All such patients received 0.02 mg $kg^{-1}$ body wt of synthetic sulphated CCK-8 (Squibb) as an i.v. bolus. This is the dose recommended for CCK cholecystography in humans. Serum CEA levels were measured (Abbott CEA RIA) prior to CCK administration and 15 min, 60 min, and 24 h later. No ill effects were observed at this dose. The following day, CEA levels were measured at similar time intervals after saline control injection. Three consecutive patients were evaluated; none showed a change in CEA level in excess of 20%, which is the maximum expected coefficient of variation of the test. These three consecutive false-negatives are sufficient to establish, with a 99% probability, that this test is not 80% sensitive. We conclude that this provocative test using low-dose CCK has no value in the diagnosis of human CCa. Higher-dose CCK might be effective, but the risk of side effects would also be greater.

**Acetylation phenotype as a risk factor in bladder cancer**

A.V. Kaisary[1], P.J.B. Smith[1], R.A. Branch[2], C.B. McAllister[2] & A. Ray[3]

*[1]Department of Urology, Bristol Royal Infirmary, Bristol, UK; and Departments of [2]Clinical Pharmacology and [3]Biostatistics, Vanderbilt University, Nashville, Tennessee, USA.*

Acetylation has been linked to carcinogenic detoxification in aromatic amine induced bladder cancer. Slow acetylation phenotype was reported to be associated with bladder cancer more than rapid acetylator phenotype. A prospective case control study of acetylation phenotype was undertaken in the Bristol area. Ninety-five patients with histologically confirmed transitional cell carcinoma of the bladder were compared with a control group of 111 patients who were sex and age matched and had bladder cancer excluded. Sixty-two of the bladder cancer patients had non-aggressive tumours (G1 and G2) and the remaining 33 had aggressive ones (G3). The acetylation phenotype was estimated from the ratio of monoacetyl dapsone concentration to dapsone concentration in a plasma sample obtained 8 h after monoacetyl dapsone oral administration. Normit plots comparing this ratio in the control population with non-aggressive (G1 and G2) and aggressive (G3) bladder cancer population suggested a biphasic curve in each group. The distribution in the aggressive tumour group (G3) and the control group were similar. A minor difference was present when the non-aggressive tumour group (G1 and G2) and controls were compared. Slow acetylation phenotype was 49% of the control group and 65% of the non-aggressive tumour population (G1 and G2). Univariate statistical analysis confers a relative risk ratio of 1.93 on slow acetylators in comparison with rapid acetylators in this population. Slow acetylation phenotype association risk with bladder cancer is comparable to previous reports but this is the first time it is linked to non-aggressive rather than aggressive tumours. It appears that non-aggressive (G1 and G2) and aggressive (G3) bladder cancer have different aetiological factors and are possibly two different diseases.

**The inflammatory response following intralesional BCG immunotherapy: The relationship to Heaf status and time course**

R. Windle[1], I.C. Talbot[2] & E.H. Mackay[2]

*Departments of [1]Surgery and [2]Pathology, Royal Infirmary, Leicester, UK.*

Immunotherapy using intralesional BCG has been used in malignant melanoma (Morton *et al.* (1974), *Ann. Surg.* **180,** 635) and lung cancer (Holmes *et al.* (1979), *J. Thorax. Cardiovasc. Surg.* **7,** 362). However there is no clear indication which patients are most likely to develop a local inflammatory response. To investigate this we have studied patients undergoing excision of rectal carcinoma having previously had an intralesional injection of BCG.

Thirty-three patients were studied. BCG was administered between 4 and 16 days prior to surgery and the excised tumour and draining nodes were examined histologically for evidence of lymphocytic infiltration (LI) or reactive follicular hyperplasia (RFH) respectively or for granuloma formation and these were related to patient age, tumour differentiation, Heaf status and immune competence (PHA induced blastogenesis, LMIT and NK activity).

Within the tumour, granulomata were seen in 10 patients and LI was found in a further 10. Granulomata were significantly more frequent when the interval following BCG administration exceeded 10 days ($P<0.05$ Fishers exact test). All but 3 patients with an inflammatory response in the tumour were Heaf positive. Of the remaining 13 only 3 patients were Heaf positive ($P<0.025$ Fishers exact test). Tumour inflammatory response was not related to patient age or immune competence or tumour differentiation. Nodes from 21 patients demonstrated RFH and 5 had granulomata. There was no significant relationship with Heaf status but granulomata were only seen in nodes from Heaf positive patients. These results may have some bearing on the type of and timing of immunotherapy in cancer.

### Inter-regional epidemiological study of childhood cancer (IRESCC). Case-control studies in children with germ cell tumours

J.R. Mann, H.E. Johnston, J. Williams, J.A.H. Waterhouse, J.M. Birch, R.A. Cartwright, G.J. Draper, A.L. Hartley, P.A. McKinney, P. Hopton & C.A. Stiller, for the IRESCC Group

*Children's Hospital and West Midlands Cancer Registry, Birmingham, Departments of Epidemiology and Social Research, Manchester Department of Epidemiology, YRCO, Leeds, University of Oxford Childhood Cancer Research Group, UK.*

In 1980–83 members of IRESCC interviewed parents of 555 children with newly diagnosed cancer on a wide range of topics of possible aetiological significance. Identical questions were asked of the parents of 1100 control children chosen from hospital admissions and general practitioner lists. Medical information was confirmed whenever possible by cross-checking with NHS records. For the 41 children with germ cell tumours and their 82 controls no differences were shown for: birth weight and rank, maternal and paternal age and chronic illnesses and smoking, mothers' gynaecological histories and oral contraceptive usage. In index pregnancies there were no case-control differences for maternal illness, infections, alcohol intake, X-ray and ultra-sound exposure, but case mothers took slightly more analgesic and fewer hormones. More case than control mothers and fathers reported occupational exposure to chemicals. The cases had slightly more congenital malformations than controls, including one neural tube defect. More relatives of cases had malformations and multiple tumours than did control relatives. Interesting case pedigrees included one each of multiple twinning probable neurofibromatosis and XY gonadal dysgenesis.

### Pharmacokinetic study of high dose etoposide infusion in patients with small cell lung cancer

A. Tarpey[1], J.A. Green[2] & H.M. Warenius[2]

*Departments of [1]Biochemistry and [2]Radiation Oncology, Clatterbridge Hospital, Merseyside, UK.*

Etoposide is one of the most active agents in small cell carcinoma of bronchus, although the optimum dose and scheduling of the drug has not been determined. Previous studies have suggested that the peak plasma level is the most important and the drug is conventionally given as a 30 min infusion. This study comprises 8 patients given 2 cycles of single agent, high dose etoposide at 600 mg m$^{-2}$ daily for 3 days. All had received 2 prior cycles of ifosfamide 8 g m$^{-2}$ in the previous 8 weeks, but no other radio- or chemotherapy. Etoposide was assayed in plasma by HPLC using a Zorbax BP ODS column with u.v. detection at 225 nm. An internal standard of phenytoin was used and this gave a typical run time of <4 min. The method was linear up to 100 $\mu$g ml$^{-1}$ with a sensitivity below 1 $\mu$g ml$^{-1}$. The method was found to be precise and accurate. When etoposide was given for 6 h out of 24 h on each of 3 consecutive days peak plasma levels of 55 $\mu$g ml$^{-1}$ were detected (range 40–70). There was little difference between successive days within each cycle, or between 2 cycles given 4 weeks apart. Data on comparison with the same total dose given as a continuous 72 h infusion will be presented, as well as correlation with acute toxicity.

### Chemical and biological oxidation of the dimethoxyphenol ring of etoposide

J.M.S. van Maanen[1], C. de Ruiter[2], R. van de Straat[2], J. Broersen[2], J. de Vries[2] & H.M. Pinedo[1]

*Departments of [1]Oncology and [2]Medicinal Chemistry, Free University, Van der Boechorststraat 7, 1081 BT Amsterdam, The Netherlands*

Studies on the metabolism of etoposide (VP-16) have yet failed to identify active metabolites of the drug. Metabolic conversions in the dimethoxyphenol ring of VP-16 may be important for its interaction with DNA and, as a consequence, for its cytotoxicity. The purpose of our study was to investigate the possible chemical and biological oxidation of the dimethoxyphenol ring of VP-16. The chemical one-electron oxidant persulphate-ferrous and the enzymatic one-electron oxidants myeloperoxidase (MPO)/$H_2O_2$ and horseradish peroxidase (HRP)/$H_2O_2$ were found to catalyze the formation of a VP-16 free radical, as observed by electron spin resonance (ESR) spectroscopy. The ESR spectra were identical to the spectrum obtained on electrochemical oxidation of VP-16 at +550 mV. This indicates that the radical produced by the chemical and the enzymatic one-electron oxidants is formed at the phenolic position of VP-16. The chemical half-life of the free radical in 1 mM Tris pH 7.4 0.1 M NaCl was found to be $253 \pm 4$ sec. A purification of rat liver cytochrome P-450 and NADPH cytochrome P-450 reductase was performed to study metabolism of the dimethoxyphenol ring of VP-16 by reconstituted mixed function oxydase. Incubations of VP-16 with cytochrome P-450 and NADPH cytochrome P-450 reductase + NADPH (oxygenation) or with cytochrome P-450 and cumene hydroperoxide (peroxygenation) resulted in O-demethylation of VP-16. The product of O-demethylation could be the ortho-dihydroxy derivative or ortho-quinone of VP-16. In conclusion the formation of oxidative products in the dimethoxyphenol ring of VP-16 was observed, which could be important for its mechanism of action.

### Influence of Tween 80 on the pharmacokinetics, metabolism and urinary excretion of adriamycin in the cancer patient

G.J. Forrest[1], J. Cummings[2], D. Cunningham[1], R. Blackie[2], N.L. Gilchrist[1] & M. Soukop[1]

*[1]Department of Medical Oncology, Royal Infirmary, Glasgow G4 0SF; and [2]Department of Clinical Oncology, University of Glasgow G12 9LX, UK.*

Tween 80 is a safe pharmaceutical vehicle used to formulate poorly water soluble VP-16 for i.v. administration to cancer patients. We have been investigating what effects the Tween 80 itself can have on the handling of other anti-cancer agents given in combination with VP-16. In 6 patients adriamycin (ADR, 30 or 40 $mg\,m^{-2}$ i.v.), was given with cyclophosphamide (cyclo, 500 $mg\,m^{-2}$ i.v.) and Tween 80 (300 $mg\,m^{-2}$ i.v.). Then, 3 weeks later, only ADR and cyclo were administered to the same 6 patients. Samples were collected for 24 h; ADR and metabolites concentrations were measured by HPLC. Sera ADR profiles were best fitted to a bi-exponential decay descriptive of a two compartment open pharmacokinetic model ($t\frac{1}{2}\alpha$, $5 \pm 2$ min; $t\frac{1}{2}\beta$, $7 \pm 4.9$ h). The Tween 80 did not affect the apparent kinetic rate constants for distribution of ADR from the central compartment and for elimination of ADR from the peripheral compartment, nor were the area under the curve (AUC) of metabolite serum profiles affected. Where the Tween 80 did seem to exert an effect was on maximum serum concentration ($C_0$) and the apparent volume of the central compartment ($V_c$). $C_0$ without Tween was $5240 \pm 2436$ $ng\,ml^{-1}$; $C_0$ with Tween was $3065 \pm 1775$ $ng\,ml^{-1}$. In all patients $\bar{C}_0$ fell with Tween (by 13% to 413%). Consequently, $V_c$ increased (from $10.8 \pm 3.41$ to $23.2 \pm 14.8$ l) in all patients. Also 24 h urinary excretion of ADR was increased in all 6 patients by Tween 80 (by 15% to 500%). In conclusion, administration of VP-16 plus Tween 80 is likely to affect the handling of ADR: peak serum concentrations will fall, clearance will increase and more drug will be excreted unchanged in the urine.

### Pharmacokinetics of 4′-epi-doxorubicin after intravenous and intravesical administration

W.J.F. van der Vijgh[1], H. Weenen[1], A. Osterop[1], S.E.J.M. van der Poort[1], K.K. Kurth[2], J.F. Bogdanowicz[1] & H.M. Pinedo[1]

*[1]Department of Oncology, Free University Hospital, 1081 HV Amsterdam; and [2]Department of Urology, Erasmus University, 3000 DR Rotterdam, The Netherlands.*

4′-Epi-doxorubicin (E) is one of the 4′-modified doxorubicin analogues. Phase I trials have indicated that, compared to doxorubicin (D), E showed comparable toxicities but less gastrointestinal toxicity and less chronic cardiotoxicity. Possibly, it also has a broader spectrum of antitumour activity. The difference in biological activity between E and D may be caused by differences in metabolic and pharmacokinetic properties.

Seventeen patients with advanced soft tissue sarcoma or advanced breast cancer received an i.v. bolus injection of 75 or 90 $mg\,E\,m^{-2}$. Blood samples and urine were collected at regular time intervals. HPLC analysis revealed the presence of E and 7 of its metabolites in plasma (mean AUC of each metabolite as % of total AUC): E (24.0%), Eol (21.9%), E-glu (12.5%), Eol-glu (23.3%), D-one (0.5%), Dol-one (1.2%), 7d-D-one (5.4%) and 7d-Dol-one (11.2%). In urine E and 3 of its metabolites were present (% of dose excreted in 48 h): E (5.9%), Eol (0.8%), E-glu (3.2%) and Eol-glu (0.8%).

Five patients with carcinoma in situ of the bladder were treated with E. Thirty or 50 mg E dissolved in 50 ml saline was instilled in the bladder for 1 h. Blood samples and urine were collected during and after installation. At least 75% of the dose was recovered in urine, while low and undetectable concentrations of only E could be detected in plasma.

It can be concluded that (a) E shows unique and abundant glucuronidation, (b) only small amounts of E reach the general circulation after intravesical administration.

### The relationship between plasma 5-FU clearance and tissue levels of 5-FU metabolites

E.M. Chisholm, P.J. Finan, L.F. Woodhouse & G.R. Giles

*University Department of Surgery, St James's Hospital, Leeds, UK.*

5-Fluorouracil (5-FU) is commonly administered by i.v. bolus injection in doses of 10–15 mg kg$^{-1}$, but is rapidly catabolised by the liver. An improvement in therapeutic effect might be achieved either by altering drug elimination or changing the uptake by the tumour tissue. This study has examined the relationship between 5-FU metabolites within tissue and the measured clearance of 5-FU from the plasma after an i.v. bolus.

Eight patients with colorectal cancer received 5-FU (15 mg kg$^{-1}$) 48 h prior to surgery. Blood samples were taken at 5 min intervals for 1 h after injection. 5-FU levels in the blood and fluorinated metabolites within normal and neoplastic tissue were estimated using HPLC. Elimination curves for plasma FU revealed a half life ($t\frac{1}{2}$) which ranged from 8.6–25.1 min with all 8 patients having single compartment curves over 60 min. The plasma clearances were also variable and ranged from 0.65–1.54 dm$^3$ min$^{-1}$. By extrapolation of the elimination curve to time zero, the theoretical maximum concentration of 5-FU was calculated and ranged from 30.4–66.3 $\mu$g ml$^{-1}$. Total fluorinated products were estimated from the levels of FU, fluorodine and fluorodeoxyuridine in the specimens and ranged from 4.37–31.05 nmol ml$^{-1}$ supernatant of normal mucosa and 13.9–75.3 nmol ml$^{-1}$ supernatant of colorectal cancer. An analysis of the total fluorinated products against $t\frac{1}{2}$, plasma clearance and maximum plasma concentration failed to demonstrate any significant linear relationship ($r$=0.01, 0.004, 0.04 respectively). Similarly the ratios of FU metabolites in tumour to normal mucosa ranged from 0.8–10.55 and could not be correlated to the pharmacokinetics of 5-FU.

It is concluded that the cellular levels of active 5-FU metabolites reflect local cellular activity rather than patient handling of the drug.

### Modulation of 5-fluorouracil (FU) metabolism in two intestine cell lines: Relation to cytotoxicity

G.J. Peters, E. Laurensse, A. Leyva & H.J. Pinedo

*Department of Oncology, Free University Hospital, P.O. Box 7075, 1007 MB Amsterdam, The Netherlands.*

Initial metabolism of FU catalyzed by pyrimidine nucleoside phosphorylase (PNP) requires ribose-1-P (Rib-1-P) or deoxyRib-1-P (dRib-1-P) as cosubstrates. Modulation of their availability can elucidate the metabolic pathway and the mechanism of action of FU in particular tumour types. We used purine nucleosides to study the modulation of FU metabolism in two human cell lines, WiDr a colon carcinoma and Intestine 407 a transformed intestine cell line. Both lines showed comparable PNP activities with FU as substrate (about 2 nmol h$^{-1}$ 10$^{-6}$ cells). With inosine as precursor PNP activity was 30 and 86% in WiDr and Intestine 407, respectively, and with deoxyinosine 7 and 19%. A 2 h incubation with 1 mM inosine increased Rib-1-P concentration 2–4-fold, while incubation with deoxyinosine gave dRib-1-P levels comparable to those of Rib-1-P. In cell culture inosine did not affect cell growth and showed no synergism with FU. 1 mM deoxyinosine moderately inhibited cell growth (20–30%). With 0.1–1 mM deoxyinosine a synergism was found at non-toxic concentrations of FU (0.1–0.5 $\mu$M). This synergism was greater with Intestine 407 than with WiDr cells. Examination of the medium after 24 h showed that in both cell lines deoxyinosine was rapidly broken down to hypoxanthine (at 0.1 mM for 80 and 50% in Intestine 407 and WiDr, respectively). dIMP (at 0.4 and 1 mM) also served as a source of dRib-1-P and enhanced the cytotoxicity of FU. Cell growth inhibition of this combination could be reversed by 2 $\mu$M thymidine in Intestine 407 cells but only partly in WiDr cells. The results show that low non-toxic FU concentrations can be made cytotoxic by supplementary dRib-1-P. The effect of thymidine showed that inhibition of thymidylate synthetase appears to be a limiting factor.

### Measurement of BZQ (2,5-diaziridinyl-3,6-*bis*(2-hydroxyethylamino)-1,4-benzoquinone; NSC 224070) in human plasma by high-performance liquid chromatography (HLPC)

A.G. Bosanquet, P. Hunt & E.D. Gilby

*Department of Clinical Investigation, Royal United Hospital, Combe Park, Bath BA1 3NG, UK.*

BZQ is about to go into Phase I clinical trials in this hospital, and we have developed a method for extracting the drug from human plasma and measuring it by HPLC. Plasma (3 ml) was injected through a pre-wetted C-18 Seppak cartridge, and the cartridge washed with water (pH 8.5; all solutions used at 0°C). BZQ was eluted with MeOH, and the eluate diluted 1 to 2 with 52.5 mM $NH_4Ac$ before 200 $\mu l$ of this solution was injected into the HPLC. The mobile phase was 50 mM $NH_4Ac$:MeOH 7:3 (v/v) pumped at 0.8 ml $min^{-1}$ through a 5 $\mu m$ ODS-Spherisorb column and detected at 254 nm (u.v.) and +0.7 V in oxidative mode (electrochemical; EC). Minimum detection was ~20 ng $ml^{-1}$ by u.v. and 3 ng $ml^{-1}$ by EC. Recovery from plasma averaged 79% with good linearity over 0–100 ng $ml^{-1}$ BZQ ($r$=0.988). The half life ($t_{\frac{1}{2}}$) of BZQ in plasma at 37°C was 209 ±13 min (mean ± s.d.; $n$=3), slightly longer than in 0.05 M phosphate or bicarbonate buffers (pH 7) at 96 and 126 min respectively. Addition of albumin (40 mg $ml^{-1}$) to BZQ in 150 mM NaCl reduced the $t_{\frac{1}{2}}$ from 10 to 5.4 h (22°C), thus it is possible that the degradation of BZQ is protected by plasma lipoproteins. BZQ was stable for 5 h in plasma at 0°C, whereas freezing at −35°C resulted in slight decomposition. This is consistent with the fact that we have found BZQ to be unstable (? polymerises) when frozen in inorganic buffers.

### Comparative tissue distribution of platinum and $^{14}C$ in mice receiving $^{14}C$ carboplatin

S.E. Dible, Z.H. Siddik, F.E. Boxall & K.R. Harrap

*Department of Biochemistry and Pharmacology, Institute of Cancer Research, Sutton, Surrey, UK.*

Recent studies have identified carboplatin (JM8) as a viable alternative to *cis*-platin. The reactivity of carboplatin will depend upon the rate of removal of the 1,1-cyclobutane decarboxylate ligand. Hence the time course of this dissociation has been determined in a number of tissues, using $^{14}C$-carboplatin (*cis*-diammine-[1-$^{14}C$]-CBCDA Pt II). Female Balb $C^-$ mice received $^{14}C$-carboplatin (80 mg $kg^{-1}$; 1.1 mCi $kg^{-1}$, i.v.) and were exsanguinated at times ranging from 5 min to 5 days. Tissues were removed, solubilized in 0.5 ml hyamine hydroxide (44% w/v) overnight at 50–60°C, and diluted in 0.1 N HCl. Aliquots were analysed for Pt and $^{14}C$ using flameless atomic absorption and liquid scintillation techniques respectively. Up to 2 h after drug administration the ratio of $^{14}C$:Pt in tissues and plasma was comparable to that in the dose solution (51 dpm $ng^{-1}$ Pt), indicating that carboplatin was structurally intact. Thereafter, the ratio decreased progressively due to a greater rate of removal of $^{14}C$ than of Pt from the plasma and tissues. At 5 days, the ratios in these tissues were 2 (kidney, ileum), 4 (muscle), 8 (heart), 12 (plasma) and 26 (liver). Thus, at least 86–96% of the Pt species was not associated with $^{14}C$, liver being the exception (~50%). The half lives (h) for Pt during the terminal phase varied with each tissue, being 40 (plasma), 54 (ileum heart), 71 (kidney), 98 (liver) and 116 (muscle). The corresponding half lives for $^{14}C$ were similar for each tissue (~27 h). with liver being an exception (216 h). These results indicate that the metabolic handling of carboplatin varies between tissues.

### Concentration of platinum in human brain tumors after intravenous administration of *cis*-platin

J.G. McVie[1], M. Chatel[2], J.A.G. Punt[3], M.P.J.W. Sokal[3] & T. Dikhof[4]

[1]*The Netherlands Cancer Institute, Amsterdam, The Netherlands,* [2]*University of Rennes, France;* [3]*Royal Infirmary, Derby, UK; and* [4]*Cyclotron Unit, Eindhoven, The Netherlands for the EORTC Brain Tumour Group.*

In planning a protocol to test the activity of *cis*-platin as a radiosensitizer in malignant gliomas a pilot study was carried out with varying doses of intravenous *cis*-platin (C) prior to craniotomy and biopsies of normal brain and glioma were removed for sampling. Seven patients (pts) were treated with a bolus of C 4, 6 or 8 mg $m^{-2}$ i.v.; 6 pts with 60 mg $m^{-2}$ i.v. 30 min prior to operation. Platinum levels were measured by proton induced X-ray emission using a cyclotron generated proton beam. This technique is highly accurate and reproducable down to 0.5 ppm dry wt. There are no disturbances in the measurements due to presence of tissue in contrast to the conventional atomic absorption technique. Patients' samples treated with low doses of C showed no detectable platinum. The following values were achieved after 60 mg $m^{-2}$.

All biopsies were also examined histologically and patient *R** has no vital tissue in any of the 5 samples studied. The entire material was necrotic. As animal and clinical experience has indicated that ~3 ppm platinum is required to achieve a radio-sensitizing of platinum, it would appear that a dose of 60 mg $m^{-2}$ i.v. *cis*-platin would be appropriate for a trial of the drug together with radiotherapy in the treatment of malignant glioma.

**Table** Concentration of platinum (ppm dry wt)

| Patient | *R** | *GUI* | *TH* | *GOU* | *GUJ* | *CHR* |
|---|---|---|---|---|---|---|
| Normal brain | 0, 0 | 0 | 1, 1 | 1, 1 | 0 | — |
| Mixed histology | | | | | | 1, 6 |
| Tumour | 0, 0, 0 | 17 | 4, 5 | — | 13 | 3, 6, 7, 8 |

### Pharmacokinetics of high dose melphalan in children and adults

C. Ardiet, B. Tranchand, P. Biron, P. Rebattu & T. Philip

*Centre Léon Bérard 69373 LYON Cedex 08, France*

The plasma kinetics of melphalan (L phenylalanine mustard L-PAM) was investigated in 22 patients (13 children, 9 adults). This Phase II trial was conducted in bearers of advanced malignant tumours with high dose L-PAM (140 mg m$^{-2}$) as mono- or polychemotherapy, followed by autologous bone marrow graft. Parent L-PAM was assayed by means of HPLC using methanolic extraction with dansyl-proline as internal standard. Our results can be summarized as follows: In all cases, L-PAM pharmacokinetics closely followed a two compartment open model. In spite of standardized hydration and L-PAM rapid infusion, initial concentrations of drug were found highly scattered (range: 9.0 to 49.9 mg l$^{-1}$). The disposition ($\beta$) phases showed also a large dispersion; $t_{1/2\beta}$ ranging from 18 to 71 min) and $\beta$-phase drug concentrations at time 0 ranging from 1.2 to 8.0 mg l$^{-1}$ of plasma (mean: 4.6 mg l$^{-1}$). In all cases, the drug level was <0.1 mg l$^{-1}$ at 8 h, allowing if necessary an early bone marrow graft. CSF samples were drawn from 45 to 150 min after L-PAM injection in 11 patients; in only 4 out of these was the drug detectable. The remaining 7 showed no drug in CSF. No kinetic difference was found between children and adults. In 5 additional patients, drug incubation with plasma showed a variable hydrolysis rate; this parameter is presently to be related to the elimination phase in connection with linearity studies.

### Report on the Phase I trial of N$^2$, N$^4$, N$^6$-trihydroxymethyl-N$^2$ N$^4$, N$^6$-trimethylmelamine (trimelamol)

I.R. Judson, C.J. Rutty, G. Abel, L. Gumbrell, K.R. Harrap & A.H. Calvert

*Department of Biochemical Pharmacology, Institute Cancer Research, Sutton, Surrey, UK.*

Trimelamol is an anti-tumour *s*-triazine which unlike hexamethylmelamine and pentamethylmelamine (PMM) does not require metabolic activation. Preclinical studies predict both enhanced activity and reduced neurotoxicity compared with PMM (Newell *et al.* (1981), *Br. J. Cancer* **44,** 281). Eighteen patients (pts) at the Royal Marsden Hospital have so far been treated by rapid i.v. infusion at 3 weekly intervals with doses escalating from 25–2000 mg m$^{-2}$. Tumours treated were: ovary 9; mesothelioma 2; large bowel 3; lung 1; uterus 1; sarcoma 1; Hodgkin's disease 1. Toxicity has been far less than that reported for PMM and consists of (1) dose-related nausea and vomiting of <6 h duration, 76% of courses 280–1100 mg m$^{-2}$, all courses >1500 mg m$^{-2}$; (2) lethargy and anorexia of 1–2 weeks duration, most pts >1100 mg m$^{-2}$; (3) minimal myelosuppression, i.e. WBC nadir <2.0 × 10$^9$ l$^{-1}$ and platelets <100 × 10$^9$ l$^{-1}$ in only 1 pt at 1500 mg m$^{-2}$, fall in Hb>2 g dl$^{-1}$ in 2 pts after 3 courses 500–1100 mg m$^{-2}$. No abnormality of liver or kidney function attributable to the drug and no alopecia or neuropathy have been observed. The maximum tolerated dose is expected to be 1500–2500 ng m$^{-2}$, limited by sedation or myelosuppression. Anti-tumour effects comprise significant alleviation of pain in 3 pts, 2 differential responses (ovary), 1 static disease for 3 months (rectum) and 1 partial response (Hodgkin's). Phase II evaluation will follow.

### Preclinical and clinical studies with N$^2$, N$^4$, N$^6$-trihydroxymethyl, N$^2$, N$^4$, N$^6$-trimethylmelamine, an alternative to pentamethylmelamine

C.J. Rutty, I.R. Judson, G. Abel, M.A. Graham, A.H. Calvert & K.R. Harrap

*Department of Biochemical Pharmacology, Institute of Cancer Research, Sutton, Surrey, UK.*

The failure of pentamethylmelamine (PPM) in the clinic has been attributed to its lack of metabolic activation in man (Rutty *et al.* (1982), *Cancer Chemother. Pharmacol.* **8,** 105). For this reason, N$^2$, N$^4$, N$^6$-trihydroxymethyl, N$^2$, N$^4$, N$^6$-trimethylmelamine (trimelamol, CB 10-375), which does not require oxidative N-demethylation, has been developed as an alternative. Trimelamol has a number of advantages over PMM, in particular, its relative lack of acute neurotoxicity. Following this observation the penetration of PMM and trimelamol into mouse brain has been examined. Mice given 90 mg $kg^{-1}$ PMM i.p. showed high levels of parent drug in the CNS compared to plasma levels (brain:plasma = 0.93), whereas animals given an equivalent dose of Trimelamol had much lower levels of the N-hydroxymethylmelamine in brain tissue (brain:plasma = 0.063). CNS penetration appears to be related to partition coefficients (Poctanol/$H_2O$) for these two drugs which are 67 and 2.5 for PMM and Trimelamol respectively. However, it does not appear to correlate with plasma protein binding which is significantly greater for PMM (68.2%) than for Trimelamol (17.5%). A Phase I clinical study of Trimelamol (25–1800 mg $m^{-2}$) has shown this drug to be substantially less emetic than PMM. Since the nausea and vomiting associated with this class of compounds appears to be centrally mediated, these differences in toxicity may also be explained in terms of the different abilities of the two drugs to penetrate the CNS.

### A clinical pharmacokinetic study of LM985 and LM975

D.J. Kerr[1], S.B. Kaye[1], J. Cassidy[1], S. Dutta[1], A. Setanoians[1], G. Forrest[2], D. Cunningham[2], M. Soukop[2] & W.R. Vezin[3]

*Departments of [1]Clinical Oncology, University of Glasgow and [2]Glasgow Royal Infirmary; and [3]CRC Drug Formulation Unit, University of Strathclyde, Glasgow, UK.*

LM985 is a new chromone derivative which was selected for clinical trials because a high percentage of cures were seen in the murine colon tumour 38 as part of the NCI preclinical screen. A Phase I clinical and pharmacokinetic trial was initiated at the starting dose of 10 mg $m^{-2}$ (10% of the LD10 in mice) using a Q3W schedule. Treatment was initially by i.v. bolus, however transient hypotension was noted at 130 mg $m^{-2}$ and the drug was administered thereafter by a 1-h infusion. Blood samples were analysed by HPLC; p-dimethylamino-benzaldehyde was utilised as the internal standard, u.v. detection was at 303 nm and a 25 cm bondapack 5 $\mu$m C18 column was used. This system was found to be rapid (retention time < 10 min) and sensitive with good precision maintained down to concentrations of 5 ng $ml^{-1}$. *In vitro* studies indicated rapid degradation of LM985 (I) to LM975 (II) by $OH^-$ catalysed first-order hydrolysis. *In vivo*, model independent pharmacokinetic data (dose range 40–800 mg $m^2$; 19 patients) show rapid degradation of I to II (the end of infusion ratio of II/I varies from 10–40). I and II decline biexponentially with respective $\beta t\frac{1}{2}$ of 0.85 h and 2.2 h. It would appear that I acts as an unstable prodrug *in vivo* and that rapid hydrolysis converts it to II, which may be the active principle.

### Clinical pharmacology of sodium butyrate in patients with acute leukaemia

A.A. Miller

*Innere Klinik und Poliklinik (Tumorforschung), West German Tumor Center, University of Essen, D-4300 Essen 1, FRG*

Butyric acid induces differentiation of leukaemic cells *in vitro* (Novogrodsky *et al.* (1983), *Cancer* **51**, 9–14). We undertook a study of the clinical pharmacology of sodium butyrate (Na-B) in 6 patients with acute leukaemia. The dosage was 500 mg $kg^{-1}$ 24 $h^{-1}$ (isotonic solution) given as continuous i.v. infusion for 10 days. Blood samples were drawn daily during and hourly after infusion. We developed a sensitive method employing high-performance liquid chromatography (HPLC) after derivatization of butyrate in order to monitor its plasma concentrations. $^{14}C$ labeled Na-B was used as standard. Plasma was ultrafiltered for 45 min at 2000 *g* (MPS-I; Amicon, Witten, FRG). To 300 $\mu$l of the ultrafiltrate, 10 $\mu$l of 1 M $KHCO_3$ were added. The sample was brought to dryness under a stream of $N_2$. The residue was dissolved in 100 $\mu$l reaction mixture and 500 $\mu$l $CH_3CN$. The reaction mixture consisted of 0.2 M 2,4'-dibromoacetophenone and 0.2 M dicyclohexyl-18-crown-6 (20:1, v:v; Merck, Darmstadt, FRG). The sample was heated at 80°C for 30 min in a shaking water bath. After centrifugation, the clear solution could be directly injected into the HPLC. The separation was achieved on a $\mu$Bondapak C18 column (Waters) with $H_2O$:$CH_3CN$ 550:450 (v:v) as solvent under isocratic conditions (2 ml/min). The UV-detector was set at 354 nm. The retention time of Na-B was 12 min. Mean plasma concentrations of Na-B ranged from 59 to 79 $\mu$M during infusion. After infusion, the plasma curve showed an exponential decay with a half-life of 6.1 min. Although high doses of Na-B were given, the concentrations known to induce differentiation *in vitro* were not reached *in vivo*; no response and no toxicity were seen.

The organisers wish to thank the following companies for their generous support of the meeting: Roche Products Ltd.; Smith, Kline & French Research Ltd.; ICI Plc; Ciba-Geigy Pharmaceuticals; Bristol-Myers Company; Lederle Ltd.; The British Technology Group; May & Baker Ltd.; Beecham Pharmaceuticals.